# Does proximity of women to facilities with better choice of contraceptives affect their contraceptive utilization in rural Ethiopia?

**DOI:** 10.1371/journal.pone.0187311

**Published:** 2017-11-13

**Authors:** Solomon Shiferaw, Mark Spigt, Assefa Seme, Ayanaw Amogne, Stein Skrøvseth, Selamawit Desta, Scott Radloff, Amy Tsui, Dinant GeertJan

**Affiliations:** 1 Department of Reproductive Health and Health Service Management, School of Public Health, College of Health Sciences, Addis Ababa University, Addis Ababa, Ethiopia; 2 Maastricht University (Netherlands), Department of Family Medicine, Faculty of Health, Medicine and Life Sciences, Maastricht, The Netherlands; 3 Mela Research Plc., Addis Ababa, Ethiopia; 4 Department of Mathematics and Statistics, UiT The Arctic University of Norway, Tromsø, Norway And Norwegian Centre for E-health Research, University Hospital of North Norway, Tromsø, Norway; 5 Bill and Melinda Gates Institute for Population and Reproductive Health, Bloomberg School of Public Health, Johns Hopkins University, Baltimore, Maryland, United States of America; National Academy of Medical Sciences, NEPAL

## Abstract

**Background:**

There is limited evidence of the linkage between contraceptive use, the range of methods available and level of contraceptive stocks at health facilities and distance to facility in developing countries. The present analysis aims at examining the influence of contraceptive method availability and distance to the nearby facilities on modern contraceptive utilization among married women in rural areas in Ethiopia using geo-referenced data.

**Methods:**

We used data from the first round of surveys of the Performance Monitoring & Accountability 2020 project in Ethiopia (PMA2020/Ethiopia-2014). The survey was conducted in a sample of 200 enumeration areas (EAs) where for each EA, 35 households and up to 3 public or private health service delivery points (SDPs) were selected. The main outcome variable was individual use of a contraceptive method for married women in rural Ethiopia. Correlates of interest include distance to nearby health facilities, range of contraceptives available in facilities, household wealth index, and the woman’s educational status, age, and parity and whether she recently visited a health facility. This analysis primarily focuses on stock provision at public SDPs.

**Results:**

Overall complete information was collected from 1763 married rural women ages 15–49 years and 198 SDPs in rural areas (97.1% public). Most rural women (93.9%) live within 5 kilometers of their nearest health post while a much lower proportion (52.2%) live within the same distance to the nearest health centers and hospital (0.8%), respectively. The main sources of modern contraceptive methods for married rural women were health posts (48.8%) and health centers (39.0%). The mean number of the types of contraceptive methods offered by hospitals, health centers and health posts was 6.2, 5.4 and 3.7 respectively. Modern contraceptive use (mCPR) among rural married women was 27.3% (95% CI: 25.3, 29.5). The percentage of rural married women who use modern contraceptives decreased as distance from the nearest SDP increased; 41.2%, 27.5%, 22.0%, and 22.6% of women living less than 2 kilometers, 2 to 3.9kilometers, 4 to 5.9 kilometers and 6 or more kilometers, respectively (p-value<0.01). Additionally, women who live close to facilities that offer a wider range of contraceptive methods were significantly more likely to use modern contraceptives. The mCPR ranged from 42.3% among women who live within 2 kilometers of facilities offering 3 or more methods to 22.5% among women living more than 6 kilometers away from the nearest facility with the same number (3 or more methods) available after adjusting for observed covariates.

**Conclusions:**

Although the majority of the Ethiopian population lives within a relatively close distance to lower level facilities (health posts), the number and range of methods available (method choice) and proximity are independently associated with contraceptive utilization. By demonstrating the extent to which objective measures of distance (of relatively small magnitude) explain variation in contraceptive use among rural women, the study fills an important planning gap for family planning programs operating in resource limited settings.

## Introduction

Family planning allows couples and individuals to attain their desired number of children and determine the spacing between pregnancies. Ensuring access to a wide range of contraceptive methods that are affordable is essential for securing the well-being and autonomy of women, while supporting the health and development of communities [1–3]. While there is sufficient literature that suggests that contraceptive use is affected by the supply environment [4–6], there is little evidence of the linkage between contraceptive use, the range of methods available and stock status of facilities using geo-referenced data in developing countries [7].

Studies have shown that the lack of method availability and choice in addition to poor quality of care are barriers to the adoption of contraceptives. Moreover, the literature suggests that these contribute to discontinuation among those who already use contraception. For example, Blanc et al. showed that across 15 sub-Saharan countries within a year of starting a method, 7–27% of women ceased to practice contraception for reasons related to the quality of the service environment [8].

With a population of more than 94 million in 2017, Ethiopia is the second most populous country in Africa (next to Nigeria [9]) with relatively low literacy levels with 57% of rural women having no formal education [10].

Although falling short of its own goal of 44% by 2015 [11], Ethiopia has demonstrated significant progress in family planning uptake in recent years with a steady increase in modern contraceptive use among married women 15 to 49 years from 8 percent in the year 2000 to 37.3 percent in the year 2016 and decline in unmet need from 37 percent to 24 percent in the same timeframe [12, 13]. According to the healthcare financing reforms of the Federal Ministry 2005, family planning services provided by primary health care units (health centers and health posts) should be given free of payment for all people regardless of willingness to pay [14].

As part of its efforts to monitor progress in health, the Government of Ethiopia has been conducting, every five years, the Demographic and Health Household Surveys–DHS-(since 2000) and Facility Assessment of Reproductive Health Commodities and Services in the past two years. However, available data so far did not allow investigation of the extent to which there is a spatial relationship between women’s contraceptive use and the availability and types of contraceptive methods in nearby health facilities since the sample enumeration areas were not linked geographically for this purpose.

This analysis uses data from the Performance Monitoring and Accountability (PMA2020) and not the DHS. PMA2020 conducts nationally representative household and service delivery points (SDP) surveys to monitor family planning indicators in African and Asian countries using mobile phones [15]. It has a unique advantage of allowing direct examination of the effect of distance from health facilities on contraceptive usage among women in the same EA from which the SDP sample was selected.

The present analysis is aimed at responding to the following research question:

- What is the association between availability and range of contraceptives and distance to the nearby facilities on an individual woman’s modern contraceptive use among married rural women?

The study intends to generate important information on the extent to which recent stock outs and range of contraceptives available may affect the woman’s likelihood of being a current user. More importantly, the spatial relationships will enable visualizing the parts of the country where access to and availability of services are problematic, as contrasted with the typical analyses that are constrained to specific administrative boundaries and units. Modern contraceptives include injectables, implants, Intrauterine Devices (IUD), Pills, condom (male and female), male and female sterilization, standard days method and lactational Amenorrhea Method.

### Conceptual framework for analysis

**Fig 1:**
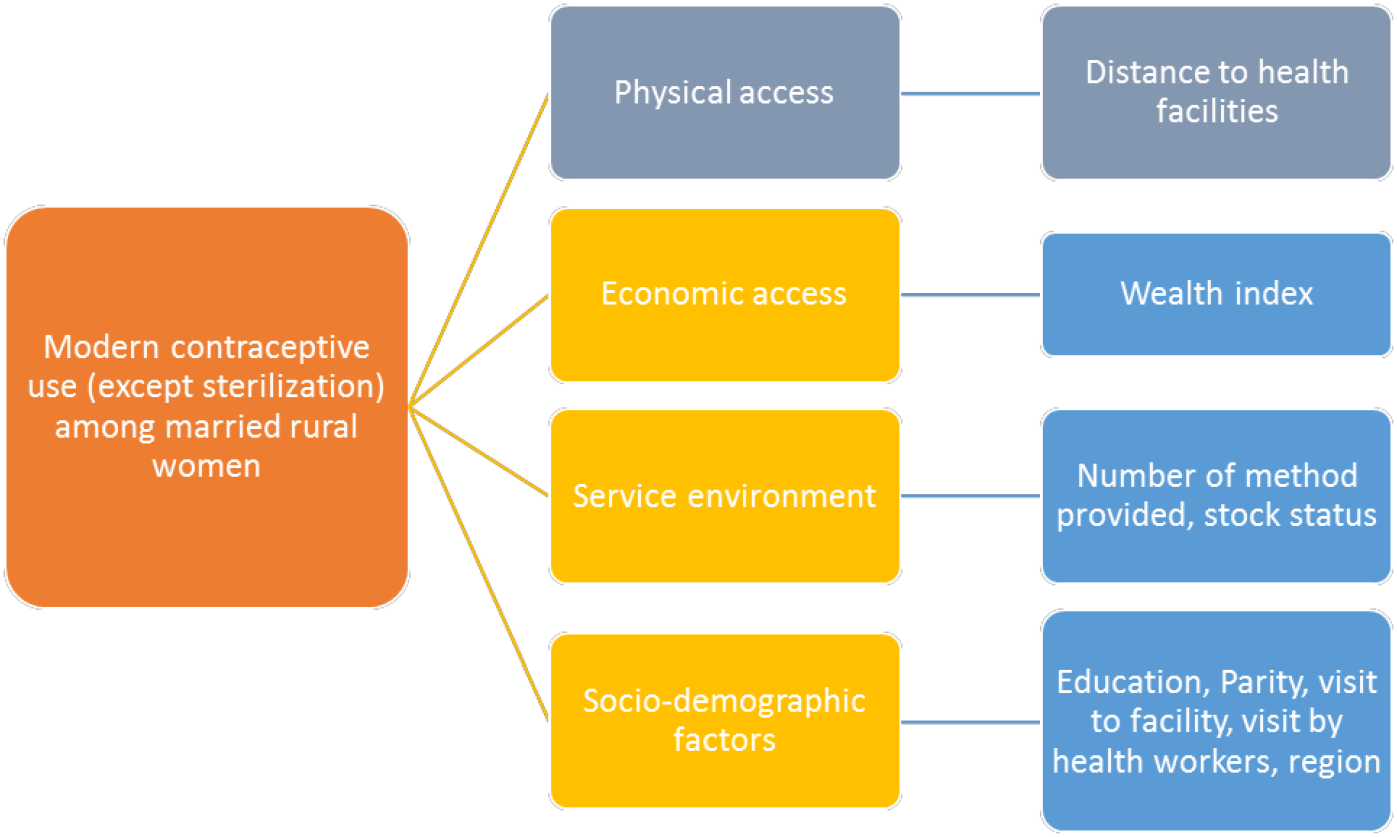
Conceptual framework for analysis.

Fig 1 below shows the relationship between the main outcome variable—modern contraceptive use—and its key influences such as physical access (as measured by straight-line distance between women's household and the nearest health facility), range of methods provided and in-stock status of contraceptives in health facilities and socio-demographic correlates, including household wealth status, educational status, parity, a recent visit to a health facility, and region.

## Methods

### Study setting

PMA2020/Ethiopia used a two-stage cluster design with urban-rural and major regions as strata. Ethiopia has 9 regions and 2 cities; namely Oromiya, Amhara, Tigray, and Southern Nations, Nationalities and Peoples (SNNP), Addis Ababa city administration, Afar, Benishangul-Gumuz, Dire-Dawa city administration, Gambella, Harari, and Somali. By design, the survey allows for the generation of regional indicators for the five big regions including Oromiya, Amhara, Tigray, SNNP, and Addis Ababa city administration. The remaining 6 regions were grouped into one ‘Other’ region.

### Sample size and sampling strategy

For each cluster (or EA), 35 households and three public service delivery points (SDPs); namely, a health post, health center and hospital as assigned by the government were selected to participate in the study.

Households were systematically sampled using random selection. Households with eligible females of reproductive age (15–49) were contacted and consented for interviews. For the SDP survey, participants were management staff at the selected health facilities who answered on behalf of the facility.

The primary health care unit in Ethiopia is comprised of a health center and these are typically surrounded by five satellite health posts. Each health post serves approximately 5,000 people and the five satellite posts together total a catchment population of 25,000 people. All these people, 25,000, can receive higher level care from each health center.

The PMA2020 survey was led by the School of Public Health at Addis Ababa University under the direction and technical support of the Bill and Melinda Gates Institute for Population and Reproductive Health, Johns Hopkins Bloomberg School of Public Health, Baltimore, Maryland, USA in collaboration with the Federal Ministry of Health and the Central Statistical Agency.

### Data management and analysis

Overall, the study surveyed at total of 6514 women 15–49 years and 378 SDPs, both public and private facilities, from a sample of 200 enumeration areas was drawn by the Central Statistical Agency from its master sampling frame.

The analytic sample for the present study was restricted to the 1,763 married women and 198 SDPs in rural areas with complete information (including geo-coordinates) as distance plays a more minimal role in urban settings. All surveys asked data collectors to collect geo-coordinates of every selected household and health facility, but due to poor network connectivity in some parts of the country it was not possible to always capture this information. Moreover, the capital city, Addis Ababa was excluded from the analysis as it is exclusively urban. Owing to the scarcity of private facilities in rural areas [16], the final regression analysis was limited to public health facilities including health posts, health centers and hospitals.

Three questionnaires were used: the household, female and service delivery point questionnaires. The majority of the questions on the household and female surveys were adapted from the Demographic and Health Surveys (DHS). The female questionnaire gathered specific information on education; fertility, family planning use, choice, and service experience, among others. The health facility module had information on the type of facility, the availability and types of contraceptives in and out of stock at the time of the interview and at any point in the last 312 months. GPS readings were taken at the time and location of the household and SDP interviews.

All PMA2020 questionnaires were administered using Open Data Kit (ODK) software and Android smart phones. The questionnaires appeared in three local languages (Amharic, Afan Oromo, and Tigrigna) in addition to English on the phone. Data collection was conducted between January and March, 2014.

This analysis excludes permanent contraceptive users (sterilization and vasectomy) as they may not represent current choice and use of contraceptives among women. The main outcome variable is current use of a modern contraceptive method among married women in rural Ethiopia. The exposure variables include distance from the woman’s household to the nearest health facility, her age, household wealth quintile, educational status, parity, region, whether she visited a health facility in the past 12 months and discussed family planning, whether she was visited by a health worker in the past 12 months, and the number (including stock out levels) of contraceptive methods offered by the facility in the past 12 months.

Stata version 14.2 was used for the analysis. Distance between SDPs and women's houses was measured using the Stata command gen D = sqrt(((lat_hosp-lat_hh)*110)^2+((long_hosp-long_hh)*110)^2). ArcGIS software version 10.4 was used to visualize the distribution of facilities with method stock-out as well as the mCPR levels by region and cluster. Point distance between women's houses and the nearest health facility was measured as a proxy for physical access to family planning services because of lack of complete information on road networks. The multivariate models were estimated using logistic regression methods.

The project proposal received ethical and technical approval by the Institutional Review Boards of the Ethiopian Health and Nutrition Research Institute (EHNRI 6.13/02) and the College of Health Sciences at Addis Ababa University (075/13/SPH).

## Results

Overall, complete information was obtained from 1,763 married rural women 15–49 years and 198 public service delivery points in rural areas (97.5% public) with 27 hospitals, 91 health centers, and 75 health posts. Close to three fourths of the respondents were less than 35 years of age with 61% having parity between one to four children. More than 2 out of 3 women had no formal education. See Table 1 for details.

**Table 1:** Background characteristics for rural married women age 15 to 49.

Characteristic	Total	Percent
Age group	n	%
15–24	404	22.9
25–34	731	41.5
35–44	500	28.4
45–49	128	7.3
Parity		
None	145	8.2
1–2	472	26.8
3–4	458	26.0
5 plus	688	39.0
Region		
Amhara	241	13.7
Oromiya	445	25.2
SNNP	385	21.8
Tigray	434	24.6
Other	258	14.6
Education		
Never	1,246	70.67
Primary	459	26.04
Secondary and higher	58	3.29
Household wealth quintile		
Lowest	365	20.7
Second	551	31.3
Middle	384	21.8
Fourth	356	20.2
Highest	107	6.1
**Total**	**1,763**	**100.0**

**Fig 2:**
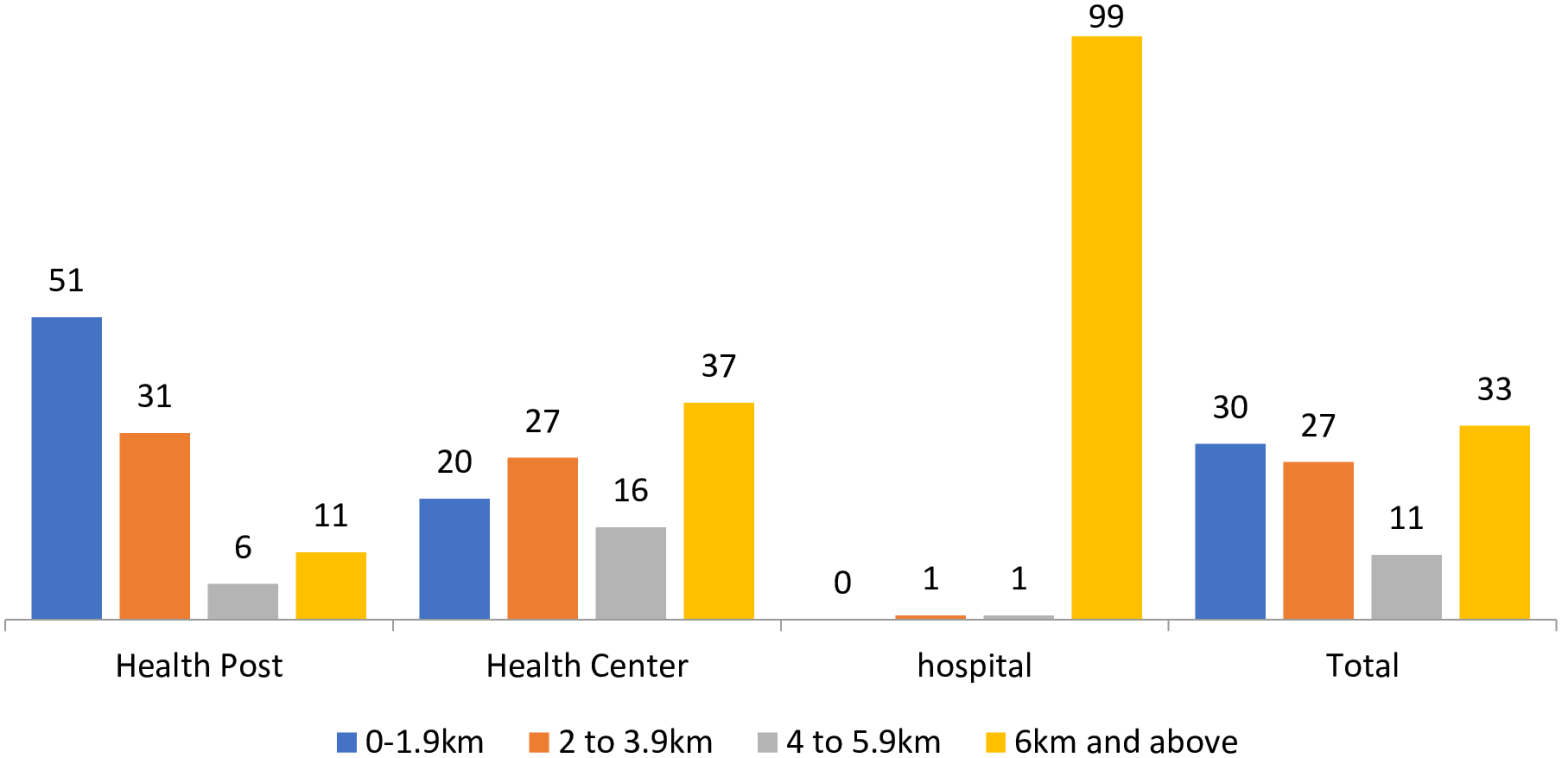
Percentage distribution of married rural women according to distance to health facilities, PMA2020/Ethiopia, 2014.

The main sources of modern contraceptive methods for married rural women were health posts (48.3%) and health centers (39.3%) (S1 Table). A majority of respondents (82%) lived within 4 kilometers of the nearest health post, while 47% lived within the same distance of the nearest health centers and 1% for hospitals. Overall, one-third of the sample population lived, on average, more than 6 kilometers from any of the nearest public health facilities. See Fig 2 below.

The mean number of modern methods offered in health facilities varied by type, with hospitals providing the greatest selection of methods available at 6.2 followed by health centers (5.4 methods) and health posts (3.7 methods) (S2 Table).

### Level and distribution of modern contraceptive users

The modern (non-permanent) contraceptive use rate (mCPR) among rural married women was 27.3% (95% CI:25.3, 29.5). The bivariate analysis showed the percentage of rural married women who use modern contraceptives decreased as distance from the SDP increased: 41.2%, 27.5%, 22.0%, and 22.6% for women living less than 2 kilometers, 2 to 3.9 kilometers, 4 to 5.9 kilometers and 6 or more kilometers, respectively (p-value<0.01). See details in Table 2.

**Table 2:** Factors influencing contraceptive use—Results from multivariate logistic regression analysis.

Characteristic	mCPR	OR (95%CI)		
		OR	Lower bound	Upper bound
**Age group (ref = 15–24)**	29.9	1.00		
25–34	27.5	0.78	0.53	1.16
35–44	29.0	0.77	0.48	1.24
45–49	18.6	**0.36**	**0.18**	**0.73**
**Parity (ref = None)**	18.3	1.00		
1–2	30.9	**2.62**	**1.45**	**4.75**
3–4	28.5	**2.83**	**1.48**	**5.44**
5 plus	27.6	**2.98**	**1.51**	**5.85**
**Region (Ref = Oromiya)**	18.8	1.00		
Amhara	43.7	**3.92**	**2.71**	**5.67**
Tigray	23.9	1.13	0.73	1.76
SNNP	38.7	**2.59**	**1.67**	**4.02**
Other^+^	10.9	0.74	0.47	1.17
**Education (ref = Never)**	26.1	1.00		
Primary	30.1	**1.60**	**1.18**	**2.16**
Secondary & above	45.8	**2.27**	**1.13**	**4.58**
**Wealth quintile (ref = Lowest)**	20.2	1.00		
Second	25.3	1.24	0.86	1.79
Middle	27.1	1.21	0.83	1.78
Fourth	33.4	**2.06**	**1.40**	**3.03**
Highest	49.2	**2.49**	**1.38**	**4.52**
**Distance to facility (ref = 0–1.9 kilometers)**	41.2	1.00		
2 to 3.9 kilometers	27.5	0.74	0.53	1.02
4 to 5.9 kilometers	22.0	**0.57**	**0.36**	**0.91**
6 kilometers and above	22.6	**0.62**	**0.43**	**0.88**
**Visited a health facility in past 12 months (ref = No)**	20.9	1.00		
Yes	36.8	**1.70**	**1.32**	**2.18**
**Number of contraceptives offered by facility (ref = 0 to 2)**	6.6	1.00		
3 to 4 methods	34.2	**2.96**	**1.35**	**6.49**
5 or more methods	25.7	**2.91**	**1.35**	**6.28**

^+^ = Group estimate for the 6 smaller regions including Benishangul-Gumuz, Gambella, Afar, Harari, Dire Dawa and Somali. Statistically significant values highlighted in **bold.** Ref = Reference category

**Fig 3:**
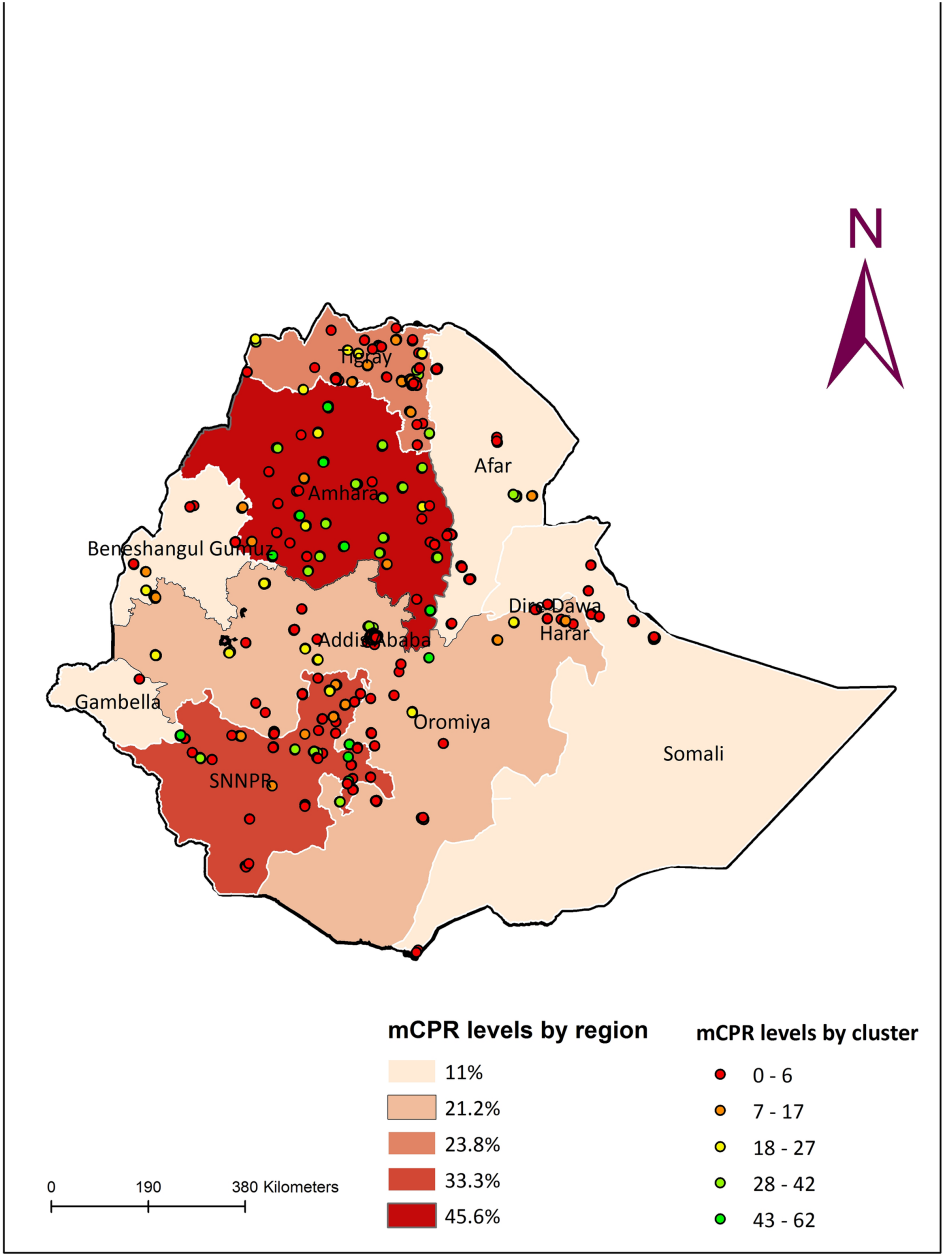
Spatial distribution of clusters with high and low rates of mCPR and regions according to levels of mCPR, 2014.

As shown in Fig 3 below, there is marked variation in the level of modern contraceptive use within and between regions with the majority of contraceptive users found in the Amhara region (43.7%) followed by SNNP (38.7%), Tigray (23.9%) and Oromiya (18.8%).

A more in-depth analysis of the distribution of clusters with high and low contraceptive use indicated that the majority of clusters having a modern contraceptive prevalence above 40% were in the Amhara and SNNP regions while nearly all clusters in the Somali and Afar regions had a prevalence less than 20%.

### Levels and spatial distribution of facilities with stock-out of contraceptives

**Fig 4:**
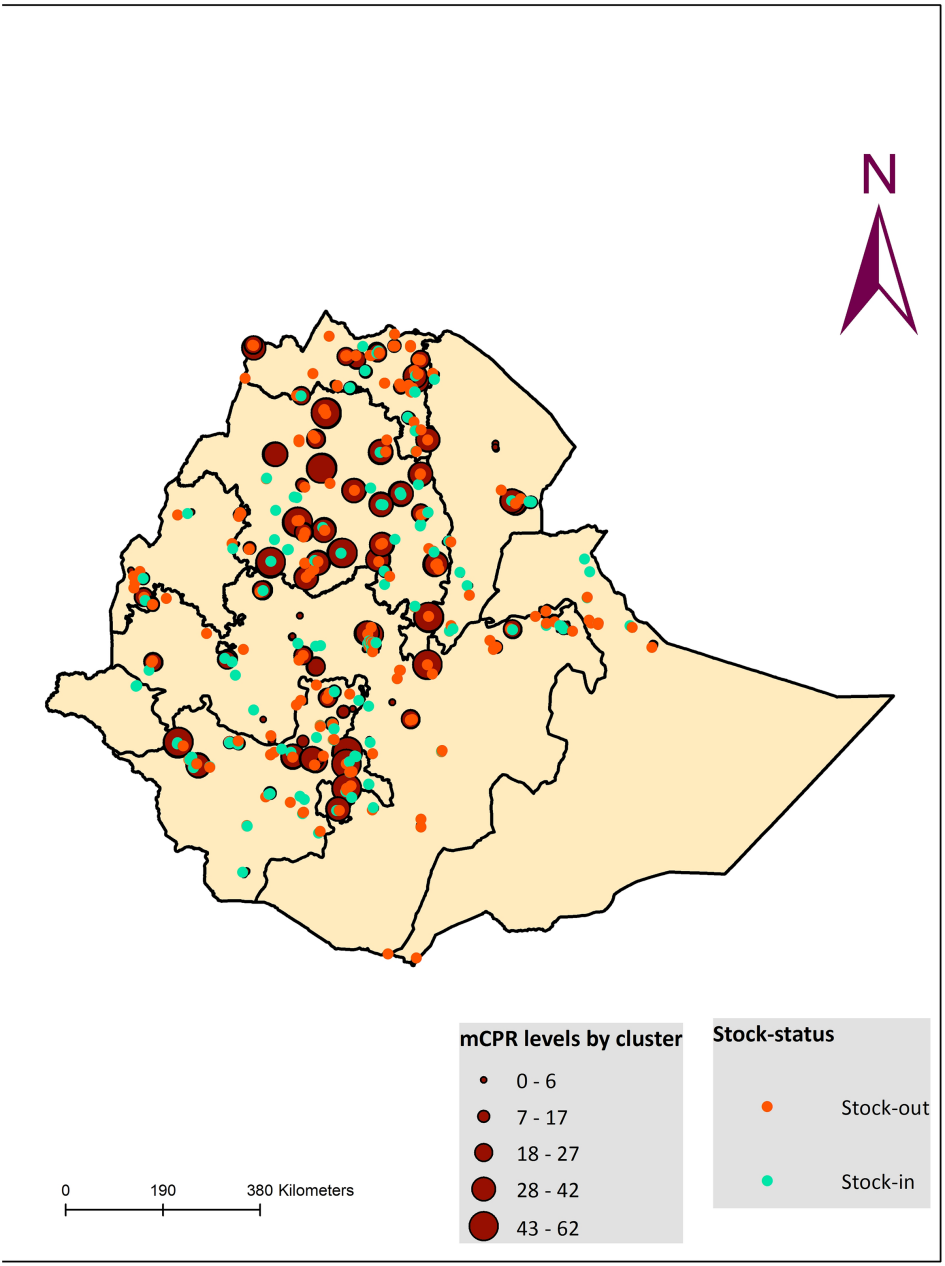
Spatial distribution of facilities with stock-out of contraceptives and clusters according to levels of MCP, 2014.

Overall, stock-out percentage among all health facilities offering at least one modern contraceptive method in the 12 months preceding the survey was 58.0%. In the following Fig (Fig 4), we show the distribution of facilities with any recent stock-out (defined as a facility having at least one method out of stock in the past 12 months). Stock-out levels in SDPs were relatively higher in Amhara and SNNP regions at 66.7% and 65.8% respectively compared to Tigray (61.5%) and Oromiya (51.6%), and while these did not differ significantly, the levels were high nonetheless (S3 Table).

### Factors influencing contraceptive use

As shown in Table 2, women who lived close to health facilities and facilities which offer wider choice of contraceptives, had significantly higher odds of using contraceptives after adjusting for observed covariates including age, parity, wealth, education, and number of methods offered by the facility. In addition, those who lived in the Amhara and SNNP regions and visited a health facility in the 12 months preceding the survey also had significantly higher odds of using modern contraceptives compared to the Oromiya region.

## Discussion

The PMA2020 survey platform allowed us to collect nationally representative household data that could be linked to the health facility environment through a geographic information system. This was important to avoid the usual challenges of misclassification errors associated with attempts to link independent facility samples and household data [17] providing the opportunity to examine both supply and demand side factors affecting contraceptive utilization. The approach also helped to visualize patterns of contraceptive use along with range of methods available and stock-out of contraceptives throughout rural Ethiopia.

The results showed that modern contraceptive use was significantly higher among women who lived relatively close to facilities (less than 2 kilometers) that have a wider range of contraceptives available, women with higher parity, higher educational status, living in households in the wealthiest quintile, and those who visited health facilities in the 12 months preceding the survey. The finding on access and proximity to facilities is consistent with previous studies which demonstrated that distance plays a key role in determining physical access to family planning services and other maternal health services using geo-referenced information [18–22].

There was also substantial geographic variation in contraceptive use and stock-out of contraceptives in health facilities by region, with the Amhara and SNNP regions representing areas with high contraceptive use. We found that women who lived beyond walking distance of health facilities (more than 4 kilometers) were significantly less likely to use modern contraceptives compared to those who lived within 2 kilometers of health facilities. It is worth noting that the constructed categories the distance to health facilities for women in this analysis could affect the results unless they are based on the actual distribution of distance as well as considerations of proximity for travel by walking.

One potential limitation of our analysis is the fact that we used measures of Euclidean distance, which are straight line distances between households and health facilities, and therefore do not take into account access to roads and travel time. However, in the absence of complete and up-to-date road network information, it might be prudent to use Euclidean distances as the difference between the two is likely to be minimal [23]. It is also important to note that women may not necessarily use the nearest facilities for several reasons and our analysis did not take into account which facilities women commonly use, or if they were in fact using the nearest public health facility.

In conclusion, the present study shows the importance of capitalizing on the availability of geo-coordinates of households and health facilities to inform programmatic and policy decisions pertaining to improving supply chain systems as well as demand and use of contraceptives. Distance to the nearest health facility and availability of a range of contraceptives methods and limited frequency of stock outs were associated with higher odds of modern contraceptive use after adjusting for important confounders including wealth index, parity, age, educational status, visit by health worker and visit to a health facility about family planning. By demonstrating the extent to which objective measures of distance (of relatively small magnitudes) are associated with contraceptive use among rural women, the study fills an important information gap for family planning program managers operating in resource-limited settings. Our study also helps to emphasize the importance of focusing interventions aimed at ensuring a reliable supply of contraceptives and expansion of range of contraceptive methods and services to areas with limited physical access to health facilities.

As a follow-up to the present analysis, we suggest that future studies attempt to measure the impact of uninterrupted availability and expansion of range of contraceptives as well as construction of new health facilities to improve contraceptive security for more rural women in Ethiopia.

## Supporting information

S1 Table Distribution of married rural women in Ethiopia according to source of contraceptives, 2014. (DOCX)

S2 Table Mean number of contraceptive methods provided by facility, 2014. (DOCX)

S3 Table Distribution of facilities with a stock-out of at least one contraceptive method by region, 2014. (DOCX)

S1 File Questionnaires. (ZIP)

## Abbreviations

AAU: Addis Ababa University
EA: Enumeration Area
mCPR: Modern Contraceptive Rate
PMA2020: Performance Monitoring and Accountability 2020 project in Ethiopia
SDPs: Service Delivery Points
SNNP: Southern Nations, Nationalities and Peoples
