# Correction: Does proximity of women to facilities with better choice of contraceptives affect their contraceptive utilization in rural Ethiopia?

**DOI:** 10.1371/journal.pone.0192258

**Published:** 2018-01-29

**Authors:** Solomon Shiferaw, Mark Spigt, Assefa Seme, Ayanaw Amogne, Stein Skrøvseth, Selamawit Desta, Scott Radloff, Amy Tsui, Dinant GeertJan

The affiliation of the ninth author is incorrect. Dinant GeertJan is not affiliated with #4 but with #2 Maastricht University (Netherlands), Department of Family Medicine, Faculty of Health, Medicine and Life Sciences, Maastricht, The Netherlands.
