# Supplementary material for: Does proximity of women to facilities with better choice of contraceptives affect their contraceptive utilization in rural Ethiopia?
Source: PLoS One. 2017 Nov 13;12(11):e0187311. doi: 10.1371/journal.pone.0187311 (PMC5683563; doi:10.1371/journal.pone.0187311)
Supplement: S1 Table — (DOCX) [file pone.0187311.s001.docx]

**Supplemental Table 1 - Distribution of married rural women in Ethiopia according to source of contraceptives, 2014**

| **Source of contraceptive methods** | **n** | **Percent** |
| --- | --- | --- |
| Govt Health Post | 277 | 49.6 |
| Govt Health Center | 224 | 40.1 |
| Private hospital/clinic | 31 | 5.6 |
| Govt Hospital/Polyclinic | 12 | 2.2 |
| Pharmacy | 7 | 1.3 |
| Family planning clinic | 6 | 1.1 |
| NGO/Shop/market | 2 | 0.4 |
| **Total** | **559** | **100** |
