# Supplementary material for: Does proximity of women to facilities with better choice of contraceptives affect their contraceptive utilization in rural Ethiopia?
Source: PLoS One. 2017 Nov 13;12(11):e0187311. doi: 10.1371/journal.pone.0187311 (PMC5683563; doi:10.1371/journal.pone.0187311)
Supplement: S2 Table — (DOCX) [file pone.0187311.s002.docx]

**Supplemental Table 2 – Mean number of contraceptive methods provided by facility, 2014**

| **Type of Public Facility** | **Mean** |
| --- | --- |
| hospital | 6.2 |
| Health center | 5.4 |
| Health Post | 3.7 |
| **Total** | **4.8** |
