# Supplementary material for: Does proximity of women to facilities with better choice of contraceptives affect their contraceptive utilization in rural Ethiopia?
Source: PLoS One. 2017 Nov 13;12(11):e0187311. doi: 10.1371/journal.pone.0187311 (PMC5683563; doi:10.1371/journal.pone.0187311)
Supplement: S3 Table — (DOCX) [file pone.0187311.s003.docx]

**Supplemental Table 3 – Distribution of facilities with a stock-out of at least one contraceptive method by region, 2014**

| **Region** | **% of facilities with stock-out of at least one method** |
| --- | --- |
| Tigray | 61.5 |
| Amhara | 65.8 |
| Oromiya | 51.6 |
| other | 48.0 |
| SNNP | 66.7 |
| **Total** | **58.0** |
