# Supplementary material for: Does proximity of women to facilities with better choice of contraceptives affect their contraceptive utilization in rural Ethiopia?
Source: PLoS One. 2017 Nov 13;12(11):e0187311. doi: 10.1371/journal.pone.0187311 (PMC5683563; doi:10.1371/journal.pone.0187311)
Supplement: S1 File — (ZIP) [file pone.0187311.s004.zip › Questionnaires/Afan Oromo version/PMA2020-FQ_Afan Oromo 13_11_2013.docx]

| **mADDS –Gaaffilee Dubartootaa** |
| --- |

| **LAK** | **GAAFIIFI CALALEESITUU** | | | | **KOODII** | | | | | | | | | | **DARB** |
| --- | --- | --- | --- | --- | --- | --- | --- | --- | --- | --- | --- | --- | --- | --- | --- |
| **EENYUMEESSITUU**  **Gaafiifi deebii jalqabuu keessan dura eenyumeesituu armaan gadii guutaa** | | | | | | | | | | | | | | | |
| A | | Maatii sirri/filatamee keessa jirta?  Fakkiin suuraan mana kanaa?.  *ODKn suuraa dubartootaa gaaffiif deebii dubartootaaf filataman kan maatii kana wajjin walqabatan nimul’isa.* | | | Eeyyee 1  Miti 0 | | | | | | | | | |  |
| B | | Gaafi fii deebii adeemsiisuuf maatii kana bira sia meeqa dhuftan? | | | Yeroo 1^ffaa^ 1  Yeroo 2^ffaa^ 2  Yeroo 3^ffaa^ 3 | | | | | | | | | |  |
| C | | Maqaa gaafatichaa: maqaan kun kan keetii?  Yoo kan kee miti ta’e maqaakee barreessi  *ODKn maqaa lakkoofisa bilbilaa wajjin walqabatee in agarsiisa.* | | | Eeyyee 1  Miti 0 | | | | | | | | | |  |
|  |  |  |  |  |  | | | | | | | | | |  |
| D | | **GUYAA FI SA’AATI AMMAA**  Guyyaa fi sa’aattin kun sirriidhaa? | | | Eeyyee 1  Miti 0 | | | | | | | | | | Eeyyeef gara G.F |
| E | | Guyyaa fi sa’aatii sirrii ta’e barreessi | | | Guyyaa | Guyyaa | Ji’a | | | | Waggaa | | | |  |
|  |  |  |  |  | Sa’aati | Sa’aati | Daqiiqaa | | | | AM/PM | | | |  |
| F | | Odeeffannoon armaan gadii kun kan gutamu galmee maatii irraa’i. Gaaffatama sirrii ta’ee argachuu kee mirkaneeffadhu.  *ODKn Naannoo,aanaa, ganda,lakkoofisa iddo qorannoo, lakkoofisa gamoo/oddo fi lakkofisa manaa galmee maatii irrat galmeefame kan gaaffi dubartiittin walsimatu nimul’isa* | | |  | | | | | | | | | |  |
| G | | Deebii keennitu tana haammam beekta? | | | Baay’ee baay’ee isi beeka 1  Baay’ee ishi 2  Baay’ee ishee hin beeku 3  Ishee hinbeeku 4 | | | | | | | | | |  |
| H | | Gaaffatamtuun guyyaa har’a mana jirti akasumas gaafatamuuf qophiidhaa? | | | Eeyyee 1  Miti 0 | | | | | | | | | | Mitif gara G>M |
| **ODEFANNO WALIGALTEE**  **Gaaffilee kanaaf dubartii umriin ishee waggaa 15-49 ta’e filadhu. Gaffilee kana baka namni kamiyyuu arguu hindandeenyeeti adeemsiifamu qaba. Waligaltee armaan gadii dubbsiif.** | | | | | | | | | | | | | | | |
| Akkam oltan/bultan. Maqaan koo________________________________jedhama. Ministeerii Fayyaa fi Univaarstiin Finfinnee dhimoolee fayyaa adda addaarraatti qoranoo adeemsiisa jiru. Anis isaan wajjin hojjachaan jira. Isiniis qorannoo kanarratti hirmaachuu keessaan isin galateeffanna. Odeeffannoo qorannoo kanarra argamuu mootummaan tajaajila fayyaa keennuu akka sirriitti karoorfatu gargaara. Odeeffannoo/deebii kennitan hundinu miseensoota qorannooo kanaattin ala namni kamiyyuu akka hinargine icccitiidhaan qabaman.  Oorannoo kana irratti hirmaachun kan feedhii keessan irrati hundaa’e yammuu ta’u gaaffii deebiisuu hinbarbaane yoo isin mudatee akka deebistan waan hindirqamneef natti himaatoo gara gaaffii itti aanuutti nidarbina akasumas gaaffif deebii yeroo kamiyyuu addaan kutuu nidandeenyaa garuu hirmaanaan keessan baay’ee barbaachiisaa waan ta’eef gaaffiif deebii akka gaggeesinu akka nuuf eeyyamtan niabdana.  Ammaa qorannoo kana ilaalchisee gaaffii nagaaffatan qabdu? | | | | | | | | | | | | | | | |
| I | | Koopii waligaltee itti kennudhaan ibsa godhiif. Itti aansuun gaaffii fi deebii eegaluu danda’aa jeechuudhaan gaafadhu? | | | Eeyyee 1  Miti 0 | | | | | | | | | | Mitiif gara G.M |
| J | | **Mallattoo deebii kenniitu**  **DEEBII KEENNITUUN QORANNOO IRATTI HIRMAACHUUF WALIGALUU KAN MIRKANEESU AKKA MALLATTEESAN YKN SANDUQAA KEESSA MALLATTO AKKA KA’AN GAAFADHU.** | | | MALLATTOO:  Check box: ☐ | | | | | | | | | |  |
| K | | Maqaa gaaffataa  **GAAFFATAMTUUN WALIGAlUU ISHE RAGAA BAHUUF MAQAA KEE BARREESSI**  **.** | | |  | | | | | | | | | |  |
| L | | **Maqaa deebii keenniituu**  **MAQAA DEEBII KEENIITU QOOFA BAREESSI** | | |  | | | | | | | | | |  |
| **LAK** | | **GAAFIIFI CALALEESITUU** | | | **KOODII** | | | | | | | | | | **DARB** |
| **Kutaa 1 Odeeffannoo Waligalaaa Deebbii keeniitu**  **Ammaa gaaafilee muraasa waa’ee haawasadiinagddee illalchisee sigaafadha.** | | | | | | | | | | | | | | | |
| 1 | | Umriin kee waggaa meeqa?  **WARA UMRIIN WAGGAA 15-49 TA’E QOFAATU GAAFATAMU QABA. YOO UMRIIN ISAANI KANA ALA TA’E GAAFII FI DEEBII XUMURII.** | | | Umrii waggaa xumurteen | | | | | | |  | | |  |
| 2 | | Barumsa hanga kutaa/sadarkaa hagamii hordoofte? Sadarkaa jalqabaa,lamaffaa,teekniikaafi ogummaa,ykn ol’aanaadha? | | | Gonkuma hin barane 0  Sadarkaa Jalqabaa 1  Sadarkaa Lamaffaa 2  Teeknikaafi Ogummaa 3  Sadarkaa Olaanaa 4 | | | | | | | | | |  |
| 3 | | Yeroo amma heerumtee /buultii qabatee/ jirta moo dhiira (hiriyaa) wajjin akka dhirsaafi nitiitti jiraacha jirtaa?  **MITI YOO TA’E,GAAFATAMTUU KAN WALHIKTEE,ADDAAN BAATE FI KAN IRRA DU’EE AKKA TA’E GAAFADHUU** | | | Gonkumaa hin heerumne 0  Eeyyeen,gaa’ela keesan jira 1  Eeyyeen,dhiira wajjin jiraacha jira 2  Kan wal hikte/adaan baatee 3  Kan irraadu’e 4 | | | | | | | | | | 0 yoo ta’e gara G8 |
| 4 | | Kan heerumtee/ akka dhirsaaf niiiti kan wallin jiraatee si’a meeqa? Al tokko qofa moo sana oli? | | | Al tokko qofa 1  Tokkoo ol 2 | | | | | | | | | | 1 yoo ta’e gara 5a 2 yoo ta’e gara G.5b |
| 5a | | Dhiirsa kee ( hirriyaa kee) waliin yoomi jiraachuu eegalte? Ji'a fi bara kam keessaa? | | | Ji’a : | | | | Q 5b | | | | | |  |
|  |  |  |  |  | Waggaa: | | | |  | | | | | |  |
| 5b | | Amma waa'ee dhiirsa (hiriyaa) kan ammaa yoom walin jiraachuu akka jalqabdee sigaaffadha.  Isa wajjin jiraachuu kan eegaltee ji'a fi bara kam keessaa? | | | Ji’a: | | | |  | | | | | |  |
|  |  |  |  |  | Waggaa: | | | |  | | | | | |  |
| 6 | | Abbaa manaa kee(hiriyyaan kee) haadha manaa biraa /dubartii biro kan wajjin jiraatuu niqaba? | | | Eeyyee 1  Miti 0  Hin beekuu -88 | | | | | | | | | |  |
|  | | **GD.3 ILAALLI/MIRKANEEFADHU :** Yeroo ammaa gaa’ela irra jirti? | | | Eeyyee 1  Miti 0 | | | | | | | | | | 0 yoo ta’e gara G.8 |
| 7 | | Dhiirsi kee (hiriyaan kee) yeroo ammaa si wajjin jiraata moo bakka biraa jiraata? | | | Wajjin jiraata 1  bakka biraa jiraata 2 | | | | | | | | | |  |
| **Kutaa 2 Walhoormaata,ulfaafi baay’ina ijoollee barbaadaman**  **Amma waa’ee baayi’na dahuumsaa umrii kee keesatti raawwatteen si**  **gaafadha**.**.** | | | | | | | | | | | | | | | |
| 8 | | Hanga ammaa si’aa meqaa daa’imma deessee jirta?  **‘0’n DEEBII TA’U NI DANDA’A** | | | Baay’ina dahuumsaa | | | | | | |  | | ‘o’f Gara G 13 | |
|  | | Yeeroo deesee kana hundaa daa’ima lbbuu qabu/du deesee ture?  **DEEBIIN MITI YOO TA’E GARA GD8 DEEB’UDHAAN LAKOOFSA DAA’IMMAA LUBUUN DHALATAN QOFA GALMEESSI** | | | Eeyyee 1  Miti 0 | | | | | | | | |  | |
| 9 | | Dahuumsii kee ini dhumaa yoom ture?  **GUYYAA DAYUUMSA ISA DHUMAA GALMEESSI**  **GUYYAA DAHUMSA SEENAA ARMAAN DURA JIRU WAJJIN WALQABSIISUDHAAN GAAFACHUUN BARBAACHIISAADHA** | | | Ji’a | | | Waggaa | | | | | | Waggaa darbe keessa miti yo ta’egara G11 ykn//fi waggaa bdarbe yoo hin taane ykn G.8 deebii 1 yoo ta’e | |
| 10 | | Dayumsa yeroo dhumaaf/xumuraaf dura jiru yoom deesse?  **GUYYAA DAYUUMSA YEROO XUMURAA DURA JIRU GALMEESSAA**  **GUYYAA DA’UMSA SEENAA ARMAAN DURA JIRU WAJJIN WALQABSIISUUN GAAFFACHUUN BARBAACHIISAADHA** | | | Ji’a | | | Waggaar | | | | | |  | |
| 11 | | Daa’imniidhuma irraati deesse lubuun jira/rti? | | | Eeyyee 1  Miti 0  Hin beeku -88 | | | | | | | | | Eeyyeen yoo ta’e gara G13 | |
| 12 | | Daa’imni dhuma irraati deesse yoom du’e/te?    **GUYYAA DAA’IMNI DHUMAIRRATI DEESSE DU’E/DUTE GALMEESSI**  **GUYYAA DU’AA SEENAA ARMAAN DURA JIRU WAJJIN WALQABSIISUUDHAAN GAAFACHUUN BARBAACHIISAADHA** | | | Ji’a | | | Waggaa | | | | | |  | |
| 13 | | Laguu/adaffii isa dhuumaa kan argitee yoomi?  **GUYYOOTA,TORBEE,JI’OOTA FI WAGOOTAAN YOO DEEBISAN MALLATTOO ‘X’ BAKKA KEENNAMEETTI GALCHAA** | | | Guyyoota dura | | | | |  | | | |  | |
|  |  |  |  |  | Torbee dura | | | | |  | | | |  |  |
|  |  |  |  |  | Ji’oota dura | | | | |  | | | |  |  |
|  |  |  |  |  | Wagoota dura | | | | |  | | | |  |  |
|  |  |  |  |  | Laguu arguu dhiisu /gadaameessii baasuu 1  Dayumsa xumura dura 2  Laguu argee hinbeeku 3 | | | | | | | | |  |  |
| 14 | | Ati yeroo amma tana ulfaa? | | | Eeyyee 1  Miti 0  Hin beeku/yaadadhuu 2 | | | | | | | | | ‘o’ yoo ta’e gara g.16 | |
| 15 | | Yeroo amma ulfa ji'a meeqaati?  **BAAY’INA JI’OOTAA GALMEESSI. KAN HINBEEKNE YOOTA’E ‘-88’ GALMEESSI.** | | | Baay’ina ji’oota | | | | | | |  | |  | |
|  | | **GD.14 ILAALLI/MIRKANEEFADHU:** Yeroo ammaa ulfa? | | | Eeyyee 1  Miti 0 | | | | | | | | | 16a yoo miti ta’e fi  16b eeyyee yoo ta’e | |
| 16a | | *Amma waa’ee fuldura gaaffilee muraasa sigaafadha*  Daa’imma/Daa'imma biro/ turtee da'uu barbaa moo daa'imma (daa'imma biroo) dahuuu hin barbaaddu? | | | Daa’imma/Daa’imma biro/ ni barbaadda 1  Gonkummaa/Daa’imma biro/ hinbarbaaddu 2  Ulfaa’u/dahuu hindanda’u jeete 3  Hin beeku/hin murteesine -88 | | | | | | | | | Skip to 17a if 1 and 18 for all other | |
| 16b | | **Amma waa’ee fulduraa gaaffilee muraasa sigaafadha**  Ulfa amma gaaraadhaa qabdu erga deessee booda , daa'ima biraa dahuu ni barbaadaa moo hinbarbaaddu? | | | Daa’imma/Daa’imma biro/ ni barbaadda 1  Gonkummaa/Daa’imma biro/ hinbarbaaddu 2  Ulfaa’u/dahuu hindanda’u jeete 3  Hin beeku/hin murteesine -88 | | | | | | | | | Skip to 17b if 1 and 18 for all other | |
| 17a | | Amma irraa jalqabdee (daa'ima/daa'ima biraa) osoo hin dahiin hangam turuu barbaadaa?  **JI’A YKN WAGGAA YOO FILATEE ISKRINII ITTI AANUU IRRATTI LAKKOOISA NI GALCHITA** | | | Ji’oota: | | | | | | |  | |  | |
|  |  |  |  |  | Wagoota: | | | | | | |  | |  |  |
|  |  |  |  |  | Ammumaa 1  Kan biroo 2  Ulfaa’u/dayuu hindandeessu 3  Hin beeku -88 | | | | | | | | |  |  |
| 17b | | Ulfa amma gaaraadhaa qabuu erga deessee booda , daa'ima biraa osoo hin dahiin hangam turuu barbaadaa?  **JI’A YKN WAGGAA YOO FILATEE ISKRINII ITTI AANUU IRRATTI LAKKOOISA NI GALCHITA** | | | Ji’oota : | | | | | | |  | |  | |
|  |  |  |  |  | Wagoota: | | | | | | |  | |  |  |
|  |  |  |  |  | Ammumaa 1  Kan biroo 2  Ulfaa’u/dahuu hindanda’u jeete 3  Hin beeku Don’t know -88 | | | | | | | | |  |  |
|  | | **GD 8 ILAALLI:** Baay’ina dahumsa?  **GD 14 ILAALLI**: Yeroo amma ulfa? | | | Baay’ina dayumsaa | | | | | | |  | | Skip to 19 if 0 births and 14: No.  Skip to 18a if 14: no and 18b if 14: yes | |
|  |  |  |  |  | Eeyyee 1  Miti 0 | | | | | | | | |  |  |
| 18a | | **Amma waa’ee daa’ima isa dhuumaa deessee ilaalchisee gaaffi sigaafadha.**  Yeroo ulfooftee sanaa yerumasanatti ulfaa’u barbaadaa turte?, moo turtee ulfaa’u barbaadaa moo, daa'imma sana(daa'imma biroo) ulfaa’u/dahuu hin barbaadnee? | | | Yerooma sanatti 1  Turee ulfaa’u 2  Ulfaa’u/dahuu hin barbaadnee turee 3 | | | | | | | | |  | |
| 18b | | **Amma immoo ulfa yeroo ammaa kana ilaalchisee gaaffi sigaafadha.**  Yammuu ulfooftee sanaa yerooma sanatti ulfaa’u barbaadee turtee? Moo turtee ulfaa’u barbaadee moo, ulfaa’y/ dahuu hin barbaadnee? | | | Yerooma sanatti 1  Turee ulfaa’u 2  Ulfaa’u/dahuu hin barbaaduu turee 3 | | | | | | | | |  | |
| **Kutaa 3 Karoora Maatii**  **Amma ati/abbaa manaan kee ulfa tursiisuudhaaf ykn haambiisuudhaaf maloota karoora maatii fayyadamtan ilaalchisee gaaffilee muraasaa sigaaffadha.** | | | | | | | | | | | | | | | |
| 19 | | | Ulfa tursiisuudhaaf ykn haambisuudhaaf mala karoora maatii fayyadamtee beektaa? | | Eeyyee 1  Miti 0 | | | | | | | | | | Miti yoo ta’r gara G25 |
| 20 | | | Yeroo jalqabaatiif mala ulfa tursiisuu/haambisuu yammuu fayyaadamtuu umriin kee waggaa meeqa turee?  **UMRII WAGGAAN GALMEESSI. KANAAN DURA YOO HINFAYYADAMNE ‘0’,YOO GUYYAA ITTI JALQABDE YAADACHUU HINDANDEENYEE ‘-88’ GALMEESSI.** | | Umrii | | |  | | | | | | |  |
| 20a | | | Yeroo jalqabaatiif mala koroora maatii fayyadamuu yammuu jalqabdu ijoollee meeqa qabdaa turee? | | Baay’ina ijoollee | | |  | | | | | | |  |
| 21 | | | Yeroo jalqabaatiif mala kam fayyadamtee?    **FILANNOO HIN DUBBISIN,FILLANNO HUNDA ILAALI.** | | Dubarti maseensuu 1  Dhiira maseensuu 2  Gadaameesa keesa kan taa’u (luuppii) 3  Lilmoo/Injectables 4  Kan irree jala awaalamu/Implaanti/ 5  Kininii (pilsii) 6  Kondomii dhiiraa 7  Kondomii dubaraa 8  Ittisa ulfaa yeroo haatatamaa(EC)…………..9  Mala guyyaa lakkaa'u /caleen 12  Daa’ima harma qoofaa hoosiisudhaan /LAM/ 13  Mala biro kan amayaa 14  Mala guyyaa lakkaa'u 15  Dhangala’a dhiiraa alati jiiksuun 16  Mala aadaa biroo 17 | | | | | | | | | |  |
|  | | | **GD 14 ILAALLI/MIRKANEEFADHU:** Yeroo amma ulfa? | | Eeyyee 1  Miti 0 | | | | | | | | | | 1 yoo ta’e gara G. 25 |
| 22 | | | Yeroo ammaa ati YKNabbaa manaa kee ulfa tursiisuudhaaf/ haambisuudhaaf malli itti fayyadamtan ni jiraa? | | Eeyyee 1  Miti 0 | | | | | | | | | | 0 yoo ta’e gara G. 25 |
| 23 | | | Yeroo ammaa tooftaa/mala kamiin fayyadamtaa?  Kan biroo?  **MALA EERAN HUNDARRATTI MARSI, FILLANNO HUNDAA ILAALI.** | | 1. Dubarti maseensuu  2. Dhiira maseensuu  3. Kan gadaamees keesa taa’u (luuppii)  4. Lilmoo/ Injectables  5. Kan irree jalati awaalamu /Implaanti  6. Kininaa (pilsii)  7. Kondomii dhiiraa  8. Kondomii dubaraa  9. Ittisa ulfaa yeroo haatatamaa(emergency)    10. Mala guyyaa lakkaa'u /caleen  11. Daa’ima harma qoofaa hoosisudhaan  12. Mala biro kan amayaa  13. Mala guyyaa lakkaa'u  14. Dhangala’a dhiiraa alati jiiksuun  15. Mala aadaa biroo | | | | | | | Y  1  1  1  1  1  1  1  1  1  1  1  1  1  1  1  1  1 | N  0  0  0  0  0  0  0  0  0  0  0  0  0  0  0  0  0 | | Skip based on most effective method only  Skip to 29 if main method is 3-17 |
| 24 | | | Malli kun yeroo dheeraadhaaf akka tajaajilu ogeessi fayyaan sitti YKN abbaa manaa keettiti hime ture? | | Eeyyee 1  Miti 0 | | | | | | | | | | gara G. 29 |
| 25 | | | Iddoo tajaajila karooraa maatii itti argachuu dandeessuu ni beektaa? | | Eeyyee 1  Miti 0 | | | | | | | | | |  |
|  | | | **GD 14 ILAALLI :** Yeroo ammaa ulfa? | | Eeyyee 1  Miti 0 | | | | | | | | | | 26a, mitiif eeyee yaata 25b |
| 26a | | | Yerooo ammaa mala karoora maatii fayyadamaa akka hinjire natti himte jirta,gara fuladuraatti, yeroo kamiyyuu keessatti ,mala ulfa tursiisu/hambisuu ni fayyadama jetee yaadaa? | | Eeyyee 1  Miti 0 | | | | | | | | | |  |
| 26b | | | Fuladuraatti, yeroo kamiyyuu keessatti ,mala ulfa tursiisun yookiin hambisuun ni fayyadama jetee yaadaa? | | Eeyyee 1  Miti 0 | | | | | | | | | |  |
|  | | | **GD 19 ILAALLI** : Mala ulfa tursiisuu YKN hambisuu ni fayyadamti? | | Eeyyee 1  Miti 0 | | | | | | | | | | Miti yoo ta’e 43 |
| 27 | | | Ji’oota 12 darban keessatti , mala ulfa tursiisuu YKN hambisuu fayyadamtee beektaa? | | Eeyyee 1  Miti 0 | | | | | | | | | | Miti yoo ta’e 43 |
| 28 | | | Yeroo ammaa tooftaa/mala karoora maattii kamitti fayyadamtaa?  Kan biroo?  **MALA FAAYYIDA OL’AANAA QABU FILLADHU**    **FILANNOO HUNDAA ILAALLI.** | | Dubarti maseensuu 1  Dhiira maseensuu 2  Gadaameesa keesa kan taa’u (luuppii) 3  Lilmoo/Injectables 4  Kan irree jala awaalamu/Implaanti/ 5  Kininii (pilsii) 6  Kondomii dhiiraa 7  Kondomii dubaraa 8  Ittisa ulfaa yeroo haatatamaa(EC)…………..9  Mala guyyaa lakkaa'u /caleen 12  Daa’ima harma qoofaa hoosiisudhaan /LAM/ 13  Mala biro kan amayaa 14  Mala guyyaa lakkaa'u 15  Dhangala’a dhiiraa alati jiiksuun 16  Mala aadaa biroo 17 | | | | | | | | | |  |
| 29 | | | Yoom irraa eegaltee (TOOFTAA AMMAA FAYYADAMAA JIRTU KANA) fayyadamuu jalqabdee?  **GUYYAA GALMEESSAA:**  **BARBAACHISAA YOO TA’E GUYYAAN SENOOTA DARBAN WALLIN WALQABSIISUDHAAN AKA YAADATAN GOODHUN NIDANDA’MA.** | | Ji’a | | | Waggaa | | | | | | |  |
|  | | | **GD 22 ilaalli/MIRKANEEFADHU:** Yeroo amma mala karoora maatii fayyaadama jirta? | | Eeyyee 1  Miti 0 | | | | | | | | | | Eeyeyoo ta’e gara G. 32 |
| 30 | | | Mala Karoora Maatii kana (YEROO DHIHOO FAYYAADAMA TURTEE) yoom adaan kutee/dhaabde?  **BARBAACHISAA YOO TA’E GUYYAAN SENOOTA DARBAN WALLIN WALQABSIISUDHAAN AKA YAADATAN GOODHUN NIDANDA’MA.** | | Ji’a | | | Waggaa | | | | | | |  |
| 31 | | | Mala Karoora Maatii knaa (YEROO DHIHOO FAYYAADAMA TURTEE) maaliif adaan kutee/dhaabdee? | | Yeroo baay’ee qunnamti saalaa waan hingooneef 1  Fayyadama osoo jiru waan ulfaa’ef 2  Ulfaa’u waan barbaaduuf 3  Abbaan manaa koo waan hineeyamneef 4  Mala irra wayaa’u waan barbaaddeef 5  Mala barbaaduu waan hinarganeef 6  Rakkoo fayyaa waan qabuuf 7  Cinaa walmaddii waanqabuuf 8  Waan hinargamnee/fagoo waan ta’eef 9  Gatii mi’a waan ta;e 10  Fayyamaduuf namatti waan hintolneef 11  Nadhukkubsa/naageessaa 12  Waan hinulfoofnee 13  Adeemsa ummamaa waan falleessuuf 14  Kan biroo 15  Hin beeku -88 | | | | | | | | | |  |
| 32 | | | Yoo jalqabaa (TOOFTAA DHIHOO/AMMAA ITTI FAYYADAMTUU ) essaa argatee? | | **Dhabbataa Fayyaa Ummataa**  Hoospitaala Mootummaa 1  Buufata Fayyaa Mootummaa 2  Keellaa Fayyaa Mootummaa 3  Kiliniikii karoora maatii 4  Kiliniikii moobaayilaa 5  Fieldworker/outreach/peer educator 6  **Dhabbataa. Fayyaa Dhunfaa**  Kiliniikii/ Hoospitaala Dhuunfaa 8  Faarmaasii 10  Kusaa mana Qorichaa 11  **Madoota biroo**  Suqii/gabaa 14  Tola oltootahaawasaa 16  Hirriyootaa 17  Miti-mootummaa/NGO 18  Kan biroo 19  Hin beeku -88 | | | | | | | | | |  |
| 33 | | | Ji’ootaan 12 darban keessatti tajaajila karoora maatii argachuudhaaf ?(mala yeroo dhihoo/ammaa fayyadama jirtuu dabalatee) kafalti kafaltee beektaa? | | Eeyyee 1  Miti 0 | | | | | | | | | | Miti yoo ta’e gara G.35 |
| 34 | | | Meeqa kafaltee?    **Kafaltii qarshii(briin galmeessaa).**  **YOO HIN BEEKNE -88 GALMEESSI.** | | Kafalti : | | | | | | | | | |  |
| 35 | | | Yammuu (MALA AMMA/DHIHOO FAYYADAMA JIRTUU) fayyadamuuf fudhatuu sanaatti waa'ee rakkoo malla kanaan walqabatee dhuufu danda'u ilaalchiisee ogeesoota fayyaatiin sitti himamee turee? | | Eeyyee 1  Miti 0 | | | | | | | | | | Miti yoo ta’e gara G.37 |
| 36 | | | Yeeroo malli karoora maatii kun rakkoo sitti fidee maal godhuu akka qabdu sitti himamee turee? | | Eeyyee 1  Miti 0 | | | | | | | | | |  |
| 37 | | | Yeroo sanaatti, (MALA AMMA/DHIHOO FAYYADAMA JIRTUUN ALATI ) filannoon biro akka jiru sitti himamee turee? | | Eeyyee 1  Miti 0 | | | | | | | | | |  |
| 38 | | | Yeroo sanaa mala filannoo kee kan ulfa tursiisuu/haambisuudhaaf fayyadamuu barbaaddu argatee turee? | | Eeyyee 1  Miti 0 | | | | | | | | | | Eeyyeen yoo ta’e gara G 40 |
| 39 | | | Kan barbaade yoo hin argane, maaliif laata? | | Mala fayyadamu guyyaa sana waan hinarganeef/dhumeef/ 1  Malli waan hin argamneef 2  Oggeessooni tajaajila keennuf leenji barbaachiisu hin fudhane 3  Oggeessi mala biroo akka fudhadhu waan nagorseef 4  Mallicha fayyadamuu waan hin dandeenyeef 5  Mala kana dhiisuf murteesee 6  Gatiin isaa mi’a waan ta’eef 7  Kan biroo 8 | | | | | | | | | |  |
| 40 | | | Yeroo sanaa, mala karoora maatii kam fayyadamuu akka qabdu murtee xumuraa kan murteessee eenyuu? | | Suma qofa 1  Ogeessaa fayyaa 2  Abbaa maanaa koo 3  Ana fi ogeessaa fayyaa 4  Ana fi abbaa manaa koo 5  Kan biroo 6 | | | | | | | | | |  |
|  | | | **GD.32 ILAALLI/MIRKANEEFADHU:** (MALA AMMAA FAYYADAMTUU) eessa irraa argatee? | | **Dhabbataa Fayyaa Ummataa**  Hoospitaala Mootummaa 1  Buufata Fayyaa Mootummaa 2  Keellaa Fayyaa Mootummaa 3  Kiliniikii karoora maatii 4  Kiliniikii moobaayilaa 5  Fieldworker/outreach/peer educator 6  **Dhabbataa. Fayyaa Dhunfaa**  Kiliniikii/ Hoospitaala Dhuunfaa 8  Faarmaasii 10  Kusaa mana Qorichaa 11  **Madoota biroo**  Suqii/gabaa 14  Tola oltootahaawasaa 16  Hirriyootaa 17  Miti-mootummaa/NGO 18  Kan biroo 19  Hin beeku -88 | | | | | | | | | | Gara g 44 yoo 32deebii 14-17 filatame |
| 41 | | | Dhaabbata/Ogeessaa fayyaaa mala karoora maatii kana siif keenne/dhiheessee biratii nideebitaa? | | Eeyyee 1  Miti 0 | | | | | | | | | |  |
| 42 | | | Hirriyaan tee dhaabbata/ ogeessaa fayyaa malakaroora maatii kana siif keenne/dhiheessee bira akka deemtu nieergitaa?aa? | | Eeyyee 1  Miti 0 | | | | | | | | | |  |
|  | | | **GD 16 ILAALLI/MIRKANEEFADHU :** gara fula duraatti daa'ima ni barbaadaa?  **GD 17 ILAALLI/ MIRKANEEFADHU**: daa'ima dabalata argachuun dura waggaa 2 YKN sanaa ol turuu barbaadii?    **GD 22 ILAALLI/ MIRKANEEFADHU :** Yeroo ammaa mala ulfa tursiisuu YKN hambisuu tii fayyadamaa jirtti ?  **GD 19 ILAALLI/ MIRKANEEFADHU :** Mala ulfa tursiisu YKN hambisuu tii fayyadamtee beekti ? | | Daa’inmma/daa’immaa biro qabdi 1  Hommaa/kanbiroo hinbarbaaddu 2  Ulfaa’u hinbar baaddu 3  Hin murteesine/Hin beeku -88 | | | | | | | | | | Ask 43 to non users (current or ever) who do not want a/another child or not before 2 years |
|  |  |  |  |  | Hommaa/daa’imma biro hinbarbaadduu 1  Waggaa lammaa gadii 2  Waggaa lamaa ol 3 | | | | | | | | | |  |
|  |  |  |  |  | Eeyyee,yeroo amma nifayyadamti 1  Miti,yeroo amma hinfayyadamtuu 0 | | | | | | | | | |  |
|  |  |  |  |  | Eeyyee 1  Miti 0 | | | | | | | | | |  |
| 43 | | | **Daa'imma/ Daa'ima biraa dahuu akka hin barbaadnee akasumas karooraa maatii ulfa ittisuu fayyadamaa akka hinjire natti himtee jirta.**    Ulfa tursisuudhaaf/hambisuudhaaf karoora maatii sababa maliif akka hinfayadamnee mee natti himi? | | Yeroo baay’ee qunnamti saalaa waan hingooneef 1  Fayyadama osoo jiru waan ulfaa’ef 2  Ulfaa’u waan barbaaduuf 3  Abbaan manaa koo waan hineeyamneef 4  Mala irra wayaa’u waan barbaaddeef 5  Mala barbaaduu waan hinarganeef 6  Rakkoo fayyaa waan qabuuf 7  Cinaa walmaddii waanqabuuf 8  Waan hinargamnee/fagoo waan ta’eef 9  Gatiin mi’a waan ta;e 10  Fayyamaduuf namatti waan hintolneef 11  Nadhukkubsa/naageessaa 12  Waan hinulfoofnee 13  Adeemsa ummamaa waan falleessuuf 14  Kan biroo 15  Hin beeku -88 | | | | | | | | | |  |
| 44 | | | Ji'ootan 12 darban keessati, waa’ee karooraa maatii ilaalchisee ogeesoota fayyaatiin daawwatamtee/ilaalamtee turee? | | Eeyyee 1  Miti 0 | | | | | | | | | |  |
| 45 | | | Ji'oota 12 dabran keessaa, kunuunsa fayyaa ofii kee YKN ijoollee keetiif jeecha gara dhaabbata fayyaa deemtee beektaa? | | Eeyyee 1  Miti 0 | | | | | | | | | | 0 yoo ta’e gara G 47 |
| 46 | | | Yeroo sanaa,miseenssota ogeessa fayyaa keessaa waa’ee karoora maatii kan sitti himan jiru? | | Eeyyee 1  Miti 0 | | | | | | | | | |  |
| 47 | | | Ji’ootan muraasa darban keessatti  Waa’ee karoora maatii raadiyoo irraa dhageessee?   Waa’ee karoora maatii TV irraa argitee?  Waa’ee karooraa maatii gazeexaa/barrulee irraa dubbistee? | |  | | | Eeyyee  1  1  1 | | | | Miti  0  0  0 | | |  |
| 48 | | | Mee ama waa’ee dhuunfaa keetiin sigaafadha.  Yeroo jalqaba qunamtii saalaa raawatu umriin kee waggaa meeqa turee?  **UMRII WAGGAAN BAREESSI. QUNNAMTI SAALAA YOO HIN RAAWWATNE ‘0’ BAREESSI.YOO HINBEEKNE ‘-88’ BAREESSI.** | | Umrii | | |  | | | | | | | ‘0’ yoo gutame gara G 50 |
| 49 | | | Yeroo xumuraatiif qunnamtii saalaa yoom akka gootee mee nati himi?  **Ji’a 12 (WAGGAA TOKKOO) OL YOO TA’E WAGGAADHAAN GALMEESSI.**  **JI’OOTA 12 GADII YOO TA’E,GUYYAADHAAN,TORBAANIIN YKN JI’AAN GALMEESSI**  **GUYYUMA SANA YOO TA’E ‘0’ GALMEESSI.** | | **GUYYOOTA DURA** | **TORBEE DURA** | | **JI’OOTA DURAA** | | | | **WAGOOTA DURA** | | |  |
| **Kutaa 4 Bishaan**  **Amma waa’ee bishaani ilaalchisee gaafilee muraasa sigaafachuu barbaadaa .** | | | | | | | | | | | | | | | |
| 50 | | Yeeroo **bonaa** bishaan tajaajila adda addaatiif oluu argachuudhaaf guyyaatti sa’aatti meeqa fayyadamta?    **GAAFFATAMTUUN BISHAAN WARAABUUF YEROON DABARSIITU GALMAA’U QABA. SA’AATIN NAMNI BIRA DABARSEE GALMAA’U HINQABU.** | | sa’aatti guyyaa keessaa dabarsituu | | | | | | | |  | | |  |
|  |  |  |  | Nama biraatu waraaba 33  Bishaan hin waraabnu 44  Hin beeku -88 | | | | | | | | | | |  |
| 51 | | Yeeroo **ganaa** bishaan tajaajila adda addaatiif oluu argachuudhaaf guyyaatti sa’aatti meeqa fayyadamta?  **GAAFFATAMTUUN BISHAAN WARAABUUF YEROON DABARSIITU GALMAA’U QABA. SA’AATIN NAMNI BIRA DABARSEE GALMAA’U HINQABU.** | | sa’aatti guyyaa keessaa dabarsituu | | | | | | | |  | | |  |
|  |  |  |  | Nama biraatu waraaba 33  Bishaan hin waraabnu 44  Hin beeku -88 | | | | | | | | | | |  |
| **Galatoomii**  **GAAFFI FI DEEBIIN XUMURAMEERRA. GARUU GAAFFIIN LAMAA KAN MANAAN ALATTI GUUTAMU ISIN HAAFA.** | | | | | | | | | | | | | | | |
| **IDDOO QORANNOON ITTI ADEEMSIFAMEE** | | | | | | | | | | | | | | | |
| M | | Naannoo seensa mannichatti koordineetii GPSii fudhadhu    Safartuun GPSii sirrii kan ta’u meetraa 6 gadi yoo ta’e dhaa. | | | *ODKn kallatiidhaan qajeelfama ni keenna*  TEESOO GAMEESSI | | | | | | | | | |  |
| BU’AA GAAFFIIFI DEEBII | | | | | | | | | | | | | | | |
| N | | Bu’aan gaaffiifi deebii | | | Xumuramee 1  Miseensii maatii kamiyyuu mana keessa hinjiru 2  Guyyaa biraatiit dabarfamee 3  Didan 4  Gartokeen xumuramee 5  Deebii keenuu hin danda’anu 6 | | | | | | | | | |  |
