# Supplementary material for: Does proximity of women to facilities with better choice of contraceptives affect their contraceptive utilization in rural Ethiopia?
Source: PLoS One. 2017 Nov 13;12(11):e0187311. doi: 10.1371/journal.pone.0187311 (PMC5683563; doi:10.1371/journal.pone.0187311)
Supplement: S1 File — (ZIP) [file pone.0187311.s004.zip › Questionnaires/Afan Oromo version/PMA2020-HR_Afan Oromo-13_11_2013.docx]

| **mADDS–Gaffannoo Maatii** |
| --- |

| **LAK** | **GAAFIIFI CALALEESITUU** | **KOODII** | | | | | | **DARBI** |
| --- | --- | --- | --- | --- | --- | --- | --- | --- |
| **EENYUMEESSITUU**  **Gaafiifi deebii jalqabuu keessan dura eenyumeesituu(identification) armaan gadii guutaa** | | | | | | | | |
| A | Maatii kana gaaffannoof yeroo meeqa daawwaattan? | Yeroo 1^ffaa^ 1  Yeroo 2^ffaa^ 2  Yeroo 3^ffaa^ 3 | | | | | |  |
| B | MAQAA GAAFATAA: maqaan kun kan keetii?  Yoo miti ta’e maqaakee barreessi  *ODKn maqaa lakkoofsa bilbilaa wajjin walqabatee ni agarsiisa.* | Eeyyee 1  Miti 0 | | | | | |  |
|  |  |  | | | | | |  |
| C | **GUYYAA FI SA’AATI AMMAA ISKRIINII IRRATTI NI MUL’ATA**  Guyyaa fi sa’aatiin mul’atee sirridha? | Eeyyee 1  Lakkii 0 | | | | | | Eeyyee yoo ta’e gara E darbi |
| D | Guyyaa fi sa’aa isa sirrii barreessi | Guyyaa | Guyyaa | | Ji’a | | Waggaa |  |
|  |  | Sa’aati | Sa’aati | Daqiiqaa | | AM/PM | |  |
| E | Naannoo    **MAQAA NAANNOO MAATIIN ITTI ARGAMUU FILADHA** | Tigraayi 1  Afaar 2  Amaaraa 3  Oromiyaa 4  Soomalee 5  Benishaanguul Gumuuz 6  SNNP 7  Gaambeelaa 8  Haarari 9  Finfinnee 10  Dirree Dawaa 11 | | | | | |  |
| E1 | Godina  **MAQAA GODINA MAATIIN ITTI ARGAMUU FILADHA** | *ODKn maqaa godinoota naannoo (GMG E) filatameef ni mul’isa* | | | | | |  |
| F | Aanaa  **MAQAA AANAA MAATIIN ITTI ARGAMUU GALMEESSAA.** | *ODKn maqaa aanoolee godina (GM G E1) filatameef ni mul’isa* | | | | | |  |
| G | Ganda  **MAQAA GANDA MAATIIN ITTI ARGAMUU GALMEESSAA.** | *ODKn maqaa gandoota aanaa (GM G F ) filatameef ni mul’isa* | | | | | |  |
| H | Lakkoofsa gandaa/araddaa qorannoo (enumeration area)  **LAKKOOFISA GANDA/ARADDAA QORANNOO MAATIIN ITTI ARGAMUU GALMEESSAA.** |  | | | | | |  |
| I | Lakkoofsa unkaa  **LAKKOOFSA UNKAA MAATIIN KUN ITTI ARGAMUU GALMEE MAATIIRRA GALMEESSAA** |  | | | | | |  |
| J | Lakkoofsa maatii  **LAKKOOFSA MAATII MAATIIN KUN ITTI ARGAMUU GALMEE MAATII IRRA GALMEESSAA** |  | | | | | |  |
| K | Guyyaa har’aatti namni miseensa maatii kanaa ta’ee, gaaffiidhaaf qophaa’e ni jiraa? | Eeyyee 1  Lakkii 0 | | | | | | Lakkii yoo ta’e gara P |
| **HIMA WALIGALTEE**  **Maatii keessa nama deebii gehaafi sirrii ta’e keennuu danda’u filadhu.Waligaltee armaan gadiifi seensa kana dubbiisaa.** | | | | | | | | |
| Akkam oltan/bultan.Maqaan koo________________________________jedhama .Ministeeraa Fayyaa fi yunivarsiitii Finfinneen dhimoolee fayyaa addaa addaarraatti qoranoo adeemsiisa jiru anis isaan wajjin hojjachaa jira.Isiniis qorannoo kanarratti hirmaachuu keessaan isin galateeffanna.Odeeffaannoo qorannoo kanarra argamuu mootummaan tajaajila fayyaa keennuu akka sirriitti karoorfatu gargaara.Odeeffannoo/deebii kennitan hundinu miseensoota qorannooo kana ala namni kamuu akka hinargine iccitiin qabamaa.  Qorannoo kana irratti feedhii gutuumma gutuu keessaniin kan hirmatan yammuu ta’u gaaffii deebiisuu hinbarbaadine yoo isin mudatee akka deebistan waan hindirqamneef natti himaatoo gara gaaffii itti aanuutti nidarbina ykn immoo gaaffif deebii yeroo kamuu addaan kutuu nidandeenyaa garuu hirmaanaan keessan baay’ee barbaachiisaa waan ta’eef gaaffiif deebii godhuuf akka nuuf eeyyamtan niabdana.  Gaaffiilee muraasaa waa’ee maatii keessanisin gaaffadha itti aansuun ,dubartoota umriin isaani waggaa 15-49 keessa jiraniif gaaffii adda adda ni gaaffanaa  Ammaa qorannoo kana ilaalchisee gaaffii nagaaffatan qabdu? | | | | | | | | |
| L | Koopii hima waligaltee itti kennuun, ibsa godhiif. Itti aansuun gaaffii fi deebii eegaluu danda’aa jeechuun gaaffii dhiheessaa? | Eeyyee 1  Lakkii- 0 | | | | | | Lakkii yoo ta’e gara P |
| M | Mallattoo deebii kennaa  **DEEBII KEENNITOONI QORANNOO KEESSATTI HIRMAACHUUF WALIGALUU ISAANI MIRKANEEFFACHUUF AKKA MALLATTEESSAN YKN SANDUQAA QOPHAA’EE KEESSATTI MALLATTO AKKA KA’AN GAAFFADHAA.** | MALLATTOO:  Saanduqa Mall.: ☐ | | | | | |  |
| N | Maqaa gaafatuu  **GAAFATAMAA WALIGAlUU ISA/ISHE RAGAA BAHUUF MAQAA KEE BARREESSA** |  | | | | | |  |
| O | Maqaa deebii kennaa  **MAQAA GAAFATAMAA BARREESSA** |  | | | | | |  |

| **KUTAA 1 Galmee Maatii**  **Ammaa waa’ee miseensoota maatii keessan gaaffilee muraasa isin gaaffadha.Mee isin irra eegalun miseensoota maatii yeeroo bay’ee isin wajjin jiraatani fi kaleessaa galgalaa kan isin wajjin bulan nagalmeessisaa.** | | | | | | | | | |
| --- | --- | --- | --- | --- | --- | --- | --- | --- | --- |
| Lak | 1 | 2 | 3 | 4 | | 5 | 6 | 7 | 8 |
|  | Maqaa | saala | Umrii | Haala gaa'elaa | | Firummaa/hariiroo (MAQAA)n dursa maatii waliin qabu maalii? | koodiimaatii | Namni kun miseensa maatii yeroo hundaati ykn kaleessaaa galgalaa mana keessaan bulee/tte? | Dubartii gaaffifi deebiif filatamtee |
|  |  | (MAQAA)n  dhiira moo  dhalaa?  Dhiira 1  Dubara 2 | (MAQAA)n umriin isaa/hee waggaa meeqa? | Yeroo ammaa haalli gaa'ela (MAQAA)n maali?  Kan fuudhe/heerumtee 1 Nama wajjin kan jiru/tu 2 walhikan/addaan bahan 3 kan irra du’e/dutee 4  Kan hin fuune/heerumnee 5 | | Duree Maatii 1  Abbaa manaa/Haadha manaa 2  Ilma ykn Intala 3  daa’ima ilmaa/intalaa 4  Akkaakayyuu 5  Warra (abbaa ykn haadha) 6  soddaa (haadha ykn abbaa haadhaa/abbaa manaa) 7  Obboleessa yookan Obboleetti 8  Kan biroo 9  Hin beeku -88 |  | miseensa maatii yeroo hundaa 1  miseensa maatii yeroo hundaa ta’ee kaleessaaa galgalaa mana keessaa kan hin bulee 2  keessummaa kaleessaaa galgalaa mana keessaa bulee 3 | Eeyyee…. 1  Lakkiii 0  ODkn namoota qorannoo kanaaf filataman ni baafata |
| 1 |  |  |  |  | |  |  |  |  |
| 2 |  |  |  |  | |  |  |  |  |
| *Odeeffannoo maatii tokkoo erga galmeesitan booda namoota hingalmeefamne jiraachuu isaani beekuuf gaaffii armaan gadii gaaffachuun ,namni hingalmaanee yoo jiratan, irra deebi’uun galmeessaa* | | | | | | | | | |
| 9 | Namoota biro yeroo baay’ee maatii kana wajjin jiraatan ykn keessummootafi yeroo muraasaaf isin bira turuuf dhufanifi kaleessaa galgalaa assitti bulani kan hin galmaanee jiru? | | | | Eeyyee 1  lakkii 0 | | | | Lakkii yoo ta’e gara G10 |

| **Kutaa 2: ODEEFANNOO QABIYYEE MAATII**  **Amma odeeffannoo qabiyyee maatii keessaan ilaalchisee gaaffilee muraasa isiin gaaffadha.** | | | | | | | | |
| --- | --- | --- | --- | --- | --- | --- | --- | --- |
| 10 | Maatiin keessan meeshaalee kanneen ni qaba?  Ibsaa Eleektrikaa?  Sa’aatii fannifamu?  Raadiyoo?  Televizyinii adii/gurraacha?  Televizyinii bifa hundaa?  Bilbila Moobaayilaa?  Bilbilaa manaa?  Qabbaneessituu/Firija?  Cabbeesituu?.................................................  Jenereteeraa Eleektriikaa?  Maashinii huccuu miicu?  Komputeraa?  Kaameeraa suuraa dijitaalii?  Kaameeraa suuraa dijitaalii hin taane?  Deekii Vidiyoo?  DVD ykn /CD tabachiiftu?  Makinaa hoccuu hodhuu?  Siree?  Minjaala?  Sanduuqa?  Biskleetii?  Doqdoqee/ Motor saaykilii?  Makinaa/konkoolaata ?  Hoboloo/bidiruu motoraa?  Hoboloo/bidiruu motora hinqabne?  Homaayyuu hin qabu  **MEESHAALEE HUNDA DUBBISUUN KAN QABAN HUNDA IRRATTI MARSI** |  | Eeyyee  1  1  1  1  1  1  1  1  1  1  1  1  1  1  1  1  1  1  1  1  1  1  1  1  1  -88 | | lakkii  0  0  0  0  0  0  0  0  0  0  0  0  0  0  0  0  0  0  0  0  0  0  0  0  0 | | |  |
| 11 | Horii/Loon qabduu? | Eeyyee 1  Lakkii 0  Beeyladoota hinqabanu | | | | | | Lakkii yoo ta’e gara G13 |
| 12 | Horii meeqa qabduu?  Horii/Beeyladoota hundaaf gaafadha?  Re'ee/Hoolaa  Hindaaqoo/lukkuu  Horii/ Farda/Harree/gaangee  **Duwwaa (0)n DEEBII TA’U NI DANDA’A YOO DEEBII HIN BEEKNE ’-88’ GALMEESSA. DEEBII YOO HINQABNE ‘-99’ BARREESSAA.** |  | | | | | |  |
| **Kutaa 3 Manaa jireenyaa qalbifachuu,**  **Lafa,baaxii fi dhaabaa mana jireenya daawwaachuun/qorachuun galmeessaa.** | | | | | | | | |
| 13 | Lafti mana keessan **irra caalaan**  maal irra hojjatame?  **DAAWWADHUU/QALBEEFADHU GALMEESSI** | LAFA UUMAMAA  Biyyoo/Cirracha 1  Dhoqee looniirra 2  LAFA HIN RAAWWATAMNEE/SALPHAA TA’EE  Mukaa  Shamabaqoo/Leemman 4  LAFA SIRRITTI XUMURAME  Paarkee/xaawulaa soofame 5  Vinaayilli/Aspaaltii 6  Taayilsii seeraamiksii /teeraazoo 7  Simintoo 8  Afa lafaa suufii 9  Afa plaastiikaa 10  Kan biroo 11 | | | | | |  |
| 14 | Uffisa gubbaa(baaxii) mana jireenyaa  keessan **caalan** maal irra  hojjatamee?  **DAAWWADHUU/QALBEEFADHU GALMEESSI** | BAAXII MEESHAALEE UUMAMAA  Baaxii ummamaa  Baaxii hinqabu 1  Citaa 2  BAAXII HINRAAWWATAMNEE  Saleenii /Rustic Mat/ 3  Leemman/shambaqoo 4  Muka /Xaawulaa hinsofamne 5  Kaartoonaa 6  BAAXII XUMURAMAA  Qorqoorroo 7  Mukaa 8  Fayberii simintoo/Cement Fiber 9  Afaa baaxii seramikaa 10  Simintoo 11  Baaxii shinglii /Roof Shingles/ 12  Asbeestosii 13  Kan biroo 14 | | | | | |  |
| 15 | Dhaabni (Girgiddaan) manaa  **Irra caalan** maal irraa  hojjatamee?  **DAAWWADHUU/QALBEEFADHU GALMEESSI** | DHAABAA UUMMAA  Dhaabaa hinqabu 1  Mukaa 2  DHAABAA HIN RAAWWATAMNEE  Shamabaqoo fi dhoqeen 3  Dhakaa fi Dhoqqee 4  Girgiddaa hinuwifamnee 5  Xaawulaa 6  Kaartoonaa 7  Muka gosa adda addaa 8  DHAABAA XUMURAMEE /FINISHED WALLS/ Finished Walls  Simmintoo 9  Dhakaa simmintoon ijaaramee 10  Xubbii/Bricks 11  Bilookeetti 12  Xuubii uwifamee 13  Mukaa hinmidhaginerra 14  Kanbiro 15 | | | | | |  |
| **Kutaa 4 Mada bishaanifi haala qulqullina**  **Amma waa’ee madootaa bishaani fi qulqullina naannoo ilaalchisee gaaffilee muraasaa isin gaafadha.** | | | | | | | | |
| 16 | Bakka itti harka dhiqatan qabduu? | Eeyyee 1  Lakkii 0  Hin beeku -88 | | | | | | Lakkii yoo ta’e gara19 |
| 17 | Natti agarsiisuu dandeessuu? | Eeyyee 1  Lakkii 0 | | | | | | lakkiiyoo ta’e gara19 |
| 18 | **MAATIIN BAKKA HARKA ITTI DHIQATAN WANTOOTA ARMAAN GADII JIRAACHUU ISAANI ILAALAA**  Saamuunaan nijira  Bishaan(meshaan kuufame) nijira  Bishaan boombaa nijira  Mana finchaani bira iddoo harka itti dhiqatan nijira  Wantoon armaan oli hundinu hinjiranu |  | | Eeyyee  1  1  1  1  1 | | | lakkii  0  0  0  0  0 |  |
| 19 | Maatiin keessan yeroo baay’ee jechuun waggaa keessaa yeroo kamuu bishaan tajaajila adda addaaf (nyaata qopheefachuuf,  huccuu michuuf..) eessa argatan?  BISHAAN UJUMMOO/BOOMBAA boobaa mana jireenyaa seenee  boobaa oddoo/dalaatti seenee  Boombaa ummata/boonoo  Bishaan boola gad fagaatee  BISHAAN BOOLA Al  Bool’a qadaadame/eegamee  Bool’a hinqadaadamne/hineegamne  BISHAAN BURQAABurqaa qadaadamee/itti ijaar  Burqaa Hinqadaadamne/hinijaaramne  Bishaan roobaa  Bishaan taankarii/bootee  Bishaa taankariin gaariin haarkifamu  Bishaan lafarraa/badhee laga/hidhaa/haroo  Bishaan plastiikaa/ Bottled Water  Bishaan laastikii /Sachet water/  **HUNDA ISAANI TOKKO TOKKOON DUBBISUUN GAAFFADHA.** |  | | Eeyyee  1  1  1  1  1  1  1  1  1  1  1  1  1  1  1 | | | lakkii  0  0  0  0  0  0  0  0  0  0  0  0  0  0  0 |  |
| 20 | Maatii keessaniif bishaan dhugaattif oluu irra caalaa eessarraa argata?  BISHAAN UJUMMOO/BOOMBAA Boombaa mana jireenyaa seenee  Boombaa oddoo/dalaatti seenee  Boombaa ummata/boonoo  Bishaan boola gad fagaatee  BISHAAN BOOLA Al  Bool’a qadaadame/eegamee  Bool’a hinqadaadamne/hineegamne  BISHAAN BURQAA  Burqaa qadaadamee/itti ijaar  Burqaa Hinqadaadamne/hinijaaramne  Bishaan roobaa  Bishaan taankarii/bootee  Bishaa taankariin gaariin haarkifamu  Bishaan lafarraa/badhee laga/hidhaa/haroo  Bishaan pilaastikaa/ Bottled Water/  Bishaan laastikkii /Sachet Water/  **HUNDA ISAANI TOKKO TOKKOON DUBBISUUN MADDA YEROO BAAY’EE FAYYADAMAN GUUTAA.**  **GAAFFII HQ 19 TI FILATAMUU QABA.** | 1  2  3  4  5  6  7  8  9  10  11  12  13  14  15 | | | | | |  |
| 21 | Maatiin kun bishaan tajaajila adda addaaf ooluu fakkeenyaaf nyaata bilcheessuuf, harka dhiqachuuf fayyadamuuf irra caalaa eessa argataa?  BISHAAN UJUMMOO/BOOMBAA Boombaa mana jireenyaa seenee boobaa oddoo/dalaatti seenee  Boombaa ummata/boonoo  Bishaan boola gad fagaatee  BISHAAN BOOLA Al  Bool’a qadaadame/eegamee  Bool’a hinqadaadamne/hineegamne  BISHAAN BURQAA  Burqaa qadaadamee/itti ijaar  Burqaa Hinqadaadamne/hinijaaramne  Bishaan roobaa  Bishaan taankarii/bootee  Bishaa taankariin gaariin haarkifamu  Bishaan lafarraa/badhee laga/hidhaa/haroo  Bishaan pilaastikaa/ Bottled Water  Bishaan laastika xixiqoo /Sachet Water/  **HUNDA ISAANI TOKKO TOKKOON DUBBISUUN MADDA YEROO BAAY’EE FAYYADAMAN GUUTAA.**  **GAAFFII HQ 19 TI FILATAMUU QABA.** | 1  2  3  4  5  6  7  8  9  10  11  12  13  14  15 | | | | | |  |
| 22 | **GAAFFII HQ 22 - HQ 24 TIIF MADOOTA BISHAANI GAAFFII 17 IRRATTI IBSAMAN SI’A MEEQA IRRA DEDEEBI’UN AKKA FAYYADAMAN GAAFFADHA:**  *ODKn madoota bishaani Gaaffii HQ 19.*  *Irratti ibsaman ni mul’isa*  (MADDA BISHANI IBSA) akka fayyadamtan natti himtanirtu.Waggaa keessatti yeroo kamiyyuu keessatti maatii keessan bishaan kana ---- tiif fayyadama  Dhugaatiif  Nyaata bilcheesuuf  Beeyladootaaf/Livestock  Qonnaa/biqiltuuf  Daldalaaf  *ODKn madoota bishaani Gaaffii HQ 19.*  *Irratti ibsaman hundaaf gaafii walfakaatuu ni mul’isa.* |  | | Eeyye  1  1  1  1  1 | | lakkii  0  0  0  0  0 | |  |
| 23 | (MADDI BISHAAN ITTI) yeroo baay’ee bishaan argamaa:  waggaa guutuu nijira  Waggaaa keessatti yeroo muraasaa  Waggaaa keessatti yeroo xiqoof 3  *ODKn madoota bishaani Gaaffii HQ 19.*  *Irratti ibsaman hundaaf gaafii walfakaatuu ni mul’isa* | 1  2  3 | | | | | |  |
| 24 | (MADDA BISHAANI) bishaan yeroo barbaaddan hunda ni argatani?  Eeyyeen, yeroo baay’ee niargama  Miti,darbee darbee ni ciccita/akka dhaabbatuu dhabamu ni beekamaa  Miti,yeroo kam akka ciccituu/dhaabbatuu hin beekamuu  *ODKn madoota bishaani Gaaffii HQ 19.*  *Irratti ibsaman hundaaf gaafii walfakaatuu ni mul’isa.* | 1  2  3 | | | | | |  |
| 25 | Bakkaa bishaan kun itti argamu deemuuf  daqiqaa meeqa fudhataa?  **“0” DEEBII TA’U NI DANDA’A**  **YEROON KUN SAA’AATTI DABAREE/WARAFAA ITTI EEGATAN NI DABALATA.**  **YOO HINBEEKNE “88”**  *ODKn madoota bishaani Gaaffii HQ 19.*  *Irratti ibsaman hundaaf gaafii walfakaatuu ni mul’isa* | Daqiiqaa | |  | | | |  |
| 26 | Maatiin keessan lafaa biqiltuu niqabaa? | Eeyyee 1  lakkii 0 | | | | | |  |
| 27 | Miseensooni maatii keessan tajaajiloota mana fincaanii armaan gadii tarreeffamaniin ni fayyadama?  MANA FINCAANII KAN BISHAANIIN HARAMEE DEEMU KAN  Tuboo balfa baasuu  Boola bobaa kuusuu(Septic tank)  Iddoo birootti kan yaa’u  hinbeekamu  Mana finchaanii boolaa qileensa baasuu qabu  Mana finchaanii boolaa lishoon hojjatamee  Mana finchaanii boolaa lishoon hinhojjatamnee  Boola bobaan xaa’oof akka oluuf hojjatamee (Composting toilet)  Poopoo  Gara lagaatti kan yaa’u  Kan biroo :  **DEEBII HUNDA TOKKO TOKKOON DUBBISUUN GAAFFADHA** |  | | Eeyyee  1  1  1  1  1  1  1  1  1  1  1 | | | lakkii  0  0  0  0  0  0  0  0  0  0  0 |  |
| 28 | **Yeroo baay’ee maatiin keessan goosa mana fincaanii kamitti fayyadama?**  MANA FINCAANII KAN BISHAANIIN HARAMEE DEEMU KAN  Tuboo balfa baasuu  Boola bobaa kuusuu(Septic tank)  Iddoo birootti kan yaa’u  hinbeekamu  Mana finchaanii boolaa qileensa baasuu qabu  Mana finchaanii boolaa lishoon hojjatamee  Mana finchaanii boolaa lishoon hinhojjatamnee  Boola bobaan xaa’oof akka oluuf hojjatamee  Poopoo  Gara lagaatti kan yaa’u  Kan biroo :  **DEEBII HUNDA TOKKO TOKKOON DUBBISUUN GAAFFACHUUN KAN YEROO BAAY’EE FAYYADAMAN FILADHA.GAAFFII 27TI FILATAMU QABA** | 1  2  3  4  5  6  7  8  9  10 | | | | | |  |
| 29 | **GAAFFIIN 25 MANA FIINCAANII FILATAMAN HUNDAAF.GAAFFII 29 YEROO –IRRA DEEBI’UUN GAAFFACHUU QABDAN**  *ODKn Mana fiinchaanii HQ 27 filataman hundaaf gaaffii walfakaatu ni mul’isa*  Maatiin keessan(GOSA MANA FINCAANII IBSAN) yeroo hangam fayyadamaa?  **MAATIIN YEROO BAAY’EE KAN FAYYADAMUU QOFA GUUTAA.** | Yeroo hundaa 1  Yeroo baay’ee 2  Dabree dabree 3  Yeroo tokko tokkoo 4 | | | | | |  |
| 30 | Maatii keessan keessaa nama meeqatu yeroo hundaa bakkeetti/bosoonaatti fayyadama?    **MAATII KANA KEESSATTI NAMA/OOTA--- NI JIRU?**  **YOO HINBEEKNE TA’E -88 GALCHA.** | Baay’ina namoota | |  | | | |  |
| 31 | *Daa’imman waggaa shanii gadi ta’aniif, bobbaa isaanii dhabamsiisuuf mala akkamiitti fayyadamtu/* qulquleessitan?  Mana finchaanii fayyadamnaa  Achumatti dhifama  Urufa/dirree irratti dhiifamaa  Mana fincaaniitti keessatti gatamaa  Balfa/koosii irratti gatamaa  Bishaan faalameerratti gatame  Koosii/xaa’o goone  Ni gubamaa  Hin beeku  *ODKn galmee (HQ3) maatiirra daa’imman umrii isaani waggaa 5 gadii ta’aniif qofa gaafata.* |  | | Eeyyee  1  1  1  1  1  1  1  1  -88 | | | Lakkii  0  0  0  0  0  0  0  0 |  |
| Gaafatama galateefadhu  **GAAFFI FI DEEBIIN XUMURAMEERRA GARUU GAAFFII LAMAA MANA ALARRATTI KAN GUUTAMU ISIN HAAFA.** | | | | | | | | |
| **IDDOO QORAANNOO ITTI ADEEMSIFAMEE FI BU’A QORANNICHA** | | | | | | | | |
| P | Naannoo seensa mannichatti koordineetii GPSii fudhadha    Safartuun GPSii sirrii kan ta’u meetraa 6 gadi yoo ta’e    **DUBBIFAMNI GPSIIN KAN FUDHATAMU MANAA ALAA.** | *ODKn kallatiidhaan qajeelfama ni keenna*  TEESOO/IDDO/ SANA GALMEESI | | | | | |  |
| Qa | Suuraa seensa manichaa kaaasuuf eeyyama gaaffadha.  Suuraa kaasuuf eeyyama argatani? | Eeyyee 1  lakkii 0 | | | | | | Lakkii yoo ta’e gara R |
| Qb | Namni kamuu suuraa keessatti akka hinjiraane mirkaneefadha. | *ODKn qajeelfama isiniif keennaa*  SUURAA KAASAA  FAKKII FILADHAA | | | | | |  |
| R | Bu’aan gaaffiifi deebii | Xumuramee 1  Miseensii maatii kamiyyuu mana keessa hinju ykn namni deebii keennuu hinjiru 2  guyyaa biraaf dabarfamee 3  Gaafiifi deebii nididaan 4  Gariin xumuramee 5  Manni duwaadha/Mana jireenya miti 6  Mannichi digameerra 7  Manichi argamuu hindandeenyee 8 | | | | | |  |
