# Supplementary material for: Does proximity of women to facilities with better choice of contraceptives affect their contraceptive utilization in rural Ethiopia?
Source: PLoS One. 2017 Nov 13;12(11):e0187311. doi: 10.1371/journal.pone.0187311 (PMC5683563; doi:10.1371/journal.pone.0187311)
Supplement: S1 File — (ZIP) [file pone.0187311.s004.zip › Questionnaires/Afan Oromo version/PMA2020-SQ_Afan Oromo 13_11_2013.docx]

| **mADDS –Gaaffannoo Dhaabbata Fayyaa** |
| --- |

| **LAK** | **GAAFIIFI CALALEESITUU** | | **KOODII** | | | | | | | | | | | | | | | | | | | | | | | | | | | | | | | | | | | **DARBI** |
| --- | --- | --- | --- | --- | --- | --- | --- | --- | --- | --- | --- | --- | --- | --- | --- | --- | --- | --- | --- | --- | --- | --- | --- | --- | --- | --- | --- | --- | --- | --- | --- | --- | --- | --- | --- | --- | --- | --- |
| **EENYUMEESSITUU**  **Gaafiifi deebii jalqabuu keessan dura eenyumeesituu(identification) armaan gadii guutaa** | | | | | | | | | | | | | | | | | | | | | | | | | | | | | | | | | | | | | | |
| A | Maatii kana gaaffannoof yeroo meeqa daawwaattan? | | Yeroo 1^ffaa^ 1  Yeroo 2^ffaa^ 2  Yeroo 3^ffaa^ 3 | | | | | | | | | | | | | | | | | | | | | | | | | | | | | | | | | | |  |
| B | Maqaa gaafatichaa: maqaan kun kan keetii?  *ODKn maqaa lakkoofisa bilbilaa wajjin walsimu ni mul’isa .*  Yoo kan kee miti ta’e maqaakee barreessi | | Eeyyee 1  Miti 0 | | | | | | | | | | | | | | | | | | | | | | | | | | | | | | | | | | |  |
|  |  |  |  | | | | | | | | | | | | | | | | | | | | | | | | | | | | | | | | | | |  |
| C | **Guyyaa fi sa’aatiin skrinii irrati nimulata.**  Guyyaa fi sa’aan kun sirriidhaa? | | Eeyyee 1  Miti 0 | | | | | | | | | | | | | | | | | | | | | | | | | | | | | | | | | | | Eeyee yoo ta’e gara GD |
| D | Guyyaa fi sa’aa sirrii ta’e barreessi | | Guyyaa | | Guyyaa | | | | | Ji’a | | | | | | | | | | | | | | | Waggaa | | | | | | | | | | | | |  |
|  |  |  | Yeroo | | Sa’aati | | | | | Daqiiqaa | | | | | | | | | | | | | | | AM/PM | | | | | | | | | | | | |  |
| E | **Naannoo**  **NAANNOO DHAABBATICHI ITTI ARGAMU FILADHU** | | Tigraayi 1  Afaar 2  Amaaraa 3  Oromiyaa 4  Soomalee 5  Beni.Gumuuz 6  SNNP 7  Gaambeelaat 8  Haararri 9  Finfinnee 10  Dirree Dawaa 11 | | | | | | | | | | | | | | | | | | | | | | | | | | | | | | | | | | |  |
| F | GODINA  **GODINA DHAABBATICHI ITTI ARGAMU BARREESSI** | | *NAANNOO gaafii E tiif filatame irrati hundaudhaan ODAn maqaa Godinaa SQ E tiif filatamee ni mul’isa* | | | | | | | | | | | | | | | | | | | | | | | | | | | | | | | | | | |  |
| G | **AANAA**  **AANAA DHAABBATICHI ITTI ARGAMU BARREESSI** | | *GODINA gaafii F tiif filatame irrati hundaudhaan ODAn maqaa Aanaa SQ F tiif filatamee ni mul’isa* | | | | | | | | | | | | | | | | | | | | | | | | | | | | | | | | | | |  |
| H | Maqaa Gandaa/Araddaa  **MAQAA GANDAA/ARADDAA DHAABBATICHI IITI ARGAMU FILADHU** | | *AANAA gaafii G tiif filatame irrati hundaudhaan ODAn maqaa GANDAA/ARADDAA SQ F tiif filatamee ni mul’isa* | | | | | | | | | | | | | | | | | | | | | | | | | | | | | | | | | | |  |
| I | Koddii iddoo qorannoo  **LAKKOOFISA/KODDII IDDOO QORANNOO MAATIIN ITTI ARGAMUU GALMEESSI.** | |  | | | | | | | | | | | | | | | | | | | | | | | | | | | | | | | | | | |  |
| J | **LAKKOFISAA DHAABBATAA**  **GALMEERRA LAKKOOFISA DHAABBATICHAA GALMEESSAA** | |  | | | | | | | | | | | | | | | | | | | | | | | | | | | | | | | | | | |  |
| K | Gosa dhaabbaticha  **GOSA DHAABBATICHA FILADHU** | | Hospitaalaa 1  Bufataa Fayyaa 2  Kellaa Fayyaa 3  Kilinika 4  Faarmaasii 5  Mana kusaa Qorichaa 6  Mana Qoricha Baadiyaa 7  Kan biroo 8 | | | | | | | | | | | | | | | | | | | | | | | | | | | | | | | | | | |  |
| L | **Hogganaa dhaabbataa**  **ITTI GAAFATAMAA/ HOGGANAA DHAABBATICHA FILADHA** | | Dhaabbata Mootummaa 1  Dhaabbata Miti Mootummaa 2  Dhaabbata amantaa 3  Kan dhuunfaa 4  Kan biro 5 | | | | | | | | | | | | | | | | | | | | | | | | | | | | | | | | | | |  |
| M | Guyyaa har’aatti miseensa dhaabbta kanaa keessaa nama gaaffannoof kan qophaa’e ni jiraa?  Is a competent respondent present and available to be interviewed today? | | Eeyyee 1  Miti 0 | | | | | | | | | | | | | | | | | | | | | | | | | | | | | | | | | | | Miti gara G R |
| **HIMA WALIGALTEE**  **Gaaffannoo kanaaf dhaabbta kana keessatti nama odeeffannoo gaha kennuu danda’u(Hoggannnaa dhaabbaticha ykn ittigaaffatamaa adeemsaa hojii karoora maatii ta’e nama hojachaa jiru dubbisuuf yaalii godha.Itti aansuun,hima waligaltee armaan gadii dubbsaaf** | | | | | | | | | | | | | | | | | | | | | | | | | | | | | | | | | | | | | | |
| Akkam oltan/bultan.Maqaan koo________________________________jedhama .Ministeeraa Fayyaa fi Univaarstii Finfinneen dhimoolee fayyaa adda addaarraatti qoranoo adeemsiisa jiru anis isaan wajjin dhaabbileen fayyaa tajaajila maal maal kenna akka jiran beekuuf kan garagaaru qorannoo hojachaa jira.  Dhaabbatni keessan carraan kan filatamee yammuu ta’u.Gaaffilee waa’ee karoora maatiifi tajaajiloota fayyaa walhoormaata wajjin walqabatan isin gaaffadha akkasumas galmee dhukkubsattotaa ni daawwannaa.Odeeffannii dhaabbata fayyaa kjeessanirra arganu,dhaabbileen fayyaa tajaajila kennaa jiran foyyeessuuf itti fayyadaman akkasumas gareen qorannoo adda addaa qorannoo itti adeemsiisuuf itti fayyadamuu.Maqaan dhaabbata keeesssan nama/dhaabbata kamifuu hin kennamu .gabaasni/bu’aan qorannoo kanaas maqaaነ dhaabbatninkeessan tuqamee kan bahuu osoo hin ta’in ragaa dhaabbilee fayyaa biro walin qindaa’e kan bahudha..  Ragaan sasaabnu hundinu sirri ta’u isa akka nuhubbatan isin gaaffadha.Gaaffilee muraasaaaf nama odeeffannoo gahaaa keenuu danda’u waan wajjin nubarsistaniif isin galateeffadha.  Gaafiif deebii yeroo kamuu dhissuu ykn gaaffii deebisuu hin barbaaadine akka deebistan hindirqamtanu  Qorrannicha ilaalchise wanta nagaaffatan qabdu? | | | | | | | | | | | | | | | | | | | | | | | | | | | | | | | | | | | | | | |
| N | Gaffatamaaf koopii waraqaa waligaltee keennaaf, ibsa godhiif. Itti aansuun akkas jedhi: Gaaffii fi deebii eegaluu danda’aa? | | Eeyyee 1  Miti 0 | | | | | | | | | | | | | | | | | | | | | | | | | | | | | | | | | | | Mitiif gara G.R |
| O | Mallattoo gaaffatamaa  **DEEBII KENNAAN BAKKA KEENNAMEETI AKKA MALLATTEESSAN GAAFADHU** | | MALLATTOO:  Checkbox: ☐ | | | | | | | | | | | | | | | | | | | | | | | | | | | | | | | | | | |  |
| P | Maqaa gaaffataa    **GAAFFATAMAA WALIGAlUU ISA/ISHE RAGAA BAHUUF MAQAAKEE BARREESSI** | |  | | | | | | | | | | | | | | | | | | | | | | | | | | | | | | | | | | |  |
| Q | Maqaa dhaabbata  **MAQAA DHAABBATA BARREESSI** | |  | | | | | | | | | | | | | | | | | | | | | | | | | | | | | | | | | | |  |
| R | **Dhaabbataa kana keessatti gaheenkee maali?**  **LEENJI/SADARKAA BARRNOOTA OL’AANAA GAAFFATAMAAN FUDHATEE GALMEESSAAA** | | Abbaa qabeenyaa 1  Hagganaa/Hogganaa bakka bu’a 2  Hojjataa 3 | | | | | | | | | | | | | | | | | | | | | | | | | | | | | | | | | | |  |
| **LAK** | **GAAFIIFI CALALEESITUU** | | **KOODII** | | | | | | | | | | | | | | | | | | | | | | | | | | | | | | | | | | | **DARBI** |
| **Kutaa 1 Odeeffannoo tajaajiloota Dhaabbatichi keennu**  **section 1 – Information about services**  **Amma dhaabbata fayyaa keessan keessatti tajaajiloota keennaman sigaaffadha** | | | | | | | | | | | | | | | | | | | | | | | | | | | | | | | | | | | | | | |
| 1 | Dhaabbatni fayyaa kun tajaajila fayyaa kannuu kan jalqabe bara kami?  **YOO HIN BEEKU TA’E 2020 BARREESSI** | | Bara | | | | |  | | | | | | | | | | | | | | | | | | | | | | | | | | | | | |  |
| 2 | Dhaabbatni fayyaa keessan yeroo baay’ee torbanitti guyyaa meeqaaf banama?  **LAKKOOFSI BARREEFFAMU 1-7 TA’U QABA**  **YOO HIN BEEKNE ‘88’ BARREESSI** | | Baay’ina guyyoota | | | | |  | | | | | | | | | | | | | | | | | | | | | | | | | | | | | |  |
| 3 | **Ammammoo waa’ee hojjettootaa dhaabbata kana keessatti hojjatan si gaaffadha**  **Dhaabbta fayyaa kana keessatti nama meeqatu ogummaa armaan gaddi kana irratti ramadamanii hojachaa jiru?**    **Guyyaa har’aa kana baay’ina hojjatoota argamnii nati hima?**  Nuti kan barbaadnu leenji adda/barumsa ol’aanaa ogeessi fudhate/argate malee yeroo amma ogeessichi bakka itti ramadamee tajaajila kennaa jiru miti.  **YOO HIN BEEKNE ‘88’ BARREESSI**  **YOO HINILAALTNE ‘77’**  **‘O’ DEEBII TA’U DANDA’A** | | Doktooraa  Nursii/Nursii deesistuu  Qondaala Fayyaa    Oggeesa Faarmaasii    Hojjatoota fayyaa biroo  Hojatoota Ekstenshinii Fayyaa | | | | | | | | | | | | | | | Baay’ina  __  __  __  __  __  __  __  __ | | | | | | | | | | | Har’a kan argaman  __  __  __  __  __  __  __  __ | | | | | | | | |  |
|  | **Gaafii K ILAALLI:**  Gosa dhaabbatichaa? | | Hospitaalaa 1  Bufataa Fayyaa 2  Kellaa Fayyaa 3  Kilinika 4  Faarmaasii 5  Mana kusaa Qorichaa 6  Mana Qoricha Baadiyaa 7  Kan biroo 8 | | | | | | | | | | | | | | | | | | | | | | | | | | | | | | | | | | | yoo I: 5, 6 ykn 7 gara G8 |
| 4 | **Hojjetaan eegumsa fayyaa yeroo hundaa kan argamu ykn waamamee (guyyaatti sa’a 24) hojjetu jiraa?** | | Eeyee,hojataa sa’aati 24 1  Miti, Hojataa sa’aati24 hinqabu 0 | | | | | | | | | | | | | | | | | | | | | | | | | | | | | | | | | | |  |
| 5 | **Dhaabbatni fayyaa kun tilmaamaan namoota meeqaaf tajaajila kennuuf karoorfate,ykn baay’ina uummataa dhaabbata kanaan tajaajilaman niqabdu?** | | Dhaabbatchi iddoo/uummata murtaa’e hinqabu 1  Eeyyee dhaabbatichi ummata tajaajilu nibeeka 2  ummata tajaajiluu fi iddoo murataa’e hin beekamu 3 | | | | | | | | | | | | | | | | | | | | | | | | | | | | | | | | | | | 1 fi 3 yoo ta’e gara G 7 |
| 6 | Baay’inni uummata dhaabbatni kun tajaaajilu( catchment population) meeqa?  **NAMOOTA NAANNOO KANAA DHAABBATA KANAAN TAJAAJILAMAN GALMEESSI** | | Baay’ina uummata | | | | |  | | | | | | | | | | | | | | | | | | | | | | | | | | | | | |  |
| 7 | **Dhaabbatichi kun siree ciisichaa dhukubsatootaaf ta’u meeqa qaba?**  **‘O’ DEEBII TA’U DANDA**  **.**  **YOO HIN BEEKU TA’E 88 BARREESSI** | | Baay’ina siree | | | | |  | | | | | | | | | | | | | | | | | | | | | | | | | | | | | |  |
| 8 | Abbaan qabeenyaa ykn to’ataan qaama alaa irraa dhufee yoomi yeroo dhumaaf kan isin daawwate? | | to’ataan qaama alaa hin dhufne 0  ji’a jeha keessaa 1  ji’a jeha oli 2  Hinbeeku -88 | | | | | | | | | | | | | | | | | | | | | | | | | | | | | | | | | | |  |
| 9 | Dhaabbatni kun ibsaa ni qabaa? | | Eeyyee 1  Miti 0 | | | | | | | | | | | | | | | | | | | | | | | | | | | | | | | | | | |  |
| 10 | Dhaabbatni kun bishaan ni qabaa? | | Eeyyee 1  Miti 0 | | | | | | | | | | | | | | | | | | | | | | | | | | | | | | | | | | |  |
|  | GAAFFII K ILAALLI:  Gosa dhaabbatichaa? | | Hospitaalaa 1  Bufataa Fayyaa 2  Kellaa Fayyaa 3  Kilinika 4  Faarmaasii 5  Mana kusaa Qorichaa 6  Mana Qoricha Baadiyaa 7  Kan biroo 8 | | | | | | | | | | | | | | | | | | | | | | | | | | | | | | | | | | | yoo I: 5, 6 ykn 7 gara G.13 |
| 11 | Yeeroo ammaa tana dhaabbata fayyaa kana keessatti iddoon harka dhiqachuuf tajaajilan meeqatu jira?  **YOO HIN BEEKU TA’E 88 BARREESSI** | | Baay’ina iddoo harka dhiqachuuf tajaajilan | | | | | | | |  | | | | | | | | | | | | | | | | | | | | | | | | | | | 0’’ gara G 13 |
| 12 | Bakka itti harka dhiqatan kan dhihoo akka siif agarsiisan gaaffadhu:  KANNEEN ARMAAN GADDI JIRAACHU ISAANI DAAWWADHU:  Saamunaan ni jiraa  Bishaan kuufame ni jiraa?  Bishaan boombaa ni jiraa  Bakki itti harka dhiqatan mana boolii/Fincaanii waliin walbiraa?  Wantoota armaan oli hinjiranu  Iddon harka itti dhiqatan hinarginee  **KAN EERAN HUNDA GALMEESSAA** | |  | | | | | Eeyyee  1  1  1  1  -88  1 | | | | | | | | | | | | | | | Miti  0  0  0  0  0 | | | | | | | | | | | | | | |  |
| 13 | Dhaabbatni kun kompitara hojjetu ni qabaa?  **LAALUUN HINBARBAACHIISUU**  **NO NEED TO OBSERVE** | | Eeyyee 1  Miti 0 | | | | | | | | | | | | | | | | | | | | | | | | | | | | | | | | | | |  |
|  | **GAAFFII K ILAALLI**:  Gosa dhaabbatichaa? | | Hospitaalaa 1  Bufataa Fayyaa 2  Kellaa Fayyaa 3  Kilinika 4  Faarmaasii 5  Mana kusaa Qorichaa 6  Mana Qoricha Baadiyaa 7  Kan biroo 8 | | | | | | | | | | | | | | | | | | | | | | | | | | | | | | | | | | | Skip to 15 if I: 5, 6 or 7 |
| 14 | Dhaabbatni kun meeshaalee qara qabaniif kaartoona meshaaleen qara qaban itti kuufanman dhuuma irratti akkamittin dhabamsiisa/barbadeessa? | | Meeshaalee qara qaban hiqabanu 0  Insinareetara/Barmeela keesati niguba 1  Dirreetti niguba 2  Osoo higubin nigatu 3  Iddoo hin mul’aneetti nigatamaa 4  Kan biroo 5 | | | | | | | | | | | | | | | | | | | | | | | | | | | | | | | | | | |  |
| **Kutaa 2 Waa’ee Tajaajiloota Karooraa Maatii**  **Amma dhaabbata fayyaa keessan keessa tajaajiloota karoora maatii keennaman sigaaffadha.** | | | | | | | | | | | | | | | | | | | | | | | | | | | | | | | | | | | | | | |
| 15 | **Tajaajila mala karoorakaroora maatii ni kennitani?** | Eeyyee 1  Miti 0 | | | | | | | | | | | | | | | | | | | | | | | | | | | | | | | | | | | 0 yoo ta’e gara G. 19 | |
| 16 | **Tajaajilli karoora maatii dhaabbatichi kun bara kam irra eegale kennuu jalqabee?**  **YOO HINBEEKNE 2020GALCHA** | Bara | | | | | | | | |  | | | | | | | | | | | | | | | | | | | | | | | | | |  | |
| 17 | **Torbanitti guyyaa meeqaaf**  **tajaajilli karoora maatii asitti kennaman/gurguraman**  **BAAY’INA GUYYOOTA TORGAANITI GUYYAA TORBA.**  **LAKOOFISA 1-7 QOFA GALCHA.**    **YOO HINBEEKNE 88 GALCHA** | Baay’na guyyoota | | | | | | | | |  | | | | | | | | | | | | | | | | | | | | | | | | | |  | |
| 18 | **Tajaajilli karoora maatii har’a dhaabata kana keesatti kennamaa jiraa?** | Eeyyee 1  Miti 0 | | | | | | | | | | | | | | | | | | | | | | | | | | | | | | | | | | |  | |
|  | **GAAFFII J ILAALLI**:  Gosa dhaabbatichaa? | Hospitaalaa 1  Bufataa Fayyaa 2  Kellaa Fayyaa 3  Kilinika 4  Faarmaasii 5  Mana kusaa Qorichaa 6  Mana Qoricha Baadiyaa 7  Kan biroo 8 | | | | | | | | | | | | | | | | | | | | | | | | | | | | | | | | | | | yoo I: 5, 6 ykn 7 gara G23 | |
| 19 | Does this facility provide family planning supervision, support, or supplies to community health volunteers?  **Dhaabbani kun dhiheessaa qoorichaa ,to’annoo fi gargaarsa karoora maatii, tolaoltoota fayyaa hawaasaatiif(ni kennaa?** | Eeyyee 1  Miti 0 | | | | | | | | | | | | | | | | | | | | | | | | | | | | | | | | | | | Miti yoo ta’e gara G.22 | |
| 20 | **Tola oltoota fayyaa hawaasaa meeqaatu dhaabbata keessaniin garagaarsa argatan?**  **ENTER -88 FOR DO NOT KNOW.** | Baay’ina CHWs | | | | | | | | | |  | | | | | | | | | | | | | | | | | | | | | | | | |  | |
| 21 | **Tola oltooni fayyaa hawaasaa**  tajaajila karoora maatii kanneen ni kennuu    Kondomaa  Kiniinaa/Pilsii  Lilmoo |  | | | | | | | | | | | | | | | | | | Eeyye  1  1  1 | | | | | | | | | | | | Miti  0  0  0 | | | | |  | |
| 22 | **Ji’oota 12 darbanitti gareen tajaajila karoora maatii kennuu, dhaabbata kana daawwatanii beekuu?** | Baay’ina yeroo  Number of times: | | | | | | | | | | | | | | | | | |  | | | | | | | | | | | | | | | | |  | |
|  | **CHECK 15: dhaabbatich tajaajila karoora maatii ni kennaa**? | Eeyyee 1  Miti 0 | | | | | | | | | | | | | | | | | | | | | | | | | | | | | | | | | | | Miti yoo ta’e gara G.25 | |
| 23 | **Dhaabbani kun tajaajila idilee kennu kamiifuu ykn tajaajila karoora maatiitin walqabateef kaffaltii ni kaffalchiisaa?**  **KAFALTIIN KUN KAFALTTI TAJAJILA MANA YAALA KAMUU FI KAARDIIF KAN BAASAN NIDABALATAA.** | Eeyyee 1  Miti 0 | | | | | | | | | | | | | | | | | | | | | | | | | | | | | | | | | | | Miti yoo ta’e gara G.25 | |
| 24 | **Gatiin kun maamilaan hundu akka arguu bakka mul’atutti maxxanfameeraa?**  **EEYYEE YOO TA’EE GATII MAXXANFAMEE ILAALAA** | Eeyeen gatiin hundinu maxxanfameerra 1  Eeyyeen gatiin muraasaa maxxanfameerra 2  Gatiin gonkumma hin maxxanfamne 0 | | | | | | | | | | | | | | | | | | | | | | | | | | | | | | | | | | |  | |
| 25 | Yaada maamiltoota keessani bifa kamini walti qabdusasaabdanu?  **Sanduuqa yaadaa**    **Foormii qo’annoo maamiltootaa**    **Foormii gaafannoo maamiltootaa**    **Walgahii idilee maamiltoota walin/ dursitoota uummataa waliin**    **Marii idileen alaa maamiltoota walii**  Hin beeku  Kan eeraman ala  **KAN ILAALATU HUNDA FILADHU** |  | | | | | | | | | | | | | | | | | | Eeyye  1  1  1  1  1  1  1  -88  1 | | | | | | | | | | | | Miti  0  0  0  0  0  0  0  0 | | | | | Kan eeran ala f gara G 29 | |
| 26 | Adeemsa yaada maamiltootaa ilaalanii kan itiitn qorattan/madaaltan qabdu? | Eeyyee 1  Miti 0 | | | | | | | | | | | | | | | | | | | | | | | | | | | | | | | | | | | Miti yoo ta’e gara G.28 | |
| 27 | Foormii odeeffannoon/mariin irratti barreeffamu/gabaasae akka siif agarsiisan gaaffadhu. | GabaasnI laalamerra 1  Gabaasni ilaalamne 2 | | | | | | | | | | | | | | | | | | | | | | | | | | | | | | | | | | |  | |
| 28 | Yaada maamiltootaa irra kaun n ji’oota 12 darbanitti kennannin sagantaa keessanwanti jijjiirame ni jiraa?  **EEYYEEN YOO TA’E,JIJJIRAMN KAN IBSAN WAJJIN WALSIMU ISA MIRKANEEFEDHA** | Miti 0  Eeyyee, haala ,akaakuun fi yeroon tajaajjilli itti keennamu foya’erra ykn halli tajaajilli itti krnnamu 1  Maamiltootaaf haala mijaawaa ummameer 2  kanbiroo 3  Hinbeeku -88 | | | | | | | | | | | | | | | | | | | | | | | | | | | | | | | | | | |  | |
| 29 | Ji’oota 12 darbanitti, walgahiin waa’ee baay’ina tajaajila karoora maatii hojjetoota waliin mariin godhame turee? | Eeyyee 1  Miti 0 | | | | | | | | | | | | | | | | | | | | | | | | | | | | | | | | | | |  | |
| 30 | Tajaajila karoora maatii hordofuuf,madaaloof Do you use any of the following to review service data for monitoring and evaluation?  Chaartii/graafii(Wall chart / graph)  Gabaasaa bareeffamaa /qaboo yaa’I Written report / minutes  Kan biroo  Wanti laallame hinjiru    **GABAASAA,GIRAAFII,CHAARTII ODEEFFANNOON TAJAAJILLI SIRRITTI MADAALAMU ISAANI MIRKANEESAA .WANATA ARGITAN KEESSA DOKUMEENTII SIRRII TA’EE FILADHA.** |  | | | | | | | | | | | | | | | Eeyee  1  1  1  1 | | | | | | | | | | | | | Miti0  0  0  0 | | | | | | |  | |
|  | **G15 ILAALI: Dhaabbatichi tajaajila qosannoo/karooraa maatii nikeennaa?** | Eeyyee 1  Miti 0 | | | | | | | | | | | | | | | | | | | | | | | | | | | | | | | | | | | Miti yoo ta’e gara G40 | |
| 31 | Dubarti maseensuu  Dhiira maseensuu  Gadameesa keesa kan ta’u/luupii/  Hawollee hosiisaniif kiniini  Lilmoo/ Injectables ji’a 3  Kan irree jalati awaalamu /Implaanti/  Kininaa (pilsii)  Kondomii dhiiraa  Kondomii dubaraa  Ittisa ulfaa yeroo haatatamaa(EC    Foam/jellly  Mala guyyaa lakkaa'u /Callee lakaa’udhaan)  Daa’ima harmaa haadhaa qoofa hosiisuudhaan (LAM)  Guyyaa lakaudhaan (Calendar method)  Dhangala’a dhiiraa alati jiksuu  Mala aadaa biroo  Qooqa keesan ol-fudhaati fillanoo hundaa duubisaaf | Cou  Yes  1  1  1  1  1  1  1  1  1  1  1  1  1  1  1  1  1 | | Cou  No  0  0  0  0  0  0  0  0  0  0  0  0  0  0  0  0  0 | | Pro  Yes  1  1  1  1  1  1  1  1  1  1  1  1  1  1 | | | Pro  No  0  0  0  0  0  0  0  0  0  0  0  0  0  0 | | | | | | Pre  Yes  1  1  1  1  1  1  1  1  1  1  1  1  1  1 | | | | | | | | | Pre  No  0  0  0  0  0  0  0  0  0  0  0  0  0  0 | | | | | | | | | | | | Chg  Yes  1  1  1  1  1  1  1  1  1  1  1  1  1  1 | Yoo hinkafalchisne Gara | |
| 32 | **Tajaajila karoora maatii tokko tokko keennitaniif altokkoof meeqa kaffalchiistan?**  Dubarti maseensuu  Dhiira maseensuu  Gadameesa keesa kan ta’u/luupii/  Hawollee hosiisaniif kiniini  Lilmoo/ Injectables ji’a 3  Kan irree jalati awaalamu /Implaanti/  Kininaa (pilsii)  Kondomii dhiiraa  Kondomii dubaraa  Ittisa ulfaa yeroo haatatamaa(EC    Foam/jellly  Mala guyyaa lakkaa'u /Callee lakaa’udhaan)  Daa’ima harmaa haadhaa qoofa hosiisuudhaan (LAM)  Guyyaa lakaudhaan (Calendar method)  Dhangala’a dhiiraa alati jiksuu  Mala aadaa biroo  Malaqqa kafalamu BIRRII fi SAATIMAAN KA’A.  *Tajaajilli dhaabbatichii kafalchisuu qoofa ODKn gaaffii 31 irraa ni mul’isa.* | Kafalti al tokkoo | | | | | | | | | | | | | | | | | | | | | | _________  _____________________________________________________________________________________________________________________ | | | | | | | | | | | | |  | |
|  | **Gaafi K ILAALLI**  Gosa dhaabbaticha? | Hospitaalaa 1  Bufataa Fayyaa 2  Kellaa Fayyaa 3  Kilinika 4  Faarmaasii 5  Mana kusaa Qorichaa 6  Mana Qoricha Baadiyaa 7  Kan biroo 8 | | | | | | | | | | | | | | | | | | | | | | | | | | | | | | | | | | | yoo I: 5, 6 ykn 7 gutame gara G39b | |
|  | **G 31 ILAALLI Kan irree jalatii ka’amu** (Inplaantiin) galeef jira? | Eeyyee 1  Miti 0 | | | | | | | | | | | | | | | | | | | | | | | | | | | | | | | | | | | Miti yoo ta’e gara G. 35 | |
| 33 | Dhaabbatichi karoora maatii kana yammuu kennu ogeesssa leenji implaantii awaalu/galchu kan fudhate nijira. | Eeyyee 1  Miti 0 | | | | | | | | | | | | | | | | | | | | | | | | | | | | | | | | | | |  | |
| 34 | Dhaabbatichi karoora maatii kana yammuu kennu ogeesssa fayyaa implaantii awaalamee baasuu leenjjii fudhate nijira/niqabaa? | Eeyyee 1  Miti 0 | | | | | | | | | | | | | | | | | | | | | | | | | | | | | | | | | | |  | |
|  | **G 31 ILAALLI Kan gadaamees keesa taa’u (luuppiin)**  keenameefi jira? | Eeyyee 1  Miti 0 | | | | | | | | | | | | | | | | | | | | | | | | | | | | | | | | | | | Miti yoo ta’e gara G. 37 | |
| 35 | Dhaabbatichi tajaajila karoora maatii (luuppii) kana yammuu kennu ogeesssa luuppii galchu irrati leenjjii fudhate nijira/niqaba?. | Eeyyee 1  Miti 0 | | | | | | | | | | | | | | | | | | | | | | | | | | | | | | | | | | |  | |
| 36 | Dhaabbatichi tajaajila karoora maatii (luuppii) kana yammuu kennu ogeesssa luuppii baasuu irrati leenjjiifudhate nijira/niqaba? | Eeyyee 1  Miti 0 | | | | | | | | | | | | | | | | | | | | | | | | | | | | | | | | | | |  | |
|  | **G 31 ILAALLI Kan irree jalati kaa’yamuu (**Inplaantiin) galeef jira? | Eeyyee 1  Miti 0 | | | | | | | | | | | | | | | | | | | | | | | | | | | | | | | | | | | Miti yoo ta’e gara G. 38 | |
| 37 | **Dhaabbatni keessan meeshaalee IMPLAANTII galchuufi ykn baasuuf gargaaran kanneen ni qabaa?**  Glaavii qulquluu  Fara ilbiisaa (Antiseptic)  Jiirbii ykn wacuu ququlluu  Hadoochiitu (Local Anesthetic)  IMPLAANTII Saamsamee (Sealed Implant Pack)  Milaacii/muurtuu (Blade)  **MEESHAALEE HUNDAA QOOQA KESSAN OLKAASUUN GAAFADHA KAN EERAN HUNDA GALMEESSA. MEESHAALEE LAALUUN HIN BARBAACHISUU** |  | | | | | | | | | | | | | | | | | Eeyyee  1  1  1  1  1  1 | | | | | | | | | | | | Miti  0  0  0  0  0  0 | | | | | |  | |
|  | **G 31 ILAALLI kan gadaameesa keesa kaa’yamu (luuppiin)** keenameefi jira? | Eeyyee 1  Miti 0 | | | | | | | | | | | | | | | | | | | | | | | | | | | | | | | | | | | Miti yoo ta’e gara G. 39 | |
| 38 | Dhaabbatni keessan meeshaalee luuppii galchuufi/baasuuf gargaaran kanneen ni qabaa?  Maqasii isponjjii ittin qaban (Sponge-holding forceps)  Meeshaa naf-saalaa keesa ilaaluf gargaaru _guddaa fi gidu-galeesa/Speculums (large and medium)  Sibila afaan gadaameesaa qabuuf kan tajaajilu /Tenaculum/  Foon waliti-qabuuf kan tajaajilu /Clamp/  **QOOQA KEESSAN OLKAASUUN GAAFADHA KAN EERAN HUNDA GALMEESSA.MEESHAALEE LAALUUN HIN BARBAACHISUU** |  | | | | | | | | | | | | | | | | | Eeyyee  1  1  1  1 | | | | | | | | | | | | Miti  0  0  0  0 | | | | | |  | |
|  | **CHECK K:** type of facility?  **Gosa tajaajila bu’uuraa** | \| Kafalti al tokkoo \| _________  _____________________________________________________________________________________________________________________ \| \| --- \| --- \| \| Hospitaalaa 1  Bufataa Fayyaa 2  Kellaa Fayyaa 3  Kilinika 4  Faarmaasii 5  Mana kusaa Qorichaa 6  Mana Qoricha Baadiyaa 7  Kan biroo 8 \| \| | | | | | | | | | | | | | | | | | | | | | | | | | | | | | | | | | | | 39a if I: 1-4,or 8  39b if I: 5, 6 or 7 | |
| **39a** | **GALMEE KAROORAA MAATII IRRA GALMEESSAA**   1. Ji’a darbee keessaa tajaajila karoora maatii jechuun maloota hundaaf daawwannaa godhame baay’ina maamiltoota(haaraa fi yeroo hunda) galmeessaa.   (maloota hundaaf tokko tokkoon gaaffadha.   1. ji’a darbee keessattibaay’ina maamiloota haaraa galmeessaa maloota hundaaf tokko tokkoon addan baafachuun gaafadha. | Dubarti maseensuu  Dhiira maseensuu  Gadameesa keesa kan ta’u/luupii/  Hawollee hosiisaniif kiniini  Lilmoo/ Injectables ji’a 3  Kan irree jalati awaalamu /Implaanti/  Kininaa (pilsii)  Kondomii dhiiraa  Kondomii dubaraa  Ittisa ulfaa yeroo haatatamaa(EC    Foam/jellly  Mala guyyaa lakkaa'u /Callee lakaa’udhaan)  Daa’ima harmaa haadhaa qoofa hosiisuudhaan (LAM)  Guyyaa lakaudhaan (Calendar method)  Dhangala’a dhiiraa alati jiksuu  Mala aadaa biroo | | | | | | | | | | | | baay’ina  dawwanna  ___  ___  ___  ___  ___  ___  ___  ___  ___  ___  ___  ___  ___  ___ | | | | | | | | | | | | | | Baay’inaMaam.haraa  ___  ___  ___  ___  ___  ___  ___  ___  ___  ___  ___  ___ | | | | | | | | |  | |
| **39b** | **GALMEE KAROORAA MAATII IRRA GALMEESSAA**  Ji’a darbee keessatti maloota karoora maatii gurguraman hundaa tokko tokkoon addan baafachuun galmeessaa  . | Gadameesa keesa kan taa’u/luupii/  Hawollee hosiisaniif kiniini  Lilmoo/ Injectables ji’a 3  Kan irree jalati awaalamu /Implaanti/  Kininaa (pilsii)  Kondomii dhiiraa  Kondomii dubaraa  Ittisa ulfaa yeroo haatatamaa(EC  Mala guyyaa lakkaa'u /Callee lakaa’udhaan)  Mala aadaa biroo | | | | | | | | | | | | | | | | | | | | | | | | Maloota gurguraman  ___  ___  ___  ___  ___  ___  ___  ___  ___  ___  ___  ___ | | | | | | | | | | |  | |
|  | **GAAFFII K ILAALLI**:  Gosa dhaabbatichaa? | \| Kafalti al tokkoo \| _________  _____________________________________________________________________________________________________________________ \| \| --- \| --- \| \| Hospitaalaa 1  Bufataa Fayyaa 2  Kellaa Fayyaa 3  Kilinika 4  Faarmaasii 5  Mana kusaa Qorichaa 6  Mana Qoricha Baadiyaa 7  Kan biroo 8 \| \| | | | | | | | | | | | | | | | | | | | | | | | | | | | | | | | | | | | 1,5,66 fi 7 Gara  G 45 | |
| 40 | Tajaajiloota kanneen keessa kamtu dhaabbata kana keessaatti kennaman?  :  Hordoffii da’umsa dura  Tajaajila da’umsa  Hordoffii da’umsa booda  Tajaajila ulfa baasuun booda Post-abortion  **DEEBII HUNDA DUBBISUN KAN EERAN HUNDA GALMEESSAA.** |  | | | | | | | | | | | | | | Eeyyee  1  1  1  1 | | | | | | | | | | | | | | | | | | Miti  0  0  0  0 | | | Hor daums/ulfa baasuu booda Miti yoo ta’e gara G45  Hor daumsa booda miti yoo ta’efi baasaa booda eeyyee yaa ta’e gara G43 | |
| 41 | Kanneen keessaa haati deessuun mucaashee fudhattee bahu ishi dura kamtu itti himama?  Nyaata,haala nyaata fi sochii qaama godhuu  Bowoo mataa da’umsa booda mudachuu danda’u  Haala irra deebite dayuu dandessuu  Addan fageessani dayuuf yeroon gaarii kam akka ta’e  **Gorsa Maloota karoora Maatiif**:  Daa’ima harma qoofa hoosisudhaan /LAM?  Guyyaa xuriin itti dhufuu lakayuun  Maloota yeroo dheeraaf tajaajilan  Maloota adaan fageessani dayuu  Kan ibsame keessa hn jiru  **DEEBII HUNDA DUBBISUN KAN EERAN HUNDA GALMEESSAA** |  | | | | | | | | | | | | | | Eeyyee  1  1  1  1  1  1  1  1 | | | | | | | | | | | | | | Miti  0  0  0  0  0  0  0  0 | | | | | | |  | |
| 42 | **Dubartooni hordooffi dahumsa boodaaf gara dhaabbata fayyaa yammuu dhufan mala karoora/ karoora maatii ni keennamaaf?** | Eeyyee 1  Miti 0 | | | | | | | | | | | | | | | | | | | | | | | | | | | | | | | | | | |  | |
|  | **G.40 ILAALLI:** tajaajila ulfa baasuun booda ni kennamaa? | Eeyyee 1  Miti 0 | | | | | | | | | | | | | | | | | | | | | | | | | | | | | | | | | | | Miti yoo ta’e gara G45 | |
| 43 | Daawwanna ulfa baasuu n booda maamila keessan wajjin kan armaan gadii keessa kam walin mar’atan:  Bowoo mataa ulfa baasuu booda mudachuu danda’u  Haala irra deebite dayuu dandessuu  Addan fageessani dayuuf yeroon gaarii kam akka ta’e  Gorsa karoora Maatiif:  Lactational Amenorrhea Method  Maloota yeroo dheeraaf tajaajilan  Maloota adaan fageessani dayuu  Kan ibsame keessa hn jiru  **DEEBII HUNDA DUBBISUN KAN EERAN HUNDA GALMEESSAA** |  | | | | | | | | | | | | | | | | | | | Eeyye  1  1  1  1  1  1 | | | | | | | | | | | | Miti  0  0  0  0  0  0 | | | |  | |
| 44 | **Dubartooni hordooffi dahumsa boodaaf gara dhaabbata fayyaa yammuu dhufan mala karoora/ karoora maatii ni keennamaaf?** | Eeyyee 1  Miti 0 | | | | | | | | | | | | | | | | | | | | | | | | | | | | | | | | | | |  | |
| 45 | Dargaggoota hin fuune ykn hinheerumneef tajaajiloota karoora maatii isa kam kennitu?  Gorsa karaaoraa maatii  Karoora maatiin kennuu  Ajaaja barreesuu/ karoora maatiif rifarii  Kan ibsame keessa hn jiru  **DEEBII HUNDA DUBBISUN KAN EERAN HUNDA GALMEESSAA** |  | | | | | | | | | | | | | | | | | | | | Eeyye  1  1  1  1 | | | | | | | | | | | | Miti  0  0  0  0 | | |  | |
| 46 | Dhabbatni kuni dhukkuboota qunnamti saalaatiin darbaniif tajaajila qorannoo,gorsaafi yaala ni kennaa? | Eeyyee 1  Miti 0 | | | | | | | | | | | | | | | | | | | | | | | | | | | | | | | | | | |  | |
| 47 | Dhaabbatni kuni tajaajila qorannoo, gargaarsaa fi wal’aansa dhibee HIV dhaaf ni kennaa? | Eeyyee 1  Miti 0 | | | | | | | | | | | | | | | | | | | | | | | | | | | | | | | | | | | Miti yoo ta’e gara G48 | |
|  | **GAAFFII K ILAALLI**:  Gosa dhaabbatichaa? | \| Kafalti al tokkoo \| _________  _____________________________________________________________________________________________________________________ \| \| --- \| --- \| \| Hospitaalaa 1  Bufataa Fayyaa 2  Kellaa Fayyaa 3  Kilinika 4  Faarmaasii 5  Mana kusaa Qorichaa 6  Mana Qoricha Baadiyaa 7  Kan biroo 8 \| \| | | | | | | | | | | | | | | | | | | | | | | | | | | | | | | | | | | | yoo I: 5, 6 ykn 7 gara G 52 | |
| 48 | Maamila dhibee HIV dhaan fudhachuuf dhufeef mala karoora maatii isa kam kennitu?  Gorsa karaaoraa maatii  Karoora maatiin kennuu  Ajaaja barreesuu/ karoora maatiif rifarii  Kan ibsame keessa hn jiru  **DEEBII HUNDA DUBBISUN KAN EERAN HUNDA GALMEESSAA** |  | | | | | | | | | | | | | | | | | | eeyyee  1  1  1  1 | | | | | | | | | | | | Miti  0  0  0  0 | | | | |  | |
| 49 | Maamila gorsa dhibee HIV fudhachuuf dhufaniif ogeessi waa’ee walhormaatafi haala ufduraati da’imma argachuu danda’a isaan gaaffatee turee?    Maamiltooni maloota Karoora filatan mar’achisee turee?  Maloota lamaa fayyadamuu akka danda’an mari’achisee turee?  Kondomaa kennee turee?  Malaata filaniif qajeelfamafi gorsa malli rokkoo fiduu danda’an mar’achisee turee?  Mala karoora maatii kennee? |  | | | | | | | | | | Yes  1  1  1  1  1  1 | | | | | | | | No  0  0  0  0  0  0 | | | | | | | | | | | | DK  -88  -88  -88  -88  -88  -88 | | | | |  | |
|  | **G 15 ILAALLI** tajaajila karaaora maatii ni kennuu? | Eeyyee 1  Miti 0 | | | | | | | | | | | | | | | | | | | | | | | | | | | | | | | | | | | Miti yoo ta’e gara  G.R | |
| 50 | **KUTAA QORANNOO KAROORA/KAROORA ITTI ADEEMSIIFAMU AKKA SIIF AGARSIISAN GAAFFADHU**  **MEESHAALEE KANNEEN KUTAA QORANNOON ITTI ADEEMSIIFAMUU KEESSAATTI JIRAACHUU ISAANI GAAFFADHU.**  [MEESHAALEE INFEEKSHIINII TO’ACHUUF GARGAARAN JIRAACHUU ISAANI ILAALLI  O: laalameera/Observed; RU: gabaafame,hin ilaalamne/Reported, Unseen; NA: Hinjiru/Not Available | Bishaan boobaa (piped)  Bishaan booba ta’ee Other running water (bishaan jariikaanaa bucket with tap or pour pitcher)  Bishaan jarikaanaa ykn meeshaa biroo Water in bucket or basin (water reused)  Saamuunaaa haraka Hand-washing soap  Fooxaa al tokkoof tajaajilu Single-use hand drying towels  Pilaastiikaa balfaa qadaada qabu Waste receptacle with lid and plastic liner  Meeshaalee qara qaban Sharps container  Guwaantii altokkoof tajaajiluuDisposable latex gloves  Qoricha infekshiini Disinfectant  Lilmoo altokoof qofa tajaajiluu Disposable needles and syringes  Kutaan qorannoo sagaalee hingalchine Auditory privacy  Kutaan qorannoo argaaf hinsaxilamne Visual privacy  Minjaala qoraannoo Examination table  Meeshaalee karooraa maatii barsiisuuf tajaajilan Client educational materials on FP | | | | | | | | | | | | | O  1  1  1  1  1  1  1  1  1  1  1  1  1  1 | | | | | | | | | RU  2  2  2  2  2  2  2  2  2  2  2  2  2  2 | | | | | | | | | | | | NA  -77  -77  -77  -77  -77  -77  -77  -77  -77  -  77  -77  -77  -77  -77 |  | |
| 51 | **IDDOO KAROORAA MAATII ITTI KEENNAMU ILAALLI** | Lafa Floor: qulqulluu balfa mul’atu hinjiru  Minjaala/baankooni/ Counters /teessoo qulqulluu balfa mul’atu hinjiru  Meeshaalee cacabboo,waraqaa,kartoonaa fi balfa adda addda iddoo hojiin hojjatamuutti nijira  Gidgiga Walls: hanga murta’e qulqullu  Balbala: hinqabu ykn caba xiqoo qaba no or Fodaa kan caccabee  Bdaabaa/gidgidaa: hin caccabine  Baaxii: hinqabu ykn iddoo xiqqootti digameerra | | | | | | | | | | | | | | | | | | | | | Yes  1  1  1  1  1  1  1 | | | | | | | | | | | | No  0  0  0  0  0  0  0 | |  | |
| 52 | Tajaajila armaan gadii akka keennitan natti himtanirrtu.  ARGUU DANDA’A.  MALOOTA ARGITAN HUNDA GAAFFILEE ARMAAN GADII GAAFFADHAAF  Ji’oota 12 darban keessaa qooricha kaaroora maatii isin irra dhume/kusaa keessan keessaa dhabamee ni beeka?  Gadameesa keesa kan taa’u/luupii/  Hawollee hosiisaniif kiniini  Lilmoo/ Injectables ji’a 3  Kan irree jalati awaalamu /Implaanti/  Kininaa (pilsii)  Kondomii dhiiraa  Kondomii dubaraa  Ittisa ulfaa yeroo haatatamaa(EC  Mala guyyaa lakkaa'u /Callee lakaa’udhaan)  Mala aadaa biroo      O: lalameera/Observed; N.O.: hin ilaalamne/Not Observed; OOS last 12 mo.: ji’oota 12 keessa dhabame/Out of stock in last 12 months  **MALLI KAROORRI MAATII JI’OOTA 12 DARBAN KEESSAA MANA KUSAA KEESSA DHABAMEE GARUU YAMMUU QORANNICHI ADEEMSIIFAMUU YOO JIRAATE DHA.**  **N.O YOO FILATAME MANA KUUSAA KEESSAA AKKA DHUME BEEKAMU QABA .GARUU BEEBIIN LAMAANU FILATAMU HINQABU.**  *ODKN SQ ti tajaajiloota dhaabbaticha keessaa kennaman qofa mul’isa* |  | | | | | O  1  1  1  1  1  1  1  1  1  1  1  1 | | | | | | N.O  0  0  0  0  0  0  0  0  0  0  0  0 | | | | | | | | | | | | | | Ji’oota 12 keesati yeero meeqa dahbame  1  1  1  1  1  1  1  1  1  1  1  1 | | | | | | | | | |  | |
| 53 | **GAAFFILEE 53-56 ARMAAN GADI JIRANIIF IDDO QORICHA KAROORA MAATII ITTI KUFAMAN ILAALUUN KAN GUUTAMANI DHA**  Qorichii/ Maloonni karoora maatii hundinu dirree/ lafa irrati nijiru? | Eeyyee 1  Miti 0 | | | | | | | | | | | | | | | | | | | | | | | | | | | | | | | | | | |  | |
| 54 | Are all the methods protected from water?  Qorichii/Malloonni karoora maatiihunduu bishaan irraa kan eegamenidha? | Eeyyee 1  Miti 0 | | | | | | | | | | | | | | | | | | | | | | | | | | | | | | | | | | |  | |
| 55 | Qorichii/Malloonni hunduu aduu irraa kan eegamanidha? | Eeyyee 1  Miti 0 | | | | | | | | | | | | | | | | | | | | | | | | | | | | | | | | | | |  | |
| 56 | Kutaan kun qulqulluudhaa, akasumas bineensotaa fi ilbiisota adda addaa kan akka hantuutaa, bararoo fi kkf irraa bilisa dhaa? | Eeyyee 1  Miti 0 | | | | | | | | | | | | | | | | | | | | | | | | | | | | | | | | | | |  | |
| .  Gaafatamaa sana galateeffadhu  **GAAFFI FI DEEBIIN XUMURAMEERRA GARUU GAAFFILEE SADEE SEENSA DHAABBATICHA ALARRATTI TAATA’NI FUUNAANUU KAN QABDAN NIJIRu.**  **.** | | | | | | | | | | | | | | | | | | | | | | | | | | | | | | | | | | | | | | |
| **IDDOO QORAANNOO ITTI ADEEMSIFAMEE FI BU’A QORANNICHA** | | | | | | | | | | | | | | | | | | | | | | | | | | | | | | | | | | | | | | |
| S | Naannoo seensa dhaabbatichaatti safartuu koordin GPSii fudhadha  Safartuun GPSii sirrii kan ta’u meetraa 6 gadi yoo ta’e  .  **KOORDINEETII GPSII FUDHATAMU KAN QABU MANA ALA TAATANI TA’U QABA** | | *ODKn qajeelfama isiniif kennaa*  IDDO SANA GALMEESI | | | | | | | | | | | | | | | | | | | | | | | | | | | | | | | | | | |  |
| S a | Suuraa kaaasuuf eeyyama gaaffadha  Suuraa kaasuuf eeyyama argatani? | | Eeyyee 1  Miti 0 | | | | | | | | | | | | | | | | | | | | | | | | | | | | | | | | | | | Miti yoo ta’e gara T |
| S b | Namni kamuu suuraa keessatti akka hinjiraane mirkaneefadha | | *ODKn qajeelfama isiniif kennaa*  SUURAA FUDHADHAA  FAKKII FILADHA | | | | | | | | | | | | | | | | | | | | | | | | | | | | | | | | | | |  |
| T | Bu’a gaaffiif deebii dhaabbatichaa | | Xumuramee 1  Dhaabani hinargamne 2  Yeroo biraaf dabarfame 3  adeemsisuuf didan 4  Gariin xumuramee 5  Kan biroo 6 | | | | | | | | | | | | | | | | | | | | | | | | | | | | | | | | | | |  |
