# Supplementary material for: Does proximity of women to facilities with better choice of contraceptives affect their contraceptive utilization in rural Ethiopia?
Source: PLoS One. 2017 Nov 13;12(11):e0187311. doi: 10.1371/journal.pone.0187311 (PMC5683563; doi:10.1371/journal.pone.0187311)
Supplement: S1 File — (ZIP) [file pone.0187311.s004.zip › Questionnaires/Amharic version/SDP_Amharic_14 Nov 13.docx]

| **mADDS –የአገልግሎት መስጫ ቦታ (SDP) መጠይቅ** |
| --- |

| **ተ.ቁ** | **ጥያቄዎችና ማጣሪያዎች** | **የመልስ አማራጮችና ኮዶች** | | | | | | | | | | | | | | | | | | | | | | | | | | | | | | | | | **እለፍ/ፊ** | |
| --- | --- | --- | --- | --- | --- | --- | --- | --- | --- | --- | --- | --- | --- | --- | --- | --- | --- | --- | --- | --- | --- | --- | --- | --- | --- | --- | --- | --- | --- | --- | --- | --- | --- | --- | --- | --- |
| **መለያ**  **እባካችሁ ቃለመጠይቁን ከመጀመራችሁ በፊት የመለያ መረጃዎችን ሙሉ፡፡** | | | | | | | | | | | | | | | | | | | | | | | | | | | | | | | | | | | | |
| A | ይህንን የጤና አገልግሎት መስጫ ቦታ ለዚህ ቃለመጠይቅ ስንት ጊዜ ጎበኘሀው/ሺው? | ለመጀመሪያ ጊዜ 1  ለሁለተኛ ጊዜ 2  ለሶስተኛ ጊዜ 3 | | | | | | | | | | | | | | | | | | | | | | | | | | | | | | | | |  | |
| B | የመረጃ ሰብሳቢ ስም፡ ይህ ያንቺ/ያንተ ስም ነው?  *ከስልኩ ተራ ቁጥር ጋር የተያያዘውን ስም ODK ያሳያል ፡፡*  *ካልሆነ* ፣ እባክህ/ሽ ስምህን/ሽን መዝግብ/ቢ: | አዎ 1  የለም 0 | | | | | | | | | | | | | | | | | | | | | | | | | | | | | | | | |  | |
|  |  |  | | | | | | | | | | | | | | | | | | | | | | | | | | | | | | | | |  |  |
| C | **የአሁኑ ቀን እና ሰዓት በስክሪኑ ላይ ይታያል፡፡**  ይህ ቀን እና ሰዓት ትክከል ነው? | አዎ 1  የለም 0 | | | | | | | | | | | | | | | | | | | | | | | | | | | | | | | | | አዎ ከሆነ ወደ ጥያቄ E እለፉ/ፊ | |
| D | ትክክለኛውን ቀን እና ሰዓት መዝግቡ/ቢ | ቀን | | | ወር | | | | ቀን | | | | | | | | | | | | | | ዓ/ም | | | | | | | | | | | |  | |
|  |  | ጊዜ | | | ሰዓት | | | | ደቂቃ | | | | | | | | | | | | | | ጥዋት/ከሰዓት | | | | | | | | | | | |  |  |
| E | ክልል  **እባካችሁ የጤና አገልግሎት ተቋሙ ያለበትን ክልል ስም ምረጡ ፡፡.** | ትግራይ 1  አፋር 2  አማራ 3  ኦሮሚያ 4  ሶማሊ 5  ቤንሻንጉል ጉሙዝ 6  ደ/ብ/ብ/ሕ/ክ/ 7  ጋምቤላ 8  ሐረሪ 9  አዲስ አበባ 10  ድሬ ዳዋ……………………………………….11 | | | | | | | | | | | | | | | | | | | | | | | | | | | | | | | | |  | |
| E | ዞን  **እባከችሁ የጤና አገልግሎት ተቋሙ የሚገኝበትን ዞን ስም መዝግቡ..** | *በዚሁ መጠይቅ E ላይ የተመረጠውን ክልል መሰረት በማድረግ ODK ተገቢውን የዞን/የወረዳ ዝርዝር ያሳያል፡፡* | | | | | | | | | | | | | | | | | | | | | | | | | | | | | | | | |  | |
| E | ወረዳ  **እባከችሁ የጤና አገልግሎት ተቋሙ የሚገኝበትን ወረዳ ስም መዝግቡ..** | *በዚሁ መጠይቅ*  F *ላይ የተመረጠውን ዞን መሰረት በማድረግ ODK ተገቢውን የወረዳ ዝርዝር ያሳያል፡፡* | | | | | | | | | | | | | | | | | | | | | | | | | | | | | | | | |  | |
| E | የቀበሌ ስም  **እባከችሁ የጤና አገልግሎት ተቋሙ የሚገኝበትን ቀበሌ ስም መለያ መዝግቡ...** | *በዚሁ መጠይቅ*  G *ላይ የተመረጠውን ወረዳ መሰረት በማድረግ ODK ተገቢውን የቀበሌ ስም ዝርዝር ያሳያል፡፡* | | | | | | | | | | | | | | | | | | | | | | | | | | | | | | | | |  | |
| E | የቆጠራ ቦታ ኮድ  **እባከችሁ የጤና አገልግሎት ተቋሙ የሚገኝበትን ወይም የተመደበበትን ቦታ የቆጠራ ቦታ ኮድ/ቁጥር መዝግቡ፡፡**  **.** |  | | | | | | | | | | | | | | | | | | | | | | | | | | | | | | | | |  | |
| F | **የጤና አገልግሎት ተቋሙ መለያ ቁጥር**  **ከዝርዝር መመዝገቢያ ቅፅ ላይ የጤና አገልግሎት ተቋሙ ተራ ቁጥሩን መዝግቡ፡፡ .** |  | | | | | | | | | | | | | | | | | | | | | | | | | | | | | | | | |  | |
| G | **የጤና አገልግሎት ተቋሙ ዓይነት**  **የጤና አገልግሎት ተቋሙ ዓይነት ምረጡ፡፡**  **.** | ሆስፒታል 1  ጤና ጣቢያ 2  የጤና ኬላ 3  የጤና ክሊኒክ 4  ፋርማሲ/መድሀኒት ቤት 5  የመድሀኒት ሱቅ/መሸጫ ቦታ 6  ሌላ 7 | | | | | | | | | | | | | | | | | | | | | | | | | | | | | | | | |  | |
| H | የሚያስተዳድረው ባለስልጣን  የጤና አገልግሎት ተቋሙን የሚያስተዳድረውን ባለስልጣን ምረጥ/ጪ። | መንግስታዊ የሆነ ድርጅት 1  መንግስታዊ ያልሆነ ድርጅት 2  በእምነት/በሀይማኖት ተቋማት ስር ያለ ድርጅት 3  የግል ድርጅት 4  ሌላ 5 | | | | | | | | | | | | | | | | | | | | | | | | | | | | | | | | |  | |
| I | በዕለቱ ቃለመጠይቁን ለማድረግ (መልስ ለመስጠት) ብቁ የሆነ መልስ ሰጭ ተገኝቷል? | አዎ 1  የለም 0 | | | | | | | | | | | | | | | | | | | | | | | | | | | | | | | | | የለም ከሆነ ወደ ጥያቄ S እለፉ/ፊ | |
| **የስምምነት ማረጋገጫ**  **ለታካሚዎች አገልግሎት መስጫ ኃላፊ ሆኖ ብቁ ችሎታ ያለውን (ዋናው አስተዳደር ወይም የቤተሰብ ምጣኔ አገልግሎት ኃላፊ) ሆኖ በአሁኑ ወቅት በጤና ተቋሙ የሚገኝ ሰው ለማግኘት ሞክሩ፡፡**  **በመቀጠል የሚከተለውን የመግቢያ/የስምምነት ማረጋገጫ አንብቡ:** | | | | | | | | | | | | | | | | | | | | | | | | | | | | | | | | | | | | |
| ጤና ይስጥልኝ፣ እኔ ስሜ ______________________________ይባላል፡፡ እኛ አዲስ አበባ ዩኒቨርስቲ እና ጤና ጥበቃ ሚኒስቴርን በመወከል ስለ ጤና አገልግሎት የበለጠ ለማወቅ እንዲያስችል መንግስትንና ማህበረሰቡን ለመርዳት እየሰራን እንገኛለን። አሁን የጥናቱን ዓላማ የሚገልፀውን አንቀፅ/አረፍተነገር አነብሎታለሁ፡፡  የእናንተ ተቋም በዚህ ጥናት እንዲሳተፍ በናሙና ተመርጧል፡፡ አሁን ስለ ቤተሰብ ምጣኔ እና ሌሎች የስነተዋልዶ አገልግሎቶች እና የህሙማን መመዝገቢያን በተመለከተ አንዳንድ ጥያቄዎችን እጠይቆታለን፡፡ በመዝገቡ ላይ የተፃፈውን የማንኛውም ተገልጋይ እና የተቋሙ መረጃ ለጥናቱ አላማ ካልሆነ በስተቀር ለማንም ሰው አይተላለፍም ማለትም በሚስጢር የሚያዝ ነው።  ስለ ጤና ተቋሙ ያለውን መረጃ የጤና ድርጅቶች የአገልግሎት አሰጣጥ ለማሻሻል ዕቅድ ለማዘጋጀት ወይም ወደፊት በአገልግሎት አሰጣጥ ላይ ጥልቅ ምርምር ለማድረግ ሊጠቀሙበት ይችላሉ፡፡ ከጤና ተቋሙ የሚሰበሰበው መረጃ ተመራማሪዎች ትንተና እንዲያረጉበትም ይጠቅማል፡፡ ሆኖም የተቋሙ ስም ለማንም አይሰጥም እንዲሁም የእናንተ ተቋም ተለይቶ እንዳይታወቅ የዚህን ተቋም መረጃ የሚጠቀም ማንኛውም ተመራማሪ መረጃዎቹን ወይም ሪፖረቶቹን የሚያቀርበው በአንድ ላይ በተጠናቀረ መልኩ ነው፡፡  አሁን እንድትረዱን የምንጠይቀው የምንሰበስበው መረጃ ትክክለኛ መሆኑን በማረጋገጥ ነው፡፡ የአንዳንድ ጥያቄዎች መረጃ የበለጠ ያብራራል ወይም ጥሩ መልስ ይሰጥበታል ብለው ያሰቡትን ሰው ሊመሩኝ/ሊጦቁሙኝ ይችላሉ፤ ለዚህ ትብብርዎ አስቀድሜ ምስጋናየ አቀርባለሁ።  በዚህ ጥናት ላይ የሚሳተፉት በፈቃደኝነት ሲሆን ያለመመልስና በማንኛዉም ግዜ ቃለ-መጠይቁን የማቆም መብት አሎት። ሆኖም አሳማኝ ሁኖ እሰካገኙት ሁሉም መልስ ቢመልሱ የበለጠ ጠቃሚ እነደሚሆን አያጠራጥርም።  አሁን ስለ ጥናቱ ሊጠይቁኝ የሚፈልጉት ጥያቄ አለ? | | | | | | | | | | | | | | | | | | | | | | | | | | | | | | | | | | | | |
| J | የጥናት አድራጊው አድራሻ የያዘው ፎርም አንድ ኮፒ ለመልስ ሰጭ በመስጠት የስምምነት አንቀፅ አስረዷቸው፡፡ በመቀጠልም ፣ ‹‹ቃለ መጠይቁን መጀመር እችላለሁ?›› ብላችሁ ጠይቁ፡፡ | አዎ 1  የለም 0 | | | | | | | | | | | | | | | | | | | | | | | | | | | | | | | | | የለም ከመለሱ ወደ ጥያቄ S እለፉ/ፊ | |
| K | የመረጃ ሰብሳቢ ስም  **የስምምነቱን ሂደት በትክክል መካሄዱን ለመመስከር ስምህን/ሽን ፃፍ/ፊ።** |  | | | | | | | | | | | | | | | | | | | | | | | | | | | | | | | | |  | |
| L | የጤና ተቋሙ ስም  **የጤና ተቋሙን ስም ፃፉ** |  | | | | | | | | | | | | | | | | | | | | | | | | | | | | | | | | |  | |
| M | በዚህ ጤና ተቋም ውስጥ የእርስዎ የስራ ድርሻ/የስራ መደብ ምንድን ነው?  **የመልስ ሰጭውን የተለያየ የስራ ሃላፊነት/ ድርሻ ካለው ከፍተኛ ሃላፊነት ያለበትን ምረጡ፡፡** | ባለቤት 1  ወኪል /ማናጄር/ዋና ኃላፊ 2  የመ/ቤቱ ሰራተኛ 3 | | | | | | | | | | | | | | | | | | | | | | | | | | | | | | | | |  | |
| **ተ.ቁ** | **ጥያቄዎች እና ማጣሪያዎች** | **የመለስ አማራጮች ኮድ** | | | | | | | | | | | | | | | | | | | | | | | | | | | | | | | | | **ይለፍ** | |
| **ክፍል 1 – ስለ አገልግሎት መስጫ ተቋሙ አጠቃላይ መረጃ**  **አሁን በዚህ የጤና ተቋም ውስጥ ስለሚሰጠው አገልግሎት አንዳንድ ጥያቄዎችን ልጠይቆት እወዳለሁ፡፡** | | | | | | | | | | | | | | | | | | | | | | | | | | | | | | | | | | | | |
| 1 | ይህ ተቋም የጤና አገልግሎት መስጠት የጀመረው መቼ ነው?  **አላውቅም ከሆነ 2020 በተሰጠው ክፍት ቦታሙሉ** | ዓመተ ምህረት | | | | |  | | | | | | | | | | | | | | | | | | | | | | | | | | | |  | |
| 2 | ተቋሙ በመደበኛነት በየሳምንቱ ስንት ቀን ይከፈታል/ይሰራል?  **ቁጥሩ ከ 1 እስከ 7 መሆን ይኖርበታል፡፡**  **አላውቅም ከሆነ -88 በተሰጠው ክፍት ቦታ አስገቡ፡፡ .** | የቀኖች ብዛት | | | | |  | | | | | | | | | | | | | | | | | | | | | | | | | | | |  | |
| 3 | **አሁን ደግሞ በዚህ ተቋም ውስጥ ስለሚሰሩ ሰራተኞች አንዳንድ ጥያቄዎችን እጠይቆታለሁ፡፡**  በአሁኑ ወቅት በዚህ ተቋም ውስጥ በሚከተሉት ሞያ የሰለጠኑ ምን ያህል ሰራተኞች በጤና ተቋሙ ተመድበው እንደሚሰሩ ይንገሩኝ፡፡  በዛሬው ዕለት የተገኙ ሰራተኞችን ድምር ይንገሩኝ፡፡  እኛ የምንፈልገው ሰራተኛው የሰለጠነበትን ወይም የተማረውን ከፍተኛ የሙያ ደረጃ እንጂ በአሁኑ ወቅት ተመድቦ የሚሰራበትን ክፍል ወይም የወሰደውን ልዩ ስልጠና አይደለም፡፡  **አላውቅም ከሆነ 88 በተሰጠው ክፍት ቦታ ሙሉ እና -77 አይመለከተውም፡፡ ዜሮ መልስ ሊሆን ይችላል፡፡** | ዶክተር  ጤና መኮንን  ነርስ/አዋላጅ ነርስ  የፋርማሲባለሙያ ሰራተኛ  ሌሎች የሕክምና ሰራተኞች  የጤና ኤክሰቴንሽን ሠራተኞች …………… | | | | | | | | | | | | Actual number  __  __  __  __  __  __ | | | | | | | | | | | | Present today  __  __  __  __  __  __ | | | | | | | | |  | |
|  | K**ተመልከቱ:** የተቋሙ ዓይነት ምን ለሆን ይችላል? | ሆስፒታል 1  የጤና ጣቢያ 2  የጤና ኬላ 3  የጤና ክሊኒክ 4  ፋርማሲ/መድሀኒት ቤት 5  የመድሀኒት መደብር 6  ሌላ 7 | | | | | | | | | | | | | | | | | | | | | | | | | | | | | | | | | 5, 6 ወይም 7 ከሆነ ወደ ጥያቄ 5 | |
| 4 | በተቋም ውስጥ በማንኛውም ሰዓት/ሁልጊዜ የሚገኝ (በቀን 24 ሰዓት የሚሰራ) ወይም በስልክ ሁልጊዜ (በቀን 24 ሰዓት) በጥሪ ለድንገተኛ የሚሰራ የጤና ባለሙያ አለ? | አዎ, 24-ሰዓት ሰራተኛ አለ 1  የለም, የ24-ሰዓት የሚሰራ የለም 0 | | | | | | | | | | | | | | | | | | | | | | | | | | | | | | | | |  | |
| 5 | ይህ ተቋም አገልግሎት የሚሰጠው ህዝብ ፣ማለትም ተቋሙ ለማገልገል ያቀደው ህዝብ ብዛት ወይም በዚህ ተቋም የሚገለገሉ በጠቅላላ በአካባቢው የሚኖሩ ህዝቦች ብዛት ስንት እንደሆነ ያውቃሉ? | ተቋሙ አገልግሎት የሚሰጥበት የተወሰነ ቦታ/ህዝብ/ የለውም 1  አዎ፣ የሚገለገለው ህዝብ ብዛትና / ይታወቃል 2  የሚገለገለው ህዝብ ብዛትና /ቦታ ግምት አላውቅም 3 | | | | | | | | | | | | | | | | | | | | | | | | | | | | | | | | | የለም ወይም አላውቅም ከሆነ ወደ ጥያቄ 7 እለፉ/ፊ | |
| 6 | የሚገለገለው ህዝብ ብዛት ምን ያህል ነው ?  **በጠቅላላ በአካባቢው የሚኖሩ እና ተቋም አገልግሎት የሚሰጠው ህዝብ ብዛት መዝግብ/ቢ፡፡.** | የሰዎች ብዛት | | | | |  | | | | | | | | | | | | | | | | | | | | | | | | | | | |  | |
| 7 | የጤና ተቋሙ ስንት የታካሚዎች መኝታ አልጋ አለው?  **ዜሮ መልስ ሊሆን ይችላል፡፡ አላውቅም ብለው ከመለሱ -88 መዝግብ/ቢ።** | የአልጋ ብዛት | | | | |  | | | | | | | | | | | | | | | | | | | | | | | | | | | |  | |
| 8 | የተቋሙ ባለቤት ወይም ከዚህ ተቋም ውጭ ያለ ተቆጣጣሪ ለመጨረሻ ጊዜ ይህንን ተቋም ለመጎብኘት የመጣው መቼ ነበር? | በጭራሽ ከተቋሙ ውጭ የመጣ ተቆጣጣሪ የለም 0  ባለፉት 6 ወራት ውስጥ 1  ከ6 ወር በፊት 2  አላውቅም -88 | | | | | | | | | | | | | | | | | | | | | | | | | | | | | | | | |  | |
| 9 | በዛሬው ዕለት ይህ ተቋም የኤሌትሪክ ኃይል አለው?  ***በቃለ-መጠይቁ ግዜ መብራት ከልነበረ ወይም ለተከታታይ 2 ሰዓታት ጠፍቶ ከነበረ የለም የሚለዉን ኮድ 0 አክብብ/ቢ*** | አዎ 1  የለም 0 | | | | | | | | | | | | | | | | | | | | | | | | | | | | | | | | |  | |
| 10 | በዛሬው ዕለት ይህ ተቋም ውሃ አለው?  ***በቃለ-መጠይቁ ግዜ ወሃ ከልነበረ ወይም ለተከታታይ 2 ሰዓታት ጠፍቶ ከነበረ የለም የሚለዉን ኮድ 0 አክብብ/ቢ*** | አዎ 1  የለም 0 | | | | | | | | | | | | | | | | | | | | | | | | | | | | | | | | |  | |
|  | K **ተመልከት/ቺ:** የተቋሙ ዓይነት? | ሆስፒታል 1  የጤና ጣቢያ 2  የጤና ኬላ 3  የጤና ክሊኒክ 4  ፋርማሲ 5  የመድሀኒት ሱቅ/መሸጫ ቦታ 6  ሌላ 7 | | | | | | | | | | | | | | | | | | | | | | | | | | | | | | | | | ኮድ 5, 6 ወይም 7 ከተከበበ ወደ ጥያቄ 13 እለፉ/ፊ | |
| 11 | በአሁኑ ወቅት **የተቋሙ ሰራተኞች** የሚጠቀሙበት ምን ያህል የእጅ መታጠቢያ ቦታ አለ?  **አላውቅም ብለው ከመለሱ -88 መዝግብ/ቢ።** | የእጅ መታጠቢያ ቦታዎች ብዛት | | | | | | | |  | | | | | | | | | | | | | | | | | | | | | | | | | መልሱ ዜሮ ከሆነ ወደ ጥያቄ 13 እለፉ/ፊ | |
| 12 | በአቅራቢያው የሚገኘውን የእጅ መታጠቢያ ቦታ እንዲያሳዪአችሁ ጠይቁ፡፡ የሚከተሉት በእጅ መታጠቢያው ቦታ መኖሩን ተመልከቱ:  ሳሙና አለ  የተጠራቀመ ውሃ አለ  የቧንቧ ውሃ አለ  የእጅ መታጠቢያ ቦታው ከመፀዳጃው ቤት አጠገብ ነው  ከላይ የተጠቀሱት አንዱም የለም  ምንም የእጅ መታጠቢያ ቦታ አልታየም  **የተጠቀሱትን በሙሉ ምረጡ፡፡** |  | | | | | አዎ  1  1  1  1  -88  1 | | | | | | | | | | | | | | የለም  0  0  0  0  0 | | | | | | | | | | | | | |  | |
| 13 | ይህ ተቋም በአሁኑ ወቅት **የሚሰራ** ኮምፒዩተር አለው?  ***መኖር አለመኖሩን ማየት አያስፈልግም*** | አዎ 1  የለም 0 | | | | | | | | | | | | | | | | | | | | | | | | | | | | | | | | |  | |
|  | K **ተመልከት/ቺ:** የተቋሙ ዓይነት? | ሆስፒታል 1  የጤና ጣቢያ 2  የጤና ኬላ 3  የጤና ክሊኒክ 4  ፋርማሲ/መድሀኒት ቤት 5  የመድሀኒት መደብር 6  ሌላ 7 | | | | | | | | | | | | | | | | | | | | | | | | | | | | | | | | | ኮድ 5, 6 ወይም 7  ከተከበበ ወደ ጥያቄ 15 እለፉ/ፊ | |
| 14 | ይህ ተቋም ጥቅም ላይ የዋሉ ስለታማ/ሹል ነገሮች ወይም ስለታማ ነገሮችን ማጠራቀሚያ ካርቶኖችን የሚያስወግደው እንዴት ነው? | ጥቅም ላይ የዋሉ/ቆሻሻ ስለታማ ነገሮች ኖሮት አያውቅም 0  ቆሻሻ ማቃጠያ (Incinerator) ውስጥ ማቃጠል 1  ክፍት በሆነ ቦታ ማቃጠል 2  ሳይቃጠል ቆሻሻ መጣያ ውስጥ መጣል 3  የማይታይበት ቦታ መጣል/ማስወገድ 4  ሌላ 5 | | | | | | | | | | | | | | | | | | | | | | | | | | | | | | | | |  | |
| **ክፍል 2 – የቤተሰብ ምጣኔ አገልግሎት**  **አሁን ደግሞ በዚህ የጤና ተቋም ውስጥ ስለሚሰጠው የቤተሰብ ምጣኔ አገልግሎት አንዳንድ ጥያቄዎችን ልጠይቆት እወዳለሁ፡፡** | | | | | | | | | | | | | | | | | | | | | | | | | | | | | | | | | | | | |
| 15 | በዚህ ተቋም አብዛኛውን ጊዜ የቤተሰብ እቅድ አገልግሎት/እርግዝናን ሊያስቀሩ ወይም ሊያዘገዩ የሚችሉ ዘዴዎች ታቀርባላችሁ /ትሰጣላችሁ? | | አዎ 1  የለም 0 | | | | | | | | | | | | | | | | | | | | | | | | | | | | | | | | | የለም ከሆነ ወደ ጥያቄ 19 እለፉ/ፊ |
| 16 | ይህ ተቋም የቤተሰብ እቅድ አገልግሎት ወይም እርግዝናን ሊያስቀሩ ወይም ሊያዘገዩ የሚችሉ ዘዴዎች መስጠት የጀመረው በየትኛው ዓመተምህረት ነው?  **አላውቅም ብለው ከመለሱ 2020 አስገባ/ቢ፡፡** | | ዓመተ ምህረት | | | | | | |  | | | | | | | | | | | | | | | | | | | | | | | | | |  |
| 17 | በተቋሙ የቤተሰብ እቅድ አገልግሎት ወይም እርግዝናን ሊያስቀሩ ወይም ሊያዘገዩ የሚችሉ ዘዴዎች በሳምንት ውስጥ ለምን ያህል ቀናት ይሰጣል/ይሸጣል?  **የቀኑን ብዛት ለማስላት የሳምንቱን 7 ቀናት ተጠቀሙ፡፡ ከ1 እስከ 7 ያሉትን ቁጥሮች ብቻ አስገቡ፡፡ አላውቅም ብለው ከመለሱ «-88» መዝግብ/ቢ፡፡.** | | የቀኖች ብዛት | | | | | | |  | | | | | | | | | | | | | | | | | | | | | | | | | |  |
| 18 | በዛሬው ዕለት እዚህ ተቋም ውስጥ የቤተሰብ እቅድ አገልግሎት (እርግዝናን ሊያስቀሩ ወይም ሊያዘገዩ የሚችሉ ዘዴዎች) እየተሰጠ ነው? | | አዎ 1  የለም 0 | | | | | | | | | | | | | | | | | | | | | | | | | | | | | | | | |  |
|  | K **ተመልከት/ቺ:** የተቋሙ ዓይነት? | | ሆስፒታል 1  የጤና ጣቢያ 2  የጤና ክሊኒክ 3  የጤና ክሊኒክ 4  ፋርማሲ/መድሀኒት ቤት 5  የመድሀኒት መደብር 6  ሌላ 7 | | | | | | | | | | | | | | | | | | | | | | | | | | | | | | | | | ኮድ 5, 6 ወይም 7 ከተከበበ ወደ ጥያቄ 23 እለፉ/ፊ |
| 19 | ይህ ተቋም ለማህበረሰብ አቀፍ የጤና በጎ ፈቃደኛ ሰራተኞች የቤተሰብ እቅድ ክትትል፣ ድጋፍ ወይም የእርግዝና መከላከያ ዘዴዎች አቅርቦት ይሰጣል?  *በጥያቄ 3 በደመወዝ ተከፋይ ባለሙያነት የተመዘገቡ የጤና ኤክስቴንሽን ባለሙያ ካሉ በጎ ፈቃደኛ ሰራተኞች ተብለው ሊቆጠሩ አይገባም* | | አዎ 1  የለም 0 | | | | | | | | | | | | | | | | | | | | | | | | | | | | | | | | | የለም ከሆነ ወደ ጥያቄ 22 እለፉ/ፊ |
| 20 | በዚህ ተቋም ድጋፍ ያገኙት ወይም የተረዱት የማህበረሰብ አቀፍ የጤና በጎ ፈቃደኛ ምን ያህል ናቸው?  *የቤተሰብ እቅድ ክትትል፣ ድጋፍ ወይም የእርግዝና መከላከያ ዘዴዎች አቅርቦት የሚሰጡ የበጎ ፈቃደኛ ሰራተኞችን ብቻ መዝግቡ.*  *በጥያቄ 3 በደመወዝ ተከፋይ ባለሙያነት የተመዘገቡ የጤና ኤክስቴንሽን ባለሙያ ካሉ በጎ ፈቃደኛ ሰራተኞች ተብለው ሊቆጠሩ አይገባም.*  ***አላውቅም ብለው ከመለሱ -88፣ መዝግብ/ቢ።*** | | የጤና በጎ ፈቃደኛ | | | | | | | |  | | | | | | | | | | | | | | | | | | | | | | | | |  |
| 21 | የማህበረሰብ አቀፍ የጤና በጎ ፈቃደኛ ሰራተኞች የሚከተሉትን እርግዝናን ሊያስቀሩ ወይም ሊያዘገዩ የሚችሉ ዘዴዎች ያቀርባሉ:  ኮንዶሞች  የወሊድ መቆጣጠሪያ ኪኒን  የወሊድ መቆጣጠሪያ መርፌ | |  | | | | | | | | | | | | | አዎ  1  1  1 | | | | | | | | | | | | | የለም  0  0  0 | | | | | | |  |
| 22 | ባለፉት 12 ወራት ውስጥ ተንቀሳቃሽ የጤና ቡድን ተጨማሪ የቤተሰብ እቅድ አገልግሎት ለመስጠት ምን ያህል ጊዜ በእናንተ ተቋም ውስጥ መጥተዋል/ጉብኝት አድርጓል? | | ጉብኝት የተደረገበት ጊዜ ብዛት: | | | | | | | | | | | | |  | | | | | | | | | | | | | | | | | | | |  |
|  | **ጥ.15 ተመልከት/ች:** የቤተሰብ እቅድ አገልግሎት/እርግዝናን ሊያስቀሩ ወይም ሊያዘገዩ የሚችሉ ዘዴዎች ይሰጣሉ/ያቀርባል? | | አዎ 1  የለም 0 | | | | | | | | | | | | | | | | | | | | | | | | | | | | | | | | | የለም ከሆነ ወደ ጥያቄ 25 እለፉ/ፊ |
| 23 | ይህ ተቋም በመደበኛነት ለሚሰጠው አገልግሎት ወይም ለማንኛውም ከቤተሰብ እቅድ ጋር ተያያዥነት ያላቸው ላለው አገልግሎት ክፍያ አለው?  **ይህም ማንኛውንም የህክምና አገልግሎት ክፍያ እና የደንበኞች የመመዝገቢያ/ የካርድ ክፍያንም ያጠቃልላል፡፡** | | አዎ 1  የለም 0 | | | | | | | | | | | | | | | | | | | | | | | | | | | | | | | | | የለም ከሆነ ወደ ጥያቄ 25 እለፉ/ፊ |
| 24 | ለህክምና አገልግሎት ሊከፈል የሚገባው ህጋዊ የክፍያ ገንዘብ መጠን ደንበኞች ሊያዩት በሚችሉበት ቦታ ላይ ተሰጥፏል?  **አዎ ከሆነ፣ የተለጠፈው የክፍያ ዝርዝር ዋጋ መታየት ይኖርበታል፡፡** | | አዎ፣የሁሉም ክፍያዎች ዝርዘር ተለጥፏል 1  አዎ፣ የተወሰኑ ክፍያዎች ዝርዘር ተለጥፏል 2  ምንም የተለጠፈ የክፍያ ዝርዘር የለም 0 | | | | | | | | | | | | | | | | | | | | | | | | | | | | | | | | |  |
| 25 | የደንበኞችን አስተያየት ለማወቅ በሚከተሉት መንገድ መረጃ ትሰበስባላችሁ?  በሃሳብ መስጫ ሳጥን  በደንበኞች አስተያየት መሰብሰቢያ ቅፅ/ፎርም  በደንበኞች ቃለ መጠይቅ ማድረጊያ ቅፅ/ፎርም  ከማህበረሰብ መሪዎች ጋር በይፋ ስብሰባ በማድረግ  መደበኛ ባልሆነ መልኩ ከደንበኞች ወይም ከማህበረሰቡ ጋር ውይይት ማድረግ  ደንበኞች በቀጥታ ለሰራተኞች ከሚሰጡት አስተያየት/ሃሳብ  ሌላ  አላውቅም  ከላይ ከተገለፁት ሁሉም አይደሉም  **ሁሉም የተጠቀሱትን ዘዴዎች ምረጥ/ጪ፡፡** | |  | | | | | | | | | | | | | Yes  1  1  1  1  1  1  1  1  -88 | | | | | | | | | | | | | No  0  0  0  0  0  0  0  0 | | | | | | | “ ከላይ ከተገለፁት ሁሉም አይደሉም “ የሚለው መልስ ከሆነ ወደ ጥያቄ 29 እለፉ/ፊ |
| 26 | ደንበኞችን የሰጡት አስተያየት ተጠናቅሮ ረፖርት እንዲደረግ የምትከተሉት አካሄድ አለ? | | አዎ 1  የለም 0 | | | | | | | | | | | | | | | | | | | | | | | | | | | | | | | | | የለም ከሆነ ወደ 28 እለፉ/ፊ |
| 27 | መረጃው የተጠናቀረበትን የሪፖርት ቅፅ/ፎርም ወይም የውይይቱ ውጤት ሪፖርት የተደረገበትን ዶክሜንት እንዲያሳዩአችሁ ጠይቁ። | | ሪፖርቱ ታይቷል 1  ሪፖርቱ አልታየም 2 | | | | | | | | | | | | | | | | | | | | | | | | | | | | | | | | |  |
| 28 | ባለፉት 6 ወራት ውስጥ የደንበኞች አስተያየት መሰረት በማድረግ በፕሮግራሙ ላይ የተደረገ ለውጥ አለ?  **አዎ ከሆነ፣ ለውጡ ከተዘረዘሩት ርዕሶች ጋር የሚመሳሰል መሆኑን አረጋግጠህ/ሽ ሙላ/ዪ።** | | የለም 0  አዎ፣የሚሰጡ የአገልግሎት አይነቶችና የሚሰጥበት ጊዜ ወይም የአገልግሎት አሰጣጥ ላይ ለውጥ አለ 1  አዎ፣ ለደንበኞች ምቹ እንዲሆን ተደርጓል 2  ሌላ 3  አላውቅም -88 | | | | | | | | | | | | | | | | | | | | | | | | | | | | | | | | |  |
| 29 | ባለፉት 6 ወራት ውስጥ በቤተሰብ እቅድ ላይ የተሰጡ የአገልግሎቶች መረጃዎችን (አጠቃላይ ቆጠራን) በተመለከተ ከሰራተኞች ጋር ውይይት/ሰብሰባ ተደርጎ ነበር? | | አዎ 1  የለም 0 | | | | | | | | | | | | | | | | | | | | | | | | | | | | | | | | |  |
| 30 | የአገልግሎት አሰጣጥ መረጃን በሚገባ ለማየት፣ ለመከታተልና ለመገምገም ከሚከተሉት ዝርዝሮች ማንኛውም ዓይነት ዘዴ ተጠቅመዋል?  በግድግዳ ላይ የሚለጠፍ ቻርት/ግራፍ  የፅሁፍ ሪፖርት/ቃለጉባኤ  ሌላ  ምንም አልታየም  **አገልግሎትን በተመለከተ ያለውን መረጃው መገምገሙን እና መጠናቀሩን ማንኛውም ሪፖርት ተጽፎ እንደሆነ ወይም ግራፍ፣ቻርት ተዘጋጅቶ በግድግዳ ላይ ተለጥፎ እንደሆነ እንዲያሳዩችሁ ጠይቅ/ቂ።**  **ካያችሁኃቸው ሰነዶች /ዶክሜንቶች ውስጥ ከላይ ከተዘረዘረው ጋር የሚመሳሰለውን ምረጥ/ጪ።** | |  | | | | | | | | | | | | | | አዎ  1  1  1  1 | | | | | | | | | | | | | የለም  0  0  0  0 | | | | | |  |
|  | **ጥ.15 ተመልከት:** የቤተሰብ ምጣኔ አገልግሎት/እርግዝናን ሊያስቀሩ ወይም ሊያዘገዩ የሚችሉ ዘዴዎች ያቀርባል? | | አዎ 1  የለም 0 | | | | | | | | | | | | | | | | | | | | | | | | | | | | | | | | | የለም ከሆነ ወደ ጥያቄ ቁ 40 እለፉ/ፊ |
| 31 | ከሚከተሉት ውስጥ የትኞቹ እርግዝናን ሊያስቀሩ ወይም ሊያዘገዩ የሚችሉ ዘዴዎች ናቸው እንዲወሰዱ ምክር የተሰጠባቸው፣ የትኞቹ ለተጠቃሚ ተሰጥተዋል፣ ወይም ሌላ ቦታ እንዲጠቀሙ ሪፈር/ማዘዣ/ ወረቀት ተሰጥቷል?  ከዚህ በታች ለተዘረዘሩት ዘዴዎች ክፍያ ታስከፍላላችሁ?  ሴቶችን በኦፕራሲዮን ማምከን  ወንዶችን በኦፕራሲዮን ማምከን  በማሕፀን የሚቀበር ሉፕ  ለሚያጠቡ እናቶች የወሊድ መከላከያ ክኒን  የወሊድ መከላከያ መርፌ  በክንድ የሚቀበር ወመ  የወሊድ መከላከያ ክኒን  የወንድ ኮንዶም  የሴት ኮንዶም  ድንገተኛ የወሊድ መቆጣጠሪያ  ስታንዳርድ የቀን አቆጣጠር ዘዴ በዶቃ/ጨሌ መሳይ……...  ህፃኑን የጡት ወተት ብቻ መመገብ  ቀን አቆጣጠር ዘዴ  የወንድ የዘር ፍሬ ውጭ ማፍሰስ  ሌላ ባህላዊ ዘዴ  ምክር፡ ስለ ዘዴው የምክር አገልግሎት፣  አቅ: አቅርቦት ለተጠቃሚ  ማዘዣ: ወደ ሌላ ተቋምእንዲሄድ  ክፍያ፡ ክፍያ ተደርጓል  **ድምፃችሁን ከፍ አድርጋችሁ ሁሉንም አማራጮች ማንበብ ይኖርብሃል/ሻል።** | | ምክር  አዎ | ምክር  የለም | | አቅ  አዎ | | አቅ  የለም | | | | ማዘዣ  አዎ | | | | | | | | | | ማዘዣ  የለም | | | | | | | | | | | ክፍያ | | | ካላስከፈሉ ወደ ጥያቄ 33 እለፉ/ፊ |
|  |  |  | 1 | 0 | | 1 | | 0 | | | | 1 | | | | | | | | | | 0 | | | | | | | | | | | 1 | | |  |
|  |  |  | 1 | 0 | | 1 | | 0 | | | | 1 | | | | | | | | | | 0 | | | | | | | | | | | 1 | | |  |
|  |  |  | 1 | 0 | | 1 | | 0 | | | | 1 | | | | | | | | | | 0 | | | | | | | | | | | 1 | | |  |
|  |  |  | 1 | 0 | | 1 | | 0 | | | | 1 | | | | | | | | | | 0 | | | | | | | | | | | 1 | | |  |
|  |  |  | 1 | 0 | | 1 | | 0 | | | | 1 | | | | | | | | | | 0 | | | | | | | | | | | 1 | | |  |
|  |  |  | 1 | 0 | | 1 | | 0 | | | | 1 | | | | | | | | | | 0 | | | | | | | | | | | 1 | | |  |
|  |  |  | 1 | 0 | | 1 | | 0 | | | | 1 | | | | | | | | | | 0 | | | | | | | | | | | 1 | | |  |
|  |  |  | 1 | 0 | | 1 | | 0 | | | | 1 | | | | | | | | | | 0 | | | | | | | | | | | 1 | | |  |
|  |  |  | 1 | 0 | | 1 | | 0 | | | | 1 | | | | | | | | | | 0 | | | | | | | | | | | 1 | | |  |
|  |  |  | 1 | 0 | | 1 | | 0 | | | | 1 | | | | | | | | | | 0 | | | | | | | | | | | 1 | | |  |
|  |  |  | 1 | 0 | | 1 | | 0 | | | | 1 | | | | | | | | | | 0 | | | | | | | | | | | 1 | | |  |
|  |  |  | 1 | 0 | |  | |  | | | |  | | | | | | | | | |  | | | | | | | | | | |  | | |  |
|  |  |  | 1 | 0 | |  | |  | | | |  | | | | | | | | | |  | | | | | | | | | | |  | | |  |
|  |  |  | 1 | 0 | |  | |  | | | |  | | | | | | | | | |  | | | | | | | | | | |  | | |  |
|  |  |  | 1 | 0 | |  | |  | | | |  | | | | | | | | | |  | | | | | | | | | | |  | | |  |
|  |  |  |  |  | |  | |  | | | |  | | | | | | | | | |  | | | | | | | | | | |  | | |  |
| 32 | ለአንድ ጊዜ ሲመጡ ለምትሰጧቸው ለእያንዳንዱ የእርግዝና መከላከያ ዘዴ ምን ያህል ታስከፍላላችሁ?  ሴቶችን በኦፕራሲዮን ማምከን  ወንዶችን በኦፕራሲዮን ማምከን  በማሕፀን የሚቀበር ሉፕ  ለሚያጠቡ እናቶች የወሊድ መከላከያ ክኒን  የወሊድ መከላከያ መርፌ  በክንድ የሚቀበር ወመ  የወሊድ መከላከያ ክኒን  የወንድ ኮንዶም  የሴት ኮንዶም  ድንገተኛ የወሊድ መቆጣጠሪያ  ስታንዳርድ የቀን አቆጣጠር ዘዴ በዶቃ/ጨሌ መሳይ……...  **የገንዘቡን መጠን በብርና ሳንቲም አስገቡ/ጻፉ፡፡.**  *ተቋሙ የሚያስከፍላቸውን ዘዴዎቸ ብቻ ODK ከጥያቄ ቁጥር 31 ያሳያል፡፡* | | የክፍያ መጠን በዝርዝር ለእያንዳንዱ | | | | | | | | | | | | | | | | | | | _________  _________  _________  _________  _________  _________  _________  _________  _________  _________  _________ | | | | | | | | | | | | | |  |
|  | K **ተመልከት/ቺ:** የተቋሙ ዓይነት? | | ሆስፒታል 1  የጤና ጣቢያ 2  የጤና ኬላ 3  የጤና ክሊኒክ 4  ፋርማሲ/መድሀኒት ቤት 5  የመድሀኒት መደብር 6  ሌላ 7 | | | | | | | | | | | | | | | | | | | | | | | | | | | | | | | | | ኮድ 5, 6 or 7 ከተከበበ ወደ ጥያቄ 39b እለፉ/ፊ |
|  | **ጥ.31 ተመልከት/ቺ:** በክንድ የሚቀበር እርግዝና መከላከያተሰጥቷል? | | አዎ 1  የለም 0 | | | | | | | | | | | | | | | | | | | | | | | | | | | | | | | | | የለም ከሆነ ወደ ጥያቄ 35 እለፉ/ፊ |
| 33 | ይህ የጤና ተቋም የቤተሰብ እቅድ አገልግሎት በሚሰጥበት ወቅት በክንድ የሚቀበር እርግዝና መከላከያእንዴት **መቀበር/መግባት** እንዳለበት የሰለጠነ የጤና ባለሙያ አለው? | | አዎ 1  የለም 0 | | | | | | | | | | | | | | | | | | | | | | | | | | | | | | | | |  |
| 34 | ይህ የጤና ተቋም የቤተሰብ እቅድ አገልግሎት በሚሰጥበት ወቅት በክንድ የሚቀበር እርግዝና መከላከያእንዴት **መውጣት/ መወገድ** እንዳለበት የሰለጠነ የጤና ባለሙያ አለው? | | አዎ 1  የለም 0 | | | | | | | | | | | | | | | | | | | | | | | | | | | | | | | | |  |
|  | **ጥ.31ተመልከት/ቺ:** በማሕፀን የሚቀበር ሉፕ ተሰጥቷል? | | አዎ 1  የለም 0 | | | | | | | | | | | | | | | | | | | | | | | | | | | | | | | | | የለም ከሆነ ወደ ጥያቄ 37 እለፉ/ፊ |
| 35 | ይህ የጤና ተቋም የቤተሰብ እቅድ አገልግሎት በሚሰጥበት ወቅት በማሕፀን የሚቀበር ሉፕ እንዴት በማህፀናቸው እንዴት መግባት እንዳለበት የሰለጠነ የጤና ባለሙያ አለው? | | አዎ 1  የለም 0 | | | | | | | | | | | | | | | | | | | | | | | | | | | | | | | | |  |
| 36 | ይህ የጤና ተቋም የቤተሰብ እቅድ አገልግሎት በሚሰጥበት ወቅት በማሕፀን የሚቀበር ሉፕ እንዴት ከማህፀናቸው እንዴት መውጣት/ መወገድ እንዳለበት የሰለጠነ የጤና ባለሙያ አለው? | | አዎ 1  የለም 0 | | | | | | | | | | | | | | | | | | | | | | | | | | | | | | | | |  |
|  | **ጥ.31ተመልከት/ቺ:** በክንድ የሚቀበር እርግዝና መከላከያተሰጥቷል? | | አዎ 1  የለም 0 | | | | | | | | | | | | | | | | | | | | | | | | | | | | | | | | | የለም ከሆነ ወደ ጥያቄ 38 እለፉ/ፊ |
| 37 | ይህ የጤና ተቋም በክንድ የሚቀበር እርግዝና መከላከያ **ለመቅበር** ወይም ለማስገባት እና/ወይም ለማውጣት/ለማስወገድ የሚገለገልበት ከዚህ በታች የተዘረዘሩት ቁሳቁሶች አሉት፡  Clean/ንፁህ የእጅ ጓንቶች  ተዋሀስያን ማስወገጃ/Antiseptic  ፋሻ ንፅህናውን የጠበቀ /Gauze/ወይም ጥጥ  ማደንዘዣ/Local Anesthetic  የታሸገ በክንድ የሚቀበር እርግዝና መከላከያ ፓኬት  ምላጭ/Surgical blade  ***እያንዳንዱ የዕቃ አቅርቦት ቃለ-መጠይቁ በተደረገበት ቀን መኖር አለበት። ቁሳቁሶቹን ማየት አስፈላጊ አይደለም፡፡.*** | |  | | | | | | | | | | | | Yes  1  1  1  1  1  1 | | | | | | | | | | | | | No  0  0  0  0  0  0 | | | | | | | |  |
|  | **ጥ. 31ተመልከቱ:** በማሕፀን የሚቀበር ሉፕ ተሰጥቷል? | | አዎ 1  የለም 0 | | | | | | | | | | | | | | | | | | | | | | | | | | | | | | | | | የለም ከሆነ ወደ ጥያቄ 39 እለፉ/ፊ |
| 38 | ይህ የጤና ተቋም በማሕፀን የሚቀበር ሉፕ በማህፀን ውስጥ ለማስቀመጥ ወይም ለማስገባት እና/ወይም ከማህፀናቸው ውስጥ ለማውጣት ከዚህ በታች የተዘረዘሩት አስፈላጊ የሆኑ አቅርቦቶች አሉት፡  -ስፖንጅ ለመያዝ የሚጠቀሙበት መቀስ /Sponge-holding forceps/  -ማህፀን ለመክፈቻ/ሰፋ አድርጎ ለማሳየት የሚያገለግል መሳሪያ( ትልቅ እና መካከለኛ) Speculums (large and medium  -የማህፀን በር መቆንጠጫ Tenaculum  -በቀዶ ጥገና ወቅት አጥብቆ ለመያዝ የሚጠቅም ለተለያዩ ነገሮች መያዣ የሚሆን መሳሪያ (Clamp)  ***እያንዳንዱ የዕቃ አቅርቦት ቃለ-መጠይቁ በተደረገበት ቀን መኖር አለበት። ቁሳቁሶቹን ማየት አስፈላጊ አይደለም*፡፡ ቁሳቁሶቹን ማየት አስፈላጊ አይደለም፡፡.** | |  | | | | | | | | | | | | | አዎ  1  1  1  1 | | | | | | | | | | | | የለም  0  0  0  0 | | | | | | | |  |
|  | **ጥ.** K**ን ተመልከት/ቺ:** የተቋሙ ዓይነት? | | ሆስፒታል 1  የጤና ጣቢያ 2  የጤና ኬላ 3  የጤና ክሊኒክ 4  ፋርማሲ/መድሀኒት ቤት 5  የመድሀኒት መደብር ቦታ 6  ሌላ 7 | | | | | | | | | | | | | | | | | | | | | | | | | | | | | | | | | ኮድ: 1-4 ወደ ጥያቄ 39a እለፉ/ፊ  ኮድ: 5, 6 ወይም 7 ከተከበበ ወደ ጥያቄ 39b እለፉ/ፊ |
| 39a | **ከቤተሰብ እቅድ አገልግሎት መዝገብ ላይ መዝግቡ:**  **(1)** ባለፈው ወር ውስጥ የቤተሰብ ምጣኔ አገልግሎት ማለትም ለእያንዳንዱ ዘዴ የተደረገ ጠቅላላ ጉብኝት (አዲስና ነባር) ብዛት በተሰጠው ቦታ አስገቡ (ለእያንዳንዱ ዘዴ በተናጠል ሙሉ)  **(2)** ባለፈው ወር ውስጥ የቤተሰብ ምጣኔ ማለትም ለእያንዳንዱ ዘዴ የወሰዱ/የተቀበሉን አዲስ ደንበኞች ብዛት አስገቡ  (ለእያንዳንዱ ዘዴ በተናጠል ሙሉ) | | ሴቶችን በኦፕራሲዮን ማምከን  ወንዶችን በኦፕራሲዮን ማምከን  በማሕፀን የሚቀበር ሉፕ  ለሚያጠቡ እናቶች የወሊድ መከላከያ  የወሊድ መከላከያ መርፌ  በክንድ የሚቀበር ወመ  የወሊድ መከላከያ ክኒን  የወንድ ኮንዶም  የሴት ኮንዶም  ድንገተኛ የወሊድ መቆጣጠሪያ  ስታንዳርድ የቀን አቆጣጠር ዘዴ በዶቃ/ጨሌ መሳይ……...  ህፃኑን የጡት ወተት ብቻ መመገብ  ቀን አቆጣጠር ዘዴ  የወንድ የዘር ፍሬ ውጭ ማፍሰስ  ሌላ ባህላዊ ዘዴ | | | | | | | | | | ጠቅላላ ጉብኝት ብዛት  ___  ___  ___  ___  ___  ___  ___  ___  ___  ___  ___  ___  ___  ___  ___ | | | | | | | | | | | | | | | | | | የአዲስ ደንበኞች ብዛት  ___  ___  ___  ___  ___  ___  ___  ___  ___  ___  ___  ___  ___  ___  ___ | | | | |  |
| 39b | **ከቤተሰብ ምጣኔ አገልግሎት መዝገብ ላይ መዝግቡ:**  ባለፈው ወር ውስጥ ጠቅላላ የተሸጡ የቤተሰብ ምጣኔ ዘዴዎች ብዛት ( ለእያንዳንዱ ዘዴ በተናጠል ሙላ/ዪ) | | በማሕፀን የሚቀበር ሉፕ  ለሚያጠቡ እናቶች የወሊድ መከላከያ  የወሊድ መከላከያ መርፌ  በክንድ የሚቀበር ወመ  የወሊድ መከላከያ ክኒን  የወንድ ኮንዶም  የሴት ኮንዶም  ድንገተኛ የወሊድ መቆጣጠሪያ  ስታንዳርድ የቀን አቆጣጠር ዘዴ በዶቃ/ጨሌ  ሌላ ባህላዊ ዘዴ | | | | | | | | | | | | | | | | | | | | | የተሸጡ መድሀኒቶች  ___  ___  ___  ___  ___  ___  ___  ___  ___ | | | | | | | | | | | |  |
|  | **ጥ.**  K **ን ተመልከት/ቺ:** የተቋሙ ዓይነት? | | ሆስፒታል 1  የጤና ጣቢያ 2  የጤና ኬላ 3  የጤና ክሊኒክ 4  ፋርማሲ/መድሀኒት ቤት 5  የመድሀኒት መደብር 6  ሌላ 7 | | | | | | | | | | | | | | | | | | | | | | | | | | | | | | | | | ኮድ: 5, 6 ወይም 7 ከተከበበ ወደ ጥ.45 እለፉ/ፊ |
| 40 | ከታች ከተዘረዘሩት ውስጥ በዚህ ተቋም ውስጥ የሚሰጡት የትኞቹ አገልግሎቶች ናቸው?  የቅድመ ወሊድ ክትትል  የወሊድ/የማዋለድ አገልግሎት  ከድህረ ወሊድ ክትትል  የውርጃ አገልግሎት  ከውርጃ በኃላ የሚደረግ ክትትል  **እያንዳንዱን የአገልግሎት አይነቶች እያነበባችሁ በማጣራት ተገቢውን ኮድ መምረጠጡ፡፡** | |  | | | | | | | | | | | | | | | | | አዎ  1  1  1  1  1 | | | | | | | | | | | | የለም  0  0  0  0  0 | | | | ከወሊድ/ከውርጃ በኃላ የሚደረግ ምርመራ የለም ከሆነ ወደ ጥያቄ  45 እለፉ/ፊ  የድህረ ወሊድ ክትትል የለም ከሆነ እና ከውርጃ በኃላ አዎ ከሆነ ወደ ጥያቄ  43 እለፉ/ፊ |
| 41 | እናትየዋ ልጅ ከወለዱ በኃላ ከጤና ተቋሙ ከመውጣታቸው በፊት ከዚህ በታች ከተዘረዘሩት የትኞቹ ምርመራዎች/የምክር ሁኔታዎች በጤና ባለሙያው ተሰጥቷቸው ነበር:  የተመጣጠነ ምግብ አመጋገብ ዘዴ እና የሰውነት እንቅስቃሴ ስለማድረግ  ከወሊድ በኃላ ሊያጋጥም ስለሚችል የአእምሮ ህመም/ድብርት  በድጋሚ ማርገዝ/ስለመውለድ ሁኔታ  **ለእርግዝና ጤናማ የሆነ ጊዜና የተራራቀ እርግዝናን በተመለከተ**  በቤተሰብ ምጣኔ ዘዴዎች ላይ ምክር:  ህፃኑን የጡት ወተት ብቻ በመመገብ እርግዝና መከላከያ ዘዴ …  ለረጅም ጊዜ የሚያገለግሉ ዘዴዎች  ለአጭር ግዜ አራርቆ መውለድ የሚያገለገሉ ዘዴዎች  ከላይ የተጠቀሱት አንዱም አልተሰጠም  **እያንዳንዱን አማራጮች እያነበባችሁ ተገቢውን ኮድ ምረጡ፡፡ .** | |  | | | | | | | | | | | | | | | | አዎ  1  1  1  1  1  1  1 | | | | | | | | | | | | የለም  0  0  0  0  0  0  0 | | | | |  |
| 42 | ሴቶቹ *ከወሊድ በኃላ* ለሚደረገው ምርመራ ወደ ጤና ተቋሙ በሚመጡበት ጊዜ የቤተሰብ ምጣኔ ዘዴ ይሰጣቸዋል? | | አዎ 1  የለም 0 | | | | | | | | | | | | | | | | | | | | | | | | | | | | | | | | |  |
|  | **ጥ.40ን ተመልከቱ:**  ከውርጃ በኃላ የሚሰጠው አገልግሎት ተሰጥቷል? | | አዎ 1  የለም 0 | | | | | | | | | | | | | | | | | | | | | | | | | | | | | | | | | የለም ከሆነ ወደ ጥያቄ 45 እለፉ/ፊ |
| 43 | ከወርጃ በኃላ በሚደረገው ምርመራ ወቅት ከዚህ በታች ከተዘረዘሩት የትኞቹ ምርመራዎች/የምክር ሁኔታዎች በጤና ባለሙያው ለደንበኞች ተሰጥቷቸው ነበር:  ከወሊድ በኃላ ሊያጋጥምስለሚችል የአእምሮ ህመም/ድብርት  በድጋሚ ስለመውለድ ሁኔታ  ለእርግዝና ጤናማ የሆነ ጊዜና የተራራቀ እርግዝና  በቤተሰብ ምጣኔ ዘዴዎች ላይ ምክር:  ለረጅም ጊዜ የሚያገለግሉ ዘዴዎች  አራርቆ የመውለድ ዘዴ  ከላይ የተጠቀሱት አንዱም አልተሰጠም  **እያንዳንዱን አማራጮች እያነበባችሁ ተገቢውን ኮድ ምረጡ፡፡** | |  | | | | | | | | | | | | | | | | አዎ  1  1  1  1  1  1 | | | | | | | | | | | | የለም  0  0  0  0  0  0 | | | | |  |
| 44 | ሴቶቹ *ከውርጃ በኃላ* ለሚደረገው ምርመራ ወደ ጤና ተቋሙ በሚመጡበት ጊዜ የቤተሰብ ምጣኔ ዘዴ ይሰጣቸዋል? | | አዎ 1  የለም 0 | | | | | | | | | | | | | | | | | | | | | | | | | | | | | | | | |  |
| 45 | ከዚህ በታች ከተዘረዘሩት የቤተሰብ ምጣኔ ዘዴዎችን የትኞቹ ላላገቡ/በጋብቻ ላይ ላልሆኑ ወጣቶች (ዕድሜያቸው ከ10-19 የሆኑትን ብቻ) በጤና ባለሙያው ተሰጥቷቸው ነበር:  የቤተሰብ ምጣኔ ዘዴዎች ላይ ምክርመስጠት  የቤተሰብ ምጣኔ ዘዴዎች መስጠት  የቤተሰብ ምጣኔ ዘዴዎች ከሌላ ቦታ እንዲያገኙ ማዘዣ መፃፍ/ወደ ሌላ ቦታ መላክ  ከላይ የተጠቀሱት አንዱም አልተሰጠም  **እያንዳንዱን አማራጮች እያነበባችሁ ተገቢውን ኮድ ምረጡ፡፡** | |  | | | | | | | | | | | | | | | አዎ  1  1  1  1  1 | | | | | | | | | | | | | | የለም  0  0  0  0  0 | | | |  |
| 46 | ይህ ተቋም በግብረስጋ ግንኙነት የሚተለለፉ በሽታ ጋር በተያያዘ ***(ኤች.አይ.ቪን ሳይጨምር)*** ማንኛውም አገልግሎት ማለትም ምርመራ፣ህክምና/ምክር ወይም ድጋፍ ይስጣል? | | አዎ 1  የለም 0 | | | | | | | | | | | | | | | | | | | | | | | | | | | | | | | | |  |
| 47 | ይህ ተቋም በኤች.አይ.ቪ ላይ/ጋር በተያያዘ ማንኛውም አገልግሎት ማለትም ምርመራ፣ህክምና/ምክር ወይም ድጋፍ ይስጣል? | | አዎ 1  የለም 0 | | | | | | | | | | | | | | | | | | | | | | | | | | | | | | | | | የለም ከሆነ ወደ ጥያቄ 50 እለፉ/ፊ |
|  | **ጥ.K ን ተመልከት/ቺ:** የተቋሙ ዓይነት? | | ሆስፒታል 1  የጤና ጣቢያ 2  የጤና ኬላ 3  የጤና ክሊኒክ 4  ፋርማሲ/መድሀኒት ቤት 5  የመድሀኒት መደብር 6  ሌላ 7 | | | | | | | | | | | | | | | | | | | | | | | | | | | | | | | | | ኮድ: 5, 6 ፣7 ከተከበበ ወደ ጥያቄ 52 እለፉ/ፊ |
| 48 | ከዚህ በታች ከተዘረዘሩት የቤተሰብ እቅድ ዘዴዎችን በኤች.አይ.ቪ ላይ አገልግሎት ለማግኘት ለሚመጡ ደንበኞች በጤና ባለሙያው ተሰጥቷቸው ነበር:  የቤተሰብ እቅድ ዘዴዎች ላይ ምክር መስጠት  የቤተሰብ እቅድ ዘዴዎች መስጠት  የቤተሰብ እቅድ ዘዴዎች ከሌላ ቦታ እንዲያገኙ ማዘዣ መፃፍ/ወደ ሌላ ቦታ መላክ  ከላይ የተጠቀሱት አንዱም አልተሰጠም  **እያንዳንዱን አማራጮች እያነበብክ/ሽ ተገቢውን ኮድ ምረጥ/ጪ** | |  | | | | | | | | | | | | | አዎ  1  1  1  1 | | | | | | | | | | | | | የለም  0  0  0  0 | | | | | | |  |
| 49 | ለኤች.አይ ቪ የምክር አገልግሎት በሚሰጥበት ወቅት የጤና ባለሙያው ደንበኞችን/ ተጠቃሚዎችን  ስለ ወደፊት ልጅ የመውለድ ሀሳብ /ስነተወልዶ ጠይቆ ነበር?  ደንበኛው/ዋ/ተጠቃሚው/ዋ ስለሚመርጠው/ስለምትመርጠው የቤተሰብ ምጣኔ ዘዴዎች አወያይቶት/አት ነበር?  ሁለት ዘዴዎች መጠቀም እንደሚችል/ምትችል አወያይቶት/አት ነበር?  ኮንዶም ተሰጥቷቸው ነበር?  የመረጠችው የቤተሰብ ምጣኔ ዘዴ በተመለከተ መመሪያና ጎጂ ጎኖቹ ላይ መወያየት?  የቤተሰብ ምጣኔ ዘዴ ተሰጦታል/ቷል/ቀርቧል? | |  | | | | | | | | አዎ  1  1  1  1  1  1 | | | | | የለም  0  0  0  0  0  0 | | | | | | | | | | | | | አላውቅም  -88  -88  -88  -88  -88  -88 | | | | | | |  |
|  | **ጥ.15ን ተመልከቱ:**  የቤተሰብ ምጣኔ ዘዴ ይሰጣሉ/ያቀርባሉ? | | አዎ 1  የለም 0 | | | | | | | | | | | | | | | | | | | | | | | | | | | | | | | | | የለም ከሆነ ወደ ጥያቄ R እለፉ/ፊ |
| 50 | **የቤተሰብ እቅድ ምርመራ የሚካሄድበትን ክፍል እንዲያሳዩአችሁ ጠይቁ፡፡**  **ለሚከተሉት ዝርዝሮች ሁሉ እያንዳንዱን ዕቃ/ቁሳቁስ በምርመራው ክፍል ውስጥ ወይም ከምርመራው ክፍል አጠገብ መሆኑን አረጋግጡ፡፡**  [ኢንፌክሽን ለመቆጣጠር የሚያስችል ቁሳቁስ መኖሩን ተመልከቱ፡፡]  ታ፡ ታይቷል  አያ፡ አለ ግን ያልታየ  አይ፡ አይመለከተውም  ተጠያቂው መልስ ሰጥቶዎታል. ከላይ የተጠቀሱት ሁሉም ወይም ምንም መሞላቱን ያረጋግጡ. | | የቧንቧ ውሃ  ሌላ የቧንቧ ውሃ ( በጀሪካን የቧንቧመክፈቻ ያለው ወይም የውሃ መቅረቢያ ጆግ ያለው)  ውሃ በባልዲ ወይም በሌላ ዕቃ (በድጋሚ የሚጠቀሙበት ውሃ)  የእጅ መታጠቢያ ሳሙና  ለአንድ ጊዜ ብቻ የሚጠቀሙበት ደረቅ የእጅ ፎጣ  ክዳን ያለው የቆሻሻ ማስቀመጫ እና ፕላስቲክ ሽፋን/መሸፈኛ)  ስለታማ ነገሮች ማስቀመጫ  ለአንዴ የሚያገለግል ጓንት  ኢንፌክሽን መከላከያ መድሃኒት  ለአንዴ የሚያገለግል መርፌ እና ሲሪንጅ  ድምፅ የማያሰማ የመመርመሪያ ክፍል  ከውጭ ሰው የማያሳይ መመርመሪያ ክፍል  ለመመርመሪያ የሚያገለግል ጠረጴዛ  ለደንበኞች በቤተሰብ እቅድ ላይ ትምህርት ሰጭ ዶክሜንቶች/ቁሳቁሶች | | | | | | | | | | | | | | | | | | | | | | ታ  1  1  1  1  1  1  1  1  1  1  1  1  1 | | | | | | አያ  2  2  2  2  2  2  2  2  2  2  2  2  2  2  2  2 | | | አየ  -77  -77  -77  -77  -77  -77  -77  -77  -77  -77  -77  -77  -77  -77  -77  -77 | |  |
| 51 | **የቤተሰብ እቅድ አገልግሎት የሚሰጥበትን ቦታ በመመልከት የሚከተለውን ሙሉ፡፡** | | ወለል: የተጠረገ ፣ ምንም የሚታይ ቆሻሻ የለም  ፊት ለፊት የሚገኝ መመዝገቢያ ካውንተር/ባልኮኒ: ተወልውሏል ንፁህ ነው፣ ምንም የሚታይ ቆሻሻ የለውም  ስራው በሚሰራበት ቦታ የተሰባበሩ ቁሳቁሶች፣ወረቀቶች፣የተዘበራረቀ ወይም ካርቶኖች እና ቆሻሻዎች የለውም  ግድግዳ: በተወሰነ ደረጃ ንፁህ ነው  በሮች: የለም ወይም የተወሰነ ቦታ ተሰብሯል  ግድግዳ: የለም ወይም የተወሰነ ቦታ ፈርሷል  ጣሪያ: የለም ወይም የተወሰነ ቦታ ፈርሷል/ተበላሽቷል | | | | | | | | | | | | | | | | | | | | | | | | አዎ  1  1  1  1  1  1  1 | | | | | | | የለም  0  0  0  0  0  0  0 | |  |
| **ከጥያቄ 52a - 52c ያሉ ጥያቄዎች በዚህ የጤና አገልግሎት መስጫ ለሚሰጡ የቤተሰብ ምጣኔ ዘዴ ሁሉ የሚጠየቁ ጥያቄዎች ናቸው፡፡ በጥያቄ 31 የተዘረዘሩትን (ሴቶችን እና ወንዶችን በኦፕራሲዮን ማምከን በስተቀር) ይመለከታል.** | | | | | | | | | | | | | | | | | | | | | | | | | | | | | | | | | | | | |
| 52a | የሚከተለውን የቤተሰብ እቅድ ዘዴ ( ) እንደምትሰጡ ነግረውኛል፡፡ ሊያሳዩኝ ይችላሉ?  *መልሱ የለም ከሆነ፣ የቤተሰብ ምጣኔ ዘዴው ዕቃ ግምጃ ቤት ውስጥ የለም? በማለት ያረጋግጡ*  *ከ52a - 52c ያሉ ጥያቄዎች ለጥያቄ ቁጥር 31በተሰጡ መልሶች መሰረት በተቋሙ ውስጥ ለሚሰጡ ዘዴዎች ሁሉ ይጠየቃሉ (ሴቶችን እና ወንዶችን በኦፕራሲዮን ማምከን በስተቀር)፡፡* | | ስቶር ውስጥ አለ እና ታይቱዋል ........................................................................1  ስቶር ውስጥ አለ ግን አልታየም .......................................................................2  ስቶር ውስጥ የለም ..............................................................................3  መልስ የለም .............................................................................. -99 | | | | | | | | | | | | | | | | | | | | | | | | | | | | | | | | | መልሱ 1 ወይም 2 ከሆነ ወደ ጥያቄ 52c ይሂዱ |
| 52b | *የቤተሰብ ምጣኔ ዘዴው ( ) ለምን ያህል ቀን ነው የሌለው*?  *ከ52a - 52c ያሉ ጥያቄዎች ለጥያቄ ቁጥር 31በተሰጡ መልሶች መሰረት በተቋሙ ውስጥ ለሚሰጡ ዘዴዎች ሁሉ ይጠየቃሉ (ሴቶችን እና ወንዶችን በኦፕራሲዮን ማምከን በስተቀር)፡፡* | |  | | | | | | | | | | | | | | | | | | | | | | | | | | | | | | | | |  |
| 52c | ባለፉት 3 ወራት ውስጥ ይህ የቤተሰብ እቅድ ዘዴ ( ) ሳይኖር ቀርቶ ያውቃል?  *ከ52a - 52c ያሉ ጥያቄዎች ለጥያቄ ቁጥር 31በተሰጡ መልሶች መሰረት በተቋሙ ውስጥ ለሚሰጡ ዘዴዎች ሁሉ ይጠየቃሉ (ሴቶችን እና ወንዶችን በኦፕራሲዮን ማምከን በስተቀር)፡፡* | |  | | | | | | | | | | | | | | | | | | | | | | | | | | | | | | | | |  |
| 53 | **ከጥያቄ 53 እስከ 56, ላሉት የቤተሰብ እቅድ** **ዘዴዎች የሚቀመጡበትን ቦታ /ስቶሩን/ግምጃ ቤቱን በመመልከት ለሚከተሉትን ሁኔታዎች መልስ/ሺ።**  የቤተሰብ እቅድ ዘዴዎች ሁሉ ወለሉ /መሬት ላይ አይደለም ያሉት? | | አዎ 1  የለም 0 | | | | | | | | | | | | | | | | | | | | | | | | | | | | | | | | |  |
| 54 | የቤተሰብ እቅድ ዘዴዎች ሁሉ ውሃ በማይደርስበት ቦታ /እንዳይበላሹ ተደርጎ ነው የተቀመጡት? | | አዎ 1  የለም 0 | | | | | | | | | | | | | | | | | | | | | | | | | | | | | | | | |  |
| 55 | የቤተሰብ እቅድ ዘዴዎች ሁሉ ከፀሃይ ብርሃን ጨረር /በማይደርስባቸው ቦታ ነው የተቀመጡት? | | አዎ 1  የለም 0 | | | | | | | | | | | | | | | | | | | | | | | | | | | | | | | | |  |
| 56 | የዕቃ ግምጃ ቤቱ/ ስቶሩ ከአይጥ፣ከበረሮ፣ከተባይ የመሳሰሉ የፀዳ ነው? | | አዎ 1  የለም 0 | | | | | | | | | | | | | | | | | | | | | | | | | | | | | | | | |  |
| **መልስ ሰጭዋ/ው ቃለመጠይቅ ለማድረግ ጊዜያቸውን ስለሰጡህ/ሽ አመስግን/ኚ፡፡**  **መረጃ ሰብሳቢ፡ መልስ ሰጭ እዚህ ላይ ጨርሰዋል ፡፡ ነገር ግን አንቺ/ተ ማጠናቀቅ ያለብሽ/ህ ከተቋሙ ውጭ የሚሞሉ ተጨማሪ ሁለት መጠይቆች አሉ፡፡**  **.** | | | | | | | | | | | | | | | | | | | | | | | | | | | | | | | | | | | | |
| **ጥናቱ የሚካሄድበት ቦታ እና የቃለመጠይቁ ውጤት** | | | | | | | | | | | | | | | | | | | | | | | | | | | | | | | | | | | | |
| S a | **ወደ ጤና ተቋሙ መግቢያ አጠገብ የጂ.ፒ. ኤስ ንባብ ውሰጂ፡፡ የጂፒ.ኤስ ኮኦርድኔቶች የሚወሰዱት ከጊቢ ውጭ ብቻ ነው፡፡**  **የጂፒ.ኤሱ የትክክለኛነት ልኬት ከ6 ሜትር በታች ከሆነ ቦታውን መዝግብ/ቢ።** | *በ ODK ሶፍት ዌር መመሪያ ይሰጣል፡፡*  ቦታውን መዝግቡ | | | | | | | | | | | | | | | | | | | | | | | | | | | | | | | | |  | |
| S b | የጤና ተቋሙን መግቢያ በር ፎቶ ለማንሳት ፈቃድ ጠይቅ/ቂ።  ፎቶ ለማንሰት ፈቃድ/ስምምነት አገኘህ/ሽ? | አዎ 1  የለም 0 | | | | | | | | | | | | | | | | | | | | | | | | | | | | | | | | | የለም ከሆነ ወደ T እለፉ/ፊ | |
| T | ምንም ዓይነት ሰው በፎቶግራፉ ላይ አለመግባቱን አረጋግጥ/ጪ። | *በ ODK ሶፍት ዌር በቀጥታ መመሪያ ይሰጣል፡፡*  ፎቶግራፍ አንሱ  መስሉን ምረጡ | | | | | | | | | | | | | | | | | | | | | | | | | | | | | | | | |  | |
|  | የአገልግሎት መስጫ ቦታ ጥናት ውጤት መዝግብ/ቢ። | ተጠናቋል 1  ተቋሙ አልተገኘም 2  ተላልፏል 3  ፈቃደኛ አይደሉም 4  በከፊል ተጠናቋል 5  ሌላ 6 | | | | | | | | | | | | | | | | | | | | | | | | | | | | | | | | |  | |
