# Supplementary material for: Does proximity of women to facilities with better choice of contraceptives affect their contraceptive utilization in rural Ethiopia?
Source: PLoS One. 2017 Nov 13;12(11):e0187311. doi: 10.1371/journal.pone.0187311 (PMC5683563; doi:10.1371/journal.pone.0187311)
Supplement: S1 File — (ZIP) [file pone.0187311.s004.zip › Questionnaires/English version/PMA2020_Female respondent survey_R1.docx]

| **mADDS –Female Respondent Questionnaire** |
| --- |

| **NO** | **QUESTIONS AND FILTERS** | | | | **CODING CATEGORIES** | | | | | | | | | | **SKIP** |
| --- | --- | --- | --- | --- | --- | --- | --- | --- | --- | --- | --- | --- | --- | --- | --- |
| **IDENTIFICATION**  **Please record the following identifying information prior to beginning the interview.** | | | | | | | | | | | | | | | |
| A | | Are you in the correct household?  This is the picture of the front of the home taken during the household roster.  *ODK will display the photo taken as part of the Household Roster linked to this Female Respondent Questionnaire.* | | | Yes 1  No 0 | | | | | | | | | |  |
| B | | How many times have you visited this household to interview this female respondent? | | | 1^st^ time 1  2^nd^ time 2  3^rd^ time 3 | | | | | | | | | |  |
| C | | Interviewer’s name: Is this your name?  If not, please record your name:  *ODK will display the name associated with the phone’s serial number* | | | Yes 1  No 0 | | | | | | | | | |  |
|  |  |  |  |  |  | | | | | | | | | |  |
| D | | **CURRENT DATE AND TIME DISPLAYED ON SCREEN.**  Is this date and time correct? | | | Yes 1  No 0 | | | | | | | | | | Skip to F if Yes |
| E | | Record the correct date and time. | | | Date | Day | | Month | | | | Year | | |  |
|  |  |  |  |  | Time | Hours | | Minutes | | | | AM/PM | | |  |
| F | | The following information is from the Household Roster. Please review to make sure you are interviewing the correct respondent.  *ODK will display the Region, Zone, Woreda/District, Kebele/Locality, Enumeration Area, Structure Number, and Household Number entered into the Household Roster linked to this Female Respondent Questionnaire.* | | |  | | | | | | | | | |  |
| G | | How well acquainted are you with the respondent? | | | Very well acquainted 1  Well acquainted 2  Not well acquainted 3  Not acquainted 4 | | | | | | | | | |  |
| H | | Is the respondent present and available to be interviewed today? | | | Yes 1  No 0 | | | | | | | | | | Skip to M IF NO |
| **INFORMED CONSENT**  **Find the woman between the age of 15-49 associated with this Female Respondent Questionnaire. The interview must have auditory privacy. Read the following greeting:** | | | | | | | | | | | | | | | |
| Hello. My name is ____________________________________ and I am working for the Addis Ababa University, and Federal Ministry of Health. We are conducting a local survey that asks women about various reproductive health issues. We would very much appreciate your participation in this survey. This information will help us inform the government to better plan health services. The survey usually takes between 15 and 20 minutes to complete. Whatever information you provide will be kept strictly confidential and will not be shown to anyone other than members of our survey team.  Participation in this survey is voluntary, and if we should come to any question you don't want to answer, just let me know and I will go on to the next question; or you can stop the interview at any time. However, we hope that you will participate in this survey since your views are important.  At this time, do you want to ask me anything about the survey? | | | | | | | | | | | | | | | |
| I | | Provide a paper copy of the Consent Form to the respondent and explain it. Then, ask: May I begin the interview now? | | | Yes 1  No 0 | | | | | | | | | | Skip to M if No |
| J | | Respondent’s signature  **PLEASE ASK THE RESPONDENT TO SIGN OR CHECK THE BOX IN AGREEMENT OF THEIR PARTICIPATION.** | | | GATHER SIGNATURE:  Check box: ☐ | | | | | | | | | |  |
| K | | Interviewer’s name  **PLEASE RECORD YOUR NAME AS A WITNESS TO THE CONSENT PROCESS.** | | |  | | | | | | | | | |  |
| L | | Respondent’s name  **PLEASE RECORD THE FIRST NAME OF THE RESPONDENT.** | | |  | | | | | | | | | |  |
| **NO** | | **QUESTIONS AND FILTERS** | | | **CODING CATEGORIES** | | | | | | | | | | **SKIP** |
| **Section 1 – Respondent’s Background, Marital Status, HH characteristics**  **Now I would like to ask about your background and socioeconomic conditions.** | | | | | | | | | | | | | | | |
| 1 | | How old were you at your last birthday?  **RECORD AGE IN COMPLETED YEARS. PLEASE RECORD A NUMBER BETWEEN 15-49. DO NOT INTERVIEW ANYONE OUTSIDE THIS RANGE.** | | | Age | | | | | | | |  | |  |
| 2 | | What is the highest level of school you attended: primary, , secondary, technical & vocational , or higher? | | | Never Attended 0  Primary 1  Secondary 2  technical & vocational 3  Higher 4 | | | | | | | | | |  |
| 3 | | Are you currently married or living together with a man as if married?  **IF NO, ASK WHETHER THE RESPONDENT IS DIVORCED, SEPARATED, OR WIDOWED.** | | | No, never in union 0  Yes, currently married 1  Yes, living with a man 2  Not currently in union: Divorced / separated 3  Not currently in union: Widow 4 | | | | | | | | | | Skip to 8 if No, never in union |
| 4 | | Have you been married or lived with a man only once or more than once? | | | Only once 1  More than once 2 | | | | | | | | | | Skip to 5a if once and 5b if more |
| 5a | | In what month and year did you start living with your husband / partner? | | | Month: | | | | |  | | | | |  |
|  |  |  |  |  | Year: | | | | |  | | | | |  |
| 5b | | Now I would like to ask about when you started living with your current husband / partner. In what month and year was that? | | | Month: | | | | |  | | | | |  |
|  |  |  |  |  | Year: | | | | |  | | | | |  |
| 6 | | Does your husband / partner have other wives or does he live with other women as if married? | | | Yes 1  No 0  Don’t know -88 | | | | | | | | | |  |
|  | | **CHECK 3:** Currently married? | | | Yes 1  No 0 | | | | | | | | | | Skip to 8 if No |
| 7 | | Is your husband/partner living with you now or is he staying elsewhere? | | | Living with respondent 1  Staying elsewhere 2 | | | | | | | | | |  |
| **Section 2 – Reproduction, Pregnancy & Fertility Preferences**  **Now I would like to ask about all the births you have had during your life.** | | | | | | | | | | | | | | | |
| 8 | | How many times have you given birth?  **0 IS A POSSIBLE ANSWER.** | | | Number of births | | | | | | | |  | | Skip to 13 if 0 |
|  | | Were all of those live births?  **IF NO, GO BACK AND CHANGE FQ8 TO RECORD ONLY LIVE BIRTH EVENTS** | | | Yes 1  No 0 | | | | | | | | | |  |
| 9 | | When was your most recent birth?  **PLEASE RECORD THE DATE OF THE LAST BIRTH.**  **THE DATE SHOULD BE FOUND BY CALCULATING BACKWARDS FROM MEMORABLE EVENTS IF NEEDED.** | | | Month | | | | Year | | | | | | Skip to 11 if not in last year and/or Q8 is 1 |
| 10 | | When did you give birth before the most recent one?  **PLEASE RECORD THE DATE OF THE BIRTH BEFORE THE LAST.**  **THE DATE SHOULD BE FOUND BY CALCULATING BACKWARDS FROM MEMORABLE EVENTS IF NEEDED.** | | | Month | | | | Year | | | | | |  |
| 11 | | Is your last baby / child still alive? | | | Yes 1  No 0  Don’t know -88 | | | | | | | | | | Skip to 13 if Yes |
| 12 | | When did your last baby / child die?  **PLEASE RECORD THE DATE OF THE CHILD’S DEATH.**  **THE DATE SHOULD BE FOUND BY CALCULATING BACKWARDS FROM MEMORABLE EVENTS IF NEEDED.** | | | Month | | | | Year | | | | | |  |
| 13 | | When did your last menstrual period start?  **IF YOU SELECT DAYS, WEEKS, MONTHS OR YEARS, YOU WILL ENTER A NUMBER FOR X ON THE NEXT SCREEN.** | | | Days ago: | | | | | |  | | | |  |
|  |  |  |  |  | Weeks ago: | | | | | |  | | | |  |
|  |  |  |  |  | Months ago: | | | | | |  | | | |  |
|  |  |  |  |  | Years ago: | | | | | |  | | | |  |
|  |  |  |  |  | Menopausal / Hysterectomy 1  Before last birth 2  Never menstruated 3 | | | | | | | | | |  |
| 14 | | Are you pregnant now? | | | Yes 1  No 0  Unsure 2 | | | | | | | | | | Skip to 16 if No |
| 15 | | How many months pregnant are you?  **PLEASE RECORD THE NUMBER OF COMPLETED MONTHS. ENTER -88 FOR DO NOT KNOW.** | | | Number of months | | | | | | | |  | |  |
|  | | **CHECK 14:** Currently pregnant? | | | Yes 1  No 0 | | | | | | | | | | 16a if no  16b if yes |
| 16a | | **Now I have some questions about the future.** Would you like to have a/another child or would you prefer not to have any / anymore children? | | | Have a/another child 1  No more/prefer no children 2  Says she can’t get pregnant 3  Undecided / Don’t know -88 | | | | | | | | | | Skip to 17a if 1 and 18 for all other |
| 16b | | **Now I have some questions about the future.**  After the child you are expecting now, would you like to have another child, or would you prefer not to have any more children? | | | Have a/another child 1  No more/prefer no children 2  Says she can’t get pregnant 3  Undecided / Don’t know -88 | | | | | | | | | | Skip to 17b if 1 and 18 for all other |
| 17a | | How long would you like to wait from now before the birth of a/another child?  **IF YOU SELECT MONTHS OR YEARS, YOU WILL ENTER A NUMBER FOR X ON THE NEXT SCREEN** | | | Months: | | | | | | | |  | |  |
|  |  |  |  |  | Years: | | | | | | | |  | |  |
|  |  |  |  |  | Soon / now 1  Other 2  Says she can’t get pregnant 3  Don’t know -88 | | | | | | | | | |  |
| 17b | | After the birth of the child you are expecting now, how long would you like to wait before the birth of another child?  **IF YOU SELECT MONTHS OR YEARS, YOU WILL ENTER A NUMBER FOR X ON THE NEXT SCREEN** | | | Months: | | | | | | | |  | |  |
|  |  |  |  |  | Years: | | | | | | | |  | |  |
|  |  |  |  |  | Soon / now 1  Other 2  Says she can’t get pregnant 3  Don’t know -88 | | | | | | | | | |  |
|  | | **CHECK 8:** Number of births  **CHECK 14**: Currently pregnant? | | | Number of births | | | | | | | |  | | Skip to 19 if 0 births and 14: No.  Skip to 18a if 14: no and 18b if 14: yes |
|  |  |  |  |  | Yes 1  No 0 | | | | | | | | | |  |
| 18a | | **Now I would like to ask a question about your last birth.** At the time you became pregnant, did you want to become pregnant then, did you want to wait until later, or did you not want to have any / anymore children at all? | | | Then 1  Later 2  Not at all 3 | | | | | | | | | |  |
| 18b | | **Now I would like to ask a question about your current pregnancy.**  At the time you became pregnant, did you want to become pregnant then, did you want to wait until later, or did you not want to have any / anymore children at all? | | | Then 1  Later 2  Not at all 3 | | | | | | | | | |  |
| **Section 3 – Contraception**  **Now I would like to ask about the times you or your partner may have used a method to avoid getting pregnant.** | | | | | | | | | | | | | | | |
| 19 | | | Have you ever used anything or tried in any way to delay or avoid getting pregnant? | | Yes 1  No 0 | | | | | | | | | | Skip to 25 if No |
| 20 | | | How old were you when you first used a method to delay or avoid getting pregnant?  **ENTER THE AGE IN YEARS. ENTER 0 IF SHE NEVER USED A METHOD. ENTER -88 IF RESPONDENT DOES NOT KNOW.** | | Age | | | |  | | | | | |  |
| 20a | | | How many living children did you have at that time, if any? | | Number | | | |  | | | | | |  |
| 21 | | | Which method did you first use to delay or avoid getting pregnant?  **DO NOT READ THE METHOD CHOICES. SCROLL TO BOTTOM TO SEE ALL CHOICES.** | | Female sterilization 1  Male sterilization 2  IUD 3  Injectables 4  Implants 5  Pill 6  Condom 7  Female condom 8  Emergency Contraception…………..9  Standard Days/Cycle Beads………10  Lactational Amen. Method 11  Rhythm method 12  Withdrawal 13 | | | | | | | | | |  |
|  | | | **CHECK 14:** Currently pregnant? | | Yes 1  No 0 | | | | | | | | | | Skip to 25 if yes |
| 22 | | | Are you/your partner currently doing something or using any method to delay or avoid getting pregnant? | | Yes 1  No 0 | | | | | | | | | | Skip to 25 if No |
| 23 | | | Which method or methods are you using?  Anything else?  **SELECT ALL METHODS MENTIONED. SCROLL TO BOTTOM TO SEE ALL CHOICES.** | | 1. Female sterilization  2. Male sterilization  3. IUD  4. Injectables  5. Implants  6. Pill  7. Condom  8. Female condom  9. Emergency Contraception      10. Std Days/Cycle Beads  11. LAM    12. Rhythm method  13. Withdrawal | | | | | | | | Y  1  1  1  1  1  1  1  1  1  1  1  1  1  1  1 | N  0  0  0  0  0  0  0  0  0  0  0  0  0  0  0 | Skip based on most effective method only  Skip to 29 if main method is 3-17 |
| 24 | | | Did the provider tell you / your partner that this method was permanent? | | Yes 1  No 0 | | | | | | | | | | Skip to 29 |
| 25 | | | Do you know of a place where you can obtain a method of family planning? | | Yes 1  No 0 | | | | | | | | | |  |
|  | | | **CHECK 14:** Currently pregnant? | | Yes 1  No 0 | | | | | | | | | | 26a if no  26b if yes |
| 26a | | | You said that you are not currently using a contraceptive method. Do you think you will use a contraceptive method to delay or avoid getting pregnant at any time in the future? | | Yes 1  No 0 | | | | | | | | | |  |
| 26b | | | Do you think you will use a contraceptive method to delay or avoid getting pregnant at any time in the future? | | Yes 1  No 0 | | | | | | | | | |  |
|  | | | **CHECK 19**: ever used contraceptives? | | Yes 1  No 0 | | | | | | | | | | Skip to 43 if No |
| 27 | | | In the last 12 months, have you ever done something or used a method to delay or avoid getting pregnant? | | Yes 1  No 0 | | | | | | | | | | Skip to 43 if No |
| 28 | | | Which method did you use most recently?  Anything else?  **SELECT MOST EFFECTIVE METHOD (HIGHEST METHOD IN LIST).**  **SCROLL TO BOTTOM TO SEE ALL CHOICES** | | Female sterilization 1  Male sterilization 2  IUD 3  Injectables 4  Implants 5  Pill 6  Condom 7  Female condom 8  Emergency Contraception 9  Std Days/Cycle Beads 10  LAM 11  Rhythm method 12  Withdrawal 13 | | | | | | | | | |  |
| 29 | | | When did you begin using your (MOST RECENT / CURRENT METHOD)?  **PLEASE RECORD THE DATE.**  **THE DATE SHOULD BE FOUND BY CALCULATING BACKWARDS FROM MEMORABLE EVENTS IF NEEDED.** | | Month | | | | Year | | | | | |  |
|  | | | **CHECK 22:** Currently using contraceptives? | | Yes 1  No 0 | | | | | | | | | | Skip to 32 if Yes |
| 30 | | | When did you stop using your (MOST RECENT METHOD)?  **PLEASE RECORD THE DATE.**  **THE DATE SHOULD BE FOUND BY CALCULATING BACKWARDS FROM MEMORABLE EVENTS IF NEEDED.** | | Month | | | | Year | | | | | |  |
| 31 | | | Why did you stop using your (MOST RECENT METHOD)? | | Infrequent sex / husband away 1  Became pregnant while using 2  Wanted to become pregnant 3  Husband / partner disapproved 4  Wanted more effective method 5  No method available 6  Health concerns 7  Side effects 8  Lack of access / too far 9  Costs too much 10  Inconvenient to use 11  Fatalistic 12  Difficult to get pregnant / menopausal 13  Interferes with body’s processes 14  Other 15  Don’t know -88 | | | | | | | | | |  |
| 32 | | | Where did you obtain your (MOST RECENT / CURRENT METHOD) when you started using it?  **SCROLL TO BOTTOM TO SEE ALL CHOICES** | | Public sector  Govt. Hospital/polyclinic 1  Govt. Health center 2  Govt. Health post/HEWs 3  Family planning clinic 4  Private medical sector  Private hospital/clinic 5  Pharmacy/drug store 6  Other source  Shop/market 7  Community volunteer (HDA) 8  Friend / relative 9  NGO 10  Other 11  Don't know -88 | | | | | | | | | |  |
| 33 | | | In the last 12 months, have you paid any fees for family planning services (including the most recent/current method)? | | Yes 1  No 0 | | | | | | | | | | Skip to 35 if No |
| 34 | | | How much did you pay?  **ENTER ALL PRICES IN BIRR. ENTER -88 IF RESPONDENT DOES NOT KNOW.** | | Fee: | | | | | | | | | |  |
| 35 | | | When you obtained your (MOST RECENT / CURRENT METHOD), were you told by the provider about side effects or problems you might have with a method to delay or avoid getting pregnant? | | Yes 1  No 0 | | | | | | | | | | Skip to 37 if No |
| 36 | | | Were you told what to do if you experienced side effects or problems? | | Yes 1  No 0 | | | | | | | | | |  |
| 37 | | | At that time, were you told by the family planning provider about methods of family planning other than the (MOST RECENT/CURRENT METHOD) that you could use? | | Yes 1  No 0 | | | | | | | | | |  |
| 38 | | | During that visit, did you obtain the method you wanted to delay or avoid getting pregnant? | | Yes 1  No 0 | | | | | | | | | | Skip to 40 if yes |
| 39 | | | If not, why not?  (Why didn’t you obtain the method you wanted?) | | Method out of stock that day 1  Method not available at all 2  Provider not trained to provide the method 3  Provider recommended a different method 4  Not eligible for method 5  Decided not to adopt a method 6  Too costly 7  Other 8 | | | | | | | | | |  |
| 40 | | | During that visit, who made the final decision about what method you got? | | You alone 1  Provider 2  Partner 3  You and provider 4  You and partner 5  Other 6 | | | | | | | | | |  |
|  | | | **CHECK 32:** Where did you obtain your (MOST RECENT / CURRENT METHOD)? | | Public sector  Govt. Hospital/polyclinic 1  Govt. Health center 2  Govt. Health post/HEWs 3  Family planning clinic 4  Private medical sector  Private hospital/clinic 5  Pharmacy/drug store 6  Other source  Shop/market 7  Community volunteer/HDA 8  Friend / relative 9  NGO 10  Other 11  Don't know -88 | | | | | | | | | | Skip to 44 if 32 is 14-17 |
| 41 | | | Would you return to this provider? | | Yes 1  No 0 | | | | | | | | | |  |
| 42 | | | Would you refer your relative or friend to this provider / facility? | | Yes 1  No 0 | | | | | | | | | |  |
|  | | | **CHECK 16:** Desire for future child?  **CHECK 17**: 2 or more years before next child?  **CHECK 22:** Currently using contraceptive method?  **CHECK 19:** Ever use a method? | | Have a/another child 1  No more/none 2  Says she can’t get pregnant 3  Undecided / Don’t know -88 | | | | | | | | | | Ask 43 to non users (current or ever) who do not want a/another child or not before 2 years |
|  |  |  |  |  | No more/none 1  Less than 2 years 2  2 or more years 3 | | | | | | | | | |  |
|  |  |  |  |  | Yes, using contraceptive 1  No, not using contraceptive 0 | | | | | | | | | |  |
|  |  |  |  |  | Yes 1  No 0 | | | | | | | | | |  |
| 43 | | | **You said that you do not want any / anymore children and that you are not using a method to avoid pregnancy.**  Can you tell me the main reason why you are not using a method to prevent pregnancy? | | Infrequent sex / husband away 1  Menopausal/Hysterectomy 2  Subfecund / infecund 3  Not menstruated since last birth 4  Breastfeeding 5  Up to God / fatalistic 6  Respondent opposed 7  Husband / partner opposed 8  Others opposed 9  Religious prohibition 10  Knows no method 11  Knows no source 12  Fear of side effects 13  Health concerns 14  Lack of access / too far 15  Costs too much 16  Preferred method not available 17  No method available 18  Inconvenient to use 19  Interferes with body’s processes 20  Other 21  Don’t know -88 | | | | | | | | | |  |
| 44 | | | In the last 12 months, were you visited by a health worker who talked to you about family planning? | | Yes 1  No 0 | | | | | | | | | |  |
| 45 | | | In the last 12 months, have you visited a health facility for care for yourself (or your children)? | | Yes 1  No 0 | | | | | | | | | | Skip to 47 if no |
| 46 | | | Did any staff member at the health facility speak to you about family planning methods? | | Yes 1  No 0 | | | | | | | | | |  |
| 47 | | | In the last few months have you:  Heard about family planning on the radio?  Seen anything about family planning on the television?  Read about family planning in a newspaper or magazine? | |  | | | | Yes  1  1  1 | | | | No  0  0  0 | |  |
| 48 | | | How old were you when you first had sexual intercourse?  **ENTER THE AGE IN YEARS. ENTER 0 IF SHE NEVER HAD SEX. ENTER -88 IF RESPONDENT DOES NOT KNOW.** | | Age | | | |  | | | | | | Skip to 50 if 0 |
| 49 | | | When was the last time you had sexual intercourse?  **IF 12 MONTHS (ONE YEAR) OR MORE AGO, ANSWER MUST BE RECORDED IN MONTHS, WEEKS, OR DAYS.**  **IF LESS THAN 12 MONTHS AGO, ANSWER MUST BE RECORDED IN DAYS, WEEKS OR MONTHS.**  **ENTER 0 DAYS FOR TODAY.**  **YOU WILL ENTER A NUMBER FOR X ON THE NEXT SCREEN** | | DAYS AGO | | WEEKS AGO | | MONTHS AGO | | | | YEARS AGO | |  |
| **Section 4 – Water**  **Now I would like to ask you a couple of questions about your water practices.** | | | | | | | | | | | | | | | |
| 50 | | How many hours per day do you spend collecting water in the dry season?  **ONLY RECORD RESPONDENT’S TIME; NOT ANYONE ELSE’S TIME**  **IF YOU SELECT MINUTES OR HOURS YOU WILL ENTER A NUMBER FOR X ON THE NEXT SCREEN** | | Number of hours/day | | | | | | | | |  | |  |
|  |  |  |  | Someone else collects water 33  No one collects water 44  Don’t know -88 | | | | | | | | | | |  |
| 51 | | How many hours per day do you spend collecting water in the wet season?  **ONLY RECORD RESPONDENT’S TIME; NOT ANYONE ELSE’S TIME**  **IF YOU SELECT MINUTES OR HOURS YOU WILL ENTER A NUMBER FOR X ON THE NEXT SCREEN** | | Number of hours/day | | | | | | | | |  | |  |
|  |  |  |  | Someone else collects water 33  No one collects water 44  Don’t know -88 | | | | | | | | | | |  |
| **Thank the respondent for her time.**  **THE RESPONDENT IS FINISHED, BUT THERE ARE STILL 2 MORE QUESTIONS FOR YOU TO COMPLETE OUTSIDE THE HOME** | | | | | | | | | | | | | | | |
| **LOCATION** | | | | | | | | | | | | | | | |
| M | | **TAKE A GPS POINT NEAR THE ENTRANCE TO THE HOUSEHOLD.**  **RECORD LOCATION WHEN THE ACCURACY IS SMALLER THAN 6 M.** | | | *Instructions are given directly by the ODK software*  RECORD LOCATION | | | | | | | | | |  |
| **QUESTIONNAIRE RESULT** | | | | | | | | | | | | | | | |
| N | | **RECORD THE RESULT OF THE FEMALE RESPONDENT SURVEY** | | | Completed 1  Not at home 2  Postponed 3  Refused 4  Partly completed 5  Incapacitated 6 | | | | | | | | | |  |
