# Supplementary material for: Does proximity of women to facilities with better choice of contraceptives affect their contraceptive utilization in rural Ethiopia?
Source: PLoS One. 2017 Nov 13;12(11):e0187311. doi: 10.1371/journal.pone.0187311 (PMC5683563; doi:10.1371/journal.pone.0187311)
Supplement: S1 File — (ZIP) [file pone.0187311.s004.zip › Questionnaires/English version/PMA2020_Householdroster_R1.docx]

| mADDS –Household Roster |
| --- |

| NO | QUESTIONS AND FILTERS | CODING CATEGORIES | | | | | | SKIP |
| --- | --- | --- | --- | --- | --- | --- | --- | --- |
| IDENTIFICATION  Please record the following identifying information prior to beginning the interview. | | | | | | | | |
| A | How many times have you visited this household? | 1^st^ time 1  2^nd^ time 2  3^rd^ time 3 | | | | | |  |
| B | Interviewer’s name: Is this your name?  If not, please record your name:  *ODK will display the name associated with the phone’s serial number* | Yes 1  No 0 | | | | | |  |
|  |  |  | | | | | |  |
| C | CURRENT DATE AND TIME DISPLAYED ON SCREEN  Is this date and time correct? | Yes 1  No 0 | | | | | | Skip to Eif No |
| D | Record the correct date and time | Date | Month | | Day | | Year |  |
|  |  | Time | Hour | Minutes | | AM/PM | |  |
| E | Region  PLEASE SELECT THE NAME OF THE REGION WHERE THE FACILITY IS LOCATED. | Tigray 1  Afar 2  Amhara 3  Oromia 4  Ethiopia Somali 5  Benishangul Gumuz 6  SNNPR 7  Gambella 8  Harari 9  Addis Ababa 10  Dire Dawa 11 | | | | | |  |
| F | District  PLEASE RECORD THE NAME OF THE DISTRICT WHERE THE HOUSEHOLD IS LOCATED. | *ODK will populate a list of appropriate district based on the region selected for SQ D* | | | | | |  |
| G | Locality name  PLEASE RECORD THE NUMBER OF THE ENUMERATION AREA WHERE THE HOUSEHOLD IS LOCATED. | *ODK will populate a list of appropriate localities based on the district selected for SQE* | | | | | |  |
| H | Enumeration area  PLEASE RECORD THE NUMBER OF THE ENUMERATION AREA WHERE THE HOUSEHOLD IS LOCATED. |  | | | | | |  |
| I | Structure number  PLEASE RECORD THE NUMBER OF THE STRUCTURE OF WHICH THIS HOUSEHOLD IS A PART FROM THE HOUSEHOLD LISTING FORM. |  | | | | | |  |
| J | Household number  PLEASE RECORD THE NUMBER OF THE HOUSEHOLD FROM THE HOUSEHOLD LISTING FORM. |  | | | | | |  |
| K | Is a member of the household and competent respondent present and available to be interviewed today? | Yes 1  No 0 | | | | | | Skip to P if No |
| INFORMED CONSENT  Find the competent member of the household. Read the following greeting: | | | | | | | | |
| Hello. My name is ________________________________ and I am working for the Addis Ababa University, and Federal Ministry of Health. We are conducting a local survey about various health issues. We would very much appreciate your participation in this survey. This information will help us inform the government to better plan health services. Whatever information you provide will be kept strictly confidential and will not be shown to anyone other than members of our survey team.  Participation in this survey is voluntary, and if we should come to any question you don't want to answer, just let me know and I will go on to the next question; or you can stop the interview at any time. However, we hope that you will participate in this survey since your views are important.  I am going to ask you questions about your family and other household members. We would then like to ask a different set of questions to female members of this household who are between the ages of 15 and 49.  At this time, do you want to ask me anything about the survey? | | | | | | | | |
| L | Provide a paper copy of the Consent Form to the respondent and explain it. Then, ask: May I begin the interview now? | Yes 1  No 0 | | | | | | Skip to P if No |
| M | Respondent’s signature  PLEASE ASK THE RESPONDENT TO SIGN OR CHECK THE BOX IN AGREEMENT OF THEIR PARTICIPATION. | GATHER SIGNATURE:  Check box: ☐ | | | | | |  |
| N | Interviewer’s signature  PLEASE RECORD YOUR NAME AS A WITNESS TO THE CONSENT PROCESS. |  | | | | | |  |
| O | Interviewee’s name  PLEASE RECORD THE FIRST NAME OF THE RESPONDENT. |  | | | | | |  |

| SECTION 1 – Household Roster  I will now ask you questions about all members of the household. Let’s begin with you. For each person who usually lives here or slept in the house last night, please record the following information: | | | | | | | | | |
| --- | --- | --- | --- | --- | --- | --- | --- | --- | --- |
| No | 1 | 2 | 3 | 4 | | 5 | 6 | 7 | 8 |
|  | First name | Sex | Age (years) | Marital Status | | Relationship to head of household | Family ID | Is this person a usual member of the household or has he/she slept in the house last night? | Eligible female respondent |
|  |  | Male 1  Female 2 |  | Married 1 Living with a partner 2 Divorced / separated 3 Widow / widower 4 Single 5 | | Head 1  Wife/Husband 2  Son/Daughter 3  Son/Daughter-in-law 4  Grandchild 5  Parent 6  Parent in law 7  Brother/Sister 8  Other 9  Don’t know -88 |  | Usual member of the household 1  Usual member of the household who did not sleep in the house last night 2  Visitor who slept in the house last night 3 | Yes 1  No 0  *ODK will determine and display eligibility* |
| 1 |  |  |  |  | |  |  |  |  |
| 2 |  |  |  |  | |  |  |  |  |
| 3 |  |  |  |  | |  |  |  |  |
| 4 |  |  |  |  | |  |  |  |  |
| 5 |  |  |  |  | |  |  |  |  |
| 6 |  |  |  |  | |  |  |  |  |
| 7 |  |  |  |  | |  |  |  |  |
| 8 |  |  |  |  | |  |  |  |  |
| 9 |  |  |  |  | |  |  |  |  |
| 10 |  |  |  |  | |  |  |  |  |
| *After recording information for one household member, the following prompt is asked to activate a looping script to record the information for another member if needed:* | | | | | | | | | |
| 9 | Are there any other usual members of your household or persons who slept in the house last night? | | | | Yes 1  No 0 | | | | Skip to 10 if No |

| Section 2 – Household Characteristics  Now I would like to ask you a few questions about the characteristics of your household. | | | | | | | | |
| --- | --- | --- | --- | --- | --- | --- | --- | --- |
| 10 | Does your household have:  Electricity?  A wall clock?  A radio?  A black/white television?  A color television?  A mobile phone?  A landline telephone?  A refrigerator?  A freezer?  Electric generator/invertor(s)?  A washing machine?  A computer?  A digital photo camera?  A non digital photo camera?  A video deck?  A DVD/CD?  A sewing machine?  A bed?  A table?  A cabinet/cupboard?  A bicycle?  A motorcycle or motor scooter?  A car or truck?  A boat with a motor?  A boat without a motor?  None of the above  READ OUT ALL TYPES AND SELECT ALL THAT APPLY. |  | Yes  1  1  1  1  1  1  1  1  1  1  1  1  1  1  1  1  1  1  1  1  1  1  1  1  1  -88 | | No  0  0  0  0  0  0  0  0  0  0  0  0  0  0  0  0  0  0  0  0  0  0  0  0  0 | | |  |
| 11 | Are livestock kept on the homestead? | Yes 1  No 0 | | | | | | Skip to 13 if No |
| 12 | For each livestock, how many livestock are kept on the homestead?  Sheep / goats  Chicken / ducks / geese  Cattle / horses / donkeys  ZERO IS A POSSIBLE ANSWER. ENTER -88 FOR DO NOT KNOW. ENTER -99 FOR NO RESPONSE. |  | | | | | |  |
| Section 3 – Household Observation  Please observe the floors, roof and exterior walls | | | | | | | | |
| 13 | Main material of the floor  OBSERVE | Natural Floor  Earth/Sand 1  Dung 2  Rudimentary Floor  Wood Planks 3  Palm/Bamboo 4  Finished Floor  Parquet or polished wood 5  Vinyl/Asphalt strips 6  Ceramic Tile/Terazzo 7  Cement 8  Woolen Carpet/Synthetic Carpet 9  Linoleum/rubber carpet 10  Other 11 | | | | | |  |
| 14 | Main material of the roof  OBSERVE | Natural Floor  No Roof 1  Thatch/Palm Leaf/ Sod 2  Rudimentary Roofing  Rustic Mat 3  Palm/Bamboo 4  Wood Planks 5  Cardboard 6  Finished Roofing  Metal 7  Wood 8  Calamine/Cement Fiber 9  Ceramic Tiles/Brick Tiles 10  Cement 11  Roof Shingles 12  Asbestos/Slate Roofing Sheets 13  Other 14 | | | | | |  |
| 15 | Main material of the exterior walls  OBSERVE | Natural Walls  No Walls 1  Cane/Palm/Trunks 2  Rudimentary Walls  Bamboo with Mud 3  Stone with Mud 4  Uncovered Adobe 5  Plywood 6  Cardboard 7  Reused Wood 8  Finished Walls  Cement 9  Stone with Lime/Cement 10  Bricks 11  Cement Blocks 12  Covered Adobe 13  Wood Planks/Shingles 14  Other 15 | | | | | |  |
| Section 4 – Water, Sanitation and Hygiene  Now I would like to ask you a few questions about water, sanitation and hygiene. | | | | | | | | |
| 16 | Do you have a place to wash your hands? | Yes 1  No 0  Don’t know -88 | | | | | | Skip to 19 if No |
| 17 | Can you show it to me? | Yes 1  No 0 | | | | | | Skip to 19 if No |
| 18 | AT THE PLACE WHERE THE HOUSEHOLD WASHES THEIR HANDS, OBSERVE IF:  Soap is present  Water source is present: stored water  Water source is present: tap water  Handwashing area is near a sanitation facility  None of the above |  | | Yes  1  1  1  1  1 | | | No  0  0  0  0  0 |  |
| 19 | Which of the following water sources does your family use on a regular basis for any part of the year for any household purpose?  Piped Water  Piped into dwelling/indoor  Pipe to yard/plot  Public tap/standpipe  Tube well or borehole  Dug Well  Protected Well  Unprotected Well  Water from Spring  Protected Spring  Unprotected Spring  Rainwater  Tanker Truck  Cart with Small Tank  Surface water (River / Dam / Lake / Pond / Stream / Canal / Irrigation Channel)  Bottled Water  Sachet Water  READ OUT ALL TYPES AND CHECK ALL THAT ARE USED. |  | | Yes  1  1  1  1  1  1  1  1  1  1  1  1  1  1  1 | | | No  0  0  0  0  0  0  0  0  0  0  0  0  0  0  0 |  |
| 20 | What is the main source of drinking water for members of your household?  Piped Water  Piped into dwelling/indoor  Pipe to yard/plot  Public tap/standpipe  Tube well or borehole  Dug Well  Protected Well  Unprotected Well  Water from Spring  Protected Spring  Unprotected Spring  Rainwater  Tanker Truck  Cart with Small Tank  Surface water (River / Dam / Lake / Pond / Stream / Canal / Irrigation Channel)  Bottled Water  Sachet Water  READ OUT ALL TYPES AND CHECK THE MAIN SOURCE. MUST BE A SELECTION IN HQ 19 | 1  2  3  4  5  6  7  8  9  10  11  12  13  14  15 | | | | | |  |
| 21 | What is the main source of water used by your household for other purposes such as cooking and handwashing?  Piped Water  Piped into dwelling/indoor  Pipe to yard/plot  Public tap/standpipe  Tube well or borehole  Dug Well  Protected Well  Unprotected Well  Water from Spring  Protected Spring  Unprotected Spring  Rainwater  Tanker Truck  Cart with Small Tank  Surface water (River / Dam / Lake / Pond / Stream / Canal / Irrigation Channel)  Bottled Water  Sachet Water  READ OUT ALL TYPES AND CHECK THE MAIN SOURCE. MUST BE A SELECTION IN HQ 19. | 1  2  3  4  5  6  7  8  9  10  11  12  13  14  15 | | | | | |  |
| 22 | QUESTIONS HQ 22 TO HQ 24 WILL REPEAT X TIMES, ONCE FOR EACH WATER SOURCE SELECTED IN HQ 17. THESE SOURCES INCLUDE:  *The ODK software will list all sources selected in HQ 19.*  You mentioned you used [WATER SOURCE]. At any time of the year, does your family use water from this source for:  Drinking  Cooking  Livestock  Gardening / agriculture  Business venture  *The same question will be generated by the ODK software for all water sources selected in HQ19* |  | | Yes  1  1  1  1  1 | | No  0  0  0  0  0 | |  |
| 23 | Is [WATER SOURCE] typically available:  All of the year  Some of the year  Small part of the year 3  *The same question will be generated by the ODK software for all water sources selected in HQ19* | 1  2  3 | | | | | |  |
| 24 | At a time when you expect to have water from [WATER SOURCE], is it usually available?  Yes, always  No, intermittent and predictable  No, intermittent and unpredictable  *The same question will be generated by the ODK software for all water sources selected in HQ19* | 1  2  3 | | | | | |  |
| 25 | How many minutes does it take to make a one-way trip to [WATER SOURCE]?  ZERO IS A POSSIBLE ANSWER. INCLUDES WAITING TIME IN LINE.ENTER -88 FOR DO NOT KNOW.  *The same question will be generated by the ODK software for all water sources selected in HQ19* | Minutes | |  | | | |  |
| 26 | Does your family have a garden? | Yes 1  No 0 | | | | | |  |
| 27 | Do members of your household use any of the following toilet facilities?  Flush/pour flush toilets connected to:  Piped sewer system  Septic tank  Elsewhere  Unknown / Not sure / Don’t know  Ventilated improved pit latrine  Pit latrine with slab  Pit latrine without slab  Composting toilet  Bucket toilet  Hanging toilet /Hanging latrine  Other:  READ OUT ALL TYPES AND CHECK ALL THAT ARE USED. |  | | Yes  1  1  1  1  1  1  1  1  1  1  1 | | | No  0  0  0  0  0  0  0  0  0  0  0 |  |
| 26 | What is the main toilet facility used by members of your household?  Flush/pour flush toilets connected to:  Piped sewer system  Septic tank  Elsewhere  Unknown / Not sure / Don’t know  Ventilated improved pit latrine  Pit latrine with slab  Pit latrine without slab  Composting toilet  Bucket toilet  Hanging toilet /Hanging latrine  Other:  READ OUT ALL TYPES AND CHECK THE MAIN FACILITY. MUST BE SELECTED IN HQ 25 | 1  2  3  4  5  6  7  8  9  10 | | | | | |  |
| 27 | QUESTION HQ 27 WILL REPEAT X TIMES, ONCE FOR EACH SANITATION FACILITY SELECTED IN HQ 25. THESE FACILITIES INCLUDE:  *The ODK software will list all sources selected in HQ 25.*  How often does your family typically use [TOILET FACILITY TYPE]?  REGULAR PRACTICES AT THE HOUSEHOLD ONLY  *The same question will be generated by the ODK software for all toilet facility types selected in HQ25* | Always 1  Most of the time 2  Occasionally 3  Rarely 4 | | | | | |  |
| 28 | How many people within your household regularly use the bush / field at home or at work?  THERE ARE X PEOPLE IN THIS HOUSEHOLD. ENTER -88 FOR DO NOT KNOW. | Number of people | |  | | | |  |
| 29 | For all children under age five: what methods, if any, does your household use to dispose of children's waste?  Children use a latrine / toilet  Leave waste where it is  Bury waste in field / yard  Dispose of waste in latrine / toilet  Dispose of waste with rubbish / garbage  Dispose of waste with waste water  Use it as manure  Burn it  Don’t know  *The ODK software will only ask this question to households that listed children under 5 in the household roster (HQ3)* |  | | Yes  1  1  1  1  1  1  1  1  -88 | | | No  0  0  0  0  0  0  0  0 |  |
| Thank the respondent for his/her time.  THE RESPONDENT IS FINISHED, BUT THERE ARE STILL TWO MORE QUESTIONS FOR YOU TO COMPLETE OUTSIDE THE HOUSE. | | | | | | | | |
| LOCATION AND QUESTIONNAIRE RESULT | | | | | | | | |
| P | Take a GPS point outside near the entrance to the household.  Record location when the accuracy is smaller than 6m.  GPS COORDINATES CAN ONLY BE COLLECTED WHEN OUTSIDE. | *Instructions are given directly by the ODK software*  RECORD LOCATION | | | | | |  |
| Qa | Ask permission to take a photo of the entrance of the house.  Did you get consent to take the photo? | Yes 1  No 0 | | | | | | Skip to R if No |
| Qb | Ensure that no people are in the photo | *Instructions are given directly by the ODK software*  TAKE PICTURE  CHOOSE IMAGE | | | | | |  |
| R | Record the result of the Service Delivery Point Survey | Completed 1  No household member at home or no competent respondent at home at time of visit 2  Postponed 3  Refused 4  Partly completed 5  Dwelling vacant or address not a dwelling 6  Dwelling destroyed 7  Dwelling not found 8 | | | | | |  |
