# Supplementary material for: Does proximity of women to facilities with better choice of contraceptives affect their contraceptive utilization in rural Ethiopia?
Source: PLoS One. 2017 Nov 13;12(11):e0187311. doi: 10.1371/journal.pone.0187311 (PMC5683563; doi:10.1371/journal.pone.0187311)
Supplement: S1 File — (ZIP) [file pone.0187311.s004.zip › Questionnaires/English version/PMA2020_SDP_R1.docx]

| **mADDS –Service Delivery Point (SDP) Questionnaire** |
| --- |

| **NO** | **QUESTIONS AND FILTERS** | **CODING CATEGORIES** | | | | | | | | | | | | | | | | | | | | | | | | | | | | | | | | | | **SKIP** |
| --- | --- | --- | --- | --- | --- | --- | --- | --- | --- | --- | --- | --- | --- | --- | --- | --- | --- | --- | --- | --- | --- | --- | --- | --- | --- | --- | --- | --- | --- | --- | --- | --- | --- | --- | --- | --- |
| **IDENTIFICATION**  **Please record the following identifying information prior to beginning the interview.** | | | | | | | | | | | | | | | | | | | | | | | | | | | | | | | | | | | | |
| A | How many times have you visited this service delivery point for this interview? | 1^st^ time 1  2^nd^ time 2  3^rd^ time 3 | | | | | | | | | | | | | | | | | | | | | | | | | | | | | | | | | |  |
| B | Interviewer’s name: Is this your name?  *ODK will display the name associated with the phone’s serial number.*  If not, please record your name: | Yes 1  No 0 | | | | | | | | | | | | | | | | | | | | | | | | | | | | | | | | | |  |
|  |  |  | | | | | | | | | | | | | | | | | | | | | | | | | | | | | | | | | |  |
| C | **CURRENT DATE AND TIME DISPLAYED ON SCREEN.**  Is this date and time correct? | Yes 1  No 0 | | | | | | | | | | | | | | | | | | | | | | | | | | | | | | | | | | Skip to E if Yes |
| D | Record the correct date and time. | Date | | Day | | | | | Month | | | | | | | | | | | | | | | Year | | | | | | | | | | | |  |
|  |  | Time | | Hour | | | | | Min | | | | | | | | | | | | | | | AM/PM | | | | | | | | | | | |  |
| E | Region  **PLEASE SELECT THE NAME OF THE REGION WHERE THE FACILITY IS LOCATED.** | Tigray 1  Afar 2  Amhara 3  Oromia 4  Ethiopia Somali 5  Benishangul Gumuz 6  SNNPR 7  Gambella 8  Harari 9  Addis Ababa 10  Dire Dawa 11 | | | | | | | | | | | | | | | | | | | | | | | | | | | | | | | | | |  |
| F | Zone  **PLEASE RECORD THE NAME OF THE DISTRICT WHERE THE FACILITY IS LOCATED.** | *ODK will populate a list of appropriate localities based on the Zone selected for SQ F* | | | | | | | | | | | | | | | | | | | | | | | | | | | | | | | | | |  |
| G | Woreda/District  **PLEASE RECORD THE NAME OF THE DISTRICT WHERE THE FACILITY IS LOCATED.** | *ODK will populate a list of appropriate district based on the region selected for SQ E* | | | | | | | | | | | | | | | | | | | | | | | | | | | | | | | | | |  |
| H | Kebele/Locality name  **PLEASE RECORD THE NAME OF THE LOCALITY WHERE THE FACILITY IS LOCATED.** | *ODK will populate a list of appropriate localities based on the district selected for SQ F* | | | | | | | | | | | | | | | | | | | | | | | | | | | | | | | | | |  |
| I | Enumeration area  **PLEASE RECORD THE NUMBER OF THE ENUMERATION AREA WHERE THE FACILITY IS LOCATED OR TO WHICH IT IS ASSIGNED.** |  | | | | | | | | | | | | | | | | | | | | | | | | | | | | | | | | | |  |
| J | Facility number  **PLEASE RECORD THE NUMBER OF THE FACILITY FROM THE LISTING FORM.** |  | | | | | | | | | | | | | | | | | | | | | | | | | | | | | | | | | |  |
| K | Type of facility  **PLEASE SELECT THE TYPE OF FACILITY.** | Hospital 1  Health center 2  Health Post 3  Health Clinic 4  Pharmacy 5  Drug shop 6  Other 7 | | | | | | | | | | | | | | | | | | | | | | | | | | | | | | | | | |  |
| L | Managing authority  **PLEASE SELECT THE MANAGING AUTHORITY FOR THE FACILITY.** | Government 1  NGO 2  Faith-based organization 3  Private 4  Other 5 | | | | | | | | | | | | | | | | | | | | | | | | | | | | | | | | | |  |
| M | Is a competent respondent present and available to be interviewed today? | Yes 1  No 0 | | | | | | | | | | | | | | | | | | | | | | | | | | | | | | | | | | Skip to S if No |
| **INFORMED CONSENT**  **Find the competent respondent responsible for patient services (main administrator and family planning in-charge) who is present at the facility. Read the following greeting:** | | | | | | | | | | | | | | | | | | | | | | | | | | | | | | | | | | | | |
| Hello. My name is ______________________________. We are here on behalf of the Addis Ababa University, and Federal Ministry of Health to assist the government and communities in knowing more about health services. Now I will read a statement explaining the survey.  Your facility was randomly selected to participate in this study. We will be asking you questions about family planning and other reproductive health services and will ask to see patient registers. No patient names from the registers will be reviewed, recorded or shared. The information about your facility may be used by health organizations for planning service improvements or further studies of health services. The data collected from your facility will also be used by researchers for analyses. However, the name of your facility will not be provided, and any reports by researchers who use your facility data will only present information in aggregate form so that your facility cannot be identified.  We are asking for your help to ensure that the information we collect is accurate. If there are questions for which someone else is the most appropriate person to provide the information, we would appreciate your introducing us to that person.  You may refuse to answer any question or choose to stop the interview at any time. Do you have any questions about the survey? | | | | | | | | | | | | | | | | | | | | | | | | | | | | | | | | | | | | |
| M | Provide a paper copy of the Consent Form to the respondent and explain it. Then, ask: May I begin the interview now? | Yes 1  No 0 | | | | | | | | | | | | | | | | | | | | | | | | | | | | | | | | | | Skip to R if No |
| N | Respondent’s signature  **PLEASE ASK THE RESPONDENT TO SIGN OR CHECK THE BOX IN AGREEMENT OF THEIR PARTICIPATION.** | GATHER SIGNATURE:  Checkbox: ☐ | | | | | | | | | | | | | | | | | | | | | | | | | | | | | | | | | |  |
| O | Interviewer’s name  **PLEASE RECORD YOUR NAME AS A WITNESS TO THE CONSENT PROCESS.** |  | | | | | | | | | | | | | | | | | | | | | | | | | | | | | | | | | |  |
| P | Name of the facility  **PLEASE RECORD THE NAME OF THE FACILITY.** |  | | | | | | | | | | | | | | | | | | | | | | | | | | | | | | | | | |  |
| Q | What is your position in this facility?  **SELECT THE HIGHEST MANAGERIAL QUALIFICATION OF THE RESPONDENT.** | Owner 1  In-charge / manager 2  Staff 3 | | | | | | | | | | | | | | | | | | | | | | | | | | | | | | | | | |  |
| **NO** | **QUESTIONS AND FILTERS** | **CODING CATEGORIES** | | | | | | | | | | | | | | | | | | | | | | | | | | | | | | | | | | **SKIP** |
| **Section 1 – Information about services**  **Now I would like to ask about the services provided at this facility** | | | | | | | | | | | | | | | | | | | | | | | | | | | | | | | | | | | | |
| 1 | What year did this facility first begin offering health services / products?  **ENTER 2020 FOR DO NOT KNOW.** | Year | | | | |  | | | | | | | | | | | | | | | | | | | | | | | | | | | | |  |
| 2 | How many days each week is the facility routinely open?  **NUMBER MUST BE BETWEEN 1 AND 7. ENTER -88 FOR DO NOT KNOW.** | Number of days | | | | |  | | | | | | | | | | | | | | | | | | | | | | | | | | | | |  |
| 3 | **Now I have some questions about staffing for this facility.**  For the following questions, please tell me how many staff with this qualification are currently assigned to this facility.  Finally, tell me the total number present at any time today.  We want to know the highest technical qualification that any staff may hold regardless of the person’s actual assignment or specialist studies.  **ENTER -88 FOR DO NOT KNOW AND -77 FOR NOT APPLICABLE. 0 IS A POSSIBLE ANSWER.** | Doctor  Nurse/midwife  Health Officer  Pharmacist  Health Extension Workers  Other medical staff | | | | | | | | | | | | | Actual number  __  __  __  __  __  __ | | | | | | | | | | | | | Present today  __  __  __  __  __  __ | | | | | | | |  |
|  | **CHECK J:** type of facility? | Hospital 1  Health center 2  Health Post 3  Health Clinic 4  Pharmacy 5  Drug shop 6  Other 7 | | | | | | | | | | | | | | | | | | | | | | | | | | | | | | | | | | Skip to 8 if I: 5, 6 or 7 |
| 4 | Is there a healthcare worker present at the facility at all times or officially on call for the facility at all times (24 hours a day) for emergencies? | Yes, 24-hr staff 1  No, no 24-hr staff 0 | | | | | | | | | | | | | | | | | | | | | | | | | | | | | | | | | |  |
| 5 | Do you have an estimate of the size of the catchment population that this facility serves that is, the target, or total population living in the area served by this facility? | No catchment area 1  Yes, knows size of catchment area 2  Doesn’t know size of catchment area 3 | | | | | | | | | | | | | | | | | | | | | | | | | | | | | | | | | | Skip to 7 if No or DK |
| 6 | What is the size of the catchment population?  **RECORD THE NUMBER OF PEOPLE LIVING IN THE AREA SERVED BY THIS FACILITY.** | Number of people | | | | |  | | | | | | | | | | | | | | | | | | | | | | | | | | | | |  |
| 7 | How many beds does the facility have?  **0 IS A POSSIBLE ANSWER. ENTER -88 FOR DO NOT KNOW.** | Number of beds | | | | |  | | | | | | | | | | | | | | | | | | | | | | | | | | | | |  |
| 8 | When was the last time an owner / supervisor from outside this facility came here to visit? | Never external supervision 0  Within the past 6 months 1  More than 6 months ago 2  Don’t know -88 | | | | | | | | | | | | | | | | | | | | | | | | | | | | | | | | | |  |
| 9 | Does this facility have electricity today? | Yes 1  No 0 | | | | | | | | | | | | | | | | | | | | | | | | | | | | | | | | | |  |
| 10 | Does this facility have water today? | Yes 1  No 0 | | | | | | | | | | | | | | | | | | | | | | | | | | | | | | | | | |  |
|  | **CHECK J:** type of facility? | Hospital 1  Health center 2  Health Post 3  Health Clinic 4  Pharmacy 5  Drug shop 6  Other 7 | | | | | | | | | | | | | | | | | | | | | | | | | | | | | | | | | | Skip to 13 if I: 5, 6 or 7 |
| 11 | How many hand washing facilities are available on site for staff to use?  **ENTER -88 FOR DO NOT KNOW.** | Number of facilities | | | | | | | |  | | | | | | | | | | | | | | | | | | | | | | | | | | Skip to 13 if 0 |
| 12 | Ask to see the nearest hand washing facility. At the hand washing facility OBSERVE:  Soap is present  Water source is present: stored water  Water source is present: tap water  Hand washing area is near a sanitation facility  None of the above  Did not see the facility  **SELECT ALL THAT APPLY** |  | | | | | Yes  1  1  1  1  -88  1 | | | | | | | | | | | | | | | No  0  0  0  0  0 | | | | | | | | | | | | | |  |
| 13 | Does the facility have a functioning computer?  **NO NEED TO OBSERVE** | Yes 1  No 0 | | | | | | | | | | | | | | | | | | | | | | | | | | | | | | | | | |  |
|  | **CHECK J:** type of facility? | Hospital 1  Health center 2  Health Post 3  Health Clinic 4  Pharmacy 5  Drg shop 6  Other 7 | | | | | | | | | | | | | | | | | | | | | | | | | | | | | | | | | | Skip to 15 if I: 5, 6 or 7 |
| 14 | How does this facility finally dispose of sharp items or filled sharps boxes? | Never have sharps waste 0  Burn in incinerator 1  Open Burning 2  Dump without burning 3  Remove offsite 4  Other 5 | | | | | | | | | | | | | | | | | | | | | | | | | | | | | | | | | |  |
| **Section 2 – Family Planning Services**  **Now I would like to ask about family planning services provided at this facility.** | | | | | | | | | | | | | | | | | | | | | | | | | | | | | | | | | | | | |
| 15 | Do you usually offer family planning services / products? | Yes 1  No 0 | | | | | | | | | | | | | | | | | | | | | | | | | | | | | | | | | | Skip to 19 if No |
| 16 | What year did this facility first begin offering family planning services / products?  **ENTER 2020 FOR DO NOT KNOW.** | Year | | | | | | | |  | | | | | | | | | | | | | | | | | | | | | | | | | |  |
| 17 | How many days in a week are family planning services / products offered / sold here?  **USE A 7-DAY WEEK TO CALCULATE NUMBER OF DAYS. ENTER A NUMBER BETWEEN 1 AND 7. ENTER -88 FOR DO NOT KNOW.** | Number of days | | | | | | | |  | | | | | | | | | | | | | | | | | | | | | | | | | |  |
| 18 | Are family planning services / products offered here today? | Yes 1  No 0 | | | | | | | | | | | | | | | | | | | | | | | | | | | | | | | | | |  |
|  | **CHECK J:** type of facility? | Hospital 1  Health center 2  Health Post 3  Health Clininc 4  Pharmacy 5  Drug shop 6Other 8 | | | | | | | | | | | | | | | | | | | | | | | | | | | | | | | | | | Skip to 23 if I: 5, 6 or 7 |
| 19 | Does this facility provide family planning supervision, support, or supplies to community health volunteers? | Yes 1  No 0 | | | | | | | | | | | | | | | | | | | | | | | | | | | | | | | | | | Skip to 22 if No |
| 20 | How many community health volunteers are supported by this facility?  **ENTER -88 FOR DO NOT KNOW.** | Number of CHWs | | | | | | | | |  | | | | | | | | | | | | | | | | | | | | | | | | |  |
| 21 | Do the community health volunteers provide any of the following contraceptives:  Condoms  Pills  Injectables |  | | | | | | | | | | | | | | | Yes  1  1  1 | | | | | | | | | | | | | No  0  0  0 | | | | | |  |
| 22 | How many times in the last 12 months has a mobile outreach team visited your facility to deliver supplementary/additional family planning services? | Number of times: | | | | | | | | | | | | | | |  | | | | | | | | | | | | | | | | | | |  |
|  | **CHECK 15:** Offer FP services/products? | Yes 1  No 0 | | | | | | | | | | | | | | | | | | | | | | | | | | | | | | | | | | Skip to 25 if No |
| 23 | Does this facility have any routine user-fees or charges for any services related to family planning?  **THIS INCLUDES ANY FEES, INCLUDING THOSE FOR REGISTRATION OR FOR CLIENT HEALTH RECORDS.** | Yes 1  No 0 | | | | | | | | | | | | | | | | | | | | | | | | | | | | | | | | | | Skip to 25 if No |
| 24 | Are the official fees posted so that the client can easily see them?  **IF YES, POSTED FEES MUST BE OBSERVED.** | Yes, all fees are posted 1  Yes, some, not all fees posted 2  No posted fees 0 | | | | | | | | | | | | | | | | | | | | | | | | | | | | | | | | | |  |
| 25 | Do you collect information about clients’ opinion in any of the following ways?  Suggestion box  Client survey form  Client interview form  Official meeting with community leaders  Informal discussion with client or community  Direct client feedback to staff  Other  Don’t know  None of the above  **SELECT ALL METHODS** |  | | | | | | | | | | | | | | | Yes  1  1  1  1  1  1  1  -88  1 | | | | | | | | | | | | | No  0  0  0  0  0  0  0  0 | | | | | | Skip to 29 if “None of the above” is selected |
| 26 | Is there a procedure for reviewing or reporting on clients’ opinions? | Yes 1  No 0 | | | | | | | | | | | | | | | | | | | | | | | | | | | | | | | | | | Skip to 28 if No |
| 27 | Ask to see a report or form on which data are compiled or discussion is reported | Report seen 1  Report not seen 2 | | | | | | | | | | | | | | | | | | | | | | | | | | | | | | | | | |  |
| 28 | In the past 12 months, have any changes been made in the program as a result of client opinion?  **IF YES, INDICATE IF THE CHANGE(S) ARE RELATED TO ANY OF THE LISTED TOPICS.** | No 0  Yes, change in services or times offered or way services are provided 1  Yes, change for client comfort 2  Other 3  Don’t know -88 | | | | | | | | | | | | | | | | | | | | | | | | | | | | | | | | | |  |
| 29 | In the past 12 months, have there been any meetings where service statistics (or inventory) for family planning are discussed with staff? | Yes 1  No 0 | | | | | | | | | | | | | | | | | | | | | | | | | | | | | | | | | |  |
| 30 | Do you use any of the following to review service data for monitoring and evaluation?  Wall chart / graph  Written report / minutes  Other  Nothing observed  **ASK TO SEE ANY REPORTS, WALL GRAPHS OR CHARTS THAT SHOW SERVICE DATA HAS BEEN REVIEWED. SELECT ALL RELEVANT TYPES OF DOCUMENTATION OBSERVED.** |  | | | | | | | | | | | | | | | | Yes  1  1  1  1 | | | | | | | | | | | | | No  0  0  0  0 | | | | |  |
|  | **CHECK 15:** Offer FP services/products? | Yes 1  No 0 | | | | | | | | | | | | | | | | | | | | | | | | | | | | | | | | | | Skip to 40 if No |
| 31 | Which of the following methods of contraception are counseled, provided, or prescribed / referred?  Do you charge for any of these methods?  Female sterilization  Male sterilization  IUD  Progestin Only Pill  Injectables  Implants  Pill  Male condom  Female condom  Emergency Contraception  Standard Days/Cycle beads  LAM  Rhythm method  Withdrawal  Cou: Counseled; Pro: Provided; Pre: Prescribed / Referred ; Chg: charge  **ALL OPTIONS SHOULD BE READ ALOUD** | Cou  Yes  1  1  1  1  1  1  1  1  1  1  1  1  1  1  1 | Cou  No  0  0  0  0  0  0  0  0  0  0  0  0  0  0  0 | | Pro  Yes  1  1  1  1  1  1  1  1  1  1  1  1 | | | Pro  No  0  0  0  0  0  0  0  0  0  0  0  0 | | | | | Pre  Yes  1  1  1  1  1  1  1  1  1  1  1  1 | | | | | | | | | | Pre  No  0  0  0  0  0  0  0  0  0  0  0  0 | | | | | | | | | | | | Chg  Yes  1  1  1  1  1  1  1  1  1  1  1  1 | Skip to 33 if no charges |
| 32 | How much do you charge for one unit of each method that you provide?  Fem. sterilization  Male sterilization  IUD  Progestin Only Pill  Injectables – 3 months  Implants  Pill  Male condom  Female condom  Emergency Contraception  Standard Days/ Cycle beads  **ENTER ALL PRICES IN NEW CURRENCY AND CEDIS (NO PESEWA).**  *ODK will only display the methods for which the facility charges from SQ 31* | Amount per unit | | | | | | | | | | | | | | | | | | | | | _________  ____________________________________________________________________________________________________________ | | | | | | | | | | | | |  |
|  | **CHECK J:** type of facility? | Hospital 1  Health center 2  Health Post 3  Health Clinic 4  Pharmacy 5  Drug shop 6  Other 8 | | | | | | | | | | | | | | | | | | | | | | | | | | | | | | | | | | Skip to 39b if I: 5, 6 or 7 |
|  | **CHECK 31:** Are implants provided? | Yes 1  No 0 | | | | | | | | | | | | | | | | | | | | | | | | | | | | | | | | | | Skip to 35 if No |
| 33 | On days when you offer family planning services, does this facility have trained personnel able to insert implants? | Yes 1  No 0 | | | | | | | | | | | | | | | | | | | | | | | | | | | | | | | | | |  |
| 34 | On days when you offer family planning services, does this facility have trained personnel able to remove implants? | Yes 1  No 0 | | | | | | | | | | | | | | | | | | | | | | | | | | | | | | | | | |  |
|  | **CHECK 31:** Are IUDs provided? | Yes 1  No 0 | | | | | | | | | | | | | | | | | | | | | | | | | | | | | | | | | | Skip to 37 if No |
| 35 | On days when you offer family planning services, does this facility have trained personnel able to insert IUDs? | Yes 1  No 0 | | | | | | | | | | | | | | | | | | | | | | | | | | | | | | | | | |  |
| 36 | On days when you offer family planning services, does this facility have trained personnel able to remove IUDs? | Yes 1  No 0 | | | | | | | | | | | | | | | | | | | | | | | | | | | | | | | | | |  |
|  | **CHECK 31:** Are implants provided? | Yes 1  No 0 | | | | | | | | | | | | | | | | | | | | | | | | | | | | | | | | | | Skip to 38 if No |
| 37 | Does this facility have the following supplies needed to insert and/or remove implants:  Clean Gloves  Antiseptic  Sterile Gauze Pad or Cotton Wool  Local Anesthetic  Sealed Implant Pack  Blade  **READ OUT ALL SUPPLIES AND SELECT ALL THAT APPLY. SUPPLIES DO NOT NEED TO BE OBSERVED.** |  | | | | | | | | | | | | | | Yes  1  1  1  1  1  1 | | | | | | | | | | | | | No  0  0  0  0  0  0 | | | | | | |  |
|  | **CHECK 31:** Are IUDs provided? | Yes 1  No 0 | | | | | | | | | | | | | | | | | | | | | | | | | | | | | | | | | | Skip to 39 if No |
| 38 | Does this facility have the following supplies needed to insert and/or remove IUDs:  Sponge-holding forceps  Speculums (large and medium)  Tenaculum  Clamp  **READ OUT ALL SUPPLIES AND SELECT ALL THAT APPLY. SUPPLIES DO NOT NEED TO BE OBSERVED.** |  | | | | | | | | | | | | | | | Yes  1  1  1  1 | | | | | | | | | | | | No  0  0  0  0 | | | | | | |  |
|  | **CHECK J:** type of facility? | Hospital 1  Health center 2  Health Post 3  Health Clinic 4  Pharmacy 5  Drug shop 6  Other 7 | | | | | | | | | | | | | | | | | | | | | | | | | | | | | | | | | | 39a if: 1-4,  39b if: 5, 6 or 7 |
| 39a | **FROM FAMILY PLANNING REGISTER, RECORD:**  **(1)** the total number of family planning visits (new and continuing) in the last completed month, for each method.  **(2)** the number of new clients who received family planning services in the last completed month, for each method. | Fem. sterilization  Male sterilization  IUD  Progestin Only Pill  Injectables – 3 months  Implants  Pill  Male condom  Female condom  Emergency contraception  Standard Days/ Cycle beads | | | | | | | | | | | | Total # of visits  ___  ___  ___  ___  ___  ___  ___  ___  ___  ___  ___  ___  ___ | | | | | | | | | | | | | # of new clients  ___  ___  ___  ___  ___  ___  ___  ___  ___  ___  ___ | | | | | | | | |  |
| 39b | **FROM FAMILY PLANNING RECORD BOOK, RECORD:**  The total number of family planning products sold in the last completed month, for each method. | IUD  Progestin Only Pill  Injectables – 3 months  Implants  Pill  Male condom  Female condom  Emergency contraception  Standard Days / Cycle beads  Other traditional methods | | | | | | | | | | | | | | | | | | | | | | | # of products sold  ___  ___  ___  ___  ___  ___  ___  ___  ___  ___  ___  ___ | | | | | | | | | | |  |
|  | **CHECK J:** type of facility? | Hospital 1  Health center 2  Health Post 3  Health Clinic 4  Pharmacy 5  Drug shop 6  Other 7 | | | | | | | | | | | | | | | | | | | | | | | | | | | | | | | | | | Skip to 45 if I: 5, 6 or 7 |
| 40 | Which of the following services are provided at this facility:  Antenatal  Delivery  Postnatal  Post-abortion  **READ ALL OPTIONS AND SELECT ALL THAT APPLY.** |  | | | | | | | | | | | | | | | | | | | Yes  1  1  1  1 | | | | | | | | | | | | No  0  0  0  0 | | | Skip to 45 if No to postnatal and post-abortion  Skip to 43 if no to postnatal and yes to post-abortion |
| 41 | Which of the following is discussed with the mother before she leaves the facility with the newborn after delivery:  Diet, nutrition, and exercises  Postpartum mental health  Return to fertility  Healthy timing and spacing of pregnancies  Advice on family planning methods:  Lactational Amenorrhea Method  Long-acting methods  Spacing methods  None of the above  **READ ALL OPTIONS AND SELECT ALL THAT APPLY.** |  | | | | | | | | | | | | | | | | | | Yes  1  1  1  1  1  1  1  1 | | | | | | | | | | | | No  0  0  0  0  0  0  0  0 | | | |  |
| 42 | Is the woman offered a method of family planning during the postnatal visit? | Yes 1  No 0 | | | | | | | | | | | | | | | | | | | | | | | | | | | | | | | | | |  |
|  | **CHECK 40:** Are post-abortion services offered? | Yes 1  No 0 | | | | | | | | | | | | | | | | | | | | | | | | | | | | | | | | | | Skip to 45 if No |
| 43 | During post-abortion visits, which of the following is discussed with the client:  Post-abortion mental health  Return to fertility  Healthy timing and spacing of pregnancies  Advice on family planning methods:  Long-acting methods  Spacing methods  None of the above  **READ ALL OPTIONS AND SELECT ALL THAT APPLY.** |  | | | | | | | | | | | | | | | | | | Yes  1  1  1  1  1  1 | | | | | | | | | | | | No  0  0  0  0  0  0 | | | |  |
| 44 | Is the woman offered a method of family planning during the post-abortion visit? | Yes 1  No 0 | | | | | | | | | | | | | | | | | | | | | | | | | | | | | | | | | |  |
| 45 | Which of the following family planning services do you offer to unmarried adolescents?  Counsel for contraceptive methods  Provide contraceptive methods  Prescribe / refer contraceptive methods  None of the above  **READ ALL OPTIONS AND SELECT ALL THAT APPLY** |  | | | | | | | | | | | | | | | | | Yes  1  1  1  1 | | | | | | | | | | | | | | No  0  0  0  0 | | |  |
| 46 | Does this facility offer any service related to diagnosis, treatment, or supportive services for STIs? | Yes 1  No 0 | | | | | | | | | | | | | | | | | | | | | | | | | | | | | | | | | |  |
| 47 | Does this facility offer any service related to diagnosis, treatment, or supportive services for HIV? | Yes 1  No 0 | | | | | | | | | | | | | | | | | | | | | | | | | | | | | | | | | | Skip to 50 if No |
|  | **CHECK J:** type of facility? | Hospital 1  Health center 2  Health Post 3  Health Clinic 4  Pharmacy 5  Drug shop 6  Other 7 | | | | | | | | | | | | | | | | | | | | | | | | | | | | | | | | | | Skip to 52 if I: 5, 6 or 7 |
| 48 | Which of the following family planning services do you offer to clients who come in for HIV services:  Counsel for contraceptive methods?  Provide contraceptive methods?  Prescribe / refer contraceptive methods?  None of the above  **SELECT ALL THAT APPLY** |  | | | | | | | | | | | | | | | Yes  1  1  1  1 | | | | | | | | | | | | | No  0  0  0  0 | | | | | |  |
| 49 | During an HIV consultation does the provider:  ask the client about reproductive intentions?  discuss the FP method preferred by the client?  discuss dual method use?  provide condoms?  discuss instructions and side effects of chosen FP method?  offer an FP method? |  | | | | | | | | | Yes  1  1  1  1  1  1 | | | | | | No  0  0  0  0  0  0 | | | | | | | | | | | | | DK  -88  -88  -88  -88  -88  -88 | | | | | |  |
|  | **CHECK 15:** Offer FP services/products? | Yes 1  No 0 | | | | | | | | | | | | | | | | | | | | | | | | | | | | | | | | | | Skip to R if No |
| 50 | **ASK TO SEE THE ROOM WHERE EXAMINATIONS FOR FAMILY PLANNING ARE CONDUCTED**  **FOR EACH OF THE FOLLOWING ITEMS, CHECK TO SEE WHETHER ITEM IS EITHER IN ROOM WHERE EXAMINATIONS ARE CONDUCTED OR IN AN ADJACENT ROOM.**  [**OBSERVED ITEMS FOR INFECTION CONTROL**]  O: Observed; RU: Reported, Unseen; NA: Not Available | Running water (piped)  Other running water (bucket with tap or pour pitcher)  Water in bucket or basin (water reused)  Hand-washing soap  Single-use hand drying towels  Waste receptacle with lid and plastic liner  Sharps container  Disposable latex gloves  Disinfectant  Disposable needles and syringes  Auditory privacy  Visual privacy  Examination table  Client educational materials on FP | | | | | | | | | | | O  1  1  1  1  1  1  1  1  1  1  1  1  1  1 | | | | | | | | | | RU  2  2  2  2  2  2  2  2  2  2  2  2  2  2 | | | | | | | | | | | | NA  -77  -77  -77  -77  -77  -77  -77  -77  -77  -77  -77  -77  -77  -77 |  |
| 51 | **ASSESS CONDITION OF FAMILY PLANNING SERVICE AREA** | Floor: swept, no obvious dirt or waste  Counters/Tables/Chairs: wiped clean, no obvious dirt or waste  No Broken equipment, papers, boxes around making area cluttered and dirty  Walls: reasonably clean  Doors: no or minor damage  Walls: no or minor damage  Roof: no or minor damages | | | | | | | | | | | | | | | | | | | | Yes  1  1  1  1  1  1  1 | | | | | | | | | | | | No  0  0  0  0  0  0  0 | |  |
| 52 | You said you provide the following methods. Can you show them to me? For all observed methods: have any been out of stock in the last 12 months?  IUD  Progestin Only Pill  Injectables – 3 months  Implants  Pill  Male condom  Female condom  Emergency Contraception  Standard Days / Cycle beads  O: In-stock Observed;  N.O.: In-stock - Not Observed;  OOS last 12 mo.: Out of stock in last 12 months  **SELECT OOS <12 MO IF THE METHOD HAS EVER BEEN OUT OF STOCK IN THE PAST 12 MONTHS, EVEN IF IT IS IN STOCK ON THE DAY OF THE INTERVIEW.**  **IF N.O. IS CHECKED, OOS ALSO NEEDS TO BE CHECKED; CANNOT CHECK BOTH O AND N.O.; EITHER O. OR N.O. MUST BE CHECKED.**  *ODK will only display the methods which are provided at the facility from SQ 31* |  | | | | O  1  1  1  1  1  1  1  1  1 | | | | | | N.O.  0  0  0  0  0  0  0  0  0 | | | | | | | | | | | | | | OOS in last 12  mos  1  1  1  1  1  1  1  1  1 | | | | | | | | | |  |
| 53 | **FOR FQ53-56, OBSERVE THE PLACE WHERE CONTRACEPTIVE SUPPLIES ARE STORED AND REPORT ON THE FOLLOWING CONDITION:**  Are all the methods off the floor? | Yes 1  No 0 | | | | | | | | | | | | | | | | | | | | | | | | | | | | | | | | | |  |
| 54 | Are all the methods protected from water? | Yes 1  No 0 | | | | | | | | | | | | | | | | | | | | | | | | | | | | | | | | | |  |
| 55 | Are all the methods protected from the sun? | Yes 1  No 0 | | | | | | | | | | | | | | | | | | | | | | | | | | | | | | | | | |  |
| 56 | Is the room clean of evidence of rodents (bats, rats) or pests (roaches…)? | Yes 1  No 0 | | | | | | | | | | | | | | | | | | | | | | | | | | | | | | | | | |  |

| Thank the respondent for his / her time.  **THE RESPONDENT IS FINISHED, BUT THERE ARE STILL 3 MORE QUESTIONS FOR YOU TO COMPLETE OUTSIDE THE FACILITY.** | | | |
| --- | --- | --- | --- |
| **LOCATION AND QUESTIONNAIRE RESULT** | | | |
| R | Take a GPS point outside near the entrance to the facility.  Record location when the accuracy is smaller than 6m.  **GPS COORDINATES CAN ONLY BE COLLECTED WHEN OUTSIDE.** | *Instructions are given directly by the ODK software:*  RECORD LOCATION |  |
| S a | Ask permission to take a photo ofthe entrance of the facility  Did you get consent to take the photo? | Yes 1  No 0 | Skip to T if No |
| S b | Ensure that no people are in the photo | *Instructions are given directly by the ODK software*  TAKE PICTURE  CHOOSE IMAGE |  |
| T | Record the result of the Service Delivery Point Survey | Completed 1  Not at facility 2  Postponed 3  Refused 4  Partly completed 5  Other 6 |  |
