# Supplementary material for: Does proximity of women to facilities with better choice of contraceptives affect their contraceptive utilization in rural Ethiopia?
Source: PLoS One. 2017 Nov 13;12(11):e0187311. doi: 10.1371/journal.pone.0187311 (PMC5683563; doi:10.1371/journal.pone.0187311)
Supplement: S1 File — (ZIP) [file pone.0187311.s004.zip › Questionnaires/Amharic version/FQ_Amharic_14 Nov 13.doc]

**mADDS –የሴት መልስ ሰጭዎች መጠይቅ**

| **ተራ ቁ** | **ጥያቄዎች እና ማጣሪያዎች** | | | | | | **የመለስ አማራጮች ኮድ** | | | | | | | | | | | | | | **እለፊ** |
| --- | --- | --- | --- | --- | --- | --- | --- | --- | --- | --- | --- | --- | --- | --- | --- | --- | --- | --- | --- | --- | --- |
| **መለያ**  **ቃለ መጠይቅ ከመጀመርሽ በፊት እባካችሽን የሚከተሉትን የመለያ መረጃዎች መዝግቢ፡፡** | | | | | | | | | | | | | | | | | | | | | |
| A | | አሁን ያለሺው ትክክለኛው ቤተሰብ ጋር ነው?  ይህ ፎቶ በቤተሰብ ምዝገባ ወቅት የተነሳ የቤቱ የፊተኛው ገፅታ ነው።  *በቤተሰብ ምዝገባ ወቅት የተነሳውን ፎቶ ከዜህ የሴቶች መጠይቅ ጋር በማያያዝ ODK ያሳየናል፡፡* | | | | | አዎ 1  የለም 0 | | | | | | | | | | | | | |  |
| B | | መልስ ሰጭዋን ቃለ መጠይቅ ለማድረግ ይህንን ቤተሰብ ስንት ጊዜ ጎበኘሺው? | | | | | ለመጀመሪያ ጊዜ 1  ለ2ኛ ጊዜ 2  ለ3ኛጊዜ 3 | | | | | | | | | | | | | |  |
| C | | የመረጃ ሰብሳቢ ስም: ይህ የአንቺ ስም ነው?  ካልሆነ ፣ስምሽን መዝግቢ:  *ODK ከስልክ ቁጥሩ ጋር የተያያዘውን ስም ያሳያል፡፡* | | | | | አዎ 1  የለም 0 | | | | | | | | | | | | | |  |
|  |  |  |  |  |  |  |  | | | | | | | | | | | | | |  |
| D | | **በስክሪኑ ላይ አሁን ያለው ትክክለኛ ቀን እና ሰዓት ይታያል፡፡**  ይህ ቀንና ሰዓት ትክክል ነው? | | | | | አዎ 1  የለም 0 | | | | | | | | | | | | | | አዎ ከሆነ ወደ F እለፊ |
| E | | ትክክለኛውን ቀንና ሰዓት መዝግቢ፡፡ | | | | | የቀኑ ቁጥር | የትኛው ቀን | | | ወር | | | | | ዓ.ም | | | | |  |
|  |  |  |  |  |  |  | ጊዜ1””ሳ” | ሰዓት | | | ደቂቃ | | | | | ጥዋት/ከሰዓት | | | | |  |
| F | | የሚከተሉትን መረጃዎች ከቤተሰብ ምዝገባ የተገኙ ናቸው፡፡ ቃለ መጠይቅ የምታደርጉት ትክክለኛው መልስ ሰጭ መሆኑን አረጋግጪ፡፡  *በቤተሰብ መመዝገቢያ ቅፅ ላይ የሚገኘውን መልስ ሰጭዋ የምትገኝበት መለያ ፣ ይኸውም ክልል፣ዞን፣ወረዳ፣አካባቢ ስም፣የቆጠራ ቦታ፣የቤት ቁጥር፣ የቤተሰብ ተራ ቁጥር፣ ከሴቶች መጠይቅ ጋር በማያያዝ ODK ያሳይሻል፡፡* | | | | |  | | | | | | | | | | | | | |  |
| G | | ከመልስ ሰጭዋ ጋር ምን ያህል ነው የምትተዋወቁት? | | | | | በጣም እንተዋወቃለን 1  በመጠኑ እንተዋወቃለን 2  በደንብ አንተዋወቅም 3  አንተዋወቅም 4 | | | | | | | | | | | | | |  |
| H | | መልስ ሰጭዋ ቃለመጠይቅ ለመደረግ በዕለቱ ተገኝተዋል? | | | | | አዎ 1  የለም 0 | | | | | | | | | | | | | | የለም ከመለሱ ወደ ጥ-M እለፊ |
| **የስምምነት ማረጋገጫ**  **ለሴቶች መጠይቅ ቃለመጠየቅ የሚደረግላጨው ዕድሜያቸው ከ15-49 የሆኑ ሴት መኖራቸውን አረጋግጡ፡፡. ቃለመጠይቁ ገለል ያለ ቦታ ወይም ማንም ሰው በማይሰማበት ቦታ ጠጠየቅ ይኖርበታል፡፡የሚከተለውን የሰላምታ/የመግቢያ አንቀጽ አንብቡ፡፡** | | | | | | | | | | | | | | | | | | | | | |
| እንደምን ዋላችሁ/አደራችሁ. እኔ ስሜ __________ ይባላል፡፡ ለአዲስ አበባ ዩኒቨርስቲ እና ለጤና ጥበቃ ሚኒስቴር እየሰራሁ እገኛለሁ፡፡ እኛ በመላው ሀገር ውስጥ ሴቶችን የሚመለከቱ የስነ ተዋልዶ እና ጤና ጉዳዮች ጥናት እያካሄድን እንገኛለን፡፡ የእርስዎን በዚህ ጥናት መሳተፍዎ እጅግ እናመሰግናለን። ይህ መረጃ መንግስት በጤና አገልግሎት ላይ በተሸለ ሁኔታ እንዲያቅድ ለማሳወቅ ይረዳናል፡፡ ቃለመጠይቁን ለማጠናቀቅ በአብዛኛው ከ15 እስከ 20 ደቂቃ ይወስዳል፡፡ እርስዎ የሚሰጡት ማንኛውም መረጃዎች/መልሶች በሙሉ በሚስጢር የሚያዙ ሲሆን ከጥናቱ አባላት በስተቀር በማንም በሌላ ወገን አይታዩም፡፡  በዚህ ጥናት ላይ የሚሳተፉት በፈቃደኝነት ሲሆን መልስ ለመስጠት የማይፈልጉት ጥያቄ ከጠየኮት ያሰውቁኝና እኔም ወደሚቀጥለው ጥያቄ አልፋለሁ ወይም እርስዎ ከፈለጉ በየትኛውም ጊዜ ቢሆን ቃለመጠይቁን ሊያቋርጡ ይችላሉ፡፡ ነገር ግን የእርስዎ በዚህ ጥናት መሳተፍና የእርስዎን አስተያየት ማግኘት ጠቃሚ ስለሆነ በጥናቱ ለመሳተፍ እና ለጥያቄዎቹ መልስ ለመስጠትም እንደሚስማሙ ተስፋ አደርጋለሁ፡፡ በዚህ ጥናት ላይ በመሳተፍዎ ግዜዎን ከመስጠት ውጭ ሌላ የሚጎዳዎት ነገር የለም። በተጨማሪ ከኛ ጋር ለሚያሳልፉት ግዜ ማካካሻ የሚሆን ___________ እንሰጦታለን።  **አሁን ስለ ጥናቱ ሊጠይቁኝ የሚፈልጉት ነገር አለ?**  **መረጃ ሰብሳቢ፡** አዎ ካሉ ለጥያቄያቸው በቂ ማብራርያ በመስጠት ወደ ሚቀጥለው እለፊ። | | | | | | | | | | | | | | | | | | | | | |
| I | | የጥናት አድራጊው አድራሻ የያዘው ፎርም አንድ ኮፒ ለመልስ ሰጭዋ በመስጠት የስምምነት አላማ አስረዷቸው፡፡ በመቀጠልም ፣ ቃለ መጠይቁን መጀመር እችላለሁ ብላችሁ ጠይቁ፡፡ | | | | | አዎ 1  የለም 0 | | | | | | | | | | | | | የለም ከመለሱ ወደ ጥያቄ M እለፊ | |
| J | | **መልስ ሰጭው/ዋ በጥናቱ ለመሳተፍ መስማማታቸውን ለማረጋገጥ በማጣሪያ ሳጥን ውስጥ ምልክት አድርጊ፡፡** | | | | | ምልክት ማድረጊያ ሳጥን: ☐ | | | | | | | | | | | | |  | |
| K | | የመረጃ ሰብሳቢ ስም  **የስምምነቱን ሂደት በትክክል መካሄዱን ለመመስከር ስምሽን መዝግቢ፡፡** | | | | |  | | | | | | | | | | | | |  | |
| L | | የመልስ ሰጭ ስም  **የመልስ ሰጭዋን የመጀመርያ ስም መዝግቢ፡፡** | | | | |  | | | | | | | | | | | | |  | |
| **ተ.ቁ** | | **ጥያቄዎች እና ማጣሪያዎች** | | | | | **የመለስ አማራጮች ኮድ** | | | | | | | | | | | | | **እለፊ** | |
| **ክፍል 1 –የመልስ ሰጭ አጠቃላይ መረጃዎች**  **በአጠቃለይ አሁነም ስለ እርስዎ ማህበራዊና ኢኮኖሚያዊ ጉዳዮች ልጠይቆት እወዳለሁ፡፡** | | | | | | | | | | | | | | | | | | | | | |
| 1 | | ዕድሜዎ ስንት ዓመት ነው?  **ዕድሜ በሙሉ ዓመት መመዝገብ ይኖርበታል፡፡**  **ዕድሜ በሚለው አቅጣጫ ባለው ሳጥን ውስጥ መመዝገብ ያለበት ከ15-49 ያለ ቁጥር ብቻ ነው፡፡ ከዚህ ዕድሜ ክልል ውጭ ላለ ሰው ቃለ መጠየቅ መደረግ አይኖርበትም፡፡** | | | | ዕድሜ በሙሉ ዓመት | | | | | | | | | | |  | | |  | |
| 2 | | የተከታተሉት የትምህርት ደረጃ ምንድን ነው የመጀመሪያ፣ሁለተኛ፣ቴክኒክና ሙያ፣ ወይስ ከፍተኛ ነው?   - ከፍተኛ ትምህርት 11 እና 12 ተኛ ክፍልንም ያጠቃልላል | | | | ፈፅሞ አልተከታተልኩም 0  የመጀመሪያ ደረጃ 1  ሁለተኛ ደረጃ 2  ቴክኒክና ሙያ/ኮሌጅ 3  ከፍተኛ 4 | | | | | | | | | | | | | |  | |
| 3 | | በአሁኑ ወቅት በጋብቻ ላይ ኖት ወይስ እንደ ባልና ሚስት ከወንድ ጋር እየኖሩ ነው?  **የለም ከመለሱ፣ መልስ ሰጭዋ የተፈቱ፣የተለያዩ ወይም ባል የሞተባቸው እንደሆኑ ጠይቁ ፡፡** | | | | የለም፣በፍፁም አግብው አያውቁም 0  አዎ፣ አሁን በጋብቻ ላይ ያሉ 1  አዎ፣ከወንድ ጋር እንደ ባልና ሚስት የሚኖሩ 2  በጋብቻ ላይ ያልሆኑ፣የተፋቱ/የተለያዩ 3  በጋብቻ ላይያልሆኑ፣ባል የሞተባቸው 4 | | | | | | | | | | | | | | የለም አግብተው የማያውቁ (ኮድ 0) ከሆነ ወደ ጥያቄ 8 እለፊ | |
| 4 | | አንድ ጊዜ ብቻ ነው ያገቡት (ከወንድ ጋር እንደ ባልና ሚስት የኖሩት) ወይንስ ከአንድ ጊዜ በላይ? | | | | አንድ ጊዜ ብቻ 1  ከአንድ ጊዜ በላይ 2 | | | | | | | | | | | | | | አነድ ጊዜ ከሆነ ወደ ጥያቄ 5a እለፉ ከአንድ ጊዜ በላይ ከሆነ ወደ ጥያቄ 5b እለፊ | |
| 5a | | በየትኛው ወርና ዓመተ ምህረት ነበር ከባለቤትዎ ጋር(እንደ ባልና ሚስት አብረውት ከኖሩት ወንድ ጋር) መኖር የጀመሩት? | | | | ወር: | | | | | | | |  | | | | | |  | |
|  |  |  |  |  |  | ዓመተ ምህረት: | | | | | | | |  | | | | | |  |  |
| 5b | | አሁን ደግሞ በአሁኑ ወቅት አብረውት ከሚኖሩት ባለቤትዎ ጋር (እንደ ባልና ሚስት አብረውት ከኖሩት ወንድ ጋር) መኖር የጀመሩት መቼ እንደሆነ እጠይቆታለሁ፡፡በየትኛው ወርና ዓመተ ምህረት ነው አብረው መኖር የጀመሩት? | | | | ወር: | | | | | | | |  | | | | | |  | |
|  |  |  |  |  |  | ዓመተ ምህረት: | | | | | | | |  | | | | | |  |  |
| 6 | | የእርስዎ ባለቤት (እንደ ባልና ሚስት አብረውት የሚኖሩት ወንድ) ሌላ ሚስቶች ወይም እንደ ባልና ሚስት አብረዋቸው የሚኖሩ ሴቶች አሏቸው? | | | | አዎ 1  የለም 0  አላውቅም -88 | | | | | | | | | | | | | |  | |
|  | | **ጥያቄ 3 ን ተመልከቺ:** አሁን በጋብቻ ላይ ናቸው? | | | | አዎ 1  የለም 0 | | | | | | | | | | | | | | የለም ከሆነ ወደ ጥያቄ 8 እለፊ | |
| 7 | | ባለቤትዎ ( አብረውት እንደ ባልና ሚስት የሚኖሩት ወንድ) በአሁኑ ጊዜ ከእርስዎ ጋር ነው የሚኖሩት ወይስ ሌላ ቦታ ነው የሚኖሩት? | | | | ከመልስ ሰጭ ጋር አብረው የሚኖሩ 1  ሌላ ቦታ የሚኖሩ 2 | | | | | | | | | | | | | |  | |
| **ክፍል 2 –ስነተዋልዶ እርግዝና እና መውለድ የሚፈልጓቸው ልጆች ብዛት**  **አሁን በህይወት ዘመንዎ በሙሉ ስለወለዷቸው ልጆች ልጠይቅዎ እወዳለሁ፡፡** | | | | | | | | | | | | | | | | | | | | | |
| 8 | | እስከዛሬ ድረስ በጠቅላላ ስንት ጊዜ ወልደዋል?  **ዜሮ መልስ ሊሆን ይችላል፡፡** | | | | የወለዱትጊዜ/የወለዷቸው ልጆች ብዛት | | | | | | | | | | |  | | 0 ከሆነ ወደ ጥያቄ 13 እለፊ | | |
|  | | እነዚህ የወለዱባቸው በሙሉ በህይወት የተወለዱ ነበሩ ወይም የወለዷቸው ልጆች ሁሉ በሕይወት የተወለዱ ናቸው?  **የለም ከመለሱ፣ወደ ጥያቄ 8 በመመለስ በህይወት የተወለዱ ልጆችን ብቻ መሆናቸውን በማረጋገጥ አስፈላጊውን ማስተካከያ አድርጉ፡፡** | | | | አዎ 1  የለም 0 | | | | | | | | | | | | |  | | |
| 9 | | ለመጨረሻ ጊዜ የወለዱት መቼ ነበር?  **የመጨረሻ የወለዱበትን ቀን መዝግቢ።**  **አስፈላጊ ከሆነ ቀኑ ቀደም ሲል ከነበረው ሁኔታ ከሚያስታውሱት ድርጊቶች በማስታወስ መገኘት ይኖርበታል፡፡.** | | | | ወር | | | | ዓመተ ምህረት | | | | | | | | | ባለፈው ዓመት ካልሆነ ወይም /እና በጥያቄ 8 ኮድ 1 ከሆነ ወደ ጥያቄ 11 እለፊ | | |
| 10 | | ከመጨረሻ ልጅ ቀደሞ የተወለደው ልጅ መቼ ነበር የወለዱት፣ በየትኛው ወርና ዓመተምህረት ነበር?  **ከመጨረሻ በፊት የተወለደውን ልጅ የተወለደበትን ቀን መዝግቡ፡፡**  **አስፈላጊ ከሆነ ቀኑ ቀደም ሲል ከነበረው ሁኔታ ከሚያስታውሱት ድርጊቶች በማስታወስ መገኘት ይኖርበታል፡፡.** | | | | ወር | | | | ዓመተ ምህረት | | | | | | | | |  | | |
| 11 | | መጨረሻ የወለዱት ልጅ በሕይወት አለ? | | | | አዎ 1  የለም 0  አላውቅም -88 | | | | | | | | | | | | | አዎ ከመለሱ ወደ ጥያቄ 13 እለፊ | | |
| 12 | | የመጨረሻ ልጅዎ የሞተው መቼ ነው?  **ልጁ የሞተበትን ጊዜ መዝግቡ፡፡**  **አስፈላጊ ከሆነ ቀኑ ቀደም ሲል ከነበረው ሁኔታ ከሚያስታውሱት ድርጊቶች በማስታወስ መገኘት ይኖርበታል፡፡.** | | | | ወር | | | | | | ዓመተ ምህረት | | | | | | |  | | |
| 13 | | የመጨረሻ የወር አበባዎ የጀመረው መቼ ነው?  **በቀን ፣በሳምንት፣በወር ወይም በዓመት ከመለሱ ቀጥሎ ባለው እስክሪን X በሚለው ቦታ ቁጥሩን አስገቡ፡፡** | | | | ቀናት በፊት: | | | | | | | | |  | | | |  | | |
|  |  |  |  |  |  | ሳምንታት በፊት: | | | | | | | | |  | | | |  |  |  |
|  |  |  |  |  |  | ወራት በፊት: | | | | | | | | |  | | | |  |  |  |
|  |  |  |  |  |  | ዓመታት በፊት: | | | | | | | | |  | | | |  |  |  |
|  |  |  |  |  |  | በዕድሜያቸው መግፋት የወር አበባ ማየት ያቆሙ/ማህፀናቸው በኦፕራሲዮን የወጣ/የተገለበጠ 1  የመጨረሻ ልጃቸውን ከመውለዳቸው በፊት 2  የወር አበባ አይተው አያውቁም 3 | | | | | | | | | | | | |  |  |  |
| 14 | | አሁን እርጉዝ (ነፍሰ ጡር) ነዎት? | | | | አዎ 1  የለም 0  እርግጠኛ አይደለሁም 2 | | | | | | | | | | | | | የለም ከመለሱ ወደ ጥያቄ 16a እለፊ | | |
| 15 | | የስንት ወር እርጉዝ( ነፍሰ ጡር) ነዎት?  **የጨረሱትን ሙሉ ወር ብዛት መዝግቡ፡፡ አላውቅም ብለው ለመለሱ ኮድ 88 አስቡ፡፡** | | | | የእርግዝና ወራት ብዛት | | | | | | | | | | |  | |  | | |
|  | | **ጥያቄ14ን ተመልከቺ:** አሁን እርጉዝ ናቸው? | | | | አዎ 1  የለም 0 | | | | | | | | | | | | | የለም ከመለሱ ወደ ጥያቄ16a እለፉ አዎ ካሉ ወደ ጥያቄ  16b እለፊ | | |
| 16a | | **አሁን ስለወደፊት ሀሳብዎ አንዳንድ ጥያቄዎች ልጠይቆት እፈልጋለሁ፡፡**  ልጅ (ተጨማሪ ልጅ/ልጆች) መውለድ ይፈልጋሉ ወይስ ሌላ ልጅ (ተጨማሪ ልጅ/ልጆች) መውለድ አይፈልጉም? | | | | ልጅ(ሌላ ልጅ) እንዲኖራቸውይፈልጋሉ 1  ምንም/ተጨማሪ ልጅ አይፈልጉም 2  ማርገዝ አልችልም ብለዋል 3  አልወሰኑም/አያውቁም -88 | | | | | | | | | | | | | ኮድ 1 ከተከበበ ወደ 17a ለሌሎቹ (ኮድ 2፣3 88) ወደ 18 እለፊ | | |
| 16b | | **አሁን ስለወደፊት ሀሳብዎ አንዳንድ ጥያቄዎች ልጠይቆት እፈልጋለሁ ፡፡**  አሁን ከሚወልዱት ልጅ በተጨማሪ ሌላ ልጅ/ልጆች/ መውለድ ይፈልጋሉ; ወይስ ሌላ ልጅ መውለድ አይፈልጉም? | | | | ልጅ(ሌላ ልጅ) እንዲኖራቸው ይፈልጋሉ 1  ምንም/ተጨማሪ ልጅ አይፈልጉም 2  ማርገዝ አልችልም ብለዋል 3  አልወሰኑም/አያውቁም -88 -88 | | | | | | | | | | | | | ኮድ 1 ከተከበበ ወደ 17 b ለሌሎቹ (ኮድ 2፣3 88) ወደ 18 እለፉ | | |
| 17a | | ከአሁን ጀምሮ ልጅ (ሌላ ተጨማሪ ልጅ) ሳይወልዱ ለምን ያህል ጊዜ መቆየት ይፈልጋሉ?  **ወር ወይም ዓመት ከመረጥሽ ቀጥሎ ባለው እስክሪን X በሚለው ቦታ ቁጥሩን አስገቢ፡፡** | | | | ወር: | | | | | | | | | | |  | |  | | |
|  |  |  |  |  |  | ዓመተ: | | | | | | | | | | |  | |  |  |  |
|  |  |  |  |  |  | በጣም በቅርብ/አሁን 1  ሌላ 2  ማርገዝ አይችሉም 3  አላውቅም -88 | | | | | | | | | | | | |  |  |  |
| 17b | | አሁን ከሚወልዱት ልጅ ቀጥሎ ሌላ ተጨማሪ ልጅ ሳይወልዱ ለምን ያህል ጊዜ መቆየት ይፈልጋሉ?  **ወር ወይም ዓመት ከመረጥሽ ቀጥሎ ባለው እስክሪን X በሚለው ቦታ ቁጥሩን አስገቢ፡፡ ፡፡** | | | | ወር: | | | | | | | | | | |  | |  | | |
|  |  |  |  |  |  | ዓመተ ምህረት: | | | | | | | | | | |  | |  |  |  |
|  |  |  |  |  |  | በጣም በቅርብ/አሁን 1  ሌላ 2  ማርገዝ አይችሉም 3  አላውቅም -88 | | | | | | | | | | | | |  |  |  |
|  | | **ጥያቄ 8ን ተመልከቺ:** የወለዷቸው ልጆች ብዛት  **ጥያቄ 14ን ተመልከቺ:**: አሁን እርጉዝ ነዎት? | | | | የተወለዱ ልጆች ብዛት | | | | | | | | | | |  | | ምንም ልጅ ካልወለዱ እና በጥያቄ 14 የለም ከመለሱ ወደ ጥያቄ 19 እለፊ፡፡  በጥያቄ ቁጥር 14 የለም ከመለሱ ወደ 18 እለፊ ነገር ግን አዎ ከመለሱ ወደ 18b እለፉ፡፡ | | |
|  |  |  |  |  |  | አዎ 1  የለም 0 | | | | | | | | | | | | |  |  |  |
| 18a | | **አሁን ስለ መጨረሻ ልጅዎ አንዳንድ ጥያቄዎችን ልጠይቆት እወዳለሁ፡፡**  የመጨረሻ ልጅዎን ባረገዙበት ወቅት በዛን ጊዜ ማርገዝ ይፈልጉ ነበር፣ የተወሰነ ጊዜ ቆይተው ልጅ እንዲኖሮት ይፈልጉ ነበር ፣ወይስ ምንም ልጅ (ተጨማሪ ልጆች) አይፈልጉም ነበር? | | | | በዛን ጊዜ/በወቅቱ እፈልግ ነበር 1  ቆይቼ 2  ጭራሽ አልፈልግም ነበር 3 | | | | | | | | | | | | |  | | |
| 18b | | **ስለአሁኑ እርግዝናዎ አንዳንድ ጥያቄዎችን ልጠይቆት እወዳለሁ፡፡**  ባረገዙበት ወቅት በዛን ጊዜ ማርገዝ ይፈልጉ ነበር የተወሰነ ጊዜ ቆይተው ልጅ እንዲኖሮት ይፈልጉ ነበር ፣ወይስ ምንም ልጅ (ተጨማሪ ልጆች) አይፈልጉም ነበር? | | | | በዛን ጊዜ 1  ቆይቼ 2  ጭራሽ አልፈልግም ነበር 3 | | | | | | | | | | | | |  | | |
| **ክፍል 3– የወሊድ መቆጣጠሪያ ዘዴዎች**  **አሁን ደግሞ እርስዎ ወይም ባለቤትዎ እርግዝናን ለመከላከል ወይም ለማዘግየት ብለው የሚጠቀሙበት ዘዴ ወይም የሚያደርጉት ነገር እንዳለ ልጠይቆት እወዳለሁ፡፡** | | | | | | | | | | | | | | | | | | | | | |
| 19 | | | እርስዎ እርግዝናን ለማዘግየት ወይም ለማስቀረት ብለው ማንኛውንም የቤተሰብ እቅድ ዘዴ ተጠቅመው ወይም ሞክረው ያውቃሉ? | አዎ 1  የለም 0 | | | | | | | | | | | | | | | የለም ከሆነ ወደ ጥ.25 እለፉ፡፡ | | |
| 20 | | | ለመጀመሪያ ጊዜ እርግዝናን ለማዘግየት ወይም ለማስቀረት ብለው ማንኛውንም ዘዴ ሲጠቀሙ ዕድሜዎ ስንት ነበር?  **ዕድሜ በሙሉ ዓመት መዝግቡ፡፡ ምንም ዓይነት ዘዴ ካልተጠቀሙ 0 አስገቡ፡፡ መልስ ሰጭዋ አላውቅም ካሉ -88 አስገቢ፡፡** | ዕድሜ በሙሉ ዓመት | | | | | | | |  | | | | | | |  | | |
| 20a | | | በዛን ጊዜ ልጅ ኖሮት ከነበረ በሕይወት ያሉ ስንት ልጆች ነበርዎት? | ቁጥር | | | | | | | |  | | | | | | |  | | |
| 21 | | | እርስዎ እርግዝናን ለማዘግየት ወይም ለማስቀረት ብለው ለመጀመሪያ ጊዜ የተጠቀሙት ምን ዓይነት የቤተሰብ እቅድ ዘዴ ነበር?  **በመልስ አማራጮች ላይ የተዘረዘሩትን ዘዴዎች አታንብቢ፡፡ ሁሉንም አማራጭ መልሶች ለማየት ወደ ታች ውረጂ፡፡** | ሴቶችን በኦፕራሲዮን ማምከን 1  ወንዶችን በኦፕራሲዮን ማምከን 2  ኦይ.ዩ.ዲ/ሎፕ/ 3  የወሊድ መከላከያ መርፌ 4  ኢምፕላንት 5  የወሊድ መከላከያ ኪኒንl 6  የወንድ ኮንዶም 7  የሴት ኮንዶም 8  ድንገተኛ የወሊድ መቆጣጠሪያ ………..9  ዲያፍራም…………………………….10  ፎም/ጄሊ …….11  ስታንዳርድ የቀን አቆጣጠር ዘዴ በዶቃ/ጨሌ መሳይ ነገር ……….………12  ላክቴሽናል አሚኖሪያ ዘዴ/LAM/ 13  ሌላ ዘመናዊ ዘዴ 14  ቀን አቆጣጠር ዘዴ 15  የወንድ የዘር ፍሬ ውጭ ማፍሰስ 16  ሌላ ባህላዊ ዘዴ 17 | | | | | | | | | | | | | | |  | | |
|  | | | **ጥያቄ 14 ን ተመልከቺ:**: አሁን እርጉዝ ነዎት? | አዎ 1  የለም 0 | | | | | | | | | | | | | | | አዎ ከመለሱ ወደ ጥያቄ ቁጥርo 25 እለፊ፡፡ | | |
| 22 | | | እርስዎ ወይም ባለቤትዎ (እንደ ባልና ሚስት አብረውት የሚኖሩት ወንድ) **በአሁኑ ጊዜ** እርግዝናን ለመከላከል ወይም ለማዘግየት ብለው የሚጠቀሙት የቤተሰብ እቅድ ዘዴ ወይም የሚያደርጉት ነገር? | አዎ 1  የለም 0 | | | | | | | | | | | | | | | የለም ከመለሱ ወደ ጥያቄ 25 እለፊ፡ | | |
| 23 | | | እርሶዎ የትኛውን የቤተሰብ እቅድ ዘዴ ነው እየተጠቀሙ ያሉት?  ሌላስ?  **የተጠቀሱትን ዘዴዎች በሙሉ ምረጭ፡፡ ሁሉንም አማራጭ መልሶች ለማየት ወደ ታች ውረጂ፡፡** | 1. ሴቶችን በኦፕራሲዮን ማምከን  2. ወንዶችን በኦፕራሲዮን ማምከን  3. ኦይ.ዩ.ዲ/ሎፕ/  4. የወሊድ መከላከያ መርፌ  5. ኢምፕላንት  6. የወሊድ መከላከያ ክኒን  7. የወንድ ኮንዶም  8. የሴት ኮንዶም  9. ድንገተኛ የወሊድ መቆጣጠሪያ  10. ዲያፍራም  11. /ፎም/ጄሊ  12. የቀን አቆጣጠር ዘዴ የዶቃ መቁጠሪያ በመጠቀም  13. ላክቴሽናል አሚኖሪያ ዘዴ/LAM/ ….  14. ሌላ ዘመናዊ ዘዴ  15. ቀን አቆጣጠር ዘዴ  16. የወንድ የዘር ፍሬ ውጭ ማፍሰስ  17. ሌላ ባህላዊ ዘዴ | | | | | | | | | | | | | Y  1  1  1  1  1  1  1  1  1  1  1  1  1  1  1  1  1 | N  0  0  0  0  0  0  0  0  0  0  0  0  0  0  0  0  0 | ከአንድ ዘዴ በላይ ተጠቅሜያለሁ ካሉ የይለፍ ትዕዛዝ በዝርዝር ውስጥ **በጣም ውጤታማ/**በቅደም ተከተሉ ቅድሚያ ያለውን ዘዴ መሰረት ያደረገ ይሆናል፡፡  ዋነኛው የተጠቀሙት ዘዴ ከተራ ቁጥር 3-17 ከሆነ ወደ ጥያቄ 29 እለፊ፡፡ | | |
| 24 | | | ለእርስዎ ወይም ለባለቤትዎ/ለጓደኛዎ ይህንን ያደረገሎት የጤና ባለሙያው ዘዴው ቋሚ የመከላከያ ዘዴ እንደሆነ ነግሮት ነበር? | አዎ 1  የለም 0 | | | | | | | | | | | | | | | ለሁሉም ወደ ጥያቄ 29 እለፉ፡፡ | | |
| 25 | | | ቤተሰብ እቅድ (ወይም የወሊድ መቆጣጠሪያ ዘዴ) አገልግሎት ሊያገኙበት የሚችሉበትን ቦታ ያውቃሉ? | አዎ 1  የለም 0 | | | | | | | | | | | | | | |  | | |
|  | | | **ጥያቄ 14 ን ተመልከቺ:**: አሁን እርጉዝ ነዎት? | አዎ 1  የለም 0 | | | | | | | | | | | | | | | የለም ከሆነ ወደ ጥያቄ 26a እለፊ፡፡  አዎ ከሆነ ወደ ጥያቄ 26b እለፊ፡፡ | | |
| 26a | | | እርስዎ በአሁኑ ወቅት እርግዝናን ለመከላከል የቤተሰብ እቅድ ዘዴ እንደማይጠቀሙ ነግረውኛል፡፡  ወደፊት በማንኛውም ጊዜ እርግዝናን ለማዘግየት ወይም ለመከላከል ብለው የየቤተሰብ ምጣኔ ዘዴ እጠቀማለሁ ብለው ያስባሉ? | አዎ 1  የለም 0 | | | | | | | | | | | | | | |  | | |
| 26b | | | ወደፊት በማንኛውም ጊዜ እርግዝናን ለማዘግየት ወይም ለመከላከል ብለው የቤተሰብ እቅድ ዘዴ እጠቀማለሁ ብለው ያስባሉ? | አዎ 1  የለም 0 | | | | | | | | | | | | | | |  | | |
|  | | | **ጥያቄ ቁጥር 19 ን ተመልከቺ**: የቤተሰብ እቅድ ዘዴ ተጠቅመው ያውቃሉ? | አዎ 1  የለም 0 | | | | | | | | | | | | | | | የለም ከሆነ ወደ ጥያቄ 43 እለፊ፡፡ | | |
| 27 | | | ባለፉት 12 ወራት ውስጥ እርግዝናን ለማስቀረት ወይም ለማዘግየት የተጠቀሙት የቤተሰብ እቅድ ዘዴ ወይም ያደረጉት ነገር አለ? | አዎ 1  የለም 0 | | | | | | | | | | | | | | | የለም ከሆነ ወደ ጥያቄ 43 እለፊ፡፡ | | |
| 28 | | | እርሶዎ ለመጨረሻ ጊዜ/ በአሁኑ ወቅት የተጠቀሙት/ እየተጠቀሙ ያሉት የትኛውን የቤተሰብ እቅድ ዘዴ ነው?  ሌላስ?  በጣም ውጤታማ የሆነውን ዘዴ ምረጡ፡፡ **(ከአንድ ዘዴ በላይ ተጠቅሜያለሁ ካሉ በዝርዝር ውስጥ መጀመሪያ ላይ ያለውን አስገቡ፡፡)**  **ሁሉንም አማራጭ መልሶች ለማየት ወደ ታች ውረጂ፡፡** | ኦይ.ዩ.ዲ/ሎፕ/ 3  የወሊድ መከላከያ መርፌ 4  ኢምፕላንት 5  የወሊድ መከላከያ ክኒን 6  የወንድ ኮንዶም 7  የሴት ኮንዶም 8  ድንገተኛ የወሊድ መቆጣጠሪያ 9  ዲያፍራም 10  ፎም/ጄል 11  ስታንዳርድ የቀን አቆጣጠር ዘዴ በዶቃ/ጨሌ መሳይ .12  ላክቴሽናል አሚኖሪያ ዘዴ/LAM ………………13  ሌላ ዘመናዊ ዘዴ……………………...14  ቀን አቆጣጠር ዘዴ 15  የወንድ የዘር ፍሬ ውጭ ማፍሰስ …………………………..16  ሌላ ባህላዊ ዘዴ 17 | | | | | | | | | | | | | | |  | | |
| 29 | | | በየትኛው ወር እና ዓመተ ምህረት ነው ያለማቋረጥ (ለመጨረሻ ጊዜ/አሁን) የተጠቀሙትን / የሚጠቀሙትን የቤተሰብ እቅድ ዘዴ መጠቀም የጀመሩት?  **ቀኑን መዝግቢ፡፡ ቃለመጠይቅ ከተደረገበት ወር ጀምሮ ወደ ኃላ በመመለስ የሚያስታውሱትን ክስተቶች እንዲያሰሉ በማድረግ ጊዜውን ለማወቅ /ሊታወቅ ይችላል፡፡** | ወር | | | | | | | | ዓመተ ምህረት | | | | | | |  | | |
|  | | | **ጥየቄ 22ን ተመልከቱ:** በአሁኑ ወቅት የቤተሰብ እቅድ ዘዴ እየተጠቀሙ ነው? | አዎ 1  የለም 0 | | | | | | | | | | | | | | | አዎ ከሆነ ወደ ጥያቄ ቁጥር 32 እለፊ፡፡ | | |
| 30 | | | (ለመጨረሻ ጊዜ የተጠቀሙትን ዘዴ) መጠቀም ያቆሙት መቼ ነው?  **ቀኑን መዝግቡ፡፡ ቃለመጠይቅ ከተደረገበት ወር ጀምሮ ወደ ኃላ በመመለስ የነበሩ ክስተቶችን እንዲያስታውሱ በማድረግ መጠቀም የጀመሩበትን ቀን ማግኘት ይቻላል፡፡.** | ወር | | | | | | | | ዓመተ ምህረት | | | | | | |  | | |
| 31 | | | (ለመጨረሻ ጊዜ የተጠቀሙትን ዘዴ) መጠቀም ያቆሙት ለምን ነበር? | አልፎ አልፎ ብቻ የግብረ ሥጋ ግንኙነት ስለሚያድርጉ/ባለቤታቸውስለሌሉ 1  ዘዴውን እየተጠቀሙ እያለ ስላረገዙ 2  ለማርገዝ ስለፈለጉ 3  ባላቸው(ወንድጓደኛቸው)ስለሚቃወሙ 4  በጣም ውጤታማ የሆነ ዘዴ ስለፈለጉ 5  የሚፈልጉት አይነት ዘዴ ስለሌለ 6  ለጤናቸው በማሰብ 7  የሚያስከትለውን ጉዳት በመፍራት 8  ማግኘትስለማይችሉ/በጣምሩቅ ስለሆነ 9  ዋጋው በጣም ውድ ስለሆነ 10  አጠቃቀሙ ምቹ ስላልሆነ 11  እሞታለሁ /ይገለኛል ብሎ ማሰብ 12  ለማርገዝ ችግር ያለባቸው/ያረጡ/የተላለፋቸው/ 13  የሰውነትን የተፈጥሮሂደትስለሚያዛባ…14  ሌላ 15  አላውቅም -88 | | | | | | | | | | | | | | |  | | |
| 32 | | | (ለመጨረሻ ጊዜ/አሁን እየተጠቀሙ ያለውን ዘዴ) መጀመሪያ ሲጠቀሙት በዚያን ጊዜ ከየት ነበር ያገኙት?  የተጠቀሙትን ዘዴ ከጥያቄ ቁጥር 28 በማየት በቅንፍ ወስጥ በማስገባት ጠይቁ፡፡  ***ሁሉንም አማራጭ መልሶች ለማየት ወደ ታች ውረጂ፡፡*** | የመንግስት ጤና ተቋም  የመንግስት ሆስፒታል 1  የመንግስት ጤና ጣቢያ 2  የመንግስት ጤና ኬላ 3  የቤተሰብ ምጣኔ ክሊኒክ 4  ተንቀሳቃሽ ክሊኒክ 5  የፊልድ ሰራተኛ/ ውሎ ገብ የጤና ሰራተኛ/አቻመምህር .6  የግል ጤና ተቋም  የግል ሆስፒታል/ክሊኒክ/ 7  የግል ዶክተር 8  ፋርማሲ/መድሃኒት ቤት 9  መድሀኒት መደብር 10  የቤተሰብ ምጣኔ ክሊኒክFP/PPAG clinic 11  ሌላ ምንጭ  ሱቅ/ገበያ 12  ቤተክርስቲያን/መስኪድ 13  የጤና ልማት ሠራዊት 14  ጓደኛ /ዘመድ 15  መንግስታዊ ያልሆነ ድርጅት 16  ሌላ 17  አላውቅም -88 | | | | | | | | | | | | | | |  | | |
| 33 | | | ባለፉት 12 ወራት ውስጥ ለቤተሰብ እቅድ አገልግሎት (ለመጨረሻ ጊዜ/አሁን እየተጠቀሙ ላለው ዘዴ) ማንኛውንም ዓይነት ክፍያ ከፍለው ነበር? | አዎ 1  የለም 0 | | | | | | | | | | | | | | | የለም ከሆነ ወደ ጥያቄ ቁጥር 35 እለፊ፡፡ | | |
| 34 | | | ምን ያህል ነበር የከፈሉት?  **የከፈሉትን የገንዘብ መጠን በኢትዮጵያ ብር ፃፊ፡፡**  **ከአንድ በላይ የቤተሰብ እቅድ** **ዘዴ እየተጠቀሙ ከሆነ ድምሩ ይወሰድ**  **መልስ ሰጭዋ አላውቅም ብለው ከመለሱ -88 በተሰጠው ክፍት ቦታ ሙሊ፡፡** | የገንዘቡ ልክ በኢትዮጵያ ብር: | | | | | | | | | | | | | | |  | | |
| 35 | | | (ለመጨረሻ ጊዜ/አሁን እየተጠቀሙ ያለውን ዘዴ) ባገኙበት ወቅት በዚያን ጊዜ ስለ ቤተሰብ እቅድ ( እርግዝናን ለማዘግየት ወይም ለማስቀረት የሚጠቀሙበት) ዘዴ ሊያስከትልብዎ ስለሚችለው ችግሮችና (ጎጂ ጎኖች) በጤና ባለሙያዎች ወይም በቤተሰብ እቅድ ሠራተኛ ተነግሮዎት ነበር? | አዎ 1  የለም 0 | | | | | | | | | | | | | | | የለም ከሆነ ወደ ጥያቄ ቁጥር 37 እለፊ፡፡ | | |
| 36 | | | ችግሮች ወይም ጎጂ ጎኖቹ ሲያጋጥምዎት ምን ማድረግ እንዳለብዎት ተነግሮዎት ነበር? | አዎ 1  የለም 0 | | | | | | | | | | | | | | |  | | |
| 37 | | | በዛን ጊዜ እርስዎ (ለመጨረሻ ጊዜ/አሁን ) እየተጠቀሙ ካለው ዘዴ ውጭ ስለ ሌሎች ሊጠቀሙባቸው ስለሚችሏቸው የቤተሰብ እቅድ ዘዴዎች በጤና ወይም በቤተሰብ ምጣኔ ሰራተኛ ተነግሮት ነበር? | አዎ 1  የለም 0 | | | | | | | | | | | | | | |  | | |
| 38 | | | በዛ ጉብኝት ወቅት እርግዝናን ለማዘግየት ወይም ለማስቀረት ሊጠቀሙበት የፈለጉትን ዘዴ አገኙ? | አዎ 1  የለም 0 | | | | | | | | | | | | | | | አዎ ከሆነ ወደ ጥያቄ ቁጥር 40 እለፊ፡፡ | | |
| 39 | | | ካላገኙ ፣ ለምን ነበር ያለገኙት?  (የቤተሰብ እቅድ (እርግዝናን ለማዘግየት ወይም ለማስቀረት ሊጠቀሙበት የፈለጉትን) ዘዴ ያላገኙት ለምን ነበር? | በዛን ቀን ዘዴው ከሰቶክ ስላለቀ 1  ዘዴው በጭራሽ አልነበረም/የለም 2  ጤና ባለሞያዎቹ ዘዴውን ለመስጠት ስልጠና አልወሰዱም 3  ጤና ባለሞያዎቹ የመከሩት ሌላ ዘዴ በመሆኑ 4  ዘዴውን ለመጠቀም ብቁ ስላልሆኑ 5  ዘዴውን እንዳይለመድ ስለተወሰነ 6  በጣም ውድ ስለሆነ 7  ሌላ 8 | | | | | | | | | | | | | | |  | | |
| 40 | | | በዛን የጉብኝት ወቅት ምን ዓይነት /የትኛውን ዓይነት ዘዴ መጠቀም /መውሰድ እንዳለብዎት የመጨረሻ ውሳኔ የሰጠው ማን ነበር? | ብቻዎን ነው የወሰኑት 1  ጤና ባለሙያው 2  ጓደኛዎ/ባለቤትዎ 3  ጤና ባለሙያውና እርስዎ 4  እርስዎና ባለቤትዎ/የወንድ ጓደኛዎ 5  ሌላ 6 | | | | | | | | | | | | | | |  | | |
|  | | | **ጥየቄ 32ን ተመልከቱ:** (ለመጨረሻ ጊዜ/አሁን እየተጠቀሙ ያለውን ዘዴ) ከየት ነው ያገኙት?  የተጠቀሙትን ዘዴ ከጥያቄ ቁጥር 28 በማየት በቅንፍ ወስጥ በማስገባት ጠይቁ፡፡  ***ሁሉንም አማራጭ መልሶች ለማየት ወደ ታች ውረጂ፡፡*** | የመንግስት ጤና ተቋም  የመንግስት ሆስፒታል 1  የመንግስት ጤና ጣቢያ 2  የመንግስት ጤና ኬላ 3  የቤተሰብ ምጣኔ ክሊኒክ 4  ተንቀሳቃሽ ክሊኒክ 5  የፊልድ ሰራተኛ/ ውሎ ገብ የጤና ሰራተኛ/አቻመምህር .6  የግል ጤና ተቋም  የግል ሆስፒታል/ክሊኒክ/ 7  የግል ዶክተር 8  ፋርማሲ/መድሃኒት ቤት 9  መድሀኒት መደብር 10  የቤተሰብ ምጣኔ ክሊኒክFP/PPAG clinic 11  ሌላ ምንጭ  ሱቅ/ገበያ 12  ቤተክርስቲያን/መስኪድ 13  የጤና ልማት ሠራዊት 14  ጓደኛ /ዘመድ 15  መንግስታዊ ያልሆነ ድርጅት 16  ሌላ 17  አላውቅም -88 | | | | | | | | | | | | | | | በጥያቄ 32 ላይ መልሱ ከኮድ 12 እስከ 15 ከተከበበ ወደ ጥያቄ ቁጥር 44 እለፊ፡፡ | | |
| 41 | | | ወደዚህ የጤና ባለሙያ/ጤና ተቋም ወደፊት ይመለሳሉ? | አዎ 1  የለም 0 | | | | | | | | | | | | | | |  | | |
| 42 | | | ወደዚህ ጤና ባለሙያ/ጤናተቋም ዘመዶችዎን ወይም ጓደኞችዎ እንዲሄዱ ይነግራሉ/ይመክራሉ? | አዎ 1  የለም 0 | | | | | | | | | | | | | | |  | | |
|  | | | **ጥየቄ 16 ን ተመልከቺ:** ወደፊት ልጅ/ተጨማሪ ልጆች ለመውለድ ይፈልጋሉ?  **ጥየቄ 17 ተመልከቺ:** ሌላ ልጅ ሳይወልዱ 2 ዓመትና ከዚያ በላይ መቆየት ይፈልጋሉ?  **ጥየቄ 22 ተመልከቺ:** በአሁኑ ጊዜ እርግዝናን ለመከላከል ወይም ለማዘግየት ማንኛውንም ዘዴ ይጠቀማሉ?  **ጥየቄ 19 ተመልከቺ:** እርግዝናን ለመከላከል ወይም ለማዘግየት ማንኛውንምየቤተሰብ ምጣኔ ዘዴ ተጠቅመው ያውቃሉ? | ልጅ(ሌላ ልጅ) እንዲኖራቸውይፈልጋሉ 1  ምንም/ተጨማሪ ልጅ አይፈልጉም 2  ማርገዝ አልችልም ብለዋል 3  አልወሰኑም/አያውቁም -88 | | | | | | | | | | | | | | | ( ተጠቅመው የማያው ወይም አሁንም የማይጠቀሙ)ልጅ/ተጨማሪ ልጅ ለማይቁፈልጉ ወይም እስከ 2 ዓመት መቆየት የሚፈልጉ  ጥያቄ 43 ጠይቂ | | |
|  |  |  |  | ምንም/ተጨማሪ ልጅ አይፈልጉም 1  ከ2 ዓመት ያነሰ 2  2 ዓመትና ከዛ በላይ 3 | | | | | | | | | | | | | | |  |  |  |
|  |  |  |  | አዎ፣ ዘዴ ይጠቀማሉ 1  የለም፣ማንኛውንም ዘዴ አይጠቀሙም 0 | | | | | | | | | | | | | | |  |  |  |
|  |  |  |  | አዎ 1  የለም 0 | | | | | | | | | | | | | | |  |  |  |
| 43 | | | **እርስዎ ምንም ልጅ ወይም ተጨማሪ ልጅ መውለድ እንደማይፈልጉና እርግዝናን ለመከላከል ማንኛውንም የወሊድ መቆጣጠሪያ ዘዴ እንደማይጠቀሙ ነግረውኛል፡፡**  እርግዝናን ለመከላከል ወይም ለማዘግየት የወሊድ መቆጣጠሪያ ዘዴ የማይጠቀሙበትን ዋነኛ ምክንያት ሊነግሩኝ ይችላሉ? | አልፎ አልፎ ብቻ የግብረ ሥጋ ግንኙነት ስለሚያድርጉ/ባለቤታቸው ራቅ ያለ ቦታ ስለሆኑ 1  ያረጡ/የተላለፋቸው/ማህፀናቸውበኦፕሬሽን የተገለበጠ 2  በተወሰነ/ሙሉ ሙሉማርገዝ የማይችሉ 3  መጨረሻ ከወለዱ የወርአበባ ስላላዩ 4  ጡት ያጠባሉ 5  እንደ እግዚአብሄር ፈቃድ/ እንደአመጣጡ የሚሉ 6  መልስ ሰጭዋ ስለሚቃወሙ 7  ባላቸው(ወንድ ጓደኛቸው)ስለሚቃወሙ 8  ሌሎች ስለሚቃወሙ 9  ሀይማኖታቸው ስለሚከለክል 10  የሚያውቁት ዘዴ የለም 11  የሚገኝበትን ቦታ አያውቁም 12  የሚያስከትለውን ጉዳት በመፍራት 13  ለጤናቸው በማሰብ 14  ማግኘት ስለማይችሉ/በጣም ሩቅ 15  ዋጋው ውድ ስለሆነ 16  የሚፈልጉት አይነት ዘዴ ስለሌለ 17  ምንም አይነት ዘዴ ስለሌለ 18  አጠቃቀሙ ምቹ ስላልሆነ 19  የሰውነትን የተፈጥሮ ሂደት ስለሚያዛባ 20  ሌላ 21  አያውቁም -88 | | | | | | | | | | | | | | |  | | |
| 44 | | | ባለፉት 12 ወራት ውስጥ ስለቤተሰብ ምጣኔ ወይም ስለ እርግዝና መከላከያ ዘዴ በሚያስተምር የጤና ሰራተኛ ወይም ሌላ የጤና ባለሙያ ተጎብኝተው ነበር? | አዎ 1  የለም 0 | | | | | | | | | | | | | | |  | | |
| 45 | | | ባለፉት 12 ወራት ውስጥ ለራስዎ ወይም ለልጂዎ/ለልጆችዎ ጤንነት ብለው ወደ ጤና ተቋም ሄደው ነበር? | አዎ 1  የለም 0 | | | | | | | | | | | | | | | የለም ከሆነ ወደ ጥያቄ ቁጥር 47 እለፊ፡፡ | | |
| 46 | | | በዚያ ወቅት ማንኛውም የጤና ተቋም ውስጥ ያለ የጤና ባለሙያ ስለቤተሰብ ምጣኔ ወይም ስለ እርግዝና መከላከያ ዘዴዎች ነግሮዎት ነበር? | አዎ 1  የለም 0 | | | | | | | | | | | | | | |  | | |
| 47 | | | በአለፉት ጥቂት ወራት ውስጥ እርስዎ፡-  እርስዎ ስለቤተሰብ ምጣኔ ወይም እርግዝና መከላከያ ዘዴዎች  በሬዲዮ ሰምተው ነበር? ስለቤተሰብ ምጣኔ ወይም እርግዝና መከላከያ ዘዴዎች  በቴሌቪዝን አይተው ነበር? ስለቤተሰብ ምጣኔ ወይም እርግዝና መከላከያ ዘዴዎች  በጋዜጣ ወይም በመጽሔት አንብበው ነበር? |  | | | | | | | | | አዎ  1  1  1 | | | | የለም  0  0  0 | |  | | |
| **ማስታወሻ ለመረጃ ሰብሳቢ፡** አሁን ግላዊ የሆኑ ሁለት ጥያቄዎች እነደምትጠይቅያቸው በመግለፅና ሚስጥራወነቱ በማስታወስ ቀጥዪ። | | | | | | | | | | | | | | | | | | | | | |
| 48 | | | ለመጀመሪያ ጊዜ የግብረሥጋ ግንኙነት ሲፈፅሙ እድሜዎ ስንት ነበር?  **በተሰጠው ክፍት ቦታ ዕድሜ በሙሉ ዓመት አስገቡ፡፡ የግብረስጋ ግንኙነት ፈጽመው የማያውቁ ከሆነ 0 አስገቡ። መልስ ሰጭዋ አላውቅም ብለው ከመለሱ -88 አስገቢ።** | ዕድሜ በሙሉ ዓመት | | | | | | | | |  | | | | | | 0 ከሆነ ወደ ጥያቄ ቁጥር 50 እለፊ፡፡ | | |
| 49 | | | ለመጨረሻ ጊዜ የግብረ ስጋ ግንኙነት ያደረጉት መቼ ነበር?  **ከ12 ወር (አንድ ዓመት በላይ) ከሆነ መልሱ በዓመት መሞላት አለበት**  **ከ 12 ወር በታች ከሆነ መልሱ በቀን፣ በሳምንት ወይም በወር መሞላት አለበት።**  **በዛው ዕለት ከሆነ 0 ቀን መዝገቢ።**  **ቀጥሎ ባለው ስክሪን X በሚለው ቦታ ቁጥሩን አስገቢ።** | ከቀናት በፊት | | | | | ከሳምንታት በፊት | | | | ከወራት በፊት | | | | ከዓመታት በፊት | |  | | |
| **ክፍል 4 – ውሃ**  **አሁን የውሃ አጠቃቀም ልምድዎትን በተመለከተ አንዳንድ ጥያቄዎችን ልጠይቆት እወዳለሁ፡፡** | | | | | | | | | | | | | | | | | | | | | |
| 50 | | በበጋ ወቅት ውሃ ወደሚገኝበት ቦታ ለመሄድ፣ ለመቅዳትና ቀድቶ ለመመለስ በቀን ምን ያህል ሰዓት ይወስድቦታል?  **መመዝገብ ያለበት መልስ ሰጭዋ ውሃ ለመቅዳት በቀን የሚፈጅባቸውን ሰዓት ብቻ ነው፤ ሌላ ሰው የቀዳበት ሰዓት መሞላት የለበትም፡፡**  **ደቂቃ ወይም ሰዓት ከመረጣችሁ ቀጥሎ ባለው ስክሪን X በሚለው ቦታ ቁጥሩን አስገቢ።** | | | በቀን ውሃ ለመቅዳት የሚያጠፉት ሰዓት | | | | | | | | | | | |  | | | |  |
|  |  |  |  |  | ውሃ የሚቀዳው ሌላ ሰው ነው 33  ውሃ የሚቀዳ ሰው የለም 44  አላውቅም -88 | | | | | | | | | | | | | | | |  |
| 51 | | በክረምት ወቅት ውሃ ወደሚገኝበት ቦታ ለመሄድ፣ ለመቅዳትና ቀድቶ ለመመለስ በቀን ምን ያህል ሰዓት ይወስድቦታል?  **መመዝገብ ያለበት መልስ ሰጭዋ ውሃ ለመቅዳት በቀን የሚፈጅባቸውን ሰዓት ብቻ ነው፤ ሌላ ሰው የቀዳበት ሰዓት መሞላት የለበትም፡፡**  **ደቂቃ ወይም ሰዓት ከመረጣችሁ ቀጥሎ ባለው ስክሪን X በሚለው ቦታ ቁጥሩን አስገቢ።** | | | በቀን ውሃ ለመቅዳት የሚያጠፉት ሰዓት | | | | | | | | | | | |  | | | |  |
|  |  |  |  |  | ውሃ የሚቀዳው ሌላ ሰው ነው 33  ውሃ የሚቀዳ ሰው የለም 44  አላውቅም -88 | | | | | | | | | | | | | | | |  |
| **መልስ ሰጭዋ ቃለመጠይቅ ለማድረግ ጊዜያቸውን ስለሰጡን አመስግኑ፡፡**  **መረጃ ሰብሳቢ፡ መልስ ሰጭዋ እዚህ ላይ ጨርሰዋል ፡፡ ነገር ግን አንቺ ማጠናቀቅ ያለብሽ ከቤት ውጭ የሚሞሉ ተጨማሪ ሁለት ጥያቄዎች አሉ፡፡** | | | | | | | | | | | | | | | | | | | | | |
| **ጥናቱ የሚካሄድበት ቦታ እና የቃለመጠይቁ ውጤት** | | | | | | | | | | | | | | | | | | | | | |
| M | | **ወደ ቤተሰቡ ቤት መግቢያ አጠገብ የጂ.ፒ. ኤስ ንባብ (ሰሜን ምስራቅ ፖይንት) ውሰጂ።**  **የጂፒ.ኤሱ ልኬቶች ንባብ ትክክለኛነቱ ከ6 ሜትር በታች ከሆነ ቦታውን መዝግቢ።** | | | | | *በ ODK ሶፍት ዌር መመሪያ ይሰጣል፡፡*  ቦታውን መዝግቢ | | | | | | | | | | | | | |  |
| **የመጠይቁ ውጤት መመዝገቢያ** | | | | | | | | | | | | | | | | | | | | | |
| N | | **የመልስ ሰጭዋን የቃለመጠይቅ ውጤት መዝግቢ።** | | | | | ተጠናቋል 1  ቤት አልተገኙም 2  ተላልፏል 3  ፈቃደኛ አይደሉም 4  በከፊል ተጠናቋል 5  መልስ መስጠት አይችሉም 6 | | | | | | | | | | | | | |  |

***ለቃለመጠይቁ ስለተባበሩኝ በጣም አመሰግናለሁ፡፡***
