# Supplementary material for: Does proximity of women to facilities with better choice of contraceptives affect their contraceptive utilization in rural Ethiopia?
Source: PLoS One. 2017 Nov 13;12(11):e0187311. doi: 10.1371/journal.pone.0187311 (PMC5683563; doi:10.1371/journal.pone.0187311)
Supplement: S1 File — (ZIP) [file pone.0187311.s004.zip › Questionnaires/Amharic version/HQ_Amharic_14 Nov 13.doc]

**mADDS –የቤተሰብ መጠይቅ**

| **ተ.ቁ** | **ጥያቄዎችና ማጣሪያዎች** | **የመልስ አማራጮችና ኮዶች** | | | | | | **እለፊ** | |
| --- | --- | --- | --- | --- | --- | --- | --- | --- | --- |
| **መለያ/መግቢያ**  **እባካችሁ ቃለመጠይቁን ከመጀመራችሁ በፊት ለጥናቱ የተመረጠውን ቤተሰብ መለያ መረጃ መዝግቡ፡፡** | | | | | | | | | |
| A | ይህንን ቤተሰብ ለምን ያህል ጊዜ ነው የጎበኘሺው? | ለመጀመሪያ ጊዜ 1  ለሁለተኛ ጊዜ 2  ለሶስተኛ ጊዜ 3 | | | | | | |  |
| B | **የመረጃ ሰብሳቢዋ ስም:** ይህ የአንቺ ስም ነው?  ካልሆነ ፣ እባክሽን ስማሽን መዝግቢ:  *ከስልክ ቁጥር ጋር የተያያዘውን ስም ODK ያሳያል ፡፡* | አዎ 1  የለም 0 | | | | | | |  |
|  |  |  | | | | | | |  |
| C | **የአሁኑ ቀን እና ሰዓት በስክሪኑ ላይ ይታያል፡፡**  ይህ ቀን እና ሰዓት ትክከል ነው? | አዎ 1  የለም 0 | | | | | | | የለም ከሆነ ወደ E እለፊ |
| D | ትክክለኛውን ቀን እና ሰዓት መዝግቢ | ቀን | ወር | | ቀን | | ዓ/ም | |  |
|  |  | ጊዜ | ሰዓት | ደቂቃ | | ጥዋት/ከሰዓት | | |  |
| E | ክልል  **እባካችሁ ቤተሰቡ ያለበትን ክልል ስም ምረጡ ፡፡** | ትግራይ 1  አፋር 2  አማራ 3  ኦሮሚያ 4  ሶማሊ 5  ቤንሻንጉል ጉሙዝ 6  ደ/ብ/ብ/ሕ/ክ/ 7  ጋምቤላ 8  ሐረሪ 9  አዲስ አበባ 10  ድሬ ዳዋ 11 | | | | | | |  |
| F | ዞን  **ቤተሰቡ የሚገኝበትን ዞን ስም መዝግቢ** | *በዚህ ቤተሰብ መጠይቅ ላይ በጥያቄ* *E በተመረጠው ክልል መሰረት ODK የተመረጠውን ዞን ዝርዘር ያሳያል/ያወጣል፡፡* | | | | | | |  |
| G | የቀበሌ/የወረዳ/ የአካባቢ ስም  **ቤተሰቡ የሚገኝበትን ቀበሌ ስም/የአካባቢ መለያ መዝግቢ** | *በዚህ ቤተሰብ መጠይቅ ላይ በጥያቄ* *E በተመረጠው ዞን መሰረት ODK የተመረጠውን ቀበሌ/ ወረዳ ዝርዘር ያሳያል/ያወጣል፡፡* | | | | | | |  |
| H | የቆጠራ ቦታ ኮድ  **ቤተሰቡ የሚገኝበትን የቆጠራ ቦታ ኮድ/ቁጥር መዝግቢ** |  | | | | | | |  |
| I | የቤት ተራ ቁጥር  **የቤቱን ተራ ቁጥር ከቤተሰብ መመዝገቢያ ቅፅ ላይ በመውሰድ መዝግቢ፡፡** |  | | | | | | |  |
| J | የቤተሰብ ተራ ቁጥር  **የቤተሰብ ተራ ቁጥሩን ከቤተሰብ መመዝገቢያ ቅፅ ላይ በመውሰድ መዝግቢ፡፡** |  | | | | | | |  |
| K | በዕለቱ ቃለ መጠይቅ የሚደረግለት አንድ የቤተሰቡ አባል እና መልስ ሊሰጥ የሚችል ሰው በዚህ ቤተሰብ ተገኝቷል? | አዎ 1  የለም 0 | | | | | | | የለም ከሆነ ወደ P እለፊ |
| **የስምምነት ማረጋገጫ**  **መልስ ለመስጠት ብቁ የሚሆነውን የቤተሰብ አባል አግኙ፡፡የሚከተለውን መግቢያና የስምምነተ ማረጋገጫ አንቀፅ አንብቡ፡፡** | | | | | | | | | |
| እንደምን ዋላችሁ/አደራችሁ። ስሜ __________________ ይባላል፡፡ ለአዲስ አበባ ዩኒቨርስቲ እና ለጤና ጥበቃ ሚኒስቴር እየሰራሁ እገኛለሁ፡፡ እኛ የተለያዩ የጤና ጉዳዮችን በተመለከተ በሀገር ውስጥ ጥናት እያካሄድን እንገኛለን፡፡ እርስዎን በዚህ ጥናት ላይ በመሳተፍዎ እጅግ አድርገን እናመሰግናለን። ይህ መረጃ መንግስት በጤና አገልግሎት ላይ በተሸለ ሁኔታ እንዲያቅድ ለማሳወቅ ይረዳናል፡፡ እርስዎ የሚሰጡት ማንኛውም መረጃዎች/መልሶች በሙሉ በሚስጢር የሚያዙ ሲሆን ከጥናቱ አባላት በስተቀር በምንም ሁኔታ በሌላ ወገን አይታዩም፡፡  በዚህ ጥናት ላይ የሚሳተፉት በፈቃደኝነት ሲሆን ያለመመልስና በማንኛዉም ግዜ ቃለ-መጠይቁን የማቆም መብት አሎት። ነገር ግን የእርስዎ በዚህ ጥናት መሳተፍና የእርስዎን አስተያየት ማግኘት ጠቃሚ ስለሚሆን በጥናቱ ለመሳተፍ እና ለጥያቄዎቹ መልስ ለመስጠት ፈቃደኛ እንደሚሆኑ ተስፋ አደርጋለሁ፡፡  አሁን ስለ እርስዎ እና የቤተሰብዎ አባላት አንዳንድ ጥያቄዎችን እጠይቆታለሁ፡፡ ከዛም ከዚህ ቤተሰብ አባላት ውስጥ ዕድሜያቸው ከ15 እስከ 49 መካከል የሆኑ ሴቶችን አንዳንድ ሴቶች የሚመለከቱ ጥያቄዎችን ለመጠየቅ እወዳለሁ፡፡  አሁን ስለ ጥናቱ ሊጠይቁኝ የሚፈልጉት ነገር አለ?  **መረጃ ሰብሳቢ፡** አዎ ካሉ ለጥያቄያቸው በቂ ማብራርያ በመስጠት ወደ ሚቀጥለው እለፊ። | | | | | | | | | |
| L | የጥናት አድራጊው አድራሻ የያዘው ፎርም ኮፒ ከሰጣችሁ በኃላ አብራሩላቸው፡፡ ከዛም ቃለመጠይቁን መጀመር እንደምትችሉ ጠይቁ፡፡  አሁን ቃለ መጠይቁን መጀመር እችላለሁ? | አዎ 1  የለም 0 | | | | | | የለም ከሆነ ወደ P እለፉ | |
| M | **መልስ ሰጭው/ዋ በጥናቱ ለመሳተፍ መስማማታቸውን ለማረጋገጥ በማጣሪያ ሳጥን ውስጥ ምልክት አድርጊ፡፡** | ማጣሪያ ሳጥን: ☐ | | | | | |  | |
| N | የመረጃ ሰብሳቢ ስም  **የስምምነቱን ሂደት በትክክል መካሄዱን ለመመስከር ስምሽን መዝግቢ፡፡** |  | | | | | |  | |
| O | የመልስ ሰጭ ስም  **የመልስ ሰጭ የመጀመርያ ስም መዝግቡ፡፡** |  | | | | | |  | |

| **ክፍል1 – የቤተሰብ መመዝገቢያ**  **አሁን ስለ ሁሉም የቤተሰብዎ አባላት አንዳንድ ጥያቄዎችን እጠይቆታለሁ ፡፡ ከእርስዎ ልጀምር እባክዎ፣ለእያንዳንዱ የቤተሰብ አባላት በአብዛኛው ከዚህ ቤተሰብ ጋር የሚኖርና በትላንትናው ዕለት ሌሊት ከዚህ ቤተሰብ ጋር ያደሩ ሰዎችን ጭምር ከእርስዎ በመጀመር ያስመዝግቡን፡፡** | | | | | | | | | |
| --- | --- | --- | --- | --- | --- | --- | --- | --- | --- |
| ተ.ቁ | 1 | 2 | 3 | 4 | | 5 | 6 | 7 | 8 |
|  | ስም | ፆታ | ዕድሜ (በሙሉ ዓመት) | የጋብቻ ሁኔታ | | ከቤተሰቡ ኀላፊ ጋር ያላቸው ዝምድና | የቤተሰብ መለያ ቁጥር | ይህ ሰው መደበኛ የቤተሰቡ አባል ነው ወይስ እሱ/እሷ በትላንትናው ሌሊት ያደሩት ከዚህ ቤተሰብ ጋር ነው? | ለጥናቱ ብቁ የሆነች ሴት መልስ ሰጭ |
|  |  | ወንድ 1  ሴት 2 |  | ያገቡ/በትዳር ላይ 1 እንደ ባልና ሚስት አብረው የሚኖሩ 2 የተፋቱ/የተለያዩ 3 የሞተባት/የሞተችበት 4 አግብተው የማያውቁ/ያላገቡ 5 | | ኃላፊ 1  ሚስት/ባል 2  ወንድ ልጅ/ሴት ልጅ 3  የልጅ ባል/ሚስት 4  የልጅ ልጅ 5  ወላጅ 6  የባል/የሚስትአባት/እናት 7  ወንድም/እህት 8  ሌላ 9  አላውቅም -88 |  | መደበኛ የቤተሰቡ አባል 1  በትላንትናው ሌሊት ከዚህ ቤተሰብ ጋር ያላደሩ መደበኛ የቤተሰቡ አባል 2  በትላንትናው ሌሊት እዚህ ቤትያደረ እንግዳ 3 | አዎ 1  የለም 0  *ለጥናቱ ብቁ የሆኑትን ODK ለይቶ ያሳያል፡፡* |
| 1 |  |  |  |  | |  |  |  |  |
| 2 |  |  |  |  | |  |  |  |  |
| 3 |  |  |  |  | |  |  |  |  |
| 4 |  |  |  |  | |  |  |  |  |
| 5 |  |  |  |  | |  |  |  |  |
| 6 |  |  |  |  | |  |  |  |  |
| 7 |  |  |  |  | |  |  |  |  |
| 8 |  |  |  |  | |  |  |  |  |
| 9 |  |  |  |  | |  |  |  |  |
| 10 |  |  |  |  | |  |  |  |  |
| *የቤተሰብ አባላት ከመዘገባችሁ በኃላ ሳይመዘገቡ የቀሩ የቤተሰቡ አባላት እንዳሉ ለማጣራት፣የሚከተለውን ማጣሪያ ጥያቄ ጠይቁ፡፡ ያልተመዘገበ ሰው ካለ መረጃው ጠይቁና ሙሉ፡፡* | | | | | | | | | |
| 9 | ሌላ መደበኛ የቤተሰቡ አባል የሆኑ ወይም በትላንትናው ሌሊት እዚህ ያደሩ ሰዎች አሉ ? | | | | አዎ 1  የለም 0 | | | | የለም ከሆነ ወደ ጥያቄ 10 እለፉ፡፡ |

| **ክፍል 2 – ቤተሰቡ የሚገለገልባቸው ቁሳቁሶች/ንብረቶች**  **አሁን ቤተሰቡ ስለሚጠቀምባቸው ቁሳቁሶች/ንብረቶች ለማወቅ የሚያስችሉ አንዳንድ ጥያቄዎችን ልጠይቆት እወዳለሁ፡፡** | | | | | | | | | | | |
| --- | --- | --- | --- | --- | --- | --- | --- | --- | --- | --- | --- |
| 10 | *መረጃ ሰብሳቢ፡ ከዚህ በታች የተዘረዘሩትን ቁሳቁሶች/ንብረቶች በክፍት ቦታው እያስገባችሁ እያንዳንዱን ጠይቁ፡፡*  ቤተሰቡ________ አለው?  ኤሌክትሪክ መብራት አለው;?  የግድግዳ ሰዓት አለው?  ራዲዮ አለው?  ጥቁር እና ነጭ ከለር ቴሌቪዢን ?  ከለር ቴሌቪዢን ?  ሞባይል /ተንቀሳቃሽ ስልክ  የቀጥታ መስመር ስልክ/የቤት መደበኛ ስልክ?  ማቀዝቀዣ?  ትልቅ ማቀዝቀዣ/deep freezer?  የኤሌትሪክ ጀነሬተር?  የልብስ ማጠቢያ ማሽን?  ኮምፒዩተር?  ዲጂታል ፎቶ ካሜራ?  ዲጂታል ያልሆነ ፎቶ ካሜራ/?  የቪዲዮ ዴክ?  ዲቪዲ/ቪሲዲ?  የልብስ ስፌት መኪና?  አልጋ?  ጠረጴዛ?  ሳጥን መደርደሪያ ያለው/ቁም ሳጥን?  ብስክሌት?  የሞተር ብስክሌት?  የቤት/የጭነት መኪና?  ሞተር ያለው ጀልባ?  ሞተር የሌለው ጀልባ?  ከላይ ከተጠቀሱት አንዱም የለውም  **.** |  | | አዎ  1  1  1  1  1  1  1  1  1  1  1  1  1  1  1  1  1  1  1  1  1  1  1  1  1  1  1  1  -88 | | የለም  0  0  0  0  0  0  0  0  0  0  0  0  0  0  0  0  0  0  0  0  0  0  0  0  0  0  0  0 | | |  | | |
| 11 | ለእርሻም ሆነ ለሌላ አገልግሎት የሚውሉ ማንኛዉም ዓይንት እንስሳት አሎት? | አዎ 1  የለም 0 | | | | | | | የለም ከሆነ ወደ ጥ. 13 እለፊ፡፡ | | |
| 12 | ለእያንዳንዱ እንስሳት ጠይቁ፡  በጎች /ፍየሎች/አሳማዎች  ዶሮዎች/ዳክዬዎች /የወፍ ዘሮች  የቀንድ ከብቶች/ ፈረሶች/አህያዎች/ግመሎች  **ዜሮ መልስ ሊሆን ይችላል፡፡**  **አላውቅም ካሉ -88 ይጻፍ፡፡**  **መልስ አልተሰጠም ከሆነ -99 ፃፊ** |  | | | | | | |  | | |
| **ክፍል 3 –ቤተሰቡ ስለሚኖርበት ቤት አጠቃላይ ሁኔታ**  **እባካችሁ የቤቱን ወለል፣ ጣሪያና የውጭውን ግድግዳ ተመልክታችሁ ሙሉ፡፡** | | | | | | | | | | | |
| 13 | የቤቱ ወለል በአብዛኛው የተሠራው/የተሸፈነው በምን እንደሆነ ***ተመልክተሽ*** አክብቢ፡፡ | የተፈጥሮ ወለል  አፈር/አሸዋ 1  በእበት የተለቀለቀ 2  በመጠኑ የተሰራ ወለል  ሣንቃ/ያልተላገ እንጨት 3  ቀርከሃ/ሸንበቆ 4  ሙሉ በሙሉ የተሰራ ወለል  ፓርኬ/የተላገ ጣውላ 5  አስፋልት 6  ሴራሚክ ታይልስ/ቴራዞ 7  ሲሚንቶ 8  የፕላስቲክ/ከሱፍ የተሰራ ምንጣፍ 9  Linoleum/rubber carpet 10  ሌላ 11 | | | | | | |  | | |
| 14 | የቤቱ ጣሪያ በአብዛኛው የተሠራው ከምን እንደሆነ ***ተመልክተሽ*** አክብቢ | የተፈጥሮ ጣሪያ  ጣሪያ የለውም 1  ሳር/ቅጠል/ጭቃ 2  መሠረታዊ ጣሪያ ያለው  Rustic Mat 3  ቀርከሃ/ሸንበቆ 4  ሣንቃ/ያልተላገ እንጨት 5  ካርቶን/ Cardboard 6  ሙሉ በሙሉ የተሠራ ጣሪያ  ቆርቆሮ 7  የተላገ እንጨት 8  አስቤስቶስ/ሲመንት ፋይበር 9  ሴራሚክ/ሸክላ 10  ሲሚንቶ/ኮንክሪት 11  Roof Shingles 12  አስቤስቶስ የተቆራረጠ ለጣራ የሚሆን 13  ሌላ 14 | | | | | | |  | | |
| 15 | የቤቱ ግድግዳ በአብዛኛው የተሰራው ከምን እንደሆነ ***ተመልክተሽ*** አክብቢ | የተፈጥሮ ግድግዳ  ግድግዳ የለውም 1  ግንድ/ቀርከሃ/ሸንበቆ 2  መሠረታዊ ግድግዳ ያለው  ቀርከሃ/እንጨት እና ጭቃ 3  ድንጋይና ጭቃ 4  እንጨትና ጭቃ 5  ክፍት ከሆነ ጡብ የተሠራ 6  ንብርብር ጣውላ 7  ካርቶን/Cardboard 8  ፍርስራሽ እንጨት 9  ሙሉ በሙሉ የተሠራ ግድግዳ  ሲሚንቶ 10  ድንጋይና ኖራ/ ሲሚንቶ 11  ጡብ 12  ድፍን የሲሚንቶ ብሎኬት 14  ድፍን ከሆነ ጡብ የተሠራ 15  ያልተላገ እንጨት/ሣንቃ 16  ሌላ 17 | | | | | | |  | | |
| **ክፍል 4 – ውሃ፣ የአካባቢ ፅዳትና ጤና አጠባበቅ**  **አሁን ቤተሰቡ ለመጠጥና ለሌላ አገልግሎት የሚጠቀምበት ውሃ ፣ ስለ አካባቢ ፅዳትና ጤና በተመለከተ አንዳንድ ጥያቄዎች ልጠይቆት እወዳለሁ፡፡** | | | | | | | | | | | |
| 16 | እጃችሁን የምትታጠቡበት የተዘጋጀ የእጅ መታጠቢያ ቦታ አለ? | | አዎ 1  የለም 0  አላውቅም -88 | | | | | | | | የለም ከሆነ ወደ ጥ ቁ 19 እለፉ |
| 17 | እባክዎን ሊያሳዩኝ ይችላሉ? | | አዎ 1  የለም 0 | | | | | | | | የለም ከሆነ ወደ ጥ ቁ 19 |
| 18 | **ቤተሰቡ ለእጅ መታጠቢያ ባዘጋጀው ስፍራ ላይ የሚከተሉት መኖራቸውን ተመልከቺ:**  ሳሙና /ፈሳሽ ሳሙና አለ?  በተለያየ ዕቃ/በጀሪካን/የተጠራቀመ ውሃ አለ?  የቧንቧ ውሃ አለ ?  የእጅ መታጠቢያው ስፍራ በመፀዳጃ ቤቱ አጠገብ ነው  ከላይ የተዘረዘረው ሁሉ የለም | |  | | አዎ  1  1  1  1  1 | | | የለም  0  0  0  0  0 | | |  |
| 19 | ቤተሰቡ አብዛኛውን ጊዜ ማለትም በዓመቱ በማንኛውም ወቅት **ለማንኛውም** አገልግሎት የሚጠቀምበትን ውሃ የሚያገኘው የት እንደሆነ እያንዳንዱን በመጠየቅ ተገቢውን ኮድ አክብቢ፡፡  ከቧንቧ ውሃ  ከቧንቧ በቤት ውስጥ  ከቧንቧ በግቢ ውስጥ  የህዝብ ቧንቧ(ቦኖ)  ከጥልቅ ጉድጓድ በእጅ/በእግር በማወዛወዝ የሚወጣ ዉሃ  የጉድጓድ ውሃ  የተጠበቀ ጉድጓድ  ያልተጠበቀ ጉድጓድ………………….  የምንጭ ውሃ  የተጠበቀ ምንጭ  ያልተጠበቀ ምንጭ  የዝናብ ውኃ  በቦቴ መኪና ከሚታደል ዉሃ  በጋሪ እየተጎተተ ከሚታደል/የሚመጣ ዉሃ  የገጸ ምድር ውሃ (ወንዝ፣ግድብ፣ሐይቅ፣  ኩሬ፣ ቦይ፣ የመስኖ ውሃ  የታሸገ ጠርሙስ/የፐላስቲክ ውሃ  **እያነዳነዱን እያነበብሽ የተጠቀሙትን ኮድ 1 አክብቢ ያልተጠቀሙትን ኮድ 2 አክብቢ፡፡** | |  | | አዎ  1  1  1  1  1  1  1  1  1  1  1  1  1  1  1 | | | የለም  0  0  0  0  0  0  0  0  0  0  0  0  0  0  0 | | |  |
| 20 | የቤተሰቡ አባላት በአብዛኛው **የመጠጥ ውኃ** የሚያገኙት ከየት ነው?  ከቧንቧ ውሃ  ከቧንቧ በቤት ውስጥ  ከቧንቧ በግቢ ውስጥ  የህዝብ ቧንቧ(ቦኖ)  ከጥልቅ ጉድጓድ በእጅ/በእግር በማወዛወዝ የሚወጣ ዉሃ  የጉድጓድ ውሃ  የተጠበቀ ጉድጓድ  ያልተጠበቀ ጉድጓድ………………….  የምንጭ ውሃ  የተጠበቀ ምንጭ  ያልተጠበቀ ምንጭ  የዝናብ ውኃ  በቦቴ መኪና ከሚታደልዉሃ  በጋሪ እየተጎተተ ከሚታደል/የሚመጣ ዉሃ  የገጸ ምድር ውሃ (ወንዝ፣ግድብ፣ሐይቅ፣  ኩሬ፣ ቦይ፣ የመስኖ ውሃ  የታሸገ የፕላስቲክ ጠርሙስ/የፕለስቲክ ከረጢት ውሃ  **ሁሉንም አንብቢላቸውና በዋነኛነት/በአብዛኛው የሚጠቀሙትን ውሃ የሚያገኙበትን ቦታ አክብቢ፡፡**  **በቤተሰብ መጠይቅ ጥያቄ ቁጥር 19 ላይ ለተመረጡ /አዎ/ ለተሞላላቸው መሆን ይኖርበታል፡፡** | | 1  2  3  4  5  6  7  8  9  10  11  12  13  14 | | | | | | | |  |
| 21 | የእርስዎ ቤተሰብ **በአብዛኛው ለሌላ አገልግሎት** ለምሳሌ ምግብ ለማዘጋጀትና እጅን ለመታጠብ የሚጠቀምበትን ውሃ የሚያገኘው ከየት ነው?  ከቧንቧ ውሃ  ከቧንቧ በቤት ውስጥ  ከቧንቧ በግቢ ውስጥ  የህዝብ ቧንቧ(ቦኖ)  ከጥልቅ ጉድጓድ በእጅ/በእግር በማወዛወዝ የሚወጣ ዉሃ  የጉድጓድ ውሃ  የተጠበቀ ጉድጓድ  ያልተጠበቀ ጉድጓድ………………….  የምንጭ ውሃ  የተጠበቀ ምንጭ  ያልተጠበቀ ምንጭ  የዝናብ ውኃ  በቦቴ መኪና ከሚታደል ዉሃ  በጋሪ እየተጎተተ ከሚታደል/የሚመጣ ዉሃ  የገጸ ምድር ውሃ (ወንዝ፣ግድብ፣ሐይቅ፣  ኩሬ፣ ቦይ፣ የመስኖ ውሃ)  የታሸገ የፕላስቲክ ጠርሙስ/የፕላስቲክ ከረጢት ውሃ  **ሁሉንም አንብቢላቸውና በዋነኛነት/በአብዛኛው የሚጠቀሙትን ውሃ የሚያገኙበትን ቦታ ክበቡ፡፡**  **በቤተሰብ መጠይቅ ጥያቄ ቁጥር 19 ላይ ለተመረጡ /አዎ/ ለተሞላላቸው መሆን ይኖርበታል፡፡** | | 1  2  3  4  5  6  7  8  9  10  11  12  13  14 | | | | | | | |  |
| 22 | **በዚህ የቤተሰብ መጠይቅ በጥያቄ ቁጥር 19 ለእያንዳንዱ ለተጠቀሰው የውሃ ምንጭ ከጥያቄ ቁጥር 22 እስከ 24 ድረስ ____ ጊዜ ይደገማል፡፡. እነዚህም የሚከተሉትን ይጨምራሉ፡**    *ODK ሶፍት ዌር በዚህ የቤተሰብ መጠይቅ በጥያቄ ቁጥር 19 የተጠቀሰውን የውሃ ምንጭ/ ውሃ የሚገኝበት ቦታ ዝርዘር ያሳያል፡፡*  እርስዎ ከ[ *ውሃው የሚገኝበት ቦታ*].እንደሚያገኙ ነግረውኛል እና በዓመት ውስጥ በማንኛውም ጊዜ እንደሚጠቅሙም ነግረውኝ ነበር፡፡ የእርስዎ ቤተሰብ ከ[ ውሃው የሚገኝበት ቦታ] የሚያገኙትን ውሃ ለ______________ ይጠቀሙበታል?  ለመጠጥ  ምግብ ለማብሰል  ለከብቶች  ለአትክልት ቦታ/ለእርሻ ቦታ  ለንግድ  *ODK ሶፍት ዌር በጥያቄ 19 ላይ ለተጠቀሱት የውሃ ምንጮች/ መገኛ ቦታዎች በሙሉ ተመሳሳይ ጥያቄ ያዘጋጃል/ያመነጫል፡፡* | |  | | አዎ  1  1  1  1  1 | | የለም  0  0  0  0  0 | | | |  |
| 23 | ይህ *[ውሃው የሚገኝበት ቦታ/ምንጭ].* አብዛኛውን ጊዜ ይኖራል :  ዓመቱን በሙሉ  ከዓመቱ የተወሰነ ጊዜ  ከዓመት ውስጥ በጣም ትንሽ ጊዜ 3  *ODK ሶፍት ዌር በጥያቄ 19 ላይ ለተጠቀሱት የውሃ ምንጮች/ መገኛ ቦታዎች በሙሉ ተመሳሳይ ጥያቄ ያዘጋጃል/ ያመነጫል፡፡* | | 1  2  3 | | | | | | | |  |
| 24 | ውሃ እናገኛለን ብላችሁ በፈለጋችሁበት/ በጠበቃችሁበት ጊዜ ሁሌ በአብዛኛው ውሃው ይኖራል?  አዎ ፣ሁልጊዜ ይኖራል  የለም፣ አልፎ አልፎ ይገኛል/መች እንደሚገኝም ይታወቃል  የለም፣ አልፎ አልፎ ይገኛል መች እንደሚገኝ ግን አይታወቅም  *ODK ሶፍት ዌር በጥያቄ 19 ላይ ለተጠቀሱት የውሃ ምንጮች/ መገኛ ቦታዎች በሙሉ ተመሳሳይ ጥያቄ ያዘጋጃል፡፡* | | 1  2  3 | | | | | | | |  |
| 25 | ውሃ ቀድቶ ለማምጣት [ውሃ የሚገኝበት ቦታ] ለመሄድ (ለአንድ መንገድ ብቻ) ምን ያህል ጊዜ ይወስዳል?    **ውሃውን ለመቅዳት ወረፋ የጠበቁበትን ጊዜ ይጨምራል፡፡**  **በጣም ቅርብ ከሆነ እና ምንም ደቂቃ አይወስድም ካሉ 0 ሊሞላ ይችላል፡፡**  **አላውቅም ካሉ -88 ይፃፍ፡፡**  *ODK ሶፍት ዌር በጥያቄ 19 ላይ ለተጠቀሱት የውሃ ምንጮች/ መገኛ ቦታዎች በሙሉ ተመሳሳይ ጥያቄ ያዘጋጃል፡፡* | | ደቂቃ | |  | | | | | |  |
| 26 | ቤተሰብዎ የአትክልት ቦታ አለው? | | አዎ 1  የለም 0 | | | | | | | |  |
| 27 | የእርስዎ የቤተሰብ አባላት ከዚህ በታች ከተዘረዘሩት የመጸዳጃ አገልግሎት ዓይነቶች የትኛውን ይጠቀማል? ቤተሰቡ______ መፀዳጃ ይጠቀማል ?  ዘመናዊ (ውሃ የሚለቅ/የሚያወርድ) መፀዳጃ  ከ____:  ቆሻሻ ማስወገጃ ቱቦ የተያያዘ  ወደ ፈሳሽና ሠገራ ማጠራቀሚያ ጉድጓድ ጋር የተያያዘ .  ከሌላ ቦታ ጋር የተያያዘ  አይታወቅም/ እርግጠኛ አይደለሁም/ አላውቅም  የአየር ማስተንፈሻ ቱቦ ያለው የጉድጓድ ሽንት ቤት (VIP)  የጉድጓድ ሽንት ቤት ሊሾ ክዳን ያለው  የጉድጓድ ሽንት ቤት ሊሾ ክዳን የሌለው  በማዳበሪያነት ለመጠቀም የተዘጋጀ የጉድጓድ ሽንት ቤት  ግዝያዊ ማጠራቀምያ ባልዲ  ከስር ወደ ወንዝ የሚጣል መፀዳጃ  ሌላ  **አማራጭ መልሶችን በሙሉ አንብበሽ የተጠቀሙትን በሙሉ ምልክት አድርጉ፡፡** | |  | | አዎ  1  1  1  1  1  1  1  1  1  1  1 | | | የለም  0  0  0  0  0  0  0  0  0  0  0 | | |  |
| 28 | የእርስዎ ቤተሰብ **በዋነኛነት/በአብዛኛው** የሚጠቀመው ምን ዓይነት መፀዳጃ ነው?  ዘመናዊ እና ውሃ የሚለቅ/የሚያፈስ መፀዳጃ ከ____የተያያዘ:  ቆሻሻ ማስወገጃ ቱቦ  ፈሳሽና ሠገራ ማጠራቀሚያ ጉድጓድ  ወደ ሌላ ቦታ የሚወርድ  አታወቅም/ እርግጠኛ አይደለሁም/ አላውቅም  የአየር ማስተንፈሻ ቱቦ ያለው የጉድጓድ ሽንት ቤት (VIP)  የጉድጓድ ሽንት ቤት ሊሾ ክዳን ያለው  የጉድጓድ ሽንት ቤት ሊሾ ክዳን የሌለው  በማዳበሪያነት ለመጠቀም የተዘጋጀ የጉድጓድ ሽንት ቤት  ግዝያዊ ማጠራቀምያ ባልዲ  ከስር ወደ ወንዝ የሚጣል መፀዳጃ  ሌላ:  **አማራጭ መልሶችን በሙሉ አንብበሽ በዋነኛነት የሚጠቀሙበትን ምልክት አድርጉ፡፡. ከጥያቄ 25 መመረጥ ይኖርበታል፡፡** | | 1  2  3  4  5  6  7  8  9  10 | | | | | | | |  |
| 29 | **በጥያቄ 27 እያንዳንዱ የመፀዳጃ ዓይነት ከተመረጠ ጥያቄ ቁጥር 29 ____ ጊዜ ይደገማል፡፡ እነዚህ የመፀዳጃ ዓይነቶች የሚከተሉተ ናቸው:**  *የODK ሶፍት ዌር በጥያቄ 27.ላይ ለተጠቀሱት የመፀዳጃ ዓይነቶች በሙሉ ይዘረዝራል፡፡*  የእርስዎ ቤተሰብ በዚህ [**የመፀዳጃ ዓይነት**] ምን ያህል ጊዜ ነው የምትጠቀሙበት?  **በቤት ሁልጊዜ የሚጠቀምበትን ብቻ ነው፡፡**    *ODK ሶፍት ዌር በጥያቄ 27. ላይ ለተጠቀሱት የመፀዳጃ ዓይነቶች በሙሉ ተመሳሳይ ጥያቄ ያዘጋጃል፡፡* | | ሁልጊዜ 1  አብዛኛውን ጊዜ 2  አልፎ አልፎ 3  አንዳንዴ 4 | | | | | | | |  |
| 30 | ከእርስዎ ቤተሰብ ውስጥ አብዛኛውን ጊዜ በሜዳ ወይም በቁጥቋጦ በቤትዎ ወይም በስራዎ አካባቢ የሚፀዳዱት /የሚጠቀሙት ምን ያህል ሰዎች ናቸው?  **በዚህ ቤተሰብ ውስጥ ____ ሰዎች ውጭ ይፀዳዳሉ፡፡ አላውቅም ከሆነ -88 ይፃፍ፡፡** | | የሰዎች ብዛት | |  | | | | | |  |
| 31 | በእርስዎ ቤተሰብ ውስጥ ዕድሜያቸው ከ 5 ዓመት በታች ካሉ የሕፃናቶቹን ሠገራ/ዓይነምድር ለማስወገድ ምን ዓይነት ዘዴ ነው የምትጠቀሙት?  ልጆች መፀዳጃ ቤት ይጠቀማሉ  ሰገራው እዛው ባለበት ይተዋል  በሜዳ ላይ/በግቢው ውስጥ ይቀበራል  መፀዳጃ ቤት ውስጥ ይጣላል/ይከተታል  ቆሻሻ ማጠራቀሚያ ውስጥ ይወረወራል  ወደ ውሃ/ከቆሻሻ መውረጃ ቱቦ ይደፋል  ለማዳበሪያ ይጠቀሙበታል  ሰገራው ይቃጠላል  አላውቅም  *ODK ሶፍት ዌር ይህንን ጥያቄ የሚጠይቀው በቤተሰብ መመዝገቢያ ቅፅ ላይ ከአምስት ዓመት በታች ልጆች ያላቸው ቤተሰቦች ብቻ ነው(የቤተሰብ መጠይቅ ቁ.3)* | |  | | አዎ  1  1  1  1  1  1  1  1  -88 | | | የለም  0  0  0  0  0  0  0  0 | | |  |
| መልስ ሰጭውን/ሰጭዋን ጊዜውን/ዋን ስለሰጠችሁ/ስለሰጠቻችሁ አመሰግኑ፡፡  **ከመልስ ሰጭው ጋር ያለው ቃለምልልስ አልቋል፡፡ ነገር ግን አንቺ ከቤት ውጭ የምትሞይው ሁለት ተጨማሪ ጥያቄዎች ይቀራሻል፡፡,** | | | | | | | | | | | |
| **የመጠይቁ ውጤትና ቦታ** | | | | | | | | | | | |
| P | የቤተሰቡ መግቢያ በር ላይ በውጭ በኩል የጂ. ፒ. ኤስ ንባብ ነጥብ ውሰጂ፡፡  ልኬቱን የሚያሳየው ከ6 ሜ. በታች ከሆነ ቦታውን መዝግቡ፡፡  **የጂ.ፒ.ኤስ ንባብ የኮኦርድኔት ነጥቦች የሚወሰደው ከቤት ውጭ ስትሆኑ ብቻ ነው፡፡** | *መመሪያው/ትዕዛዝ የሚሰጠው በቀጥታ በODK ሶፍት ዌር ነው፡፡*  **የጂ.ፒ.ኤስ ንባብ** **መዝግቡ** | | | | | | | |  | |
| Qa | የቤቱን መግቢያ ፎቶ ለመንሳት ፍቃድ ጠይቂ፡፡ ፎቶውን ለማንሳት ፍቃድ አግኝተሻል? | አዎ 1  የለም 0 | | | | | | | | የለም ከሆነ ወደ R እለፊ | |
| Qb | ምንም ዓይነት ሰው በፎቶግራፉ ላይ አለመኖሩን አረጋግጪ፡፡ | *መመሪያው/ትዕዛዝ የሚሰጠው በቀጥታ በODK ሶፍት ዌር ነው፡፡*  ፎቶ አንሺ  ምስሉን ምረጪ | | | | | | | |  | |
| R | የቤተሰብ መጠይቅ ቃለመጠይቅ ውጤት መዝግቢ፡፡ | ተጠናቋል 1  በጉብኝት ወቅትአንድም የቤተሰቡ አባል እቤት አልተገኘም ወይም መልስ የሚሰጥ አልተገኝም 2  በቀጠሮ ተላልፏል 3  ፈቃደኛ አይደሉም 4  በከፊል ተጠናቋል 5  መኖሪያ ቤቱ ባዶ ሆኗል/መኖሪያ ቤት አይደለም 6  መኖሪያ ቤቱ ፈርሷል 7  መኖሪያ ቤቱ አልተገኘም 8 | | | | | | | |  | |

***ለቃለመጠይቁ ስለተባበሩኝ በጣም አመሰግናለሁ፡፡***
