# Supplementary material for: Does proximity of women to facilities with better choice of contraceptives affect their contraceptive utilization in rural Ethiopia?
Source: PLoS One. 2017 Nov 13;12(11):e0187311. doi: 10.1371/journal.pone.0187311 (PMC5683563; doi:10.1371/journal.pone.0187311)
Supplement: S1 File — (ZIP) [file pone.0187311.s004.zip › Questionnaires/Tigrigna Version/PMA2020_Female Respondent Survey_Tigrigna_ R1.doc]

**mADDS –መጠይቕ ደቂ ኣንስትዮ**

| **ቑፅሪ** | **ሕቶን መፃረዪን** | | | | | **ናይ መማረፅታት ኮድ** | | | | | | | | | | **ሕለፍ** |
| --- | --- | --- | --- | --- | --- | --- | --- | --- | --- | --- | --- | --- | --- | --- | --- | --- |
| **መለለዪ**  **ቃለመሕትት ቅድሚ ምጅማር እዞም ዝስዕቡ መለለዪ መረዳእታ መዝግቡ::** | | | | | | | | | | | | | | | | |
| A | | ኣብ ትኽክለኛ (ዝተመርፀ) ስድራቤት ዲኹም ዘለኹም? እዚ ፎቶ ናይቲ ገዛ ቕድሚቱ እንትኾን ቅጥዒ መመዝገቢ ዝርዝር ስድራቤት ኣብ ዝምለአሉ እዋን ዝተልዓለ ፎቶ እዩ::  *ኣብ እዋን ምዝገባ ስድራ ቤት ዝተወሰድ ፎቶ ምስቲ መሕትት እዚ ብምትሕሓዝ ODK ከርእየና እዩ።* | | | | እወ 1  ኣይሉን 0 | | | | | | | | | |  |
| B | | ምስ መልሲ ወሃቢት ቃለመሕትት ንምክያድ ናብዚ ስድራቤት ክንደይ ጊዘ ዑደት ተገይሩ? | | | | ንመጀመርታ ግዘ 1  ንኻልኣይ ግዘ 2  ንሳልሳይ ግዘ 3 | | | | | | | | | |  |
| C | | ሽም መረዳእታ ሰብሳቢት፡ እዚ ሽምኪ ድዩ?  እንተድኣ ዘይኮይኑ ሽምኪ ፀሓፊ  *ODK ምስ ስልኪ ቑፅሪ ዝዛመድ ሽም ከውፀአልኪ እዩ* | | | | እወ 1  ኣይኮነን 0 | | | | | | | | | |  |
|  |  |  |  |  |  |  | | | | | | | | | |  |
| D | | **ኣብ እስክሪን ዘሎ ሰዓትን ዕለትን** ተመልከቲ  እቲ ኣብ እስክሪን ዘሎ ዕለትን ሰዓትን ትኽክል ድዩ? | | | | እወ 1  ኣይፋሉን 0 | | | | | | | | | | እወ እንተኾይኑ ናብ F ሕለፍ/ፊ |
| E | | ትኽክለኛ ዕለትን ሰዓትን መዝግቢ | | | | ዕለት | ዕለት | | ወርሒ | | | | ዓ/ም | | |  |
|  |  |  |  |  |  | ሰዓት | ሰዓት | | ደቒቓ | | | | Nንጉሆ/ድሕሪ ሰዓት | | |  |
| F | | ቀፂሉ ዘሎ መረዳእታ ካብ መመዝገቢ ዝርዝር ስድራቤት ዝተወሰደ እንትኾን፣ ቃለ መሕትት እትገብርሉ ዘለኺ ሰብ ትኽክለኛ መልሲ ዝህብ ምኑ ኣረጋግፂ ፡፡  *ኣብ መመዝገቢ ዝርዝር ስድራቤት ዘሎ ክልል፣ዞን፣ወረዳ፣ ኣከባቢ፣ ቖፀራ ቦታ፣ ቑፅሪ ገዛን መለለዪ ቑፅሪ ስድራቤትን ምስ መጠይቕ ደቂ ኣንስትዮ ብምትሕሓዝ ODK የርኢ እዩ ::* | | | |  | | | | | | | | | |  |
| G | | ምስ መልሲ ወሃቢት ዘለኩም ኣፍልጦ ከመይ ይመስል? | | | | ብጣዕሚ ንፋለጥ 1  ፅቡቕ ንፋለጥ 2  ኣፀቢቕና ኣይንፋለጥን 3  ኣይንፋለጥን 4 | | | | | | | | | |  |
| H | | መልሲ ወሃቢት ሎመዓንቲ ቃለ መሕትት ንኽግበረለን ተረኺበን ዶ? | | | | እወ 1  ኣይፋሉን 0 | | | | | | | | | | የለን እንተኾይኑ ናብ M ሕለፍ/ፊ |
| **ስምምዕ ፍቓድ**  **ቃለ መሕትት ንምክያድ ዕድመአን ካብ 15-49 ዓመት ዝኾና ደቂ ኣንስትዮ ምህላወን ኣረጋግፁ፡፡ እቲ ቃለመሕትት ንምጅማር ማንም ሰብ ኣብዘይብሉን ዘይሰምዐሉን ቦታ ክካየድ ኣለዎመልሲ ወሃቢት ግዴታ ንበይና ምኻና ኣረጋግፂ፡፡ቐፂሉ ዝቐረበ ፍቓድ ስምምዕ ድማ ከም መእተዊ ኣንብብለን** | | | | | | | | | | | | | | | | |
| ጥዕና ይሃበለይ፡፡ሽመይ________________________________ይበሃል፡፡ኣብ ኣዲሰ ኣበባ ዩኒቨርስትን ሚንስትሪ ሓለዋ ጥዕናን እየ ዝሰርሕ፡፡ኣብዚ እዋን እዚ ኣብ ምሉእ ኢትዮጵያ ንደቂ ኣንስትዮ ብዛዕባ ስነተዋልዶ ጥዕና ብዝምልከት መፅናዕቲ እናካየድና ንርከብ፡፡ኣብዚ መፅናዕቲ ተሳታፊት ብምኻንክን ካብ ልቢ ኢና ነመስግን ፡፡ዝህባና መረዳእታ ድማ መንግስቲ ብዛዕባ ጥዕና ንምምሕያሽ ንዝነድፎም ትልምታት ዓብዪ ረብሓ ዘለዎ ፡፡እዚ ቃለ መሕትት ኣስታት ካብ 15-20 ደቒቓ ኣቢሉ እዩ ዝወስድ፡፡ ዝህባና ሓበሬታ ብምሽጥር ዝተሓዝ ኣብ ርእሲ ምኻኑ ካብ ኣባላት ጉጅለ መፅናዕቲ ወፃኢ ኻልእ ሰብ ክሪኦ ኣይኽእልን፡፡ ኣብዚ መፅናዕቲ ምስታፍ ኣብ ሰናይ ድልየትክን ዝተመስረተ እንትኾን ክትምልስኦ ዘይትደልየኦ ዓይነት ሕቶ እንተልዒለ ድማ ይንገራኒ እሞ ናብ ዝቕፅል ሕቶ ክሓልፍ እየ ወይድማ ነዚ ቓለ መሕትት ኣብ ዝኾነ እዋን ከቋርፅኦ ይኽእላ እየን ኾይኑ ግና ዝህባና ሓበሬታ ንፅንዓትና ዓብዪ ረብሓ ስለዘለዎ ኣብዚ መፅናዕቲ ሙሉእ ብሙሉእ ከምዝሳተፋ ተስፋ ንገብር፡፡  ብዛዕባ እዚ መፅናዕቲ ብዝምልከት ዝኾነ ዓይነት ሕቶ ዶ ይህልወክን? | | | | | | | | | | | | | | | | |
| I | | ስምምዕ ፍቓድ ዝሓዘ ኮፒ ወረቐት ንመልሲ ወሃቢት ብምቕራብ ኣብራህርህለን ብምቕፃል ድማ ሐዚ ነቲ ቃለመሕትት ክጅምር ዶ ይኽእል? ብምባል ሕተቲ፡፡ | | | | እወ 1  ኣይፋሉን 0 | | | | | | | | | | የለን ተኾይኑ ናብ M ሕለፍ/ፊ |
| J | | ፊርማ መልሲ ወሃቢት  ወልሲ ወሃቢት ኣብዚ ፅንዓት ተሳታፊት ንምን ንኽፍርማ ወይድማ ኣብ ዝተዳለወ ሳፁን ምልክት ንኽገብራ ሕተቲ፡፡ | | | | ፊርማ፡___________  ምልክት መግበሪ ሳፁን ☐ | | | | | | | | | |  |
| K | | ሽም መረዳእታ ኣካቢት  መልሲ ወሃቢት ኣብዚ መፅናዕቲ ንምስታፍ ከም ዝተስማዕመዓ ንምምስካር ሽምኪ ፅሓፊ | | | |  | | | | | | | | | |  |
| L | | ሽም መልሲ ወሃቢት  ሙሉእ ሽም መልሲ ወሃቢት ፀሓፊ | | | |  | | | | | | | | | |  |
| **ቑፅሪ** | | **ሕቶን መፃረዪን** | | | | **ናይ መማረፅታት ኮድ** | | | | | | | | | | **ዝለል** |
| **ቀደማይ ክፋል፡-ጠቕላላ መረዳእታ መልሲ ወሃቢት**  **ሐዚ ድማ ብዛዕባ ማሕበራውን ኢኮኖምያውን ጉዳያትክን ዝምልከት ዝተወሰኑ ሕቶታት ክሓተክን እየ ፡፡** | | | | | | | | | | | | | | | | |
| 1 | | ዕድመኽን ክንደይ እዩ?  ዕድመ ብሙሉኡ ዓመት ክፀሓፍ ኣለዎ። **ዕድመ ኣብ መንጎ 15 ን 49 ዘሎ ጥራሕ እዩ ዝምላእ ካብዚ ወፃኢ እንተኾይኑ ቃለ መሕትት ምክያድ የብሉን፡፡** | | | | ዕድመ ብሙሉኡ ዓመት | | | | | | | |  | |  |
| 2 | | ዝተኸታተልክንኦ ዝለዓለ ብርኪ ትምህርቲ ክንደይ እዩ?  ቐደማይ ብርኪ፣ካልኣይ ብርኪ፣ኮሌጅ ወይድማ ቴክንክን ሞያን ወይስ ላዕለዋይ ብርኪ (ዩኒቨርስቲ) እዩ? | | | | ተኸታቲለ ኣይፈልጥን 0  ቐደማይ ብርኪ 1  ካልኣይ ብርኪ 2  ቴክንክን ሞያን/ኮሌጅ 3  ላዕለዋይ(ዩኒቨርስትን ካብኡ ንላዕልን) 4 | | | | | | | | | |  |
| 3 | | ሐዚ ሓዳር ዶ ኣለኽን ወይድማ ልክዕ ከም ስበኣይን ሰበይትን ኾይንኩም ብሓንሳብ ትነብሩ መናብርቲ ዶ አለውኽን?  **መልሰን እብ ሓዳር ኣይኮንኩን ወይድማ ከም ስብኣይ ዝነብር ፈታዊ የብለይን እንተድኣኾይኑ፡፡ተፋቲሐን ምኻነን፣ተፈላልየን ምኻነን ወይድማ በዓል ገዝአን ሞይቶምወን ም****ኖም ብምሕታት ንዝምለከቶ ኮድ ምልኡ፡፡** | | | | ኣይፋሉን ፣ ሓዳር ፈቲነን ዘይፈልጣ 0  እወ፣ኣብ ሓዳር ዘለዋ 1  እወ፣ምስ ስብኣይ ብሓባር ዝነብራ 2  ሐዚ ኣብ ሓዳር ዘየለዋ፣ ዝተፋተሓ/ ዝተፈላለያ 3  ሐዚ ኣብ ሓዳር ዘየለዋ፤በዓል ገዝአን ዝሞትወን 4 | | | | | | | | | | የለን፣ ሓዳር ፈቲነን ዘይፈልጣ እንተኾይኑ ናብ 8 ሕለፍ/ፊ |
| 4 | | ሓንሳብ ጥራሕ ድዩ ሓዳር ገይርክን/ከም ስብኣይን ሰበይትን ኾንኩም ምስ ወዲ ተባዕታይ ምንባር ዝጀመርክን/ ወይስ ካብ ሓደ ጊዘ ንላዕሊ? | | | | ሓንሳብ ጥራሕ 1  ካብ ሓደ ጊዘ ንላዕሊ 2 | | | | | | | | | | ሓንሳብ ጥራሕ ተኾይኑ ናብ 5a ሕለፍ/ፊ ካብ ሓደ ጊዘ ንላዕሊ ተኾይኑ ናብ 5b ሕለፍ/ፊ |
| 5a | | ምስ በዓል ገዛኽን/በዓልገዛኽን/ከም ስብኣይ ምሳኽን ዝነብሩ ፈታዊኽን ብሓንሳብ ምንባር ዝጀመርክምሉ በየናይ ወርሕን ዓመተምሕረትን እዩ? | | | | ወርሒ | | | | |  | | | | |  |
|  |  |  |  |  |  | ዓመተ­-ምሕረት | | | | |  | | | | |  |
| 5b | | ምስ ናይ ሐዚ በዓልገዛኽን/ በዓልገዛኽን/ከም ስብኣይ ምሳኽን ዝነብሩ ፈታዊኽን ብሓንሳብ ምንባር ዝጀመርኩምሉ በየናይ ወርሕን ዓመተ ምሕረትን እዩ? | | | | ወርሒ | | | | |  | | | | |  |
|  |  |  |  |  |  | ዓመተምሕረት | | | | |  | | | | |  |
| 6 | | በዓል ገዛኽን/በዓልገዛኽን/ከም ስብኣይ ምሳኽን ዝነብሩ ፈታዊኽን ካልኦት ኣንሽቲ/ከም ሰበይቶም ኾይነን ብሓንሳብ ዝነብራ ደቂ ኣንስትዮ ኣለወኦም ዶ? | | | | እወ 1  የብለይን 0  ኣይፈለጥኩን -88 | | | | | | | | | |  |
|  | | **ሕቶ 3ተ ተመልከቲ:** ኣብዚ ሐዚ እዋን ኣብ ሓዳር ድየን ዘለዋ? | | | | እወ 1  ኣይፋሉን 0 | | | | | | | | | | የለን እንተኾይኑ ናብ 8 ሕለፍ |
| 7 | | ኣብዚ ሐዚ እዋን በዓልገዛኽን/ከም ስብኣይ ምሳኽን ዝነብሩ ፈታዊኽን ምሳኽን ዲዮም ዝነብሩ ወይስ ኣብ ካልእ ቦታ እዮም ዝነብሩ? | | | | ምስ መልሲ ወሃቢት ሓቢሮም ይነብሩ 1  ኣብ ካልእ ቦታ ይነብሩ 2 | | | | | | | | | |  |
| **ካልኣይ ክፋል- ስነ ተዋልዶ፣ጥንስን ምጣነ ስድራን**  **ሐዚ ድማ ብዛዕባ ዝወለድክነኦም ኩሎም ውላድክን ብዝምልከት ዝተወሰኑ ሕቶታት ክሓተክን እየ** | | | | | | | | | | | | | | | | |
| 8 | | | | ክሳብ ሐዚ ብጠቕላላ ክንደይ ግዘ/ቆልዑ ወሊድክን?  **መልሲ 0 ክኸውን ይኽእል እዩ፡፡** | | በዝሒ ውላድ | | | | | | | |  | | 0 ተኾይኑ ናብ 13 ሕለፍ/ፊ |
|  | | | | ኩሎም ዝወለድክንኦም ቆልዑ ብሂወት ድዮም ተወሊዶም?  **ኣፋሉን እንተኾይኑ ናብ ሕቶ 8 ብምምላስ ብሂወት ዝተወለዱ በዝሒ ቖልዑ ጥራሕ ምልኡ** | | እወ 1  ኣይፋሉን 0 | | | | | | | | | |  |
| 9 | | | | ናይ መወዳእታ ውላድክን መዓዝ ወሊድክንኦ/ኣ?  **ናይ መወዳእታ ውላደን ዝተወለደሉ ወርሕን ዓመተ ምሕረትን ምልኡ።**  **ኣድላዪ እንተኾይኑ ካብ ዝሓለፉ ታሪካዊ ክስተታት ብምትሓዝ ትኽክለኛ ዕለት ንምርካብ ሞክሪ** | | ወርሒ | | | | ዓመተምሕረት | | | | | | ኣብ ዝሓለፈ ዓመት እንተዘይኮይኑን /ወይድማ ሕቶ 8 ኮድ 1 ተተመሊኡን ናብ 11 ሕለፍ/ፊ |
| 10 | | | | ቅድሚ እቲ ናይ መወዳእታ ውላድክን ዝወለድክናሉ እዋን መዓዝ እዩ?  **ቕድሚ ናይ መወዳእታ ውላድ ዝተወለደሉ/ትሉ ወርሕን ዓመተ ምሕረትን ምልኡ**  **ኣድላዪ እንተኾይኑ ካብ ዝሓለፉ ታሪካዊ ክስተታት ብምስትዋስ ትኽክለኛ ዕለት ንምርካብ ሞክሪ** | | ወርሒ | | | | ዓመተምሕረት | | | | | |  |
| 11 | | | | ናይ መወዳእታ ውላድክን ብሂወት ኣሎ/ላ ዶ? | | እወ 1  ኣይፋሉን 0  ኣይፈለጥኩን -88 | | | | | | | | | | እወ እንተኾይኑ ናብ 13 ሕለፍ/ፊ |
| 12 | | | | ናይ መወዳእታ ውላድክን መዓዝ ሞይቱ/ታ?  **ናይ መወዳእታ ውላደን ዝሞተሉ/ዝሞተትሉ ወርሕን ዓመተ ምሕረትን ምልኡ**  **ኣድላዪ እንተኾይኑ ካብ ዝሓለፉ ታሪካዊ ክስተታት ብምስትዋስ ትኽክለኛ ዕለት ንምርካብ ሞክሪ፡፡** | | ወርሒ | | | | ዓመተምሕረት | | | | | |  |
| 13 | | | | ንመወዳእታ ጊዘ ወርሓዊ ፅግያትክን ምርኣይ ዝጀመርክናሉ እዋን መዓዝ ነይሩ?  **መዓልታት፣ሰሙናት፣ኣዋርሕ ወይድማ ዓመታት እንተመሪፅኪ ቐፂሉ ኣብ ዘሎ እስክሪን X ኣብ ዝብል ቑፅሪ ፀሓፊ** | | መዓልታት፡ | | | | | |  | | | |  |
|  |  |  |  |  |  | ሰሙናት: | | | | | |  | | | |  |
|  |  |  |  |  |  | ኣዋርሕ: | | | | | |  | | | |  |
|  |  |  |  |  |  | ዓመታት: | | | | | |  | | | |  |
|  |  |  |  |  |  | ብዕድመ ምድፋእ ምኽንያት ወርሓዊ ፅግያ ምርኣይ ዘቋረፃ/ማህፀነን ብኦፕሬሽን ዘውፅኣ 1  ቅድሚ ናይ መወዳእታ ቆልዓ ምውላደን 2  ወርሓዊ ፅግያት ሪአን ዘይፈልጣ 3 | | | | | | | | | |  |
| 14 | | | | ኣብዚ ሐዚ እዋን ጥንስቲ/ነፍሰፁር ዲኽን? | | እወ 1  ኣይፋሉን 0  እርግፀኛ ኣይኮንኩን 2 | | | | | | | | | | የለን እንተኾይኑ ናብ 16 ሕለፍ/ፊ |
| 15 | | | | ናይ ክንደይ ወርሒ ጥንስቲ/ነብሰፁር ኢኽን?  **ዕድመ ጥንሲ ብሙሉእ ኣዋርሕ ምልኡ፡፡ ኣይፈልጦን እንተኾይኑ -88 ምልኡ፡፡** | | በዝሒ ኣዋርሕ | | | | | | | |  | |  |
|  | | | | **ሕቶ 14 ተመልከቲ:** ሐዚ ጥንስቲ/ነብሰፁር ድየን? | | እወ 1  ኣይፋሉን 0 | | | | | | | | | | የለን እንተኾይኑ ናብ16a ሕለፍ/ፊ እወ እንተኾይኑ  16b ሕለፍ/ፊ |
| 16a | | | | ሐዚ ድማ ብዛዕባ መፃኢ ሓሳብክን/ትልምኽን ዝተወሰኑ ሕቶታት ክሓተክን እየ ፡፡  ንመፃኢ ቆልዓ (ተወሳኺ ቆልዓ) ክትወልዳ ትደልያ ዶ ወይስ ቆልዓ (ተወሳኺ ቆልዓ) ክትወልዳ ኣይትደልያን? | | ቆልዓ/ተወሳኺ ቆልዓ ክወልዳ ይደልያ 1  ቆልዓ/ተወሳኺ ቆልዓ ክወልዳኣይደልያን 2  ክጠንሳ ኣይኽእላን 3  ኣይወሰኑን/ኣይፈለጥኩን -88 | | | | | | | | | | ንኮድ1 ጥራሕ ናብ 17a ሕለፍ/ፊ ብዝተረፈ ንኹሎም ናብ 18 ሕለፍ/ፊ |
| 16b | | | | ሐዚ ድማ ብዛዕባ መፃኢ ሓሳብክን/ትልምኽን ዝተወሰኑ ሕቶታት ክሓተክን እየ ዝደሊ፡፡  ሐዚ ጠኒስክንኦ ዘለኽን ቖልዓ ድሕሪ ምውላድክን ቆልዓ (ተወሳኺ ቆልዓ) ክትወልዳ ትደልያ ዶ ወይስ ቆልዓ (ተወሳኺ ቆልዓ) ክትወልዳ ኣይትደልያን? | | ቆልዓ/ተወሳኺ ቆልዓ ክወልዳ ይደልያ 1  ቆልዓ/ተወሳኺ ቆልዓ ክወልዳኣይደልያን 2  ክጠንሳ ኣይኽእላን 3  ኣይወሰኑን/ኣይፈለጥኩን -88 | | | | | | | | | | ንኮድ1 ጥራሕ ናብ 17b ሕለፍ/ፊ ብዝተረፈ ንኹሎም ናብ 18 ሕለፍ/ፊ |
| 17a | | | | ድሕሪ ሐዚ ቆልዓ (ተወሳኺ ቆልዓ) እንተይወለድክን ንኽንደይ እዋን ክትፀንሓ ትደልያ?  **ኣዋርሕ ወይ ድማ ዓመታት እንተመሪፅኪ ቐፂሉ ኣብ ዘሎ እስክሪን X ኣብዝብል ቑፅሪ ፀሓፍ/ፊ፡፡** | | ኣዋርሕ: | | | | | | | |  | |  |
|  |  |  |  |  |  | ዓመታት: | | | | | | | |  | |  |
|  |  |  |  |  |  | ኣብ ቀረባ እዋን 1  ካልእ 2  ክጠንሳ ኣይኽእላን 3  ኣይፈለጥኩን -88 | | | | | | | | | |  |
| 17b | | | | ጠኒስክንኦ ዘለኽን ቖልዓ ድሕሪ ምውላድክን ቆልዓ (ተወሳኺ ቆልዓ) እንተይወለድክን ንኽንደይ እዋን ክትፀንሓ ትደልያ?  **ኣዋርሕ ወይድማ ዓመታት እንተመሪፅካ/ኪ ቐፂሉ ኣብ ዘሎ እስክሪን X ኣብዝብል ቑፅሪ ፀሓፍ/ፊ፡፡** | | ኣዋርሕ: | | | | | | | |  | |  |
|  |  |  |  |  |  | ዓመታት: | | | | | | | |  | |  |
|  |  |  |  |  |  | ኣብ ቀረባ እዋን 1  ካልእ 2  ክጠንሳ ኣይኽእላን 3  ኣይፈለጥኩን -88 | | | | | | | | | |  |
|  | | | | **ሕቶ 8 ተመልከቲ:** ዝወለድኦም በዝሒ ቖልዑ  **ሕቶ 14 ተመልከቲ**: ሐዚ ነብሰፁር ዲየን? | | ዝወለድኦም በዝሒ ቖልዑ | | | | | | | |  | | ቖልዓ ወሊደን ንዘይፈልጣን ንሕ14 መልሲ ኣፋሉን እንተኾይኑ ናብ 19 ዝለሊ ወሊደን ንዝፈልጣን ንሕ14 መልሲ ኣይፋሉን እንተኾይኑ ናብ ሕ18a ዝለሊ ወሊደን ንዝፈልጣን ንዘይፈልጣን  14 እወ እንተኾይኑ ናብ18b ዝለሊ |
|  |  |  |  |  |  | እወ 1  ኣይፋሉን 0 | | | | | | | | | |  |
| 18a | | | | **ሐዚ ድማ ብዛዕባ ናይ መወዳእታ ውላድክን ብዝምልከት ዝተወሰኑ ሕቶታት ክሓተክን እየ**  ናይ መወዳእታ ውላድክን ክትጠንሳ ከለኽን ኣብቲ ሽዑ እዋን ንኽትጠንሳ ድልየት ነይሩክን ዶ ፣ ፀኒሕኽን ቆልዓ (ተወሳኺ ቆልዓ) ንኽትወልዳ ድዩ ነይሩ ድልየትክን ወይስ ፈፂምክን ቆልዓ/ተወሳኺ ቆልዓ ክትወልዳ ኣይትደልያን ነይርክን? | | ሽዑ ድልየት ነይሩኒ 1  ፀኒሐ ክወልድ ድልየት ነይሩኒ 2  ፈፂመ ክወልድ ድልየት ኣይነበረንን 3 | | | | | | | | | |  |
| 18b | | | | **ሐዚ ድማ ብዛዕባ ናይ ሐዚ ጥንስኽን ብዝምልከት ዝተወሰኑ ሕቶታት ክሓተክን እየ**  ነዚ ጥንሲ ክትጠንሳ ተለኽን ኣብቲሽዑ እዋን ንኽትጠንሳ ድልየት ነይሩክን ዶ፣ ፀኒሕኽን ንኽትጠንሳ ድዩ ነይሩ ድልየትክን ወይስ ፈፂምክን ቆልዓ/ተወሳኺ ቆልዓ ክትወልዳ ኣይትደልያን ነይርክን? | | ሽዑ ድልየት ነይሩኒ 1  ፀኒሐ ክወልድ ድልየት ነይሩኒ 2  ፈፂመ ክወልድ ድልየት ኣይነበረንን 3 | | | | | | | | | |  |
| **ሳልሳይ ክፋል -ሜላታት መከላኸሊ ጥንሲ**  **ሐዚ ድማ ንስኽን ወይ ድማ በዓልገዛኽን/ፍቕረኛኽን ጥንሲ ንምክልኻል ኢልኩም ብዛዕባ ዝተጠቐምኩምዎም ሜላ መከላኸሊ ጥንሲ ብዝምልከት ዝተወሱ ሕቶታት ክሓተክን እየ፡፡** | | | | | | | | | | | | | | | | |
| 19 | | | ጥንሲ ንምክልኻል ወይድማ ንምዝንጋዕ ኢልክን ዝኾነ ዓይነት ሜላ መከላኸሊ ጥንሲ ተጠቒምክን/ ፈቲንክን ዶ ትፈልጣ? | | | እወ 1  ኣይፋሉን 0 | | | | | | | | | | የለን ተኾይኑ ናብ 25 ሕለፍ/ፊ |
| 20 | | | ንፈለማ እዋን ሜላ መከላኸሊ ጥንሲ ምጥቃም ክትጅምራ ከለኽን ጓል ክንደይ ዓመት ነይርክን?  **ዕድመ ብሙሉእ ዓመት ይመላእ፡፡ ዝኾነ ዓይነት ሜላ ተጠቒመን ዘይፈልጣ ተኾይነን 0 ዘይፈልጠኦ ተኾይነን ድማ -88 ይመላእ፡፡** | | | ዕድመ | | | |  | | | | | |  |
| 20a | | | ኣብቲ ሽዑ እዋን ኽንደይ ቖልዑ ወሊድክን ነይርክን? | | | በዝሒ ውላድ | | | |  | | | | | |  |
| 21 | | | ንፈለማ እዋን ዝተጠቐምክንኦ ሜላ መከላኸሊ ወይድማ መዘንግዒ ጥንሲ አየናይ ነይሩ ?  **ኣብ መማረፂ ዝተዘርዘሩ ዓይነት ሜላ መከላኸሊ ኣይተንብብለን፡፡**  **ኩሎም መማረፅታት ንምርኣይ ናብ ታሕቲ ውረዲ፡፡** | | | ደቂ ኣንስትዮ ብኦፕሬሽን ምምካን 1  ደቂ ተባዕትዮ ብኦፕራሽን ምምካን 2  በማህፀን የሚወቀበር ሉፕ 3  መርፍእ 4  ኣብ ኩርናዕ ዝቕበር ኢምፕላነትስ/ኢመፕላኖን 5  መከላኸሊ ጥንሲ ክኒን (ፒል) 6  ኮነደም 7  ናይ ጓል ኮነደም 8  ድንገተኛ መከላኸሊ ጥንሲ ክኒን 9  ስታንዳርድ ምቑፃር መዓልቲ /ዕንቒ ዝመስልነገር ብምጥቃም ዑደት ወርሓዊ ፅግያ ምክትታል 10  ህፃን ጡብ ኣዶ ብቻ ምጥባው (ላም) 11  ካልኦት ዘመናዊ ሜላ መከላኸሊ …...12  ምቑፃር መዓልቲ 13  ዘርኢ ወዲተባዕታይ ካብ ብልዕቲ ጓል ተንስተይቲ ወፃኢ ምፍሳስ 14  ካልኦት ባህላዊ ሜላ መከላኸሊ 15 | | | | | | | | | |  |
|  | | | **ሕቶ 14 ተመልከቲ:** ኣብዚ ሐዚ እዋን ነፍሰፁር ድየን? | | | እወ 1  ኣይፋሉን 0 | | | | | | | | | | እወ ተኾይኑ ናብ 25 ዝለሊ |
| 22 | | | ኣብዚ ሐዚ እዋን ንስኽን ወይድማ በዓልገዛኽን/ በዓልገዛኽን/ከም ስብኣይ ምሳኽን ዝነብሩ ፈታዊኽን ጥንሲ ንምክልኻል ወይድማ ንምዝንጋዕ ኢልኩም ትጥቀምዎ ዝኾነ ዓይነት ሜላ መከላኸሊ ጥንሲ ኣሎ ዶ? | | | እወ 1  ኣየፋሉን 0 | | | | | | | | | | ኣይፋሉን ተኾይኑ ናብ 25 ዝለሊ |
| 23 | | | አየናይ ሜላ መከላኸሊ ጥንሲ ኢኹም ትጥቀሙ ዘለኹም?  ካልእ ኸ?  **ዝገለፀኦም ኩሎም ሜላ መከላኸሊ ጥንሲ ይመላእ፡፡**  **ኩሎም መማረፅታት ንምርኣይ ናብ ታሕቲ ውረዲ፡፡** | | | 1. ደቂ ኣንስትዮ ብኦፕሬሽን ምምካን  2. ደቂ ተባዕትዮ ብኦፕራሽን ምምካን  3. በማህፀን የሚወቀበር ሉፕ  4. መርፍእ  5. ኣብ ኩርናዕ ዝቕበር ኢምፕላነትስ/ኢመፕላኖን  6. መከላኸሊ ጥንሲ ክኒን (ፒል)  7. ኮንደም  8. ናይ ጓል ኮንደም  9. ድንገተኛ መከላኸሊ ወሊድ  10. ስታንዳርድ ምቑፃር መዓልቲ /ዕንቒ ዝመስልነገር ብምጥቃም ዑደት ወርሓዊ ፅግያ ምክትታል  11. ህፃን ጡብ ኣዶ ብቻ ምጥባው  12. ካልኦት ዘመናዊ ሜላ መከላኸሊ  13. ምቑፃር መዓልቲ  14. ዘርኢ ወዲተባዕታይ ካብ ብልዕቲ ጓል ተንስተይቲ ወፃኢ ምፍሳስ  15 ካልኦት ባህላዊ ሜላ መከላኸሊ | | | | | | | | እ  1  1  1  1  1  1  1  1  1  1  1  1  1  1  1  1  1 | የለ  0  0  0  0  0  0  0  0  0  0  0  0  0  0  0  0  0 | አዐርዩ ውፅኢታዊ ዝኾነ ሜላ መከላኸሊ (ብመሰረት ቅደም ሰዓብ መጀመርታ ዝርከብ) ናይ ሕለፍ ትእዘዝ ተኸተሉ  ካብ 3-17 ዘለው ተመሪፅኩም  ናብ 29 ሕለፍ/ፊ |
| 24 | | | እቲ በዓል ሞያ ጥዕና ቐዋሚ ሜላ መከላኸሊ ጥንሲ ምኑ ንዓኽን ወይድማ ንበዓልገዛኽን/ፍቕረኛኽን ነጊሮምኹም ዶ ነይሮም? | | | እወ 1  ኣይፋሉን 0 | | | | | | | | | | ንኽሎም ናብ 29 ሕለፍ/ፊ |
| 25 | | | ሜላ መከላኸሊ ጥንሲ/ምጣነ ስድራ ኣበይ ክርከብ ከምዝኽእል ትፈልጣ ዶ? | | | እወ 1  ኣይፋሉን 0 | | | | | | | | | |  |
|  | | | **ሕቶ 14 ተመልከቲ**: ሐዚ ነብሰፁር/ጥንስቲ ዲየን? | | | እወ 1  ኣይፋሉን 0 | | | | | | | | | | የለን እንተኾይኑ ናብ 26a ሕለፍ/ፊ  እወ እንተኾይኑ ናብ  26b ሕለፍ/ፊ |
| 26a | | | ኣብዚ ሐዚ እዋን ሜላ መከላኸሊ ጥንሲ ከምዘይትጥቀማ ነጊርክናኒ ነይርክን ንሓዋሩ ኣብ ዝኾነ ይኹን እዋን ‘ከ ጥንሲ ንምክልኻል ወይድማ ንምዝንጋዕ ኢልክን ሜላ መክላኸሊ ጥንሲ ንምጥቃም ትሓስባ ዶ? | | | እወ 1  ኣይፋሉን 0 | | | | | | | | | |  |
| 26b | | | ንሓዋሩ ኣብ ዝኾነ ይኹን እዋን ጥንሲ ንምክልኻል ወይድማ ንምዝንጋዕ ኢልክን ሜላ መክላኸሊ ጥንሲ ንምጥቃም ትሓስባ ዶ? | | | እወ 1  ኣይፋሉን 0 | | | | | | | | | |  |
|  | | | **ሕቶ 19 ተመልከቲ**: ሜላ መከላኸሊ (መዘንግዒ) ጥንሲ ተጠቒምክን ትፈልጣ ዶ? | | | እወ 1  ኣይፋሉን 0 | | | | | | | | | | ኣይፋሉን እንተኾይኑ ናብ 43 ሕለፍ/ፊ |
| 27 | | | ኣብ ዝሓለፉ 12 ኣዋርሕ ጥንሲ ንምክልኻል ወይድማ ንምዝንጋዕ ኢልክን ዝኾነ ዓይነት ሜላ መከላኸሊ ጥንሲ ተጠቒምክን ዶ ትፈልጣ? | | | እወ 1  ኣይፋሉን 0 | | | | | | | | | | ኣየፋሉን እንተኾይኑ ናብ 43 ሕለፍ/ፊ |
| 28 | | | ንመወዳእታ ግዘ/ ኣብዚ ሐዚ እዋን ዝተጠቐምክንኦ ሜላ መከላኸሊ (መዘንግዒ) ጥንሲ ኣየናይ እዩ?  ካልእ ኸ?  **አዐርዩ ውፅኢታዊ ዝኾነ ሜላ መከላኸሊ** (**ብኣቀማምጣ ቅደም ሰዓብ መሰረት ዝሓሸ ሜላ) ምልኡ፡፡**  **ኩሎም መማረፅታት ንምርኣይ ናብ ታሕቲ ውረዲ** | | | 1. ደቂ ኣንስትዮ ብኦፕሬሽን ምምካን  2. ደቂ ተባዕትዮ ብኦፕራሽን ምምካን  3. በማህፀን የሚወቀበር ሉፕ  4. መርፍእ  5. ኣብ ኩርናዕ ዝቕበር ኢምፕላነትስ/ኢመፕላኖን  6. መከላኸሊ ጥንሲ ክኒን (ፒል)  7. ኮንደም  8. ናይ ጓል ኮንደም  9. ድንገተኛ መከላኸሊ ወሊድ  10. ስታንዳርድ ምቑፃር መዓልቲ /ዕንቒ ዝመስልነገር ብምጥቃም ዑደት ወርሓዊ ፅግያ ምክትታል  11. ህፃን ጡብ ኣዶ ብቻ ምጥባው  12. ካልኦት ዘመናዊ ሜላ መከላኸሊ  13. ምቑፃር መዓልቲ  14. ዘርኢ ወዲተባዕታይ ካብ ብልዕቲ ጓል ተንስተይቲ ወፃኢ ምፍሳስ  15 ካልኦት ባህላዊ ሜላ መከላኸሊ | | | | | | | | | |  |
| 29 | | | ሐዚ**/**ኣብ ቀረባ እዋን እትጥቀማሉ ዘለኽን **ሜላ መከላኸሊ ጥንሲ** ምጥቃም ዝጀመርክን ካብ መዓዝ ወርሕን ዓመተምሕረትን ጀሚሩ እዩ?  **ምጥቃም ዝጀመራሉ ወርሕን ዓመተምሕረትን ይመላእ፡፡**  **ኣድላዪ እንተኾይኑ ቃለ መሕትት ካብ ዝተገበሩ ወርሒ ጀሚርኪ ናብ ድሕሪት ተመሊስኪ ዝነበሩ ክስተታት ንኸስታውስኦ ብምግባር ምጥቃም ዝጀመርኦ ትኽክለኛ ወርሒ ብምርካብ ምልኢ፡፡** | | | ወርሒ | | | | ዓመተምሕረት | | | | | |  |
|  | | | **ሕቶ 22 ተመልከቲ:** ኣብዚ ሐዚ እዋን ሜላ መከላኸሊ (መዘንግዒ) ጥንሲ ይጥቀማ ዶ ኣለዋ? | | | እወ 1  ኣየፋሉን 0 | | | | | | | | | | እወ እንተኾይኑ ናብ 32 ሕለፍ/ፊ |
| 30 | | | **ንመወዳአታ እዋን ዝጥቀማሉ ዝነበራ ሜላ መከላኸሊ ጥንሲ** ምጥቃም ዘቋርፀኦ ግዘ መዓዝ ነይሩ?  **ምጥቃም ዘቋርፃሉ ወርሕን ዓመተ ምሕረትን ይመላእ፡፡**  **ኣድላዪ እንተኾይኑ ካብ ዝሓለፉ ታሪካዊ ክስተታት ብምስትዋስ ትኽክለኛ ዕለት ንምርካብ ሞክሪ** | | | ወርሒ | | | | ዓመተምሕረት | | | | | |  |
| 31 | | | **ንመወዳአታ እዋን ዝጥቀማሉ ዝነበራ ሜላ መከላኸሊ ጥንሲ** ምጥቃም ዘቋርፃሉ ምኽንያት እንታይ ነይሩ | | | በዓል ገዝአን ስለዘየለ/ ሓልሓሊፉ ጥራሕ ፆታዊ ርክብ ስለዝፍፅማ 1  ኣብ ዝሓለፈ ሜላ መከላኸሊ ጥንሲ እንዳተጠቐሙ እንዳሃለው ስለ ዝጠነሳ 2  ክጠንስ ስለ ዝደለኹ 3  በዓል ሓዳረን (ፍቕረኘአን) ስል ዝቃወሙ 4  ካልእ ብጣዕሚ ዝሐሸ ሜላ ስለ ዝደለዩ 5  ዝደልየኦ ዓይነት ሜላ ሰለ ዘየለ 6  ንጥዕነአን ብምሕሳብ 7  ዘምፅኦ ሳዕብየን ብምፍራሕ 8  ግልጋሎት ሜላ መከላኸሊ ስለዘየለ/ርሑቕ ስለዝኾነ 9  ዋግኡ ክባር ስለዝኾነ 10  ንኣጠቓቕማ ኣመቺ ስለዘይኮነ 11  ከመውት እየ/ክቐትለኒ እዩ ኢለን ብምሕሳብ 12  ዕድመ ዝሓለፈን/ብዕድመ ምኽንያት ምውላድ ዘቋረፃ ስለዝኾና 13  ከይዲ ተፈጥሮ ሰውነት ስለዘዛብዑ 14  ካለእ 15  ኣይፈለጥኩን 88 | | | | | | | | | |  |
| 32 | | | **ሐዚ/ንመወዳእታ ግዘ ዝተጠቐምኦ ሜላ መከላኸሊ ጥንሲ** ምጥቃም ክትጅምራ እንተለኽን ሽዑ እዋን ዝረኸብክንኦ ካበይ ነይሩ?  **ዝተጠቐምኦ ዓይነት ሜላ ካብ ሕቶ 28 ብምውሳድ ኣብቲ ቕንፍ ብምእታው ሕተቲ፡፡**  **ኩሎም መማረፅታት ንምርኣይ ናብ ታሕቲ ውረዲ፡፡** | | | መንግስታዊ ትካላት ጥዕና  መንግስታዊ ሆስፒታል 1  መንግስታዊ ጥዕና ጣብያ 2  መንግስታዊ ጥዕና ኬላ 3  ክሊኒክ መጣነ ስድራቤት 4    ናይ ግሊ ትካላት ጥዕና  ናይግሊ ሆስፒታል/ክልኒክ 5    ፋርማሲ 6    ካልኦት ምንጪ  ድንኳን/ዕዳጋ 7    ጉጅለ ልምዓት ጥዕና (ልምዓት ሰራዊት ደቂ ኣንስትዮ) 8  መሓዛ/ዘመድ 9  መንግስታዊ ዘይኮነ ግብረ ሰናይ ድርጅት 10  ካልእ 11  ኣይፈለጥኩን -88 | | | | | | | | | |  |
| 33 | | | ኣብ ዝሓለፉ 12 ኣዋርሕ ን(**ሐዚ/ኣብ ቀረባ እዋን) ዝጥቀማሉ ዝነበራ ሜላ መከላኸሊ ጥንሲ** ሓዊሱ ግልጋሎት ሜላ መከላኸሊ ጥንሲ/ምጣነ ስድራ ንርካብ ኢልክን ዝኸፈልክንኦ ክፍሊት ዶ ነይሩ? | | | እወ 1  ኣይፋሉን 0 | | | | | | | | | | ኣይፋሉን እንተኾይኑ ናብ 35 ሕለፍ/ፊ |
| 34 | | | ዝኸፈልክንኦ ኽንደይ ዘኣክል ገንዘብ ነይሩ?  **ዝኸፈልኦ መጠን ብብር መዝግቢ፡፡መልሲ ወሃቢት ኣይፈልጦን ዝብላ እንተኾይነን -88 ምልኡ፡፡** | | | ክፍሊት ብብር: | | | | | | | | | |  |
| 35 | | | **ሐዚ/ኣብ ቀረባ እዋን ዝጥቀማሉ ዝነበራ ሜላ መከላኸሊ ጥንሲ** ምጥቃም ኣብ ትጅምራሉ እዋን ግልጋሎት ዝሃቡኽን በዓል ሞያ ጥዕና ናይቲ ሜላ መከላኸሊ ጥንሲ ጎናዊ ፀገማት ወይድማ ከስዕቦ ዝኽእል ሳዕቤን እንታይ ከም ዝኾነ ነጊሮምኽን ዶ ነይሮም? | | | እወ 1  ኣይፋሉን 0 | | | | | | | | | | ኣፋሉን እንተኾይኑ ናብ 37 ሕለፍ/ፊ |
| 36 | | | ጎናዊ ፀገማት ኣብ ዘጓንፈክን እዋን እንታይ ክትገብራ ከም ዘለክን ነጊሮምኽን ዶ ነይሮም? | | | እወ 1  ኣይፋሉን 0 | | | | | | | | | |  |
| 37 | | | ን(**ሐዚ/ኣብ ቀረባ እዋን) ዝጥቀማሉ ዝነበራ ሜላ መከላኸሊ ጥንሲ** ካብ እትጥቀማሉ ወፃኢ ካልኦት መማረፅታት ሜላ መከላኸሊ ጥንሲ ክትጥቀማ ከም ትኽእላ ከም መማረፂ ነጊሮምኽን ዶ ነይሮም? | | | እወ 1  ኣይፋሉን 0 | | | | | | | | | |  |
| 38 | | | ኣብቲ ሽዑ ንምጥቃም ኣብ ዝኸድክናሉ እዋን ዝደለኽነኦ ዓይነት ሜላ መከላኸሊ ጥንሲ ረኺብክን ዶ? | | | እወ 1  ኣይፋሉን 0 | | | | | | | | | | እወ እንተኾይኑ ናብ 40 ሕለፍ/ፊ |
| 39 | | | እንድሕር ዘይረኺብክን፣ ዘይረኽብክናሉ ምኽንያት እንታይ ነይሩ? | | | ኣብቲ ሽዑ መዓልቲ ዝደለኽዎ ሜላ መከላኸሊ ስለዝተወደአ 1  ሜላ መከላኸሊ ፈፂሙ ስለዘይነበረ 2  እቶም በዓል ሞያ ጥዕና ብዛዕባ እቲ ዝደለኽዎ ሜላ መከላኸሊ ስልጠና ስለዘይወሰዱ 3  እቶም በዓል ሞያ ጥዕና ካልኦት ሜላ መከላኸሊ ንኽጥቀም ስለዝመዓዱኒ 4  ሜእቲ ሜላ መከላኸሊ ንምውሳድ ብቑዕ ስለዘይነበርኩ 5  እቲ ሜላ መከላኸሊ ንኸይወስድ ስለወሰንኩ 6  ዋግኡ ክባር ስለዝኾነ 7  ካልእ 8 | | | | | | | | | |  |
| 40 | | | ኣብቲ ሽዑ እዋን እንታይ ዓይነት ሜላ መከላኸሊ ጥንሲ ክትወስዳ/ክትጥቀማ ከም ዘለክን ናይ መወዳእታ ውሳነ ዝሃበ መን ነይሩ? | | | ተቃሚት ባዕለን 1  በዓል ሞያ ጥዕና 2  በዓልገዝአን/ፍቕረኝአን 3  ተቃሚት ምስ በዓል ሞያ ጥዕናን ብሓባር 4  ተጠሚት ምስ በዓልገዝአንን/ፍቕረኝአንን ብሓባር 5  ካልእ 6 | | | | | | | | | |  |
|  | | | **ሕቶ 32 ተመልከቲ:** **ሐዚ/ኣብ ቀረባ እዋን ዝጥቀማሉ ዝነበራ ሜላ መከላኸሊ ጥንሲ** ካበይ ረኺበንኦ?  **ዝተጠቐምኦ ዓይነት ሜላ ካብ ሕቶ 28 ብምውሳድ ኣብቲ ቕንፍ ብምእታው ሕተቲ፡፡**  **ኩሎም መማረፅታት ንምርኣይ ናብ ታሕቲ ውረዲ፡፡** | | | መንግስታዊ ትካላት ጥዕና  መንግስታዊ ሆስፒታል 1  መንግስታዊ ጥዕና ጣብያ 2  መንግስታዊ ጥዕና ኬላ 3  ክሊኒክ መጣነ ስድራቤት 4    ናይ ግሊ ትካላት ጥዕና  ናይግሊ ሆስፒታል/ክልኒክ 5    ፋርማሲ 6    ካልኦት ምንጪ  ድንኳን/ዕዳጋ 7    ጉጅለ ልምዓት ጥዕና (ልምዓት ሰራዊት ደቂ ኣንስትዮ) 8  መሓዛ/ዘመድ 9  መንግስታዊ ዘይኮነ ግብረ ሰናይ ድርጅት 10  ካልእ 11  ኣይፈለጥኩን -88 | | | | | | | | | | ኣብ ሕቶ 32 ካብ 14-17 ዘለው መማረፅታት ተተመሪፁ ናብ 44 ሕለፍ/ፊ |
| 41 | | | ናብቲ ግልጋሎት ዝረኸብክናሉ ትካል/በዓል ሞያ ጥዕና ንመፃኢ ተመሊስክን ትኸዳ ዶ? | | | እወ 1  ኣይፋሉን 0 | | | | | | | | | |  |
| 42 | | | ናብቲ ግልጋሎት ዝረኸብክናሉ ትካል/በዓል ሞያ ጥዕና ቤተሰብክን ወይድማ መሓዙትክን ከይደን ንኽጥቀማ ትምዕዳ ዶ? | | | እወ 1  ኣይፋሉን 0 | | | | | | | | | |  |
|  | | | **ሕቶ 16 ተመልከቲ:**ንመፃኢ ቖልዓ/ተወሳኺ ቖልዓ ክህልወን ይደልያ ዶ?  **ሕቶ 17 ተመልከቲ**:ቖልዓ/ተወሳኺ ቖልዓ እንተይወለዳ ክልተ ዓመትን ካብኡ ንላዕልን ክፀንሓ ዶ ይደልያ  **ሕቶ 22 ተመልከቲ:**ኣብዚ ሐዚ እዋን ዝኾነ ዓይነት ሜላ መከላኸሊ ጥንሲ ይጥቀማለዋ ዶ  **ሕቶ 19 ተመልከቲ:** ኣብ ዝኾነ ይኹን እዋን ሜላ መከላኸሊ ጥንሲ ተጠቒመን ዶ ይፈልጣ? | | | ቆልዓ/ተወሳኺ ቆልዓ ክወልዳ ይደልያ 1  ቆልዓ/ተወሳኺቆልዓ ክወልዳ ኣይደልያን 2  ክጠንሳ ኣይኽእላን 3  ኣይወሰንኩን/ኣይፈለጥኩን -88 | | | | | | | | | | ንሓዋሩ  ቆልዓ/ተወሳኺ ቆልዓ ቅድሚ ኽልተዓመት ክወልዳ ንዘይደልያ ከምኡድማ መከላኸሊ ጥንሲ ሐዚ ይኹን ቅድሚ ሐዚ ተጠቒመን ንዘይፈልጣ ሕቶ 43 ሕተቲ፡፡ |
|  |  |  |  |  |  | ቆልዓ/ተወሳኺቆልዓ ክወልዳ ኣይደልያን 1  ቅድሚ ክልተ ዓመት ክወልዳ ይደልያ 2  2 ዓመትን ካብኡ ንላዕልን ፀኒሐን ክወልዳ ይደልያ 3 | | | | | | | | | |  |
|  |  |  |  |  |  | እወ፣ መከላኸሊ ይጥቀማ ኣለዋ 1  ኣይፋሉን፣መከላኸሊ ኣይጥቀማን ዘለዋ 0 | | | | | | | | | |  |
|  |  |  |  |  |  | እወ 1  ኣይፋሉን 0 | | | | | | | | | |  |
| 43 | | | ንሓዋሩ ቖልዓ (ተወሳኺ ቖልዓ) ከምዘይትደልያ ከምኡ ድማ ሜላ መከላኸሊ ጥንሲ ቅድሚ ሐዚ ይኹን ኣብዚ ሐዚ እዋን ከምዘይትጥቀማ ነጊርክናኒ ኣለኽን፡፡  ሜላ መከላኸሊ ጥንሲ ዘይትጥቀማሉ ቐንዲ ምኽንያት እንታይ ከምዝኾነ ክትነግራኒ ዶ ትኽእላ? | | | ሓልሓሊፉ ጥራሕ ፆታዊ ርክብ ስለዝፍፅማ/ በዓል ገዝአን ኣብ ርሑቕ ቦታ ስለ ዘሎ 1  ማህፀነን ብኦፕሬሽን ዘገልበጣ/ ብዕድመ ምውላድ ዘቋረፃ(ብዕድመ ምኽንያት) 2  መኻን/ክጠንሳ ስለዘይኽእላ 3  ድሕሪ ናይ መወዳእታ ውላድ ወርሓዊ ፅግያት ዘይምርኣይ 4  ጡብ ስለ ዘጥብው 5  ናይ ፈጣሪ ፍቓድ ዝብሉ 6  መልሲ ወሃቢት ስለዝቃወማ 7  በዓል ገዛ/ፍቕረኛ ስለዝቃወም 8  ካልኦት ስለዝቃወሙ 9  ብሃይማኖት ስለዘይፈቅድ 10  ዝኾነ ዓይነት ሜላ ዝፈልጠኦ የለን 11  ካበይ ከም ዝርከብ ዘይምፍላጥ 12  ጎናዊ ፀገማት ወይም ካልኦት ሳዕብየናት ሜላ ጥንሲ ምክልኻል ብምፍራሕ 13  ንጥነአንና ብምሕሳብ 14  ግልጋሎት ሜላ መከላኸሊ ስለዘየለ/ርሑቕ ስለዝኾነ 15  ዋግኡ ክባር ስለዝኾነ 16  ዝደልዮ ዓይነት መከላኸሊ ስለዘየለ 17  ዝኾነ ዓይነት ሜላ መከላኸሊ ስለ ዘየለ 18  ንኣጠቓቕማ ኣመቺ ስለዘይኮነ 19  ከይዲ ተፈጥሮ ሰውነት ስለዘዛብዕ 20  ካልእ 21  ኣይፈለጥኩን -88 | | | | | | | | | |  |
| 44 | | | ኣብ ዝሓለፉ 12 ኣዋርሕ ብዝዕባ ምጣነ ስድራ/ሜላ መከላኸሊ ጥንሲ ንምዝርራብ ኢሉ ናብ ገዛኽን ዝመፀ ዝኾነ ሰብ/በዓል ሞያ ጥዕና ነይሩ ዶ? | | | እወ 1  ኣይፋሉን 0 | | | | | | | | | |  |
| 45 | | | ኣብ ዝሓለፉ 12 ኣዋርሕ ብዛዕባ ኩነታት ጥዕናኽን (ጥዕና ውላድክን) ንምክትታል/ንምፍላጥ ኢልክን ናብ ትካላት ጥዕና ኼድክን ዶ ነይርክን? | | | እወ 1  ኣይፋሉን 0 | | | | | | | | | | የለን እንተኾይኑ ናብ 47 ሕለፍ/ፊ |
| 46 | | | ኣብቲ እዋን እቲ ኣብ ውሽጢ ትካል ጥዕና ዘሎ ዝኾነ ይኹን በዓል ሞያ ጥዕና ብዛዕባ ምጣነ ስድራ/ሜላመከላኸሊ ጥንሲ ኣዘራሪቦምኽን ዶ ነይሮም? | | | እወ 1  ኣይፋሉን 0 | | | | | | | | | |  |
| 47 | | | ኣብ ዝሓለፉ ውሕዳት ኣዋርሕ ብዛዕባ ምጣነ ስድራ/ሜላ መከላኸሊ ጥንሲ ዝተመሓላለፉ ዝኾኑ ዓይነት መልእኽታት፡ ብራድዮ ሰሚዕኽን ዶ ነይርክን? ካብ ተሌቪዥን ርኢኽን ዶ ነይርክን? ካብ ጋዜጣ ወይድማ መጋዝን ኣንቢብክን ዶ ነይርክን? | | |  | | | | እወ  1  1  1 | | | | ኣይ  0  0  0 | |  |
| 48 | | | ንመጀመርታ ግዘ ፆታዊ ርክብ ክትፍፅማ ከለኽን (ፈፂመን ዝፈልጣ እንተኾይነን) ጓል ክንደይ ዓመት ነይርክን?  **ዕድመ ብሙሉእ ዓመት ይመላእ፡፡ፈፂመን ዘይፈልጣ እንተኾይነን 0 ይመላእ፡፡ንመጀመርታ ግዘ ፆታዊ ርክብ ክፍፅማ ከለዋ ዕድመአን ዘይፈልጠኦ እንተኾይነን-88 ይመላእ፡፡** | | | ዕድመ | | | |  | | | | | | 0 እንተኾይኑ ናብ 50 ሕለፍ/ፊ |
| 49 | | | ንመወዳእታ ግዘ ፆታዊ ርክብ መዓዝ ፈፂምክን?  **12 ወርሕን ካብኡ ንላዕልን እንተኾይኑ ዓመት ኣብ ዝብል ይመላእ፡፡ ትሕቲ 12 ወርሒ እንትኾይኑ ብመዓልታት፣ሰሙናት ወይድማ ኣዋርሕ ይመላእ፡፡**  **ሎመዓንቲ እንተኾይኑ 0 ይመላእ**  **ቐፂሉ ኣብ ዘሎ እስክሪን ድማ X ኣብ ዝብል ቑፅሪ ይመላእ፡፡** | | | መዓልታት | | ሰሙናት | | ኣዋርሕ | | | | ዓመታት | |  |
| **ራብዓይ ክፋል፡ ኣጠቓቕማ ማይ**  **ሐዚ ድማ ኣብ ማይ ኣጠቓቕማ ዘለክን ልምዲ ብዝምልከት ዝተወሰኑ ሕቶታት ክሓተክን እየ** | | | | | | | | | | | | | | | | |
| 50 | | ኣብ እዋን ሓጋይ ማይ ንምሕዋር/ቐዲሕኽን ንምምላስ ኣብ መዓልቲ ክንደይ ሰዓት ይወስደልክን?  **መልሲ ወሃቢት ማይ ንምሕዋር ዝወደአለን ሰዓት ጥራሕ ይመላእ፡፡ካልኦት ሰባት ንምሕዋር ዝወስደሎም ሰዓት ክምላእ የብሉን**  **ሰዓት ወይድማ ደቒቓ እንተመሪፅካ/ኪ ቐፂሉ ኣብ ዘሎ እስክሪን X ንዝብል ቑፅሪ ፅሓፊ፡፡** | | | ብመዓልቲ ዝወስደለን ሰዓት | | | | | | | | |  | |  |
|  |  |  |  |  | ማይ ዝሓውራ ካልኦት እየን 33  ማይ ዝሓውር የብልናን 44  ኣይፈለጥኩን -88 | | | | | | | | | | |  |
| 51 | | ኣብ እዋን ክረምቲ ማይ ንምሕዋር ኣብ መዓልቲ ክንደይ ሰዓት ይወስደልክን?  **መልሲ ወሃቢት ማይ ንምሕዋር ዝወደአለን ሰዓት ጥራሕ ይመላእ፡፡ካልኦት ሰባት ንምሕዋር ዝወስደሎም ሰዓት ክምላእ የብሉን**  **ሰዓት ወይድማ ደቒቓ እንተመሪፅካ/ኪ ቐፂሉ ኣብ ዘሎ እስክሪን X ንዝብል ቑፅሪ ፅሓፍ/ፊ** | | | ብመዓልቲ ዝወስደን ሰዓት | | | | | | | | |  | |  |
|  |  |  |  |  | ማይ ዝሓውራ ካልኦት እየን 33  ማይ ዝሓውራ የብልናን 44  ኣይፈለጥኩን -88 | | | | | | | | | | |  |
| **መልሲ ወሃቢት ንዝሃባኻ/ኺ ግዘ ኣመስጊንካ/ኪ ተሰናበት/ቲ፡፡**  **T**  **መረዳእታ ሰብሳቢት፡ ንመልሲ ወሃቢት ዝሕተቱ ሕቶታት ወዲእኺ ኣለኺ። እንተኮነ ግና ዝስዕቡ ከልተ ሕቶታት ብኣኺ ዝምልኡ ኣለው።** | | | | | | | | | | | | | | | | |
| **ቦታ** | | | | | | | | | | | | | | | | |
| M | | **ጥቓ ኣፍደገ መልሲ ወሃቢት ብም**  **ን ንባብ ጂፒኤስ (ሰሜን ምስራቕ) ውሰዲ፡፡**  **ናይቲ ጂፒኤስ ኣኩራሲይ ትሕቲ 6 ሜ ምስኮነ ንባብ ይወሰድ፡፡** | | | | *መምርሒ ጂፒኤስ ኣብ ODK ሶፍትዌር ክቐርብ እዩ።*  ንባብ ጂፒኤስ ምልኡ | | | | | | | | | |  |
| **ውፅኢት ቃለ መሕትት** | | | | | | | | | | | | | | | | |
| N | | **ውፅኢት ቃለ መሕትት ደቂ ኣንስትዮ ምልኡ፡፡** | | | | ሙሉእ ብሙሉእ ተጠናቒቑ 1  ኣብ ገዛ ኣይነበራን 2  ንኻልእ እዋን ተመሓላሊፉ 3  ፍቃደኛ ኣይኮናን 4  ብዝተወሰነ መልክዑ ተጠናቒቑ 5  መልሲ ምሃብ ዘይኽእላ/ኣካለ ጎዶሎ 6 | | | | | | | | | |  |

ንዝገበርክናለይ ምትሕብባር ኣዝየ የመስግን። የቐንየለይ
