# Supplementary material for: Does proximity of women to facilities with better choice of contraceptives affect their contraceptive utilization in rural Ethiopia?
Source: PLoS One. 2017 Nov 13;12(11):e0187311. doi: 10.1371/journal.pone.0187311 (PMC5683563; doi:10.1371/journal.pone.0187311)
Supplement: S1 File — (ZIP) [file pone.0187311.s004.zip › Questionnaires/Tigrigna Version/PMA2020_householdroster_Tigrigna_ R1.doc]

**mADDS –መመዝገቢ ዝርዝር ስድራቤት (መጠይቕ ቤተሰብ)**

| **ቑፅሪ** | **ሕቶን መፃረዪን** | **ኮድን መማረፅታትን** | | | | | | **ሕለፍ** | |
| --- | --- | --- | --- | --- | --- | --- | --- | --- | --- |
| **መለለዪ ቃለ መሕትት ምገባር ቅድሚ ምጅማርካ/ኪ እዞም ዝስዕቡ መለለዪ መረዳእታ ምላእ/ኢ** | | | | | | | | | |
| A | ናብዚ ስድራቤት ክንደይ ጊዘ ዑደት ተገይሩ? | ንመጀመርታ ግዘ 1  ንኻልኣይ ግዘ 2  ንሳልሳይ ግዘ 3 | | | | | | |  |
| B | ሽም መረዳእታ ሰብሳቢ/ት፡ እዚ ሽምኪ/ካ ድዩ?  እንተድኣዘይኮይኑ ሽምኪ/ካ ፀሓፊ/ፍ  *ODK ምስ ስልኪ ቑፅሪ ዝዛመድ ሽም ከውፀአልካ/ኪ እዩ* | እወ 1  የለን 0 | | | | | | |  |
|  |  |  | | | | | | |  |
| C | **ሐዚ ኣብ እስክሪን ዘሎ ሰዓትን ዕለትን**  እቲ ኣብ እስክሪን ዘሎ ዕለትን ሰዓትን ትኽክል ድዩ? | እወ 1  የለን 0 | | | | | | | የለንእንተኾይኑ ናብ E ሕለፍ/ፊ |
| D | ትኽክለኛ ዕለትን ሰዓትን ምልኡ | ዕለት | ወርሒ | | መዓልቲ | | ዓመት | |  |
|  |  | ሰዓት | ሰዓት | ደቒቓ | | AM/PM | | |  |
| E | ክልል  **እቲትካል/ስድራቤት ዝርከበሉ ክልል ምልኡ** | ትግራይ 1  ዓፋር 2  አምሓራ 3  ኦሮሚያ 4  ሶማሊ 5  ቤንሻንጉል ጉሙዝ 6  ደ/ብ/ብ/ሕ/ክ/ 7  ጋምቤላ 8  ሐረሪ 9  አዲስ አበባ 10  ድሬ ዳዋ……………………………………….11 | | | | | | |  |
| F | ዞን/ወረዳ  **ስድራቤት ዝርከበሉ ዞን/ወረዳ ምልኡ** | *ኦዲከይ ኣብ SQE ብዝተመረፀ ክልል መሰረት ዝርዝር ተቐራራቢ ዞን/ወረዳ ከቕርበልኩም እዩ* | | | | | | |  |
| G | ሽም ከባቢ/ጣብያ  **እቲ ስድራቤት ኣብ ዝርከበሉ ቆፀራ ቦታ መለለዪ ቑፅሪ መዝግብ** | *ኦዲከይ ኣብ SQF ብዝተመረፀ ዞን/ወረዳ መሰረት ዝርዝር ተቐራራቢ ሽም ከባቢ/ጣብያ ከቕርበልኩም እዩ* | | | | | | |  |
| H | ቁፅሪ መለለዪ ቖፀራ ቦታ  **እቲ ስድራቤት ኣብ ዝርከበሉ ቆፀራ ቦታ መለለዪ ቑፅሪ ምልኡ** |  | | | | | | |  |
| I | መለለዪ ተራ ቑፅሪ ገዛ  **እዚ ስድራቤት ካብ ቅጥዒ መመዝገቢ ዝርዝር ስድራቤት ዝተወሰደ ምኻኑ ንምምልኻት መለለዪ ተራ ቑፅሪ ገዛ ካብ ቅጥዒ መመዝገቢ ዝርዝር ስደራቤት ብምውሳድ ምልኡ** |  | | | | | | |  |
| J | መለለዪ ቑፅሪ ስድራቤት  **መለለዪ ቑፅሪ ስድራቤት ካብ ቅጥዒ መመዝገቢ ዝርዝር ስድራቤት ብምውርራስ ምልኡ** |  | | | | | | |  |
| K | መልሲ ክህቡ ዝኽእሉ ኣባል ስድራቤት ሎመዓንቲ ቃለ መሕትት ንኽግበረሎም ኣብ ገዛ ተረኺቦም ዶ? | እወ 1  የለን 0 | | | | | | | የለን እንተኾይኑ ናብ P ሕለፍ/ፊ |
| **ስምምዕ ፍቓድ**  **መልሲ ክህብ ዝኽእል ኣባል ስድራቤት ብምርካብ ቀፂሉ ዝቐረበ ስምምዕ ፍቓድ ኣምብበሎም፡፡** | | | | | | | | | |
| ጥዕና ይሃበለይ፡፡ሽመይ ________________________________ይበሃል፡፡ ኣብ ኣዲሰ ኣበባ ዩኒቨርስትን ሚንስትሪ ሓለዋ ጥዕናን እየ ዝሰርሕ ፡፡ኣብዚ ሐዚ እዋን ብዛዕባ ጥዕናን ምስ ጥዕና ዝዛመዱ ጉዳያትን ብዝምልከት መፅናዕቲ እናካየድና ንርከብ፡፡ኣብዚ መፅናዕቲ ተሳታፊት ብምኻንክን ካብ ልቢ ኢና ነመስግን ፡፡ዝህባና መረዳእታ ድማ መንግስቲ ብዛዕባ ጥዕና ንምምሕያሽ ንዝነድፎም ትልምታት ዓብዪ ረብሓ እዩ ዘለዎ ፡፡ ዝህባና ሓበሬታ ብምሽጥር ዝተሓዝ ኣብ ርእሲ ምኻኑ ካብ ኣባላት ጉጅለ መፅናዕቲ ወፃኢ ኻልእ ሰብ ክሪኦ ኣይኽእልን፡፡ኣብዚ መፅናዕቲ ምስታፍ ኣብ ሰናይ ድልየትክን ዝተመስረተ እንትኾን ክትምልስኦ ዘይትደልየኦ ሕቶ እንተልዒለ ድማ ይንገራኒ እሞ ናብ ዝቕፅል ሕቶ ክሓልፍ እየ ወይድማ ነዚ ቓለ መሕትት ኣብ ዝኾነ እዋን ከቋርፅኦ ይኽእላ እየን ኾይኑ ግና ዝህባና ሓበሬታ ንፅንዓትና ዓብዪ ረብሓ ስለዘለዎ ኣብዚብዚ መፅናዕቲ ምሉእ ብሙሉእ ከምዝሳተፋ ተስፋ ንገብር፡፡ ብዛዕባ ስድራቤትክንን ካልኦት ኣባላት ስድራቤትክንን ብዝምልከት ዝተወሰኑ ሕቶታት ክሓተክን እየ፡፡ ቀፂለ ድማ ካብ መንጎ ኣባላት ስድራቤትክን ዕድመአን ካብ 15 ክሳብ 49 ዓመት ዘለዋ ደቂ ኣንስትዮ ነንሕድሕደን መጠይቕ ደቅ ኣንስትዮ ክሓተን እየ፡፡  ብዛዕባ እዚ መፅናዕቲ ብዝምልከት ዝኾነ ዓይነት ሕቶ ዶ ይህልወክን? | | | | | | | | | |
| L | ስምምዕ ፍቓድ ዝሓዘ ኮፒ ወረቐት ንመልሲ ወሃቢት ብምቕራብ ኣብራህርሀለን ብምቕፃል ድማ ሐዚ እቲ ቃለመሕትት ክጅምር ዶ ይኽእል? ብምባል ሕተት/ቲ | እወ 1  የለን 0 | | | | | | የለን እንተኾይኑ ናብ P ሕለፍ/ፊ | |
| M | ፊርማ መልሲ ወሃቢት  **ወልሲ ወሃቢት ኣብዚ ፅንዓት ንምስታፍ ከም ዝተስማዕማዓ ምኻነን ንምምልካት ንኽፍርማ ወይድማ ኣብ ዝተዳለወ ሰፁን ምልክት ንኽገብራ ሕተት/ቲ፡፡** | ፊርማ መውሃቢ  ምልክት መግበሪ ሳፁን: ☐ | | | | | |  | |
| N | ሽም መረዳእታ ኣካቢ/ት  **ኣብዚ መፅናዕቲ ንምስታፍ ከምዝተስማዕመዓ ከም ምስክር ብምኻን ሽምካ/ኪ ፅሓፍ/ፊ፡፡** |  | | | | | |  | |
| O | ሽም መልሲ ወሃቢት  **ናይ መጀመርታ ሽም መልሲ ወሃቢት ፅሓፍ/ፊ** |  | | | | | |  | |

| **ክፍሊ 1 መመዝገቢ ዝርዝር ስድራቤት**  **ሐዚ ድማ ብዛዕባ ኩሎም ኣባላት ስድራቤት ዝምልከቱ ዝተወሰኑ ሕቶታት ካብ ባዕለን ብምጅማር ብዛዕባ ሕድሕድ ኣባላት ስድራቤት ማለት መብዛሕቲኡ እዋን ኣብዚ ዝነብሩን ትማሊ ለይቲ ጋሻ ኾይኖም ኣብዚ ዝሓደሩን ብምልኦም ዝምለከቱ ዝተወሰኑ ሕቶታት ክሓተክን እየ ዝደሊ፡፡** | | | | | | | | | |
| --- | --- | --- | --- | --- | --- | --- | --- | --- | --- |
| ቁፅሪ | 1 | 2 | 3 | 4 | | 5 | 6 | 7 | 8 |
|  | ናይ መጀመርታ ሽም | ፆታ | ዕድመ (ብሙሉእ ዓመት) | ኩነታት ሓዳር | | ምስ መራሒ ስድራ ዘለዎም ዝምድና | መለለዪ ቑፅሪ ስድራቤት | (ሽም) መብዛሕቲኡ እዋን ምስዚ ስድራቤት ድዩ ዝነብር ወይድማ ትማሊ ለይቲ ጋሻ ኾይኑ ኣብዚ ገዛ ዶ ሓዲሩ? | ነዚ መፅናዕቲ ብቑዓት ዝኾና ደቂ ኣንስትዮ መልሲ ወሃብቲ |
|  |  | ወዲ 1  ጓል 2 |  | በዓል ሓዳር 1 ከም ሰብኣይንሰበይትን ብሓባር ዝነብሩ 2 ዝተፋትሑ/ዝተፈላለዩ 3 ስብኣየን ዝሞትወን/ሰበይቶም ዝሞተኦም 4 ሐዳር ዘይፈተኑ 5 | | መራሒ ስድራ 1  ስብኣይ/ሰበይቲ 2  ጓል/ወዲ መራሒ ስድራ 3  ስብኣይ/ሰበይቲ ጓል/ወዲ መራሒ ስድራ (ዘማ) 4  ወዲ/ጓል ውልድ ወራሒ ስድራ 5  ወለዲ 6  ኣቦ/እነ ሰብኣይ/ሰበይቲ (ሓሙ/ሓማት) 7  ሓው/ሓፍቲ 8  ካልኦት 9  ኣይፈለጥኩን -88 |  | መብዛሕትኡ እዋን ምስዚ ስድራዝነብሩ ኣባል ስድራቤት 1  መብዛሕቲኡ እዋን ምስዚ ስድራቤት ዝነብሩኾይኖም ትማሊ ለይቲ ኣብ ገዛ ዘይሓደሩ 2  ጋሻ ኾይኖም ትማሊ ለይቲ ኣብ ገዛ ዝሓደሩ 3 | እወ 1  የለን….0  *ኦዲከይ ነዚ መፅናዕቲ ብቑዓት ዝኾና ደቂ ኣንስትዮ ብምፍላይ ከቕርበልኩም እዩ* |
| 1 |  |  |  |  | |  |  |  |  |
| 2 |  |  |  |  | |  |  |  |  |
| 3 |  |  |  |  | |  |  |  |  |
| 4 |  |  |  |  | |  |  |  |  |
| 5 |  |  |  |  | |  |  |  |  |
| 6 |  |  |  |  | |  |  |  |  |
| 7 |  |  |  |  | |  |  |  |  |
| 8 |  |  |  |  | |  |  |  |  |
| 9 |  |  |  |  | |  |  |  |  |
| 10 |  |  |  |  | |  |  |  |  |
| *ነዞም መረዳእታ ንሓደ ስድራቤት መዝጊብኩም ምስ ወዳእኹም ቐፂሉ ዝቐረበ መፃረዪ ዝተረሰዐ ንኸይህሊ ንምፅራይ ብምሕታት ዝተረሰዐ እንተሃልዩ ናብ ላዕሊ ተመሊስኩም ኣድላዪ መረዳእታ ምልኡ፡፡* | | | | | | | | | |
| 9 | ካልኦትመብዛሕትኡ እዋን ምስዚ ስደራቤት ዝነብሩ ኣባላት ስድራ ወይድማ ትማሊ ለይቲ ጋሻ ኾይኖም ዝሓደሩ ዘይተመዝገቡ ኣለው ዶ? | | | | እወ 1  የለን 0 | | | | የለን እንተኾይኑ ናብ 10 ሕለፍ/ፊ |

| **ካልኣይ ክፋል-ትሕዝቶ (ናውቲ)/ንዋይ ሰድራቤት**  **ሐዚ ድማ ብዛዕባ ሰድራቤትኩም ዝጥቀምሎም ናውቲ ወይድማ ዘለዎ ንዋይ/ጥሪት ብዝምልከት ዝተወሰኑ ሕቶታት ክሓተክን እየ** | | | | | | | | | | |
| --- | --- | --- | --- | --- | --- | --- | --- | --- | --- | --- |
| 10 | ስድራቤትኩም እዞም ዝስዕቡ ናውቲ ኣለዎ ዶ?  ኤሌክትሪክ መብራህቲ?  ናይ መንደቕ ሰዓት?  ራድዮ  ፃዕዳ/ፀሊም ሕብሪ ስክሪንቴሌቪዥን?  ሕብራዊ ስክሪን (ከለር) ቴሌቪዥን?  ስልኪ ጁባ/ሞባይል ስልኪ?  ስልኪ ገዛ?  ፍሪጅ?  መዝሓሊ?  ኤሌክትሪክ ጀነሬተር?  ማሽን መሕፀቢ ኽዳን?  ኮምፒተር?  ዲጂታል ፎቶ ካሜራ?  ዲጂታል ዘይኮነ ፎቶ ካሜራ?  ዴክ?  ዲቪዲ/ቪሲዲ?  ናይ ስፌት መኪና?  ዓራት?  ጠረጴዛ?  ኣርማድዮ?  ሳይክል?  ሞተር ሳይክል?  መኪና?  ሞተር ዘለወን ጀልባ?  ሞተር ዘይብለን ጀልባ?  ምንም የብሎምን  **ንኹሎም ዝተዘርዘሩ ብምንባብ ዝምልከቶም ኮድ ምልኡ፡፡** |  | እወ  1  1  1  1  1  1  1  1  1  1  1  1  1  1  1  1  1  1  1  1  1  1  1  1  1  -88 | | ኣይ  0  0  0  0  0  0  0  0  0  0  0  0  0  0  0  0  0  0  0  0  0  0  0  0  0 | | | |  | |
| 11 | ጥሪትኩም ኣብ ገዛ (መኖርያ) ውሸጢ ድዮም ዝነብሩ? | እወ 1  የለን 0 | | | | | | | ኣይብልናን እንተኾይኑ ናብ 13 ሕለፍ/ፊ | |
| 12 | ንሕድሕድ ጥሪት፡  ኣብ ውሽጢ ገዛ ዝነብሩ ክንደይ ጥሪት ኣለውኹም?  በጊዕ/ጤል  ደርሆ/ማይ ደርሆ/ቆቋሕ  ከፍቲ/ፈረስ/ኣድጊ  **ዜሮ መልሲ ክኸውን ይኽእል እዩ፡፡ ኣይፈለጥኩን እንተኾይኑ -88 ፤ክምልሱ እንተዘይደልዮም (ንዘይመለስዎም) -99 ምልኡ፡፡** |  | | | | | | |  | |
| **ሳልሳይ ክፋል - ምልከታ መንበሪ ገዛ**  **ናይ መንበሪ ገዝኦም መሬት፣ ክዳን ገዛን መንደቕ ደገን ተመልከት/ቲ፡፡** | | | | | | | | | | |
| 13 | ቐንዲ መሬት ገዛ ዝተሰርሐሉ ኣቕሓ ካብ ምንታይ እዩ  **ምልከታ** | **ናይተፈጥሮ መሬት**  ጭቃ/ሓመድ 1  ብዒባ ዝተለቕለቐ 2  **ብመጠኑ ዝተሰርሐ መሬት**  ዝተደርደረ ዕንፀይቲ 3  ኣርቃይ/ሸምባቆ 4  **ሙሉእብሙሉእዝተሰርሐመሬት**  ፖርኬ/ዝተፀርበ ጣውላ 5  ኣስፋልት/ቫኔል 6  ቴራዞ/ሴራሚክስ 7  ስሚንቶ 8  ናይ ሱፍ/ሃሪ/ጡጥ ምንፃፍ መሬት 9  ናይ ፕላስቲክ ምንፃፍመሬት 10  ካልእ 11 | | | | | | |  | |
| 14 | ቐንዲ ክዳን ገዛ ዝተሰርሐሉ ኣቕሓ  **ምልከታ** | **ክዳን ተፈጥሮ**  ክዳን ዘይብሉ 1  ሳዕሪ/ዱርቃ 2  **ብመጠኑ ዝተሰርሐ ክዳን**  Rustic Mat 3  ሻምባቆ/ኣርቃይ 4  ዘይተፀረበ ጣውላ 5  ካርቶን 6  **ሙሉእ ብሙሉእ ዝተዛዘመ ክዳን**  ቆርቆሮ 7  ዕንፀይቲ 8  ካላማይን/ሰመንት ፋይበር 9  ብሴራሚክ/ሸኽላ ጡብ ዝተኸደነ 10  ስሚንቶ 11  ዝተፀረበ/ዝተቖራረፀ ጣውላ 12  ኣስቤስቶስ/ሙቐት ዝቓቖሞ ፕላስቲክ 13  ካልእ 14 | | | | | | |  | |
| 15 | ቐንዲ መንደቕ ደገ ገዛ ዝተስርሐሉ ኣቕሓ  **ምልከታ** | **ናይ ተፈጥሮ መንደቕ**  መንደቕ ዘይብሉ 1  ጉንዲ/ሸምባቆ/ኣርቃይ 2  **ብመጠኑ ዝተነድቐ**  ዕንጨይቲ ምስ ጭቃ 3  እምኒ ምስ ጭቃ 4  ካብ ክፍቲ ጡብ ዝተሰርሐ 5  ንብርብር ጣውላ 6  ካርቶን 7  ካብ ዘገልገለ ዕንጨይቲ ዝተስርሐ 8  **ምሉእ ብሙሉእ ዝተነደቐ**  ስሚንቶ 9  እምኒ ምስ ሰሚንቶ 10  ጡብ 11  ብሎኬት 12  ካብ ሕቱም ጡብ ዝተሰርሐ 13  ዝተፀረበ/ዝተቖራረፀ ጣውላ 14  ካልእ 15 | | | | | | |  | |
| **ራብዓይ ክፋል ዝስተማይ፣ፅርየት ከባብን መሕፀብ ኢድን ዝርከበሉ ቦታን**  ሐዚ ድማ ብዛዕባ ዝስተማይ፣ፅርየት ከባብን ሓለዋጥዕናን ዝምልከቱ ዝተወሰኑ ሕቶታት ክሓተኩም እየ | | | | | | | | | | |
| 16 | ንመሕፀቢ አእዳውኩም ዝኸውን ዝተፈልየ ስፍራ ኣለኩም ዶ? | እወ 1  የለን 0  ኣይፈለጥኩን -88 | | | | | | | | የለን ወይድማ ኣይፈለጥኩን ተኾይኑ ናብ 19 ሕለፍ/ፊ |
| 17 | ክተርእዩኒ/ያኒ ዶ ትኽእሉ/ትኽእላ? | እወ 1  የለን 0 | | | | | | | | የለን ተኾይኑ ናብ 19 ሕለፍ/ፊ |
| 18 | **ኣባላት ስድራ አእዳዎም ኣብ ዝሕፀብሉ ስፍራ ቐፂሎም ዝተዘርዘሩ ምህላዎም ተመልከት/ቲ**  ሳሙና ኣሎ  ዝተቐመጠ ማይ ኣሎ  ማይ ቧምቧ ኣሎ  መሕፀቢ ኢድ ስፍራ ጥቓ ሽንትቤት እዩ ዝርከብ  ካብቶም ዝተዘርዘሩ ምንም ነገር የለን |  | | እወ  1  1  1  1  1 | | | | የለን  0  0  0  0  0 | |  |
| 19 | ካብዞም ቐፂሎም ዝተዘርዘሩ ዓይነት ፍልፍል ማይ መብዛሕትኡ እዋን ኣባላት ስድራቤትኩም ኣብ ዓመት ኣብ ዝኾነ ይኹን እዋን ንዝኾነ ይኹን ግልጋሎት ዝጥቀመሎም ኣየኖት እዮም?  **ማይ ቧምቧ**  ቧምቧ ኣብ ውሽጢ ገዛ  ቧምቧ ኣብ ውሽጢ መረባ  ናይ ህዝቢ ቧምቧ/ቦኖ  ጥልቀት ዘለዎ ማይ ጉድጓድ  **ማይ ጉድጓድ**  ዝተኸለለ ማይ ጉድጓድ  ዘይተኸለለ ማይ ጉድጓድ  **ፍልፍል ዓይኒ ማይ**  ዝተኸለለ/ዝተሓለ ፍልፍል ዓይኒ ማይ  ዘይተኸለለ ፍልፍል ማይ ዓይኒ  ናይ ዝናብ ማይ  ብቦቲ መኪና ዝከፋፈል ናይ ታንከር ማይ  ብጋሪ ዝጉተት ማይ ታንከር  ናይ ገፀ ምድሪ ማይ (ሩባ/ዒላ/ቓላይ/ግድብ/ጠለፋ/ማይ መስኖ)  ዝተዓሸገ ማይ  ኣብ ላስቲክ/ጎማ ዝመልአ ማይ  **ኩሉም ዝተዘርዘሩ ብምንባብ ንዝምልከቶም ኮድ ምልኡ፡፡** |  | | እወ  1  1  1  1  1  1  1  1  1  1  1  1  1  1 | | | | የለን  0  0  0  0  0  0  0  0  0  0  0  0  0  0 | |  |
| 20 | መብዛሕትኡ እዋን ኣባላት ስድራቤትኩም ዝጥቀምሉ ቐንዲ ፍልፍል ዝስተ ማይ እንታይ እዩ?  **ማይ ቧምቧ**  ቧምቧ ኣብ ውሽጢ ገዛ  ቧምቧ ኣብ ውሽጢ መረባ  ናይ ህዝቢ ቧምቧ/ቦኖ  ጥልቀት ዘለዎ ማይ ጉድጓድ  **ማይ ጉድጓድ**  ዝተኸለለ ማይ ጉድጓድ  ዘይተኸለለ ማይ ጉድጓድ  **ፍልፍል ዓይኒ ማይ**  ዝተኸለለ ፍልፍል ዓይኒ ማይ  ዘይተኸለለ ፍልፍል ማይ ዓይኒ  ናይ ዝናብ ማይ  ብቦቲ መኪና ዝከፋፈል ናይ ታንከር ማይ  ብጋሪ ዝጉተት ማይ ታንከር  ናይ ገፀ ምድሪ ማይ (ሩባ/ዒላ/ቓላይ/ግድብ/ጠለፋ/ማይ መስኖ)  ዝተዓሸገ ማይ  ኣብ ላስቲክ/ጎማ ዝተመልአ ማይ  **ኩሎም ዝተዘርዘሩ ብምንባብ ቐንዲ ፍልፍል ዝሰተ ማይ ምልኡ፡፡ቐንዲ ፍልፍል ዝስተ ማይ ግዴታ ኣብ ሕቶ HQ 19 እወ ዝብል ዝተኸበበሉ ክኸውን ኣለዎ፡፡** | 1  2  3  4  5  6  7  8  9  10  11  12  13  14  15 | | | | | | | |  |
| 21 | መብዛሕትኡ እዋን ንስድራቤትኩም ዝኸውን ምግቢ ንምብሳል፣ኢድ ንምሕፃብን ክዳን ንምሕፃብን ዝኣመሳሰሉ ጉዳያት ንምክንዋን እትጥቀምሉ ማይ ካበይ ትረኽቡ?  **ማይ ቧምቧ**  ቧምቧ ኣብ ውሽጢ ገዛ  ቧምቧ ኣብ ውሽጢ መረባ  ናይ ህዝቢ ቧምቧ/ቦኖ  ጥልቀት ዘለዎ ማይ ጉድጓድ  **ማይ ጉድጓድ**  ዝተኸለለ ማይ ጉድጓድ  ዘይተኸለለ ማይ ጉድጓድ  **ፍልፍል ዓይኒ ማይ**  ዝተኸለለ ፍልፍል ዓይኒ ማይ  ዘይተኸለለ ፍልፍል ማይ ዓይኒ  ናይ ዝናብ ማይ  ብቦቲ መኪና ዝከፋፈል ናይ ታንከር ማይ  ብጋሪ ዝጉተት ማይ ታንከር  ናይ ገፀ ምድሪ ማይ (ሩባ/ዒላ/ቓላይ/ግድብ/ጠለፋ/ማይ መስኖ)  ዝተዓሸገ ማይ  ኣብ ላስቲክ/ጎማ ዝመልአ ማይ  **ኩሎም ዝተዘርዘሩ ብምንባብ ቐንዲ ፍልፍል ዝሰተ ማይ ምልኡ፡፡ቐንዲ ፍልፍል ዝስተ ማይ ዝምላእ ግዴታ ኣብ ሕቶ HQ 19 እወ ዝብል ኮድ ዝተኸበበሉ ክኸውን ኣለዎ፡፡** | 1  2  3  4  5  6  7  8  9  10  11  12  13  14  15 | | | | | | | |  |
| 22 | **ኣብ ሕቶ 19 ንዝተመረፁ ዓይነት ፍልፍል ማይ ካብ ሕቶ 22 ጀሚሩ ክሳብ ሕቶ 24 ን X ሻዕ ክደጋገሙ እዮም፡፡ እዞም ፍልፍል ማይ ዘጠቓልልዎም፡**  *ኣብ ሕቶ19 ንዝተመርፁ ዓይነት ፍልፍል ማይ ኦዲከይ ሶፍትዌር ከቕርበልኩም እዩ*  ኣብ ዝኾነ ይኹን እዋን ማይ ካብ [ዓይነት ፍልፍል ማይ] ከም ዝጥቀማ ነጊረናኒ ነይረን ስድራቤተን ካብቲ ፍልፍል ማይ ኣብ ዝኾነ ይኹን እዋን፡  ንምስታይ ይጥቀም ዶ  ንፀብሒ መስርሒ ይጥቀም ዶ  ኸፍቲ(እንስሳት) ንምስታይ ይጥቀም ዶ  ንኣትክልቲ/ሕርሻ ይጥቀም ዶ  ንንግዲ/ትካል ይጥቀም ዶ  *ኣብ ሕቶ HQ19 ዝተመርፁ ዓይነት ፍልፍል ማይ መሰረት ብምግባር ኦዲከይ ሶፍትዌር ተመሳሳሊ ሕቶ ከቕርበልኩም እዩ፡፡* |  | | እወ  1  1  1  1  1 | | የለን  0  0  0  0  0 | | | |  |
| 23 | እቲ [ፍልፍል ማይ] ብዘይምቁራፅ ዝርከበሉ እዋን፡  ዓመት ሙሉእ  ኣብ ዓመት ዝተወሰነ ግዘ  ኣብ ዓመት ውሑድ ግዘ 3  ***ኣብ ሕቶ HQ19 ንዝተመርፁ ዓይነት ፍልፍል ማይ መሰረት ብምግባር ኦዲከይ ሶፍትዌር ተመሳሳሊ ሕቶ ከቕርበልኩም እዩ፡፡*** | 1  2  3 | | | | | | | |  |
| 24 | ካብቲ [ፍልፍል ማይ] ኣቕርቦት ማይ ይህልው ኢዩ ኢልክልን ኣብ ዝኸድክናሉ እዋን ዝበዝሕ ግዘ ማይ ትረኽባ ዶ?  እወ፣ኩሉግዘ  የለን፣ሓልሓሊፉ መዓዝ ከም ዘቋርፅ ዝፍለጥ  የለን፣ሓልሓሊፉ መዓዝ ከም ዘቋርፅ ዘይፍለጥ  ***ኣብ ሕቶ HQ19 ዝተመርፁ ንካልኦት ዓይነት ፍልፍል ማይ መሰረት ብምግባር ኦዲከይ ሶፍትዌር ተመሳሳሊ ሕቶ ከቕርበልኩም እዩ፡፡*** | 1  2  3 | | | | | | | |  |
| 25 | ካብቲ [ፍልፍል ማይ] ሓደ ግዘ ማይ ቐዲሕኽን ንምምላስ ክንደይ ደቒቓ ይወስደልክን?  **ዜሮ መልሲ ክኸውን ይኽእል እዩ፡፡ዝወሰደለን ግዘ ተራ ንምፅባይ ዘጥፈአኦ ጊዘለ ኣብ ግምት ብምእታው ክምዝገብ ኣለዎ፡፡ ኣይፈለጥክዎን ዝብላ እንተኾይነን -88 ምልኡ፡፡**  ***ኣብ ሕቶ HQ19 ዝተመርፁ ዓይነት ፍልፍል ማይ መሰረት ብምግባር ኦዲከይ ሶፍትዌር ተመሳሳሊ ሕቶ ከቕርበልኩም እዩ፡፡*** | ደቒቓ | |  | | | | | |  |
| 26 | ስድራቤትኩም ናይ ኣትክልቲ ስፍራ ኣለኩም ዶ? | እወ 1  የለን 0 | | | | | | | |  |
| 27 | ካብ ኣባላት ስድራቤትኩም ካብዞም ቐፂሎም ዝተዘርዘሩ ዓይነት ሽንትቤት ዝጥቀሙ ኣለዉዶ?  **ዘመናዊ ኾይኑ ብማይ ዝወርድ ምስ-------ዝተተሓሓዘ**፡  መወገዲ ርስሓት ትቦ  ሰገራ ወይድማ ርስሓት ጉድጓድ  ናብ ካልእ ቦታ ዝወርድ  ናበይከም ዝወርድዘይፍለጥ/እርግፀኛ ኣይኮንኩን/ ኣይፈለጥኩን  መተንፈሲ ኣየር ዘለዎ ጉድጓድ ሽንትቤት (VIP)  ብሊሾዝተሰርሐ ጉድጓድ ሽንትቤት  ብሊሾ ዘይተሰርሐ ጉድጓድ ሽንትቤት  ንዱኽዒ ዝተዳለወ ሽንትቤት  ባልዲ/ባዞ  ናብ ሩባ ዝወርድ  ካልእ  **ኩሎም ዝተዘርዘሩ ብምንባብ ንዝምልከቶም ኮድ ምልኡ** |  | | እወ  1  1  1  1  1  1  1  1  1  1  1 | | | | የለን  0  0  0  0  0  0  0  0  0  0  0 | |  |
| 28 | መብዛሕትኡ እዋን ኣባላት ስድራቤትኩም እንታይ ዓይነት ሽንትቤት እዮም ዝጥቀሙ?  **ዘመናዊ ኾይኑ ብማይ ዝወርድ**፡  መወገዲ ርስሓት ትቦ  ሰገራ ወይድማ ርስሓት ጉድጓድ  ናብ ካልእ ቦታ ዝወርድ  ናበይከም ዝወርድዘይፍለጥ/እርግፀኛ ኣይኮንኩን/ ኣይፈለጥኩን  መተንፈሲ ኣየር ዘለዎ ጉድጓድ ሽንትቤት (VIP)  ብሊሾዝተሰርሐ ጉድጓድ ሽንትቤት  ብሊሾ ዘይተሰርሐ ጉድጓድ ሽንትቤት  ንዱኽዒ ዝተዳለወ ሽንትቤት  ባልዲ/ባዞ  ናብ ሩባ ዝወርድ  ካልእ  **ኩሎም ዓይነት ሽንትቤት ብምንባብ ብቐንዲ ዝጥቀምሉ ዓይነት ሽንት ቤት ጥራሕ ምልኡ፡፡እቲ ዝምላእ ኮድ ኣብ ሕቶ HQ 25 ግዴታ ዝተመረፀ ክኸውን ኣለዎ** | 1  2  3  4  5  6  7  8  9  10 | | | | | | | |  |
| 29 | **ኣብ ሕቶ HQ 27ንዝተመረፁ ዓይነት ሽንትቤት ሕቶ HQ 29 ነንሕድሕዶም ንምሕታት ንX ግዘ ክደጋገም እዩ፡**  *ኣብ ሕቶ HQ 27 ዝተመሩፁ ዓይነት ሽንትቤትብ ODK ሶፍትዌር ከቕርበልኩም እዩ*  ኣባላት ስድራቤትኩም ኣብ[ዓይነት ሽንትቤት] ንኽንደይ እዋን ይጥቀሙ?  **ስድራቤቶም ኹሉ ግዘ ዝጥቀምሉ ዓይነት ሽንት ቤት ጥራሕ**  *ኣብ ሕቶ HQ27ንዝተመረፁ ዓይነት ሽንትቤት ብODK ሶፍትዌር ሓደ ዓይነት ሕቶ ክቐርበልኩም እዩ* | ኩሉግዘ 1  ዝበዝሕ ግዘ 2  ሓደ ሓደ ግዘ 3  ሓልሓሊፉ 4 | | | | | | | |  |
| 30 | ካብ መንጎ ኣባላት ስድራቤትኩም ኣብ ገዛ ይኹን ኣብ ስራሕ መብዛሕቲኡ እዋን ፍታኖም ኣብ ደገ/ጫካ ዝፍትኑ ክንደይ ሰባት እዮም?  **ካብ መንጎ ኣባላት ስደራቤት X ሰባት ፍታኖም ኣብ ደገ/ጫካ ይፍትኑ፡፡ኣይፈለጥኩን እንተኾይኑ**  **-88 ምልኡ** | በዝሒ ሰባት | |  | | | | | |  |
| 31 | ስድራቤትኩም ትሕቲ ሓሙሽተ ዓመት ዝዕድመኦም ኩሎም ህፃውንቲ ፍታኖም ንምውጋድ ትጥቀምዎ ሜላ እንታይ እዩ?  ኩሎም ህፃናት ሽንት ቤት ይጥቀሙ እዮም  እቲ ፍታን ኣብ ዘሎ ንገድፎ  ኣብ ውሽጢ መረባ ንቐብሮ  እቲ ፍታን ናብ ሽንትቤት ንደፍኦ  እቲ ፍታን ናብ መዋህለሊ ጎሓፍ ንደፍኦ  እቲ ፍታን ናብ ረሳሕማይ ዝወርደሉ ቱቦ ንደፍኦ  ንዱኽዒ ንጥቀመሉ  ነቲ ፍታን ነቃፅሎ  ኣይፈለጥኩን  *ዕድመኦም ትሕቲ ሓሙሽተ ዓመት ዝኾኑ ህፃናት ODK ሶፍትዌር ካብ መመዝገቢ ዝርዝር ስድራቤት (HQ3) ከቕርበልኩም እዩ* |  | | እወ  1  1  1  1  1  1  1  1  -88 | | | የለን  0  0  0  0  0  0  0  0 | | |  |
| መልሲ ወሃቢት ንዝሃባኻ/ኺ ጊዘ ኣመስጊንካ/ኪ ተሰናበት/ቲ፡፡  **እዚ ቃለ መሕትት ምስ መልሲ ወሃቢት ወላእኳ ተተወድአ ነገር ግን ካብ ናይ መልሲ ወሃቢት ገዛ ወፃኢ ብኣኻ/ኺ ዝምልኡ ዝተወሰኑ ሕቶታት ኣለውኻ/ኺ፡፡** | | | | | | | | | | |
| **ቦታን ውፅኢት ናይቲ ቃለመሕትትን** | | | | | | | | | | |
| P | **ጥቓ ኣፍደገ መልሲ ወሃቢት ብምኻን ንባብ ጂፒኤስ ውሰድ/ዲ፡፡**  **ኣኩራሲይ ትሕቲ 6 M ምስኮነ ንባብ ጂፒኤስ ምልኡ**  **ንባብ ጂፒኤስ ንወስድ ናብ ደገ ምስ ወፃእና ጥራሕ እዩ፡፡** | *መምርሒ ጂፒኤስ ካብ ኦዲኬ ሶፍትዌር ቐሪቡልኩም ኣሎ*  ንባብ ጂፒኤስ መዝግብ/ቢ | | | | | | |  | |
| Qa | ኣፍደገ ገዛ ፎቶ ንምልዓል ፍቓድ ሕተቱ፡፡  ፎቶ ንምልዓል ፍቓድ ረኺብኩም ዶ? | እወ 1  የለን 0 | | | | | | | የለን እንተኾይኑ ናብ R ሕለፍ/ፊ | |
| Qb | ኣብቲ ዝተልዓለ ፎቶ ማንም ሰብ ዘይምእታው ኣረጋግፁ | *መምርሕታት ብODK ሶፍትዌር ብቀጥታ ቐሪቡልኩም ኣሎ*  ፎቶ ኣልዕል/ሊ  ካብ ዝተልዓሉ ፎቶ ዝሓሸ ምረፁ | | | | | | |  | |
| R | ውፅኢት ቃለ መሕትት መመዝገቢ ዝርዝር ስድራቤት ምልኡ | ተጠናቒቑ 1  ኣብ ግዘ ዑደት መልሲ ክህብ ዝኽእል/ዝኾነ ኣባል ስድራቤት ኣይነበረን 2  ንኻልእ እዋን ተመሓላሊፉ 3  ፍቓደኛ ኣይኮኑን 4  ብዝተወሰነ መልክዑ ተጠናቒቑ 5  መንበሪ ገዛ ሰብ ኣይነብረሉን/መንበሪ ገዛ ኣይኮነን 6  ዝፈረሰ ገዛ 7  መንበሪ ገዛ ኣይተረኽበን 8 | | | | | | |  | |
