# Supplementary material for: Does proximity of women to facilities with better choice of contraceptives affect their contraceptive utilization in rural Ethiopia?
Source: PLoS One. 2017 Nov 13;12(11):e0187311. doi: 10.1371/journal.pone.0187311 (PMC5683563; doi:10.1371/journal.pone.0187311)
Supplement: S1 File — (ZIP) [file pone.0187311.s004.zip › Questionnaires/Tigrigna Version/PMA2020_SDP_Tigrigna_ R1.doc]

**mADDS –መጠይቕ ንግልጋሎት ወሃብቲ ትካላት (SDP)**

| **ቑፅሪ** | **ሕቶን መፃረይን** | | **ኮድን መማረፅታትን** | | | | | | | | | | | | | | | | | | | | | | | | | | | | | | | | | | **ሕለፍ** |
| --- | --- | --- | --- | --- | --- | --- | --- | --- | --- | --- | --- | --- | --- | --- | --- | --- | --- | --- | --- | --- | --- | --- | --- | --- | --- | --- | --- | --- | --- | --- | --- | --- | --- | --- | --- | --- | --- |
| **መለለዪ**  **ቃለመሕትት ምክያድ ቅድሚ ምጅማርካ/ኪ ቐፂሎም ዝቐረቡ መረዳእታታት ምምላእኻ/ኺ ኣረጋግፅ/ፂ** | | | | | | | | | | | | | | | | | | | | | | | | | | | | | | | | | | | | | |
| A | ነዚ ቃለ መሕትት ንምክያድ ናብዚ ትካል ክንደይ ጊዘ ዑደት ጌርክን? | | ንመጀመርታ ግዘ 1  ንኻልኣይ ግዘ 2  ንሳልሳይ ግዘ 3 | | | | | | | | | | | | | | | | | | | | | | | | | | | | | | | | | |  |
| B | ሽም መረዳእታ ሰብሳቢ፡ እዚ ሽምኪ/ካ ድዩ?  *ODK ምስ ስልኪ ቑፅሪ ዝዛመድ ሽም ከውፀአልኩም እዩ*  እንተድኣዘይኮይኑ ሽምኪ/ካ ፀሓፊ/ፍ | | እወ 1  ኣይፋሉን 0 | | | | | | | | | | | | | | | | | | | | | | | | | | | | | | | | | |  |
|  |  |  |  | | | | | | | | | | | | | | | | | | | | | | | | | | | | | | | | | |  |
| C | **ሐዚ ኣብ እስክሪን ዘሎ ሰዓትን ዕለትን ተመልከቲ**  ኣብ እስክሪን ዘሎ ዕለትን ሰዓትን ትኽክል ድዩ? | | እወ 1  ኣይፋሉን 0 | | | | | | | | | | | | | | | | | | | | | | | | | | | | | | | | | | ኣይፋሉን እንተኾይኑ ናብ E ሕለፍ/ፊ |
| D | ትኽክለኛ ዕለትን ሰዓትን መዝግብ/ቢ፡፡ | | ዕለት | | መዓልቲ | | | | | ወርሒ | | | | | | | | | | | | | | | ዓመት | | | | | | | | | | | |  |
|  |  |  | ግዘ | | ሰዓት | | | | | ደቒቓ | | | | | | | | | | | | | | | ቅ/ድ ቀትሪ | | | | | | | | | | | |  |
| E | ክልል  **እቲ ትካል ጥዕና ዝርከበሉ ክልል ምልኡ** | | ትግራይ 1  ዓፋር 2  አምሓራ 3  ኦሮሚያ 4  ሶማሊ 5  ቤንሻንጉል ጉሙዝ 6  ደ/ብ/ብ/ሕ/ክ/ 7  ጋምቤላ 8  ሐረሪ 9  አዲስ አበባ 10  ድሬዳዋ……………………………………….11 | | | | | | | | | | | | | | | | | | | | | | | | | | | | | | | | | |  |
| F | ዞን  ሽም እቲ ትካል ጥዕና ዝርከበሉ ዞን ምልኡ | | *ODK ኣብ ሕቶ E ብዝተመረፀ ክልል መሰረት ኣብ ትሕቲኡ ዝርከቡ ዝርዝር ዞን ከርእየኩም እዩ* | | | | | | | | | | | | | | | | | | | | | | | | | | | | | | | | | |  |
| G | ወረዳ  ሽም እቲ ትካል ጥዕና ዝርከበሉ ወረዳ ምልኡ | | *ODK ኣብ ሕቶ F ብዝተመረፀ ዞን መሰረት ኣብ ትሕቲኡ ዝርከቡ ዝርዝር ወረዳታተ ከርእየኩም እዩ* | | | | | | | | | | | | | | | | | | | | | | | | | | | | | | | | | |  |
| H | ጣብያ  ሽም እቲ ትካል ጥዕና ዝርከበሉ ጣብያ ምልኡ | | *ODK ኣብ ሕቶ G ብዝተመረፀ ወረዳ መሰረት ኣብ ትሕቲኡ ዝርከቡ ዝርዝር ጣብያታት ከርእየኩም እዩ* | | | | | | | | | | | | | | | | | | | | | | | | | | | | | | | | | |  |
| I | ቆፀራ ቦታ    ኮድ/ቁፅሪ እቲ ትካል ጥዕና ዝርከበሉ ናይ ቆፀራ ቦታ ምልኡ። | |  | | | | | | | | | | | | | | | | | | | | | | | | | | | | | | | | | |  |
| J | መለለዪ ቁፅሪ ትካል ጥዕና  መለለዪ ቁፅሪ ትካል ጥዕና ኣብቲ ናይ ቤተሰብ መመዝገቢ ቅጥዒ ብምርኣይ ምልኡ። | |  | | | | | | | | | | | | | | | | | | | | | | | | | | | | | | | | | |  |
| K | ዓይነት ትካል ጥዕና  **ዓይነት ናይቲ ትካል ጥዕና ካብዞም ዝተዘርዘሩ ብምምራፅ ምልኡ፡፡** | | ሆስፒታል 1  ጥዕና ጣብያ 2  ጥዕና ኬላ 3    ፋርማሲ 4  መደብር መድሓኒት 5    ካልእ 6 | | | | | | | | | | | | | | | | | | | | | | | | | | | | | | | | | |  |
| L | ዘመሓዳድሮ ኣካል ጥዕና  **ነቲ ትካል ዘመሓዳድሮ በዓል ስልጣን ከካብቶም ዝተዘርዘሩ ብምምራፅ ምልኡ።** | | መንግስታዊ 1  ዘይመንግስታዊ 2  ብሃይማኖት ተቋማት ዝመሓዳደር 3  ናይ ውልቀ 4  ካልእ 5 | | | | | | | | | | | | | | | | | | | | | | | | | | | | | | | | | |  |
| M | ነቲ ቃለ መሕትት ዝሓሸ መረዳእታ ክህብ ዝኽእል መልሲ ወሃቢ  ተረኺብኩም ‘ዶ? | | እወ 1  ኣይፋሉን 0 | | | | | | | | | | | | | | | | | | | | | | | | | | | | | | | | | | ኣይፋሉን እንተኾይኑ ናብ R ሕለፍ/ፊ |
| **ፍቓድ ስምምዕ**  **ብቑዕ መልሲ ክህብ ዝኽእል መልሲ ወሃቢ ብፍላይ ድማ ንተሓከምቲ ግልጋሎት ኣብ ምሃብ ሓላፍነት ዘለዎ ከም ዋና ኣመሓዳዳሪ/ወኪል ወይድማ ሓላፈፊ ግልጋሎት ምጣነ ሰድራ ብምርካብ ቃለመሕትት ግበር/ሪ፡፡ቐፂሉ ዝቐረበ መእተዊ ስምምዕ ፍቓድ ኣንብበሎም/ለን፡፡** | | | | | | | | | | | | | | | | | | | | | | | | | | | | | | | | | | | | | |
| ጥዕና ይሃበለይ፡፡ሹመይ ______________________________. ይበሃል፡፡ኣብ ክንዲ ኣዲስ ኣበባ ዩኒቨርስትን ሚኒስትሪ ሓለዋ ጥዕናን ብምኻን ብዛዕባ ግልጋሎት ጥዕና ዝምልከት መፅናዕቲ እናካየድና እንትንኮን ንእክቦ ዘለና መረዳእታ ድማ መንግስቲ ኣብ መዳይ ጥዕና ዝወሃብ ዘሎ ግልጋሎት ንምፍላጥን መደብ ጥዕና ኣብ ምምሕያሽ ንዝነድፎም ትልምታት ንምትላምን ዓብዪ ሓገዝ እዩ ዝገብረሉ፡፡ሐዚ ብዛዕባ እዚ መፅናዕቲ ብዝብልከት ቀፂለ ከብራህርሀልኩም እየ፡፡  ትካልኩም ኣብዚ መፅናዕቲ ንምስታፍ ብዕፃ ዝተመረፀ እንትኾን ብዛዕባ ምጣነ ስድራ፣ ካልኦት ምስ ስነ ተዋልዶ ጥዕና ዝዛመዱን ናይ ሕሙማት መመዝገቢ ብዝተመልከተን ዝተወሰኑ ሕቶታት ክሓተኩም እየ ፡፡ካብቲ መዝገብ ናይ ዝኾነ ሕሙም ሽም ኮነ ካልእ መረዳእታ ንካልእ ሰብ ኣይነርእየን። ካብ ትካልኩም ንእክቦ መረዳእታ መንግስቲ ይኹን ካልኦት ኣብ ጥዕና ዝሰርሑ ትካላት ትልሚ ንምትላምን ብዛዕባ ጥዕና ብዝምልከት ተወሳኺ መፅናዕቲ ንምክያድ ክጥቀምሉ ይኽእሉ እዮም፡፡ ከምኡ ድማ ካብ ትካልኩም ዝስብሰብ መረዳእታ ተመራመርቲ ዓሚቕ ትንተና ንክገብሩሉ ይጠቅም። ይኹን ድኣ እምበር ሽም ትካልኩም ዝሓዘ መረዳእታ ብተመራመርቲ ይኹን ንኻልእ ኣካል ኣይወሃብን።  ንዐኦም ንክትሓባበሩና ዝጥይቖም ትኽክለኛ መረዳእታ ንክህቡን እዩ፡፡ንሶም ክምልስዎም ንዘይኽእሉ ሕቶ ኣድላዪ መረዳእታ ናብ ክህበኒ ዝኽእል ዝምልከቶ ሰራሕተኛ ንከላልዪኒ እላቦ፡፡  ክምልስዎ ዘይደልይዎ ዓይነት ሕቶ እንተልዒለ ድማ ይንገሩኒ እሞ ናብ ዝቕፅል ሕቶ ክሓልፍ እየ ወይድማ ነዚ ቃለ መሕትት ኣብ ዝኾነ እዋን ከቋርፅዎ ይኽእሉ እዮም፡፡  ሐዚ ብዛዕባ እዚ መፅናዕቲ ብዝምልከት ዝኾነ ሕቶ ዶ ይህልወኩም? | | | | | | | | | | | | | | | | | | | | | | | | | | | | | | | | | | | | | |
| N | ስምምዕ ፍቓድ ዝሓዘ ኮፒ ወረቐት ንመልሲ ወሃቢት ብምቕራብ ኣብራህርህለን ብምቕፃል ድማ ሐዚ ነቲ ቃለመሕትት ክጅምር ዶ ይኽእል? ብምባል ሕተቲ፡፡ | | እወ 1  ኣይፋሉን 0 | | | | | | | | | | | | | | | | | | | | | | | | | | | | | | | | | | የለን እንተኾይኑ ናብ R ሕለፍ/ፊ |
| O | ፊርማ መልሲ ወሃቢ  **ወልሲ ወሃቢት ኣብዚ ፅንዓት ንምስታፍ ዝተስማዕምዑ ምኻኖም ንምምልካት ንኽፍርሙ ወይድማ ኣብ ዝተዳለወ ሰፁን ምልክት ንኽገብሩ ሕተት/ቲ፡፡** | | ፊርማ መውሃቢምልክት መግበሪ ሳፁን: ☐ | | | | | | | | | | | | | | | | | | | | | | | | | | | | | | | | | |  |
| P | ሽም መረዳእታ ኣካቢ/ት  **ከይዲ ስምምዕ ፍቓድ ብትኽክል ምክያዱ ከም ምስክር ብምኻን ሽምኩም ፅሓፉ** | |  | | | | | | | | | | | | | | | | | | | | | | | | | | | | | | | | | |  |
| Q | ናይቲ ትካል ጥዕና ሽም  **ናይቲ ትካል ጥዕና ስም ፅሓፉ** | |  | | | | | | | | | | | | | | | | | | | | | | | | | | | | | | | | | |  |
| R | ኣብዚ ትካል መደብ ስራሕኹም እንታይ እዩ?  **መልሲ ወሃቢ ዝተፈላለየሓላፍነት እንተሃልይዎ፣ ዝለዓለ ናይ ስራሕ ሓላፊነቱ ጥራሕ ፅሓፍ** | | ወናኒ ናይቲ ትካል 1  ዋና ኣመሓዳዳሪ (ሓላፊ) ናይቲ ትካል 2  ናይቲ ትካል ሰራሕተኛ 3 | | | | | | | | | | | | | | | | | | | | | | | | | | | | | | | | | |  |
| **ቑፅሪ** | **ሕቶን መፃረዪን** | | **ኮድን መማረፅታትን** | | | | | | | | | | | | | | | | | | | | | | | | | | | | | | | | | | **ሕለፍ** |
| **ክፍሊ 1 – ብዛዕባ ዝወሃቡ ግልጋሎታት ጠቕላላ መረዳእታ**  **ሐዚ ድማ ኣብ ትካልኩም ብዛዕባ ዝወሃቡ ግልጋሎታት ብዝምልከት ዝተወሰኑ ሕቶታት ክሓተኩም እየ፡፡** | | | | | | | | | | | | | | | | | | | | | | | | | | | | | | | | | | | | | |
| 1 | | እዚ ትካል ግልጋሎት ጥዕና ምሃብ ዝጀመረሉ እዋን መዓዝ ዓመተምሕረት እዩ?  **ኣይፈለጥኩን ዝብሉ እንተኾይኖም 2020 መዝግብ/ቢ** | ዓመተምሕረት | | | | |  | | | | | | | | | | | | | | | | | | | | | | | | | | | | |  |
| 2 | | እዚ ትካል ጥዕና ኣብ ሰሙን ንኽንደይ መዓልታት ግልጋሎት ይህብ?  **ትምዝግብዎ በዝሒ መዓልታት ካብ 1-7 ዘሎ ክኸውን ኣለዎ፡፡ኣይፈለጥኩን እንተኾይኑ -88 ምልኡ** | በዝሒ መዓልታት | | | | |  | | | | | | | | | | | | | | | | | | | | | | | | | | | | |  |
| 3 | | **ሐዚ ድማ ብዛዕባ ኣብዚ ትካል ዝሰርሑ ሰራሕተኛ ብዝምልከት ዝተወሰኑ ሕቶታት ክሓተኩም እየ**  ኣብዚ ሐዚ እዋን ትካልኩም በዞም ዝስዕቡ ዓይነት ሙያ ክንደይ ሰራሕተኛታት ከም ዘለዎን ከምኡ ድማ ኣብ መወዳእታ  ሎመዓንቲ ኣብ ስራሕ ዝተረኸቡ ሰራሕተኛታት ክንደይ ከም ምኻኖምን ‘ዶ ትነግሩኒ  ኣነ ዝደልዮ ዝሰልጠነሉ ወይም ዝተምሃሮ ዝለዓለ ብርኪ ትምህርቲ እምበር ሐዚ ተመዲቡ ዝሰርሐሉ ወይም ሓፂር ስልጠና ዝወሰደሉ ኣይኮነን፡፡  **ኣይፈለጥኩን እተኾይኑ-88 ኣይምልከቶምን ተኾይኑ -77 መዝግብ/ቢ ፡፡ 0 መልሲ ክኸውን ይኽእል እዩ** | ዶክተር  ነርስ/መዋለዳን ነርስ  ጥዕና መኮነን    ፋርማሲስት    ካልእት ሰራሕተኛታት ሕክምና | | | | | | | | | | | | | በዝሒ ሰራሕተኛ  __  __  __  __  __  __  __ | | | | | | | | | | | | | ሎመዓንቲ ዝተረኸቡ  __  __  __  __  __  __  __ | | | | | | | |  |
|  | | **ሕቶ K ተመልከት/ቲ:** ዓይነት ትካል? | ሆስፒታል 1  ጥዕና ጣብያ 2ጥዕና ኬላ 3  ክሊኒክ 4  ፋርማሲ 5  መደብር መድሓኒት 6  7  ካልእ 8 | | | | | | | | | | | | | | | | | | | | | | | | | | | | | | | | | | 5፣6 ወይ ድማ 7 ተኾይኑ ናብ 8 ሕለፍ/ፊ |
| 4 | | ኣብ ጥዕና ትካልኩም ውሽጢ ኩሉ ግዘ ክርከብ ዝኽእል በዓል ሞያ ጥዕና (24 ዓት ዝሰርሕ) ወይድማ ንድንገተኛ ፃውዒት ስልኪ ኩሉ ግዘ (24 ሰዓታት) ግልጋሎት ክህብ ዝኽእል በዓል ሞያ ጥዕና ኣለኩም ዶ? | እወ፣24 ሰዓት ግልጋሎት ዝህቡ ሰራሕተኛታት ኣለው 1  ኣይፋሉን፣ 24 ሰዓት ግልጋሎት ዝህብ ሰራሕተኛ የብልናን 0 | | | | | | | | | | | | | | | | | | | | | | | | | | | | | | | | | |  |
| 5 | | ኣብዚ ጥዕና ትካልኩም ተጠቀምቲ በዝሒ ህዝቢ ማለት ትካልኩም ግልጋሎት ጥዕና ክህቦም ዝኽእል ጠቕላላ በዝሒ ህዝቢ ክንደይ ይኾኑ? | ኣይፋሉን፣፣ ትካልና ግልጋሎት ዝህቦም በዝሒ ህዝቢ ብቦታ ውሱን ኣይኮነን 1  እወ፣እቲ ትካል ዝህቦም በዝሒ ህዝቢ ቁፅሮም ይፍለጡ እዮም 2  ኣይፈለጥኩን፡ እቲ ትካል ዘገልግሎም በዝሒ ህዝቢ ኣይፈልጥዎን 3 | | | | | | | | | | | | | | | | | | | | | | | | | | | | | | | | | | ኣይፋሉን ወይድማ ኣይፈለጥኩን እንተኾይኑ ናብ 7 ሕለፍ/ፊ |
| 6 | | ጠቕላላ በዝሒ ተጠቀምቲ ክንደይ እዮም?  **ኣብዚ ከባቢ ነበርቲ ዝኾኑ ወይመ ትካልኩም ግልጋሎተ ዝህቦም ተጠቀምቲ በዝሒ ህዝቢ ምልኡ** | በዝሒ ተጠቀምቲ | | | | |  | | | | | | | | | | | | | | | | | | | | | | | | | | | | |  |
| 7 | | ትካልኩም ንሕሙማት ዝኸውን ክንደይ ዓራት ኣለዎ?  **0 መልሲ ክኸውን ይኽእል እዩ፡፡ ኣይፈለጥኩን እንተኾይኑ -88 ምልኡ** | በዝሒ ዓራት | | | | |  | | | | | | | | | | | | | | | | | | | | | | | | | | | | |  |
| 8 | | ናይዚ ትካል በዓል ዋና ወይድማ ናይ ደገ ተቖፃፃሪ ኣብ ቀረባ እዋን ዑደት ንምግባር ናብዚ ትካል መዓዝ መፂኡ ነይሩ? | ፍፁም ካብ ደገ ዝመፀ ተቖፃፃሪ የለን 0  ኣብ ውሽጢ ዝሓለፉ 6 ኣዋርሕ 1  ቅድሚ 6 ወርሒ 2  ኣይፈለጥኩን -88 | | | | | | | | | | | | | | | | | | | | | | | | | | | | | | | | | |  |
| 9 | | ሎመዓንቲ ኣብዚ ትካል ጥዕነና ግልጋሎት መብራህቲ (ኤሌክትሪክ) ኣሎ ‘ዶ? | እወ 1  ኣይፋሉን 0 | | | | | | | | | | | | | | | | | | | | | | | | | | | | | | | | | |  |
| 10 | | ሎመዓንቲ ኣብዚ ትካል ጥዕና ማይ ኣሎ ዶ? | እወ 1  ኣይፋሉን 0 | | | | | | | | | | | | | | | | | | | | | | | | | | | | | | | | | |  |
|  | | **ሕቶ K ተመልከት/ቲ:** ዓይነት ትካል? | ሆስፒታል 1  ጥዕና ጣብያ 2ጥዕና ኬላ 3  ክሊኒክ 4  ፋርማሲ 5  መደብር መድሓኒት 6  7  ካልእ 8 | | | | | | | | | | | | | | | | | | | | | | | | | | | | | | | | | | 5፣6 ወይ 7 ተኾይኑ ናብ 13 ሕለፍ/ፊ |
| 11 | | ኣብዚ ሐዚ እዋን ሰራሕተኛታት ዝጥቀምሉ ክንደይ መሕፀቢ ኢድ ቦታ ኣለኩም?  **ኣይፈለጥኩን እንተኾይኑ -88 ምልኡ** | በዝሒ ቦታ መሕፀቢ ኢድ | | | | | | | |  | | | | | | | | | | | | | | | | | | | | | | | | | | 0 ተኾይኑ ናብ 13 ሕለፍ/ፊ |
| 12 | | ዝቐረበ መሕፀቢ ኢድ ስፍራ ንኸርእዩኻ/ኺ ሕተት/ቲ፡፡  እዞም ቐፂሎም ዝቐረቡ ነገራት ኣብቲ መሕፀቢ ኢድ ስፍራ ምህላዎም ተመልከት/ቲ  ሳሙና ኣሎ  ዝዓቖረዝተዋህለለ) ማይ ኣሎ  ማይ ቧንቧ ኣሎ  እቲ መሕፀብ ኢድ ስፍራ ጥቓ ሽቓቕ እዩ ዝርከብ  ካብቶም ዝተዘርዘሩ ዋላ ሓደ  መሕፀብ ኢድ ቦታ ኣየርአዩንን  **ንዝምልከቶም ኮድ ምልኡ** |  | | | | | እወ  1  1  1  1  -88  1 | | | | | | | | | | | | | | | የለን  0  0  0  0  0 | | | | | | | | | | | | | |  |
| 13 | | ትካልኩም ኣብዚ ሐዚ እዋን ዝሰርሕ ኮምፒተር ኣለዎ ዶ?  **ኮምፒተር ምህላውን ዘይምህላውን ምርኣይ ኣድላዪ ኣይኮነን፡፡** | እወ 1  ኣይፋሉን 0 | | | | | | | | | | | | | | | | | | | | | | | | | | | | | | | | | |  |
|  | | **ሕቶ K ተመልከት/ቲ:** ዓይነት ትካል? | ሆስፒታል 1  ጥዕና ጣብያ 2ጥዕና ኬላ 3  ክሊኒክ 4  ፋርማሲ 5  መደብር መድሓኒት 6  7  ካልእ 8 | | | | | | | | | | | | | | | | | | | | | | | | | | | | | | | | | | 5፣6 ወይ 7 እንተኾይኑ ናብ 15 ሕለፍ/ፊ |
| 14 | | ኣብ ጥቕሚ ዝወዓሉ በላሕቲ ነገራትን በላሕቲ ነገራት ዝሓዙ ካርቶናትን ኣብ መወዳእታ ናብ ምንታይ/ብኸመይ ትጉሕፍዎሞ? | ዝውገዱ በላሕቲ ነገራት የብልናን 0  ኣብ ናይ ጎሓፍ መቃፀሊ ቢታ (ኢንሲኔተር) ነቃፅሎ 1  ክፍቲ ኣብ ዝኾነ ስፍራ ነቃፅሎ 2  እንተየቃፀልና ናብ መዋህለሊ ጎሓፍ ንጉሕፎ 3  ካብ ግቢ ወፃኢ ናብ ዘይረአ ቦታ ኣርሒቕና ንጉሕፎ 4  ካልእ 5 | | | | | | | | | | | | | | | | | | | | | | | | | | | | | | | | | |  |
| **ክፍሊ 2-ግልጋሎት ምጣነ ስድራ**  **ሐዚ ድማ ኣብ ትካልኩም ብዛዕባ ዝወሃቡ ግልጋሎት ምጣነ ስድራ ብዝምልከት ዝተወሰኑ ሕቶታት ክሓተኩም እየ፡፡** | | | | | | | | | | | | | | | | | | | | | | | | | | | | | | | | | | | | | |
| 15 | ትካልኩም ዝበዝሕ ግልጋሎት ምጣነ ስድራ/ጥንሲ ክከላኸሉ ወይድማ ከዘንግዑ ዝኽእሉ ሜላ መከላኸሊ ጥንሲ ግልጋሎት ትህቡ/ተቕርቡ ዶ? | | እወ 1  ኣይፋሉን 0 | | | | | | | | | | | | | | | | | | | | | | | | | | | | | | | | | | ኣይፋሉን ተኾይኑ ናብ 19 ሕለፍ/ፊ |
| 16 | ትካልኩም ግልጋሎት ምጣነ ስድራ/ጥንሲ ክከላኸሉ (ከዘንግዑ) ዝኽእሉ ሜላ መከላኸሊ ጥንሲ ምሃብ ዝጀመረሉ ዓመተምሕረት መዓዝ እዩ?  **ኣይፈለጥኩን ተኾይኑ 2020 ምልኡ** | | ዓመተምሕረት | | | | | | | |  | | | | | | | | | | | | | | | | | | | | | | | | | |  |
| 17 | ትካልኩም ኣብ ሰሙን ንኽንደይ መዓልቲ ዝኸውን ግልጋሎት ምጣነ ስድራ/ጥንሲ ክከላኸሉ ወይድማ ከዘንግዑ ዝኽእሉ ሜላ መከላኸሊ ጥንሲ ይህብ/ይሸጥ?  **በዝሒ መዓልታት ንምስላሕ ኣብ ሰሙን 7 መዓልታት ተጠቐም/ሚ፡፡ካብ 1-7 ዘለው መዓልታት ጥራሕ ምልኡ፡፡መልሶም ኣይፈለጥኩን ተኾይኑ -88 ምልኡ** | | በዝሒ መዓልታት | | | | | | | |  | | | | | | | | | | | | | | | | | | | | | | | | | |  |
| 18 | ሎመዓንቲ ትካልኩም ግልጋሎት ምጣነ ስድራ/ጥንሲ ክከላኸሉ ወይድማ ከዘንግዑ ዝኽእል ሜላ እናሃበ ድዩ? | | እወ 1  ኣይፋሉን 0 | | | | | | | | | | | | | | | | | | | | | | | | | | | | | | | | | |  |
|  | **ሕቶ ክ ተመልከት/ቲ:** ዓይነት ትካል? | | ሆስፒታል 1  ጥዕና ጣብያ 2ጥዕና ኬላ 3  ክሊኒክ 4  ፋርማሲ 5  መደብር መድሓኒት 6  7  ካልእ 8 | | | | | | | | | | | | | | | | | | | | | | | | | | | | | | | | | | 5፣6፣7 እንተኸይኑ ናብ 23 ሕለፍ/ፊ |
| 19 | ትካልኩም ንጉጅለ ልምዓት ጥዕና ኣብ ምጣነ ሰድራ ክትትል ፣ሐገዝ ወይድማ ኣቕርቦት ይግበረለን ዶ? | | እወ 1  ኣይፋሉን 0 | | | | | | | | | | | | | | | | | | | | | | | | | | | | | | | | | | የለን ተኾይኑ ናብ 22 ሕለፍ/ፊ |
| 20 | ብትካልኩም ድጋፍ ዝግበረለን በዝሒ ጉጅለ ልምዓት ጥዕና ክንደይ እየን?  **ኣይፈለጥኩን እንተኾይኑ -88 ምልኡ** | | በዝሒ ጉጅለ ልምዓት ጥዕና | | | | | | | | |  | | | | | | | | | | | | | | | | | | | | | | | | |  |
| 21 | ቐፂሎም ካብ ዝቐረቡ ሜላ መከላኸሊ ወይድማ መዘንግዒ ጥንሲ ብጉጅለ ልምዓት ጥዕና ዝቐርቡ/ዝወሃቡ ኣየነኦም እዮም  ኮንደም  ጥንሲ ዝከላኸል ክኒን (ፒልስ)  ጥንሲ ዝከላኸል መርፍእ | |  | | | | | | | | | | | | | | | እወ  1  1  1 | | | | | | | | | | | | | የለን  0  0  0 | | | | | |  |
| 22 | ኣብ ዝሓለፉ12 ኣዋርሕ ተንቀሳቐስቲ ጉጅለ ጥዕና ተወሳኺ ግልጋሎት ምጣነ ስድራ ንምሃብ ናብ ትካልኩም ክንደይ ግዘ መፂኦም? | | ዝመፅሉ በዝሒ | | | | | | | | | | | | | | |  | | | | | | | | | | | | | | | | | | |  |
|  | **ሕቶ 15 1መልከት:** ግልጋሎት ምጣነ ስድራ/ሜላ መከላኸሊ ወይድማ መዘንግዒ ጥንሲ ይህቡ/የቕርቡ ዶ? | | እወ 1  ኣይፋሉን 0 | | | | | | | | | | | | | | | | | | | | | | | | | | | | | | | | | | ኣይፋሉን ተኾይኑ ናብ 25 ሕለፍ/ፊ |
| 23 | ትካልኩም ንዝህቦም ግልጋሎት ወይድማ ምስ ምጣነ ስድራ ዝተተሓሓዙ ዝኾኑ ዓይነት ግልጋሎት ንምሃብ ንተገልገልቲ ዘኽፍሎ ክፍሊት ኣሎ ዶ?  **እቲ ክፍሊት ንተገልገልቲ መመዝገቢ ወይድማ ካርዲ መውፅኢ ሓዊሱ ዝኾነ ዓይነት ክፍሊት እዩ ዘጠቓልል፡፡** | | እወ 1  ኣይፋሉን 0 | | | | | | | | | | | | | | | | | | | | | | | | | | | | | | | | | | ኣይፋሉን እንተኸይኑ ናብ 25 ሐለፍ/ፊ |
| 24 | ሕጋዊ ዝኾነ ክፍሊት ዝሓዘ ተገልገልቲ ብቐሊሉ ክሪእዎ ኣብ ዝኽእሉ ስፍራ ዝተለጠፈ ዝርዝር ዋጋ ክፍሊት ኣለኩም ዶ?  **እወ እንተኾይኑ ዝተለጠፈ ዝርዝር ክፍሊት ግዴታ ተመልከት/ቲ፡፡** | | እወ፣ናይ ኩሎም ዝርዝር ክፍሊት ተለጢፉ 1  እወ፣ናይ ዝተወሰኑ ዝርዝር ክፍሊት ተለጢፉ 2  ዝተለጠፈ ዓይነት ዝርዝር ክፍሊት የለን 0 | | | | | | | | | | | | | | | | | | | | | | | | | | | | | | | | | |  |
| 25 | ድልየት (ሓሳብ)፣ ሪኢቶ ተገልገልቲ ንምፍላጥ ብዞም ዝስዕቡ ሜላታት ትጥቀሙ ‘ዶ?  ሓሳብ መውሃቢ ሳፁን  ሓሳብ ተገልገልቲ ዝህብሉ ቅጥዒ  ቃለመሕትት መግበሪ ቅጥዒ  ምስ መራሕቲ ኣካላት ማሕበረሰብ ኣኼባ ብምክያድ  ምስ ተገልገልቲ ወይድማ ማሕበረሰብ ዝግበር ዘይስሩዕ ዘተ  ተገልገልቲ ንሰራሕተኛታት ብቐጥታ ዝህብዎ ርኢቶ  ካልእ  ኣይፈለጥኩን………………………………….  ካብዞም ዝተገለፁ ዝግበር ነገር የለን  **ኹሎም ዝምልከቶም ኮድ ምልኡ** | |  | | | | | | | | | | | | | | | እወ  1  1  1  1  1  1  1  -88  1 | | | | | | | | | | | | | የለን  0  0  0  0  0  0  0  0 | | | | | | ዝግበር ነገር የለን ተኾይኑ ናብ 29 ሕለፍ/ፊ |
| 26 | ካብ ተገልገልቲ ዝኣከብክምዎ ሓሳብ ወይድማ ሪኢቶ ብዝተጠናኸረ መንገዲ ሪፖርት ንምግባር ትኽተልዎ ኣከያይዳ ኣሎ ዶ? | | እወ 1  ኣይፋሉን 0 | | | | | | | | | | | | | | | | | | | | | | | | | | | | | | | | | | ኣይፋሉን እንተኾይኑ ናብ 28 ሕለፍ/ |
| 27 | ዝተጠናኸረ ሪፖርት ወይ ድማ ብኣግባቡ ዝተቐመጠ ፅሑፍ ንኸርእዩኹም ሕተቱ። | | ሪፖርት ተርእዩ 1  ሪፖርት ኣይተርኣየን 2 | | | | | | | | | | | | | | | | | | | | | | | | | | | | | | | | | |  |
| 28 | ኣብ ዝሓለፉ 12 ኣዋርሕ ሓሳብ (ድልየት)፣ ሪኢቶ ተገልገልቲ መሰረት ብምግባር ዝቐየርኩምዎ መደብ ወይም ኣሰራርሓ ኣሎ ዶ?  **እወ እንተኾይኑ ዝቀየርዎ መደብ ምስዞም ዝተዘርዘሩ ዝመሳሰል ብምምራፅ ንዝምልከቶም ምልኡ** | | የለን 0  እወ፣ዓይነት ግልጋሎት፣ዝወሃበሉ ሰዓት ወይድማ ግልጋሎት ዝወሃበሉ መንገዲ ተቐይሩ 1  እወ፣ንተገልገልቲ ከም ዝጥዕም ተገይሩ 2  ካልእ 3  ኣይፈለጥኩን -88 | | | | | | | | | | | | | | | | | | | | | | | | | | | | | | | | | |  |
| 29 | ኣብ ዝሓለፉ 12 ኣዋርሕ ብዛዕባ ዝተውሃቡ በዝሒ /ዝርዝር ግልጋሎት ምጣነ ስድራ ብዝምልከት ምስ ሰራሕተኛታት ኣኼባ/ዘተ ገይርኩም ዶ ነይርኩም? | | እወ 1  ኣይፋሉን 0 | | | | | | | | | | | | | | | | | | | | | | | | | | | | | | | | | |  |
| 30 | ንኣወሃህባ ግልጋሎት መረዳእታ ብዝግባእ ንምርኣይ፣ ንምክትታልን ንምግምጋምን ካብዞም ዝስዕቡ ዝርዝራት ኣየናይ ሜላ ተጠቂምኩም  ቻርት/ኣብ ግድግዳ ዝልጠፍ ግራፍ  ዝተፅሓፈ ሪፖርት/ቃለጉባኤ  ካልእ  ፍፁም ዝተርኣየ ነገር የለን  **ዝተውሃቡ ዓይነት ግልጋሎት ዘመላኽቱ ዝተኸለሱ መረዳእታታት ማለት ሪፖርታት፣ ቻርት ወይድማ ግራፍ መንደቕ ንኸርእዩኻ ሐተት/ቲ፡፡**    **ንዝተመልከትክምዎም ኩሉም ሜላታትምስ ዝተዘርዘሩ ዝመሳሰል ብምምራፅ ንዝምልከቶም ኮድ ምልኡ፡፡** | |  | | | | | | | | | | | | | | | | እወ  1  1  1  1 | | | | | | | | | | | | | የለን  0  0  0  0 | | | | |  |
|  | **ሕቶ 15 ተመልከት:** ትካሎም ግልጋሎት ምጣነ ስድራ ይህብ ዶ? | | እወ 1  ኣይፋሉን 0 | | | | | | | | | | | | | | | | | | | | | | | | | | | | | | | | | | ኣይፋሉን ተኾይኑ ናብ 40 ሕለፍ/ፊ |
| 31 | ቐፂሎም ካብ ዝተዘርዘሩ ዓይነት መከላኸሊ/መዘንግዒ ጥንሲ ትካልኩም ግልጋሎት ምኽሪ ዝሃበሎም፣ንተገልገልቲ ዘቕረቦም ወይድማ ኣብ ካልእ ኽይዶም ንኽጥቀሙ መአዘዚ ወረቐት/ሪፈር ዝተፅሓፈሎም ዓይነት ሜላ መከላኸሊ/ መዘንግዒ ጥንሲ ኣየንኦም እዮም?  ካብዞም ዝተዘርዘሩ ዓይነት ሜላ መከላኸሊ/መዘንግዒ ጥንሲ ትካልኩም ክፍሊት ተኽፍሉ ዶ?  ደቂ ኣንስትዮ ብኦፕራስዮን ምምካን  ደቂ ተባዕትዮ ብኦፕራስዮን ምምካን  ኣብ ማህፀን ዝቕበር ሉፕ  ንዘጥቡው ኣዴታት መከላኸሊ ክኒን  መከላኸሊ መርፍእ (ን3 ወርሒ ዝወሃብ)  ኣብ ኩርናዕ ዝቕበር  መከላኸሊ ጥንሲ ክኒን  ናይ ወዲ ኮንደም  ናይ ጓል ኮንደም  ድንገተኛ መከላኸሊ ክኒን    ዑንቂ ዝመስልነገር ብምጥቃም ዑደት ወርሓዊ ፅግያት ምክትታል  ህፃን ጡብ ኣዶ ብቻ ምጥባው (ላም)  መዓልቲ ምቑፃር  ፈሳሲ ወዲ ተባዕታይ ካብ ብልዕቲ ጓል ተንስተይቲ ወፃኢ ብምፍስስ  ካልእ ባህላዊ ሜላ  ምኽ: ግልጋሎት ምኽሪ; ኣቕ: ኣቕርቦት; ሪፈ: ሪፈር ክፍ፡ክፍሊት  **ኩሎም መማረፅታት ዋዕ ኢልካ/ኪ ኣንብበሎም/ኣንብብሎም፡፡** | | ምኽ  እወ  1  1  1  1  1  1  1  1  1  1  1  1  1  1  1  1  1 | ምኽ የለን  0  0  0  0  0  0  0  0  0  0  0  0  0  0  0  0  0 | | ኣቕ እወ  1  1  1  1  1  1  1  1  1  1  1  1  1  1 | | | ኣቕ የለን  0  0  0  0  0  0  0  0  0  0  0  0  0  0 | | | | | ሪፈ እወ  1  1  1  1  1  1  1  1  1  1  1  1  1  1 | | | | | | | | | | ሪፈ የለን  0  0  0  0  0  0  0  0  0  0  0  0  0  0 | | | | | | | | | | | | ክፍ  እወ  1  1  1  1  1  1  1  1  1  1  1  1  1  1 | ምንም ዓይነት ክፍሊት ተዘይሃልዩ ናብ 33 ሕለፍ/ፊ |
| 32 | ንሕድሕድ ሜላ መከላኸሊ ጥንሲ ሓደ ግዘ ግልጋሎት ንምሃብ ክንደይ ተኽፍሉ?  ደቂ ኣንስትዮ ብኦፕራስዮን ምምካን  ደቂ ተባዕትዮ ብኦፕራስዮን ምምካን  ኣብ ማህፀን ዝቕበር ሉፕ  ንዘጥቡው ኣዴታት መከላኸሊ ክኒን  መከላኸሊ መርፍእ (ን3 ወርሒ ዝወሃብ)  ኣብ ኩርናዕ ዝቕበር  መከላኸሊ ጥንሲ ክኒን  ናይ ወዲ ኮንደም  ናይ ጓል ኮንደም  ድንገተኛ መከላኸሊ ክኒን    ዑንቂ ዝመስል ነገር ብምጥቃም ዑደት ወርሓዊ መዓልቲ ምቑፃር  ፈሳሲ ወዲ ተባዕታይ ካብ ብልዕቲ ጓል ተንስተይቲ ወፃኢ ብምፍስስ  ካልእ ባህላዊ ሜላ  **ንኹሎም ክፍሊት ብብር ምልኡ**  *እቲ ትካል ዘኽፈሎም ዓይነት ሜላ መከላኸሊ ጥንሲ ODK ካብ ሕቶ 31 ከርእየኩም እዩ* | | ክፍሊት ንሓደ ግዘ ግልጋሎት | | | | | | | | | | | | | | | | | | | | | _________  _____________________________________________________________________________________________________________________ | | | | | | | | | | | | |  |
|  | **ሕቶ K ተመልከት/ቲ:** ዓይነት ትካል? | | ሆስፒታል 1  ጥዕና ጣብያ 2ጥዕና ኬላ 3  ክሊኒክ 4  ፋርማሲ 5  መደብር መድሓኒት 6  ካልእ 7 | | | | | | | | | | | | | | | | | | | | | | | | | | | | | | | | | | 5፣6፣7 እንተኾይኑ ናብ 39b ሕለፍ/ፊ |
|  | **ሕቶ 31 ተመልከት/ቲ፡**ኣብ ኩርናዕ ዝቕበር (ኢምፕላንትስ) የቕርቡ ዶ? | | እወ 1  ኣይፋሉን 0 | | | | | | | | | | | | | | | | | | | | | | | | | | | | | | | | | | ኣይፋሉን ተኾይኑ ናብ 35 ሕለፍ/ፊ |
| 33 | ግልጋሎት ምጣነ ስድራ ኣብ ትህብሉ እዋን ትካልኩም ኣብ ኩርናዕ ዝቕበር መከላኸሊ (ኢምፕላንትስ)) ኣብ ኩርናዕ ከእትው ዝኽእል ዝሰልጠነ በዓል ሞያ ኣለዎ ዶ? | | እወ 1  ኣይፋሉን 0 | | | | | | | | | | | | | | | | | | | | | | | | | | | | | | | | | |  |
| 34 | ግልጋሎት ምጣነ ስድራ ኣብ ትህብሉ እዋን ትካልኩም ኣብ ኩርናዕ ዝቕበር መከላኸሊ (ኢምፕላንትስ) ካብ ኩርናዕ ከውፅእ ዝኽእል ዝሰልጠነ በዓል ሞያ ኣለዎ ‘ዶ? | | እወ 1  ኣይፋሉን 0 | | | | | | | | | | | | | | | | | | | | | | | | | | | | | | | | | |  |
|  | **ሕቶ 31 ተመልከት/ቲ:** ግልጋሎት ኣብ ማህፀን ዝቕበር ሉፕ (ኣዩዲ/ሉፕ) የቕርቡ/ይህቡ ዶ? | | እወ 1  ኣይፋሉን 0 | | | | | | | | | | | | | | | | | | | | | | | | | | | | | | | | | | ኣይፋሉን እንተኾይኑ ናብ 37 ሕለፍ/ፊ |
| 35 | ግልጋሎት ምጣነ ስድራ ኣብ ትህብሉ እዋን ትካልኩም ኣብ ማህፀን ዝቕበር ሉፕ (ኣዩዲ/ሉፕ) ናብ ውሽጢ ማህፀን ከእትው ዝኽእል ዝሰልጠነ በዓል ሞያ ኣለዎ ዶ? | | እወ 1  ኣይፋሉን 0 | | | | | | | | | | | | | | | | | | | | | | | | | | | | | | | | | |  |
| 36 | ግልጋሎት ምጣነ ስድራ ኣብ ትህብሉ እዋን ትካልኩም ኣብ ማህፀን ዝቕበር ሉፕ (ኣዩዲ/ሉፕ) ካብ ውሽጢ ማህፀን ከውፅእ ዝኽእል ዝሰልጠነ በዓል ሞያ ኣለዎ ዶ? | | እወ 1  ኣይፋሉን 0 | | | | | | | | | | | | | | | | | | | | | | | | | | | | | | | | | |  |
|  | **ሕቶ 31 ተመልከት/ቲ:** ኣብ ኩርናዕ ዝቕበር መከላኸሊ (ኢምፕላንትስ) የቕርቡ/ግልጋሎት ይህቡ ዶ? | | እወ 1  ኣይፋሉን 0 | | | | | | | | | | | | | | | | | | | | | | | | | | | | | | | | | | ኣይፋሉን ተኾይኑ ናብ 38 ሕለፍ/ፊ |
| 37 | ትካልኩም እዞም ቐፂሎም ዝቐረቡ ኣብ ኩርናዕ ዝቕበር መከላኸሊ (ኢምፕላንትስ) ኣብ ውሽጢ ኩርናዕ ንምቕባርን ካብ ኩርናዕ ንምውፃእን ከገልግሉ ዝኽእሉ ናውቲ ሕክምና ኣለዎ ዶ?  ፅርየቶም ዝሓለው ጓንቲ  መወገዲ ህዋሳት (ኣንቲሴፕቲክ)  ፅርየቶም ዝሓለው / ካብ ጀርሚ ነፃ ዝኾኑ ፋሻ/ጡጥ  መደምዘዚ/ፀረ­-ቃንዛ  ዝተዓሸገ ፓኬት ኢምፕላንት  ላማ/መላፀ  **ንኩሎም ዝተዘርዘሩ ዓይነት ናውቲ ሕክምና ምህላዎምን ዘይምህላዎምን ብምሕታት ንዝምለከቶም ኮድ ምልኡ፡፡ነቶም ናውቲ ሕክምና ምርኣይ ኣድላዪ ኣይኮነን፡፡** | |  | | | | | | | | | | | | | | እወ  1  1  1  1  1  1 | | | | | | | | | | | | | የለን  0  0  0  0  0  0 | | | | | | |  |
|  | **ሕቶ 31 ተመልከት/ቲ:** ኣብ ማህፀን ዝቕበር መከላኸሊ ጥንሲ (ኣዩዲ/ሉፕ) የቕርቡ/ይህቡ ዶ? | | እወ 1  ኣይፋሉን 0 | | | | | | | | | | | | | | | | | | | | | | | | | | | | | | | | | | ኣፋሉን ተኾይኑ ናብ 39 ሕለፍ/ፊ |
| 38 | ትካልኩም እዞም ቐፂሎም ዝቐረቡ ኣዩዲ/ሉፕ ናብ ውሽጢ ማህፀን ኣብ ምእታውን ካብ ውሽጢ ማህፀን ኣብ ምውፃእን ከገልግሉ ዝኽእሉ ናውቲ ሕክምና ኣለዎ ዶ?  ስፖንጅ ንምትሓዝ ዘገልግል መቐስ Sponge-holding forceps  ማህፀን ንምኽፋት/ኣስፊሑ ንምርኣይ ዘገልግል መሳርሒ (ዓብዪን ማእኸላይን) Speculums (large and medium)  ርስሓት ንውጋድ/ንምልዓል ዘገልግል መሳርሒ Tenaculum  ኣብ እዋን መጥባሕቲ ነገራት ቆንጢጡ ዝሕዝ መሳርሒ Clamp  **ንኹሎም ዝተዘርዘሩ ዓይነት ናውቲ ሕክምና ምህላዎምን ዘይምህላዎምን ብምሕታት ንዝምለከቶም ምልኡ፡፡ነቶም ናውቲ ሕክምና ምርኣይ ኣድላዪ ኣይኮነን፡፡** | |  | | | | | | | | | | | | | | | እወ  1  1  1  1 | | | | | | | | | | | | የለን  0  0  0  0 | | | | | | |  |
|  | **ሕቶ K** **ተመልከት/ቲ፡** ዓይነት ትካል? | | ሆስፒታል 1  ጥዕና ጣብያ 2ጥዕና ኬላ 3  ክሊኒክ 4  ፋርማሲ 5  መደብር መድሓኒት 6  ካልእ 7 | | | | | | | | | | | | | | | | | | | | | | | | | | | | | | | | | | ካብ 1-4 ወይ 8 ተኾይኑ ናብ 39a ሕለፍ/ፊ 5፣6 ወይ 7 እንተኾይኑ ናብ  39b ሕለፍ/ፊ |
| 39a | **ካብ መዝገብ ግልጋሎት ምጣነ ስድራ ብምርኣይ መዝግብ/ቢ**  **(1)** ኣብ ውሽጢ ዝሓለፈ ሓደ ወርሒ ሜላ ምጣነ ስድራ/መከላኸሊ ወይድማ መዘንግዒ ጥንሲ ንምርካብ ኢለን ናብ ትካልኩም ዝመፃ (ሓደሽትን ነባራትን) ተገልገልቲ ብጠቕላላ  (ንሕድ ሕድ ሜላ መከላኸሊ ኣብቲ ክፍቲ ቦታ ምልኡ)  **(2)** ኣብ ወሽጢ ዝሓለፈ ሓደ ወርሒ ሜላ ምጣነ ስድራ/መከላኸሊ ወይድማ መዘንግዒ ጥንሲ ንምርካብ ኢለን ናብ ትካልኩም ዝመፃ በዝሒ ሓደሽቲ ተገልገልቲ ብጠቕላላ  (ንሕድሕድ ሜላ ኣብቲ ክፍቲ ቦታ ምልኡ) | | ደቂ ኣንስትዮ ብኦፕራስዮን ምምካን  ደቂ ተባዕትዮ ብኦፕራስዮን ምምካን  ኣብ ማህፀን ዝቕበር መከላኸሊ ጥንሲ (ኣዩዲ/ሉፕ)  ንዘጥብዋ ኣዴታት ዝወሃብ መከላኸሊ ጥንሲ    ናይ 3 ወርሒ መርፍእ  ኣብ ኩርናዕ ዝቕበር መከላኸሊ (ኢመፕላነትስ)  መከላኸሊ ክኒን  ናይ ወዲ ኮንደም  ናይ ጓል ኮንደም  ድንገተኛ መከላኸሊ ክኒን    ዕንቁ ዝመስል ነገር ብምጥቃም ወርሓዊ ፅግያ ምክትታል  ፀባ ጡብ ኣዶ ጥራሕ ምጥባው  መዓልቲ ምቑፃር  ፈሳሲ ወዲ ተባዕታይ ካብ ብልዕቲ ጓል ተንስተይቲ ወፃኢ ብምፍስስ  ካልእ ባህላዊ ሜላ | | | | | | | | | | | | # ጠቕላላ ዝመፃ  ___  ___  ___  ___  ___  ___  ___  ___  ___  ___  ___  ___  ___  ___  ___  ___ | | | | | | | | | | | | | # ሓደሽቲ ተገልገልቲ  ___  ___  ___  ___  ___  ___  ___  ___  ___  ___  ___  ___  ___  ___  ___  ___ | | | | | | | | |  |
| 39b | **ካብ መዝገብ ግልጋሎት ምጣነ ስድራ ብምርኣይ መዝግብ/ቢ**  ኣብ ውሽጢ ዝሓለፈ ሓደ ወርሒ ብትካሎም ዝተሸጡ በዝሒ ሕድ ሕድ ሜላ መከላኸሊ ጥንሲ ምልኡ | | ኣብ ማህፀን ዝቕበር መከላኸሊ ጥንሲ (ኣዩዲ/ሉፕ)    ንዘጥብዋ ኣዴታት ዝወሃብ መከላኸሊ ጥንሲ  ናይ 3 ወርሒ መርፍእ  ኣብ ኩርናዕ ዝቕበር መከላኸሊ (ኢመፕላነትስ)  መከላኸሊ ጥንሲ ክኒን  ናይ ወዲ ኮንደም  ናይ ጓል ኮንደም  ድንገተኛ መከላኸሊ ክኒን  ዲያፍራም  ፎም/ጄሊ  ዕንቁ ዝመስል ነገር ብምጥቃም ወርሓዊ ፅግያ ምክትታል  ካልእ ባህላዊ ሜላ | | | | | | | | | | | | | | | | | | | | | | | ዝተሸጡ በዝሒ ሜላ  ___  ___  ___  ___  ___  ___  ___  ___  ___  ___  ___  ___ | | | | | | | | | | |  |
|  | **ሕቶ K** **ተመልከት/ቲ፡** ዓይነት ትካል? | | ሆስፒታል 1  ጥዕና ጣብያ 2ጥዕና ኬላ 3  ክሊኒክ 4  ፋርማሲ 5  መደብር መድሓኒት 6  ካልእ 7 | | | | | | | | | | | | | | | | | | | | | | | | | | | | | | | | | | 5፣6 ወይ 7 እንተኾይኑ ናብ 45 ሕለፍ/ፊ |
| 40 | ካብዞም ቐፂሎም ዝተዘርዘሩ ዓይነታት ግልጋሎት ብትካልኩም ዝወሃቡ መኒኦም እዮም  ክትትል ቅድመ ወሊድ  ግልጋሎት ወሊድ  ክትትል ድሕረ ወሊድ  ጥንሲ ድሕሪ ምስዳድ ዝግበር ክትትል  **ንኩሎም ዝተዘርዘሩ ብምንባብ ንዝምልከቶም ኮድ ምልኡ** | |  | | | | | | | | | | | | | | | | | | | እወ  1  1  1  1 | | | | | | | | | | | | የለን  0  0  0  0 | | | ንክትትል ድሕረወሊድን ድሕሪ ጥንሲ ምስዳድን የለን እንተኾይኑ ናብ 45 ሕለፍ/ፊንክትትል ድሕረ ወሊድ የለን ንክትትል ድሕሪ ጥንሲ ምስዳድ እወ ተኾይኑ ናብ 43 ሕለፍ/ፊ |
| 41 | ሓንቲ ኣዶ ወሊዳ ካብ ትካልኩም ቅድሚ ምውፃኣ ብዛዕባ ቐፂሎም ዝቐረቡ ግልጋሎት ንኣዶ ተዋሂቡዋ ‘ዶ?  ብዛዕባ ዝተስተኻኸለ ኣመጋግባ፣ስርዓተ ምግብን ምንቅስቓስ ሰውነት ምግባርን  ድሕሪ ወሊድ ክፍጠር ዝኽእል ሕማም ርእሲ  ብዛዕባ ቐፃላይ ወሊድ  ብዛዕባ ምርሕሓቕ/ምፍንታት ጥንስን ጥዕንኡ ዝሓለወ እዋን ጥንስን  ምኽሪ ብዛዕባ ሜላ መከላኸሊ ጥንሲ  ሜላ ላክተሸናል ኣሚኖሪያ /ላም (ን6 ወርሒ ዝኸውን ብዘይምቁራፅ ፀባ ጡብ ኣዶ ጥራሕ ምምጋብ)  ናይ ወርሓዊ ፅግያ መዓልቲ ብምቑፃር  ንነዊሕ እዋን ዘገልግሉ ሜላ መከላኸሊ ጥንሲ  ኣረሓሒቕካ ምውላድ  ብዛዕባ እዚኣቶም ተዛቲና ኣይንፈልጥን  **ንሕድ ሕድ ዝተዘርዘሩ ብምንባብ ንዝምልከቶም ኮድ ምልኡ** | |  | | | | | | | | | | | | | | | | | | እወ  1  1  1  1  1  1  1  1 | | | | | | | | | | | | የለን  0  0  0  0  0  0  0  0 | | | |  |
| 42 | ክትትል ድሕሪ ወሊድ ንምግባር ናብ ትካልኩም ዝመፃ ኣዴታት ግልጋሎት ሜላ መከላኸሊ/መዘንግዒ ጥንሲ ይወስዳ ዶ? | | እወ 1  ኣይፋሉን 0 | | | | | | | | | | | | | | | | | | | | | | | | | | | | | | | | | |  |
|  | **ሕቶ 40 ተመልከት/ቲ:** ትካሎም ክትትል/ ምርመራ ድሕሪ ጥንሲ ምስዳድ ይገብር ዶ? | | እወ 1  ኣይፋሉን 0 | | | | | | | | | | | | | | | | | | | | | | | | | | | | | | | | | | የለን ተኾይኑ ናብ 45 ሕለፍ/ፊ |
| 43 | ግልጋሎት ክትትል ድሕሪ ጥንሲ ምስዳድ ንምግባር ናብ ትካልኩም ምስ ዝመፃ ተገልገልቲ ብዛዕባ ቐፂሎም ዝተዘርዘሩ ነገራት ትመያየጡ ዶ?  ድሕሪ ወሊድ ክፍጠር ዝኽእል ሕማም ርእሲ  ብዛዕባ ቐፃላይ ወሊድ  ብዛዕባ ምርሕሓቕ ጥንስን ጥዕንኡ ዝሓለወ ጥንስን  ምኽሪ ብዛዕባ ሜላ መከላኸሊ ጥንሲ  ንነዊሕ እዋን ዘገልግሉ ሜላ መከላኸሊ ጥንሲ  ጥንሲ ከዘንግዑ ዝኽእሉ ሜላመከላኸሊ ጥንሲ  ብዛዕባ እዚኣቶም ተዛቲና ኣይንፈልጥን  **ንሕድ ሕድ ዝተዘርዘሩ ብምንባብ ንዝምልከቶም ኮድ ምልኡ** | |  | | | | | | | | | | | | | | | | | | እወ  1  1  1  1  1  1 | | | | | | | | | | | | የለን  0  0  0  0  0  0 | | | |  |
| 44 | ግልጋሎት ክትትል ድሕሪ ምስዳድ ጥንሲ ንምርካብ ናብ ትካልኩም ዝመፃ ደቂ ኣንስትዮ ኣብ ዝመፃሉ እዋን ሜላ መከላኸሊ ጥንሲ ይወስዳ ዶ? | | እወ 1  ኣይፋሉን 0 | | | | | | | | | | | | | | | | | | | | | | | | | | | | | | | | | |  |
| 45 | ካብዞም ቐፂሎም ዝተዘርዘሩ ግልጋሎት ምጣነ ስድራ ትካልኩም ንዘይተመርዐዋ ደቂኣንስትዮ (ጎርዞ) ዝህቦም ግልጋሎት ኣየኖት እዮም?  ብዛዕባ ሜላ መከላኸሊ ጥንሲ ምምኻር  ግልጋሎት ሜላ መከላኸሊ ጥንሲ ምሃብ/ ምቕራብ  ብዛዕባ ሜላ መከላኸሊ ጥንሲ ናብ ካልእ ቦታ ከይደን ንኽጥቀማ ሪፈር ምፅሓፍ  ካብቶም ዝተዘርዘሩ ትካልና ዝህቦ ግልጋሎት የለን  **ንሕድሕድ ዝተዘርዘሩ ብምንባብ ንዝምልከቶም ኮድ ምልኡ** | |  | | | | | | | | | | | | | | | | | እወ  1  1  1  1 | | | | | | | | | | | | | | የለን  0  0  0  0 | | |  |
|  | ትካልኩም ድንገተኛ ናይ ወሊድን ናይ ህፃናትን ምንክብክብ ግልጋሎትን ብዝተማለአ መልክዑ ከምዝህብ ካብዚ ንታሕቲን ዝተዘርዘሩ ግልጋሎትን ብምንባብ ሕተት/ቲ።  ብሱር ደም ዝወሃብ ፀረ­-ተሃዋስያን (Parental Antibiotics)  ናይ Eclampsia/Preeclampsia ሕክምና anticoagulant  ድሕሪ ወሊድ ንዘጋጥም ሕክምና መፍሰስቲ ደም (Uterotonics)  ኣብ ወሊድ እዋን ኣብ ማህፀን ዝተርፉ ናይ ጥንሲ ኣካላት ንምውፃእ ዘገልግል ሕከምና (Mnaual Vaccuum Aspiration)  ብ Vaccuum ዝተሓገዘ ወሊድ  ኢድ ብምጥቃመ መድሓንቲ ካበ ማህፀን ምውጋድ (Manual Removal of Placenta)  ተዓፊኖም ዝተወለዱ ዕሸላት ንክትንፍሱ ምግባር (Resuscitation) | |  | | | | | | | | | | | | | | | | |  | | | | | | | | | | | | | |  | | |  |
| 46 | ትካልኩም ምስ ብፃታዊ ርክብ ዝመሓላለፉ ሕማማት ዝተተሓሓዘ ዝኾነ ዓይነት ግልጋሎት ምርመራ፣ክትትል (ክንክን) ወይድማ ድጋፍ ይህብ/ይገብር ዶ? | | እወ 1  ኣይፋሉን 0 | | | | | | | | | | | | | | | | | | | | | | | | | | | | | | | | | |  |
| 47 | ትካልኩም ምስ ኤች.ኣይ ቪ ዝተተሓሓዘ ዝኾነ ዓይነት ግልጋሎት ምርመራ፣ ክትትል (ክንክን) ወይድማ ድጋፍ ይህብ/ይገብር ዶ? | | እወ 1  ኣይፋሉን 0 | | | | | | | | | | | | | | | | | | | | | | | | | | | | | | | | | | ኣይፋሉን ተኾይኑ ናብ 30 ሕለፍ/ፊ |
|  | **ሕቶ K** **ተመልከት/ቲ፡** ዓይነት ትካል? | | ሆስፒታል 1  ጥዕና ጣብያ 2ጥዕና ኬላ 3  ክሊኒክ 4  ፋርማሲ 5  መደብር መድሓኒት 6  ካልእ 7 | | | | | | | | | | | | | | | | | | | | | | | | | | | | | | | | | | 5፣6 ወይ 7 ተኾይኑ ናብ 52 ሕለፍ/ፊ |
| 48 | ሰባት ግልጋሎት ኤች.ኣይ/ቪ ንምርካብ ናብ ትካልኩም ኣብ ዝመፅሉ እዋን ካብዞም ቐፂሎም ዝተዘርዘሩ ዓይነት ግልጋሎት ምጣነ ስድራ ብትካልኩም ዝወሃቡ መኒኦም እዮም?  ብዛዕባ ሜላ መከላኸሊ ጥንሲ ምኽሪ ምሃብ?  ሜላ መከላኸሊ ጥንሲ ምቕራብ/ምሃብ?  ብዛዕባ ሜላ መከላኸሊ ጥንሲ ሪፈር ምፅሓፍ?  ካብቶም ዝተገለፁ ንህቦ ግልጋሎት የለን  **ንሕድ ሕድ ዝተዘርዘሩ ብምንባብ ንዝምልከቶም ኮድ ምልኡ** | |  | | | | | | | | | | | | | | | እወ  1  1  1  1 | | | | | | | | | | | | | የለን  0  0  0  0 | | | | | |  |
| 49 | ግልጋሎት ምኽሪ ኤች.ኣይ.ቪ ኣብ ትህብሉ እዋን ትካልኩም ንተገልገልቲ፡:  ንሓዋሩ ቖልዓ ንምውላድ ዘለዎም ሓሳብ (ድልየት) ትሓትዎም ዶ?  ብዛዕባ ዝመርፅዎም ዓይነት ሜላ መከላኸሊ ጥንሲ ተዛትይዎም ዶ?  ብዛዕባ ክልተ ዓይነት ሜላ ክጥቀሙ ከም ዝኽእሉ ተዛትይዎም ዶ?  ኮንደም ተቕርቡሎም ዶ?  ንዝመረፅዎ ዓይነት ሜላ መከላኸሊ(መዘንግዒ) ጥንሲ ብዛዕባ ዘለዎም ደካማ ጎኑን መምርሒታቱን ተዛትይዎም ዶ?  ሜላ ምጣነ ስድራ ተቕርቡሎም ዶ? | |  | | | | | | | | | እወ  1  1  1  1  1  1 | | | | | | የለን  0  0  0  0  0  0 | | | | | | | | | | | | | ኣይፈለጥኩን  -88  -88  -88  -88  -88  -88 | | | | | |  |
|  | **ሕቶ 15 ተመልከት/ቲ፡** ግልጋሎት ምጣነ ስድራ ይህቡ/የቕርቡ ዶ? | | እወ 1  ኣይፋሉን 0 | | | | | | | | | | | | | | | | | | | | | | | | | | | | | | | | | | ኣይፋሉን ተኾይኑ ናብ R ሕለፍ/ፊ |
| 50 | **ምርመራ ግልጋሎት ምጣነ ስድራ ዝገብርሉ ስፍራ ንኸርእዩኻ/ኺ ሕተት/ቲ**  **ቐፂሎም ዝተዘርዘሩ ሕድሕድ ናውቲ ምርመራ ግልጋሎት ምጣነ ስድራ ኣብ ዝወሃበሉ ስፍራ ወይድማ ኣብ ጥቕኡ ዘሎ ስፍራ ምህላዎምን ዘይምህላዎምን ብምርኣይ ንዝምልከቶም ኮድ ክበብ/ቢ፡፡**  [ንኢንፌክሽን ክከላኸሉ ዝኽእሉ ኣቕሑ/ናውቲ ተመልከት/ቲ]  ዝ፡ዝተርኣየ፤  ዘይ፡ዘሎ ኾይኑ ነገር ግን ዘይተርኣየ ኣይ፡ኣይምልከቶምን | | ማይ ቧንቧ  ካልእ ማይ ቧንቧ (ቧንቧ ዘለዎ ጀሪካን ወይድማ ባልዲ)  ኣብ ባልዲ ወይድማ ጀሪካን ዝተቐመጠ/ዝተዋህለለ ማይ  መሕፀብ ኢድ ሳሙና  ንሓደ ሻዕ ጥራሕ ዘገልግል ናይ ኢድ መዳረቒ ፎጣ  መኽደን ዘለዎ መጠራቐሚ/መዋህለሊ ጎሓፍ  መቐመጢ በላሕቲ ነገራት  ንሓደሻዕ ጥራሕ ዘገልግል ጓንቲ  ንኢንፌክሽን ዝከላኸል መድሓኒት  ንሓደ ሽዕ ጥራሕ ዘገልግሉ መርፍእን ስሪንጋን  ድምፂ ዘየሳምዕ ምርመራ ክፍሊ  ደገ ዘሎ ሰብ ናብ ወሽጢ ዘየራኢ ምርመራ ክፍሊ  ንምርመራ ዘገልግሉ ጠረጴዛ  ንተገልገልቲ መምሀሪ ምጣነ ስድራ ዘገልግሉ ናውቲ | | | | | | | | | | | ዝ  1  1  1  1  1  1  1  1  1  1  1  1  1  1 | | | | | | | | | | ዘይ  2  2  2  2  2  2  2  2  2  2  2  2  2  2 | | | | | | | | | | | | ኣይ  -77  -77  -77  -77  -77  -77  -77  -77  -77  -77  -77  -77  -77  -77 |  |
| 51 | **ግልጋሎት ምጣነ ሰድራ ዝወሃበሉ ስፍራ ተመልከት/ቲ** | | መሬት ገዛ፡ዝተፀረገ፣ዝረአ ርስሓት ዘይብሉ  ባንኮኒ/ጠረጴዛ/ወንበር፡ዝተፀረገ፣ ዝርአ ርስሓት ዘይብሉ  እቲ ስፍራ ብዝተሰበሩ ኣቕሑት፣ዝተደብረዩ ወረቓቕቲ ወይድማ ካርቶን ዝረሰሐ  መንደቕ፡ ብዝተወሰነ መልክዑ ፅርየቱ ዝሓለወ  ማዕፆ፡ምንም ወይድማ ዝተወሰነ ስብራት(ነቓዕ) ዘለዎ  መንደቕ፡ምንም ወይድማ ዝተወሰነ ስብራት(ነቓዕ) ዘለዎ  ክዳን ገዛ፡ምንም ወይድማ ዝተወሰነ ስብራት(ነቓዕ) ዘለዎ | | | | | | | | | | | | | | | | | | | | እወ  1  1  1  1  1  1  1 | | | | | | | | | | | | የለን  0  0  0  0  0  0  0 | |  |
| 52 | እዞም ቐፂሎም ዝተዘርዘሩ ዓይነት ሜላ መከላኸሊ ጥንሲ ትካልኩም ግልጋሎት ከም ዝህብ ነጊርኩምኒ ነይርኩም፡፡  እቶም ሜላ መከላኸሊ ክተርእዩኒ ዶ ትኽእሉ?  ንዘርኣይኹም ኩሎም ዓይነት ሜላ መከላኸሊ ጥንሲ ከም ዝስዕብ ሕተቱ፡፡  ኣብ ውሽጢ ዝሓለፉ 12 ኣዋርሕ ኣብ መጋዝንኩም/መቐመጢ መድሓኒት ዘይነበረሉ እዋን ነይሩ ዶ?  ኣብ ማህፀን ዝቕበር (ኣዩዲ/ሉፕ)  ንመጥበውቲ ኣዶታት ዝወሃበ መከላኽሊ    ናይ 3 ወርሒ መከለኸሊ መርፍእ  ኣብ ኩርናዕ ዝቕበር (ኢምፕላንትስ)  መከላኸሊ ክኒን  ናይ ወዲ ኮንደም  ናይ ጓል ኮንደም  ድንገተኛ ሜላ መከላኸሊ ክኒን      ዑንቒ ዝመስል ነገር ብምጥቃም ወርሓዊ ፅግያ ንምክትታል ዘገልግል  ካልኦት ባህላዊ ሜላ  ዝ፡ዝተርኣየ፣  ዘይ፡ዘይተርኣየ;  OOS፡ኣብ ዝሓለፉ12 ኣዋርሕ ኣብ መጋዝኖም ንሓደ ጊዘን ካብኡ ንላዕልን ዘይነበረ  **OOS ዝብል ኮድ ዝኽበብ ቃለ መሕትት ኣብ ዝካየደሉ እዋን ወላእኳ እቲ ሜላ መከላኸሊ እንተሃለወ ኣብ ዝሓለፉ 12 ኣዋርሕ ኣብ መጋዝኖም ንሓደ ግዘን ካብኡ ንላዕልን ዘይነበረሉ እዋን እንተሃልዩ እዩ፡፡ስለዚ OOS ንዝ፡ዝተኸበበሎምን ንዘይ፡ዝተኸበበሎምን ይሕተት ማለት እዩ፡፡**  **.***ዝርዝር ግልጋሎት ዝህብዎም ዓይነት ሜላ መከላኸሊ ODK ሕቶ SQ 31 መሰረት ብምግባር ከቕርበልኩም እዩ፡፡* | |  | | | | ዝ  1  1  1  1  1  1  1  1  1  1  1  1 | | | | | | ዘይ  0  0  0  0  0  0  0  0  0  0  0  0 | | | | | | | | | | | | | | OOS  ኣብ ዝሓለፉ12 ኣዋርሕ  1  1  1  1  1  1  1  1  1  1  1  1 | | | | | | | | | |  |
| 53 | **ካብ ሕቶ 53-56 ዘለው ሜላ መከላኸሊ ዘቕምጥሉ ቦታ ብምርኣይ ነዞም ዝስዕቡ ብምሕታት ዝምልከቶም ኮድ ምልኡ**  ኩሎም ሜላ መከላኸሊ/መዘንግዒ ጥንሲ ኣብ መሬት ካብ ምቕማጥ ዝረሓቑ ድዮም? | | እወ 1  ኣይፋሉን 0 | | | | | | | | | | | | | | | | | | | | | | | | | | | | | | | | | |  |
| 54 | ኩሎም ሜላ መከላኸሊ ማይ ኣብ ዘይረኽቦም ቦታ ድዮም ተቐሚጦም? | | እወ 1  ኣይፋሉን 0 | | | | | | | | | | | | | | | | | | | | | | | | | | | | | | | | | |  |
| 55 | ኩሎም ሜላ መከላኸሊ ፅሓይ ብርሃን ኣብ ዘይረኾቦም ቦታ ድዮም ተቐሚጦም? | | እወ 1  ኣይፋሉን 0 | | | | | | | | | | | | | | | | | | | | | | | | | | | | | | | | | |  |
| 56 | እቲ መቐመጢ ቦታ ፅርየቱ ዝሓለወ ኾይኑ ካብ ኣናፁ፣ድድዕን ካልኦት ነብሳትን ዝተሓለወ ድዩ? | | እወ 1  ኣይፋሉን 0 | | | | | | | | | | | | | | | | | | | | | | | | | | | | | | | | | |  |
| መልሲ ወሃቢ ንዝሃብኹም ግዘ ኣመስጊንኩም ተሰናበቱ፡፡  **መረዳእታ ኣካቢ፡ መረዳእታ ሰብሳቢት፡ ንመልሲ ወሃቢት ዝሕተቱ ሕቶታት ወዲእኺ ኣለኺ። እንተኮነ ግና ዝስዕቡ ከልተ ሕቶታት ብኣኺ ዝምልኡ ኣለው።** | | | | | | | | | | | | | | | | | | | | | | | | | | | | | | | | | | | | | |
| **ፅንዓት ዝካየደሉ ቦታን ውፅኢት ቃለመሕትትን** | | | | | | | | | | | | | | | | | | | | | | | | | | | | | | | | | | | | | |
| S | ጥቓ ኣፍደገ መእተዊ ናይቲ ትካል ብምን ንባብ ጂፒኤስ መዝግብ/ቢ፡፡    ኣኩዩረሲ ትሕቲ 6ሜ ምስኮነ ንባብ ጂፒኤስ መዝግብ/ቢ፡፡  **ንባብ ጂፒኤስ ዝውሰድ ናብ ደገ (ካብ ግቢወፃኢ) ጥራሕ እዩ፡፡** | | *ኣጠቓቕማ መምርሕታት ብ ODK ሶፍትዌር ቐሪብልኩም ኣሎ፡፡:*  መመዝገቢ ንባብ ጂፔኤስ | | | | | | | | | | | | | | | | | | | | | | | | | | | | | | | | | |  |
| S a | መእተዊ ኣፍደገ ናይቲ ትካል ፎቶ ንምልዓል ፍቓድ ሕተት/ቲ  ፎቶ ንኽተልዕሉ ፍቓድ ረኺብካኩም ዶ? | | እወ 1  ኣይፋሉን 0 | | | | | | | | | | | | | | | | | | | | | | | | | | | | | | | | | | ኣይፋሉን ተኾይኑ ናብ T ሕለፍ/ፊ |
| S b | ኣብቲ ዝተልዓለ ፎቶ ዋላ ሓደ ሰብ ዘይምእታው ኣረጋግፁ | | *ኣጠቓቕማ መምርሕታት ብ ODK ሶፍትዌር ቐሪብልኩም ኣሎ፡፡:*  ፎቶ ኣልዕሉ  ካብቲ ዝተልዓለ ፎቶ ዝሓሸ ምረፁ | | | | | | | | | | | | | | | | | | | | | | | | | | | | | | | | | |  |
| T | ውፅኢት ቃለመሕትት ግልጋሎት ወሃብቲ ትካላት ምልኡ | | ተጠናቒቑ 1  Not at facility 2  ንኻልእ ግዘ ተመሓላሊፉ 3  ፍቓደኛ ኣይኮኑን 4  ብዝተወሰ መልክዑ ተጠናቒቑ 5  ካልእ 6 | | | | | | | | | | | | | | | | | | | | | | | | | | | | | | | | | |  |
